# Dynamics of (Hetero)aryl Motifs: An Integrative Approach To Study the Conformational Landscape in Macrocycles

**DOI:** 10.1021/jacs.6c09765

**Published:** 2026-07-22

**Authors:** Anton F. Ketzel, Matthew Diamandas, Xiao-Lu Li, Yang Daniel Ou, Xinxiang Lei, Andrei K. Yudin, Han Sun

**Affiliations:** a Research Unit of Structural Chemistry & Computational Biophysics, 28417 Leibniz-Forschungsinstitut für Molekulare Pharmakologie, Berlin 13125, Germany; b Institut für Chemie, Strukturelle Chemische Biologie und Cheminformatik, Technische Universität Berlin, Berlin 10623, Germany; c Davenport Research Laboratories, 7938 University of Toronto, 80 St. George Street, Toronto, Ontario M5S 3H6, Canada; d State Key Laboratory of Natural Product Chemistry, Lanzhou Magnetic Resonance Center, College of Chemistry and Chemical Engineering, Lanzhou University, Lanzhou 730000, China

## Abstract

Macrocyclic peptides are emerging as a powerful therapeutic modality owing to their potential oral bioavailability and capacity to engage targets long considered undruggable. The introduction of aromatic heterocycles into macrocycles further expands this structural space by imparting distinct conformational preferences. However, accurate determination of the solution-state structures and dynamics of the resulting molecules remains challenging. Here, we integrate complementary isotropic and anisotropic NMR observables with enhanced sampling simulations and density functional theory (DFT) calculations to systematically investigate aryl- and heterobiaryl-containing cyclic peptides in different solvent systems. Our results based on residual dipolar coupling (RDC) measurements reveal conformational dynamics of macrocycles and significantly extend the knowledge gained by conventional NMR analysis based on nuclear Overhauser effects (NOEs) by more faithfully capturing the solution-state ensembles. Our methodology enables conformational analysis in both fast- and slow-exchange regimes on the NMR time scale. Notably, up to three interconverting backbone conformers at the DFT level are required to fully reconcile the experimental data for each ring system. We identify aryl and heterobiaryl motifs as structural elements governing the conformational landscape in our systems, modulating the populations of multiple thermodynamically accessible states characterized by distinct intramolecular hydrogen-bonding networks and unusual backbone geometries. Together, this integrative NMR-computational framework provides a precise and general strategy for resolving complex conformational ensembles of macrocycles, which should inform the objectives for synthetic modification and pave a way for the structure-based rational design of next-generation peptide therapeutics.

## Graphical abstract

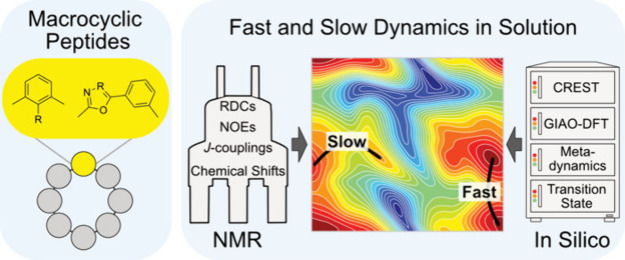

## Introduction

Cyclic peptides have emerged as an important class of therapeutics, with an increasing number of approvals per year. They are particularly attractive for targeting protein–protein interactions, for which the identification of potent small molecule inhibitors remains challenging.^1–5^ Compared with their linear counterparts, cyclic peptides exhibit enhanced resistance to proteolysis and, in favorable cases, can adopt “chameleonic” conformations that facilitate cell entry.^6–9^ To further expand the conformational and chemical space of conventional peptides, the incorporation of aromatic and heteroaromatic motifs into the peptide backbone has been extensively explored.^10^ The corresponding macrocycles often display increased rigidity and unique hydrogen-bonding capabilities.^11–15^ However, accurately characterizing their dynamic solution-state conformations remains challenging, partly due to complex electronic and steric features that are difficult to capture using classical computational approaches alone.^16^ This limitation currently hampers the structure-based rational design of macrocyclic constructs for drug discovery.^4,14^ In particular, the unpredictability of so-called activity cliffs, which are large drops of activity or permeability due to minor chemical changes,^17^ remains difficult to rationalize when structural data are sparse or when low-resolution structures are used for optimization.^18^

Solution-state NMR remains the state-of-the-art experimental method for determining the conformations of cyclic peptides and other macrocyclic compounds. Conventional approaches primarily rely on isotropic NMR observables, such as nuclear Overhauser effects (NOEs) or rotating-frame Overhauser effects (ROEs), and are often complemented by temperature coefficients. These experimental parameters are typically combined with molecular dynamics (MD) simulations to reveal the solution-state conformations of the molecules.^9,11–13,19–22^ However, standard force fields are often not well parametrized for noncanonical heterocycles.^23^ Moreover, conformational analysis based solely on NOE-derived restraints can be problematic for flexible systems, because NOEs report only on time-averaged interproton distances.^19^ As a result, structural models may converge on a single intermediate state that satisfies these averaged constraints but does not reflect the multitude of thermodynamically populated conformers in solution. While NOE/ROE build-up curves, often referred to as exact NOE analysis,^24^ can improve the accuracy of distance restraints by mitigating the effects of spin diffusion, it often requires a sufficient number of unambiguous cross-peaks to reliably define the conformational ensemble of flexible systems.^20^

In this context, anisotropic NMR parameters have significantly advanced the structural elucidation of organic molecules and peptides in solution, enabling more accurate characterization of their conformations and dynamic states.^30–32^ Residual dipolar couplings (RDCs), for example,^33–40^ provide long-range orientational restraints that are independent of interatomic distance, making them powerful reporters of the global molecular shape and the relative orientation of individual peptide subunits. In cyclic peptides, the application of RDCs has indeed substantially improved conformational characterization.^37–45^ Several studies have revealed solvent-dependent conformational ensembles, indicating the “chameleonic” structural adaptability of complex molecules, which may contribute to membrane permeability.^8,9,46–49^ In this work, we extended anisotropic NMR-based approaches for the accurate conformational characterization of a series of cyclic peptides containing diverse aryl^12^ and heterobiaryl^11^ backbone linkages (Figure 1a) in polar protic (methanol) as well as polar aprotic (DMSO) solvent systems. Particularly, we systematically integrated independent isotropic and anisotropic NMR observables, including chemical shifts, scalar couplings, ROEs, and RDCs, and validated the resulting structural ensembles using temperature coefficients. Computationally, in contrast to the MD-based approaches, we employed the conformer–rotamer ensemble sampling tool (CREST),^25,26^ followed by the automated commandline energetic sorting (CENSO)^27^ approach to generate pools of energetically accessible conformers using density functional theory (DFT). These conformers were subsequently cross-validated and selected based on their agreement with experimental NMR data. The present work describes an integrative approach for challenging macrocycles that populate dynamic conformational ensembles spanning both fast and slow exchange regimes on the NMR time scales (Figure 1c).

**Figure 1:**
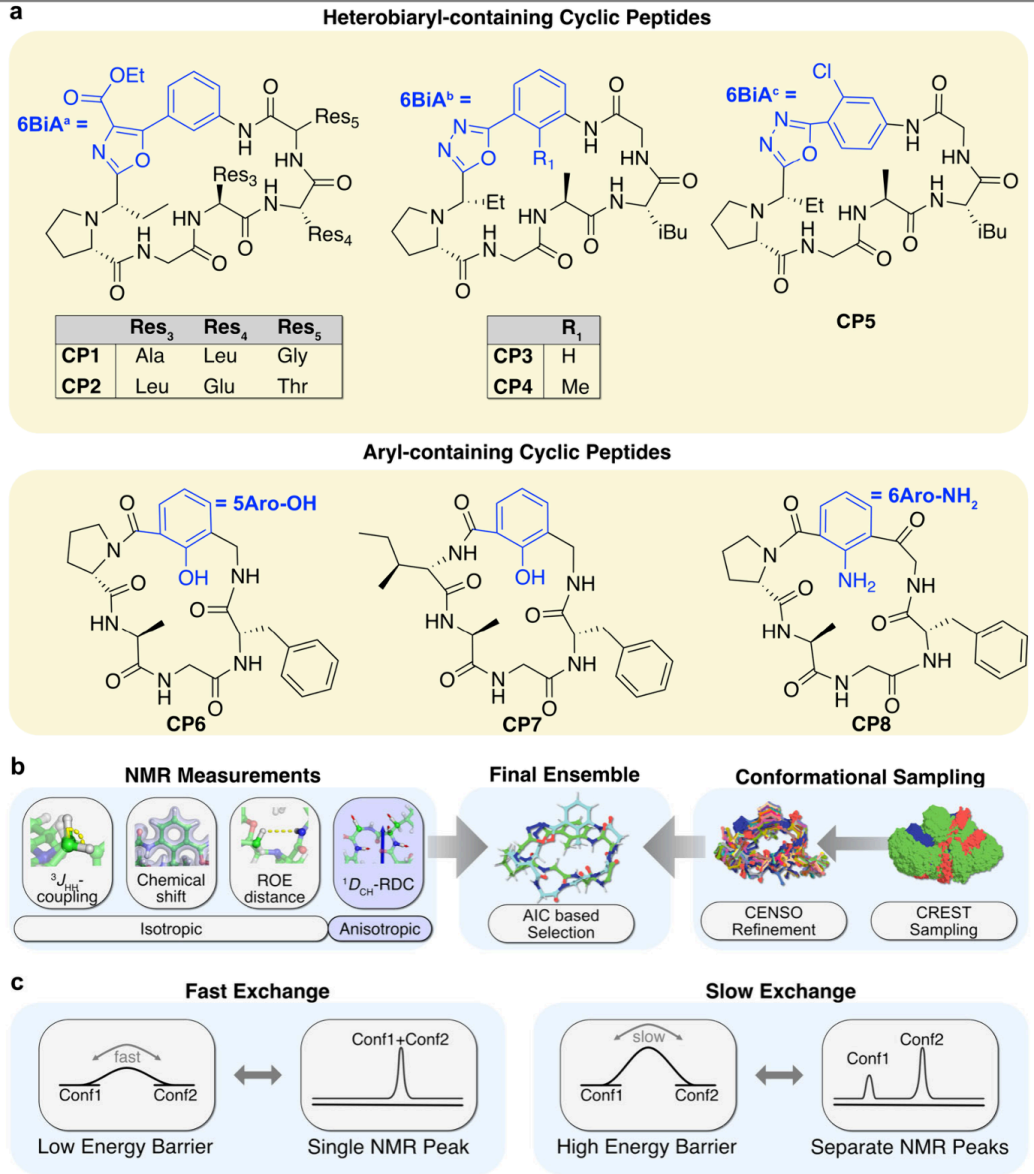
Overview of the cyclic peptides investigated and the overall procedure for conformational analysis. (a) Chemical structures of the cyclic peptides investigated in this study. Backbone motifs not found in canonical amino acids are highlighted in blue and labeled with the abbreviations used throughout the text. (b) Schematic representation of the conformational analysis workflow, combining state-of-the-art conformational sampling techniques (CREST^25,26^ /CENSO^27^) with NMR-guided conformer selection using Stereofitter.^28^ AIC stands for Akaike information criterion.^29^ (c) Illustration for fast and slow exchange on the time scale of NMR experiments.

## Results and Discussion

### Rationale for the Selection of Heterocyclic Peptides for NMR Analysis

To systematically investigate the determinants of conformational dynamics, we selected a collection of eight recently reported cyclic peptides featuring either heterobiaryl (**CP1**–**CP5**)^11^ or aryl (**CP6**–**CP8**)^12^ backbone linkages (Figure 1a). This set was designed to disentangle the contributions of amino acid sequence from linkage identity. Specifically, **CP1** and **CP2** share the same heterobiaryl linkage but differ in amino acid sequence, allowing for an assessment of influence of amino acid side-chains. Conversely, the subseries **CP1** and **CP3**–**CP5** maintain an identical sequence while varying the heterobiaryl linkage, thereby isolating the influence of the backbone heterocyclic motif. The phenol-containing cyclic peptides **CP6** and **CP7** were included to probe the effect of amino acid composition on the backbone conformation within the aryl series. Although these peptides differ by only a single residue (Pro in **CP6** vs Ile in **CP7**), they exhibit markedly different NMR behavior: **CP7** displays multiple sets of signals, indicative of multiple exchanging conformations at slow NMR time scales, whereas **CP6** shows only a single set. Finally, **CP8** was selected to evaluate the impact of the aniline linkage compared to the phenol series. All eight cyclic peptides are soluble in DMSO, while only the heterobiaryl CPs (**CP1**–**CP5**) are additionally soluble in methanol. To evaluate the applicability of our approach in different polar solvents for the conformational analysis of the heterobiaryl CPs, **CP1** and **CP5** were measured in DMSO, while the spectra for **CP2**–**CP4** were acquired in methanol. The aryl CPs (**CP6**–**CP8**) were measured exclusively in DMSO due to their limited solubility in methanol. Complete signal assignments were established for the eight peptides using standard isotropic NMR experiments (Tables S1–S9), with key HMBC and ROESY correlations shown in Figures S1–S9.

### Integrative NMR/DFT Workflow for Ensemble Conformational Analysis Using Isotropic and Anisotropic NMR Parameters

To resolve the backbone dynamics of **CP1**–**CP8** with high fidelity, we employed a unified DFT-based framework that integrates diverse isotropic and anisotropic NMR observables, different from our previous approach that relied primarily on ROE-derived distances combined with MD simulations.^11,12^ Specifically, ^13^C chemical shift, ^3^ *J*_NH‑Hα_-couplings, ROE-derived distances, and ^1^ *D*_CH_-RDCs, were integrated into a simultaneous fitting protocol, where structural ensembles were selected based on optimal agreement between experimental observables and back-calculated data using the Akaike information criterion (AIC)^29^ within Stereofitter.^28^

Although ^1^H chemical shifts have been extensively used as conformational restraints,^53–55^ they yielded markedly higher AIC values than all other observables in our analysis and were therefore excluded from the final ensemble fitting (Supporting Information Figure 21). This discrepancy likely arises from the limited ability of implicit-solvent DFT calculations to accurately capture specific solute–solvent interactions, which can have a pronounced impact on proton chemical shifts. To ensure thermodynamic relevance, the candidate pool was strictly limited to minima identified via CREST^25,26^ enhanced sampling and subsequently refined at the DFT level using CENSO.^27^ Using this NMR/DFT workflow, we found that the conformational behavior of all eight cyclic peptides is best described by ensembles comprising up to three conformational states (Table 1) rather than a single static structure. For all peptides, the resulting ensembles show good agreement with the experimental data (Figures S10–S20), reflected by low AIC values (18–74) and low Cornilescu *Q*-factor^50^ for the RDC fitting alone (0.05–0.15). To further assess the quality and contribution of individual NMR observables to the determination of the final ensemble, we used **CP1** as a representative example and evaluated ensembles generated from different combinations of NMR parameters (Figure S21). Notably, although each observable yields markedly different ensembles when used in isolation, the addition of any single observable to the RDC data resulted in only minor changes to the resulting ensemble. This result underscores the dominant contribution of the RDCs to the selection of final ensembles. We also noted that comparable fits were obtained for RDC data sets measured in different alignment media (AAKLVFF and OPA) and solvent systems (methanol and DMSO), indicating that both alignment media provide high-quality RDC data for macrocyclic compounds across these solvent conditions.

**Table 1:** Summary of the NMR Measurement Conditions and the Number of NMR Data Used for the Conformational Analysis

cyclic peptide	solvent	alignment medium	number of backbone RDCs	number of backbone ^13^C shifts	number of backbone ^3^ *J*_NH‑Hα_	number of backbone ROE distances	AIC^a^ (all data)	*Q*-factor^b^ (RDC)	ensemble size
**CP1**	DMSO	OPA^c^	12	20	2	10	33	0.08	3
**CP2**	MeOH	AAKLVFF^d^	10	19	5	18	45	0.15	3
**CP3**	MeOH	OPA^c^	12	18	5	11	67	0.13	3
**CP4**	MeOH	AAKLVFF^d^	11	20	4	6	50	0.15	3
**CP5**	DMSO	OPA^c^	11	19	5	12	74	0.10	3
**CP6**	DMSO	OPA^c^	9	11		7	18	0.13	2
**CP7.1** ^e^	DMSO	OPA^c^	10	16	4	8	34	0.07	2
**CP7.2** ^e^	DMSO	OPA^c^	7	15	4	10	49	0.05	1
**CP8**	DMSO	OPA^c^	10	15	2	18	22	0.09	2

a Akaike information criterion^29^ as implemented in Stereofitter.^28^

b Cornilescu quality (*Q*) factor^50^ for RDC fitting.

c OPA is an amphiphilic lipopeptide-based alignment medium that is compatible with multiple organic solvents.^51^

d AAKLVFF is an oligopeptide-based alignment medium that is compatible with methanol.^52^

e These are separate peak sets of **CP7**, indicating slow conformational exchange.

### Conformational Analysis of CP1: Ensemble vs Single State

We examined more closely the NMR/DFT-derived ensemble of **CP1** in comparison to the previously reported ROE/MD-based structure (Figure 2). Fitting the complete set of isotropic and anisotropic NMR observables to the single ROE/MD structure resulted in an AIC value of 215, indicating that this model does not adequately describe the full experimental data set (Figure 2b). In contrast, the best single conformer selected from the NMR/DFT pool markedly improved the fit, reducing the AIC value to 113. Inclusion of a second conformer further decreased the AIC value substantially to 39, while a three-state ensemble led to a slight additional improvement (AIC = 33). Notably, addition of further conformers did not further improve the fit, indicating that a two- or three-state ensemble is sufficient to capture the experimentally observed conformational landscape.

**Figure 2:**
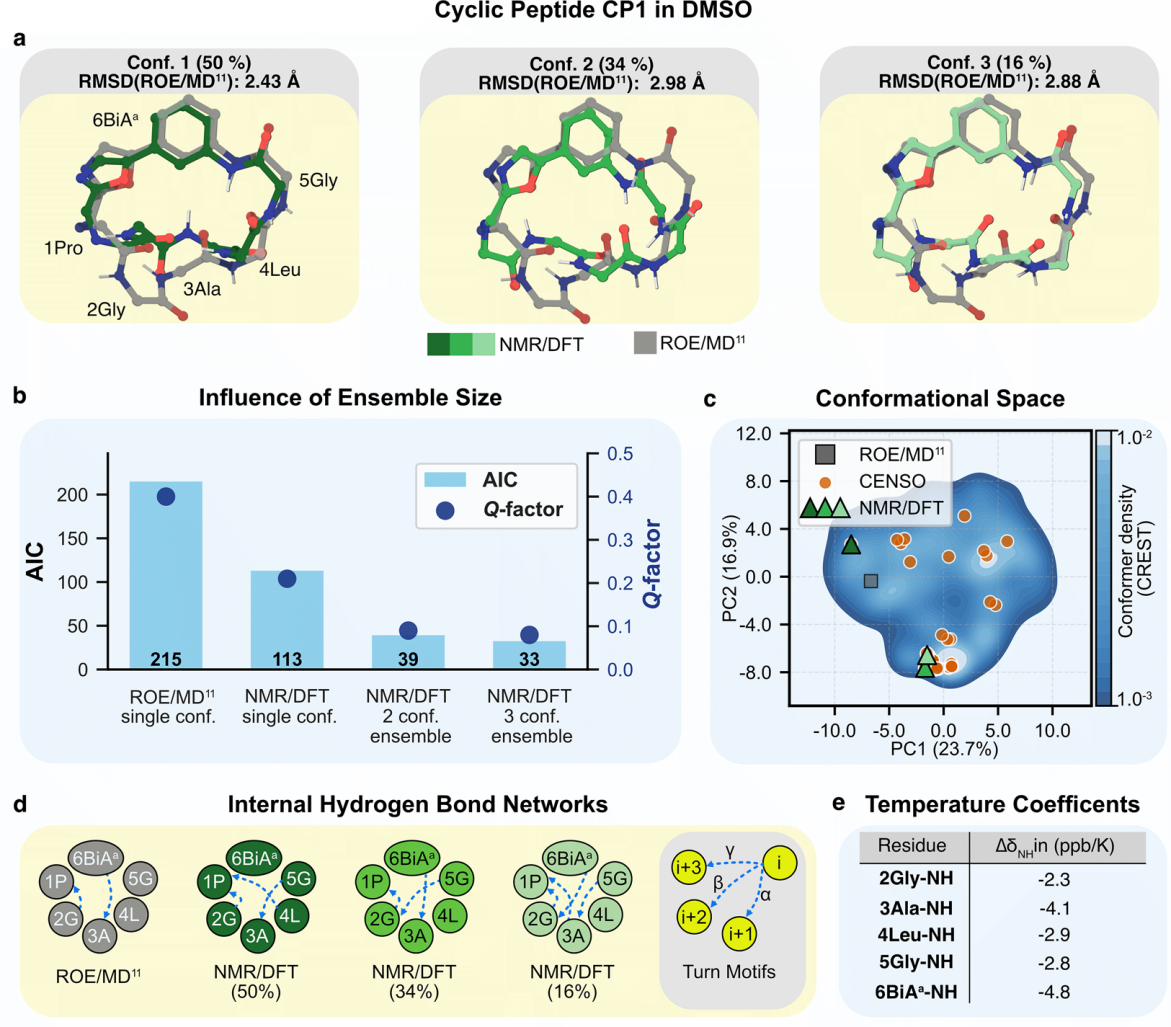
(a) Conformational ensemble of **CP1** at 300 K in DMSO determined using Stereofitter^28^ (green, with darker shades reflecting higher population), superimposed on the previously reported ROE/MD conformer^11^ (gray). (b) Influence of the ensemble size on the agreement between the structural models and the experimental NMR data. (c) PCA of backbone atom coordinates from the CREST^25,26^ ensemble (blue kernel density) and CENSO-optimized structures^27^ (orange dots) for the cyclic peptide **CP1**. For each principal component the percentage of variance is shown in parentheses. Torsion-based PCA was explored as an alternative using **CP1** as an example (Figure S22). However, Cartesian coordinate-based PCA was selected for the current analysis as it more effectively captures differences in overall molecular shape. (d) Schematic comparison of the internal hydrogen bonding patterns observed in the ROE/MD conformer and in the conformers of the NMR/DFT-derived ensemble. Hydrogen bonds are depicted going from the donor to the acceptor. (e) Amide proton temperature coefficients determined from variable-temperature NMR measurements in DMSO over the temperature range of 295–319 K.

When superimposing the NMR/DFT-derived three-state ensemble of **CP1** with the previously reported ROE/MD single static structure, substantial structural differences are observed, with RMSDs ranging from 2.4 to 3.0 Å (Figure 2a). To further compare these conformers with the computationally predicted conformational landscape, we performed principal component analysis (PCA) on the backbone atoms (Figure 2c).

The ROE/MD model (dark grey square) occupies a region of conformational space distinct from both the thermodynamically favored DFT minima (orange dots) and the final experimentally restrained ensemble (green triangles) (Figure 2c). Notably, although enhanced sampling with CREST initially accessed conformers close to the ROE/MD structure, the DFT-optimized ensemble shifted away from this region. This observation suggests that the previously determined ROE/MD structure of **CP1** likely represents a higher-energy intermediate rather than a significantly populated solution state conformation. In addition, the NMR/DFT-derived ensemble is further supported by analysis of the intramolecular hydrogen-bond (IMHB) network (Figure 2d/e). Analysis of amide temperature coefficients obtained from variable-temperature NMR (Δδ/Δ*T* > −4.0 ppb/K)^57^ supports the presence of at least three IMHBs in **CP1**. Notably, two of the three conformers in the NMR/DFT ensemble feature a hydrogen bond involving the 6BiA^a^–NH group, even though this residue exhibits a temperature coefficient of −4.8 ppb/K, which lies slightly outside the conventional cutoff used to classify hydrogen-bonded amide protons. This interaction was however also captured in our previous static ROE/MD model, where it forms a γ-turn motif (6BiA^a^–NH/3Ala–CO). We therefore previously proposed that the empirical temperature-coefficient threshold established for canonical amino acids may not be directly transferable to the heterocyclic 6BiA^a^–NH residue.^11^ More importantly, while the static ROE/MD model revealed only an additional endocyclic amine hydrogen bonding interaction (2Gly–NH/1Pro–N; here endocyclic and exocyclic denote hydrogen-bond acceptors located on the peptide backbone versus on a side chain, respectively), the NMR/DFT ensemble provides a more consistent interpretation of the experimental data by distributing all four hydrogen-bonding interactions across three interconverting conformational states. The dominant conformer (50%) retains the endocyclic amine hydrogen bond of ROE/MD but introduces a γ-turn involving 4Leu-NH and another β-turn from 5Gly-NH, while the second conformer (34%) adopts an alternative γ-turn at 5Gly-NH but shows the same 6BiA^a^–NH hydrogen bond as ROE/MD. The minor conformer (16%) exhibits an extensive IMHB network but lacks the 4Leu-NH interaction. It should be noted that no single conformer simultaneously satisfies the complete hydrogen-bonding pattern inferred from experiment, while only the ensemble description recapitulates the full set of experimentally indicated interactions.

### Fast *cis*/*trans*-Proline Isomerization in Aryl-Linked CP Series

Extending the NMR/DFT analysis to the aryl-linked peptides **CP6** and **CP8** further revealed that both macrocycles are best described by a two-state ensemble comprising *cis*- and *trans*-proline conformers (Figure 3a). When compared with the previously determined ROE/MD single-structure models, both conformers of **CP8** deviate substantially from the ROE/MD structure, with backbone RMSDs of 2.1–2.8 Å. In contrast, for **CP6** the major conformer (56%) closely resembles the ROE/MD model (0.4 Å RMSD). However, inclusion of a second minor conformer (2.4 Å RMSD) is required to satisfy the full set of experimental data. Additionally, we noted that incorporation of this two-state model markedly improves agreement with the experimental data, reducing the AIC from 155 to 18 for **CP6** and from 742 to 22 for **CP8** relative to a single-conformer fit (Figure 3c). Despite the presence of two conformational states, only a single set of NMR signals is observed for each peptide, indicating that the *cis* and *trans* proline conformers interconvert rapidly on the NMR time scale. This behavior contrasts with canonical peptides, where proline *cis*/*trans* isomerization is typically slow^58,59^ and therefore produces separate sets of resonances.

**Figure 3:**
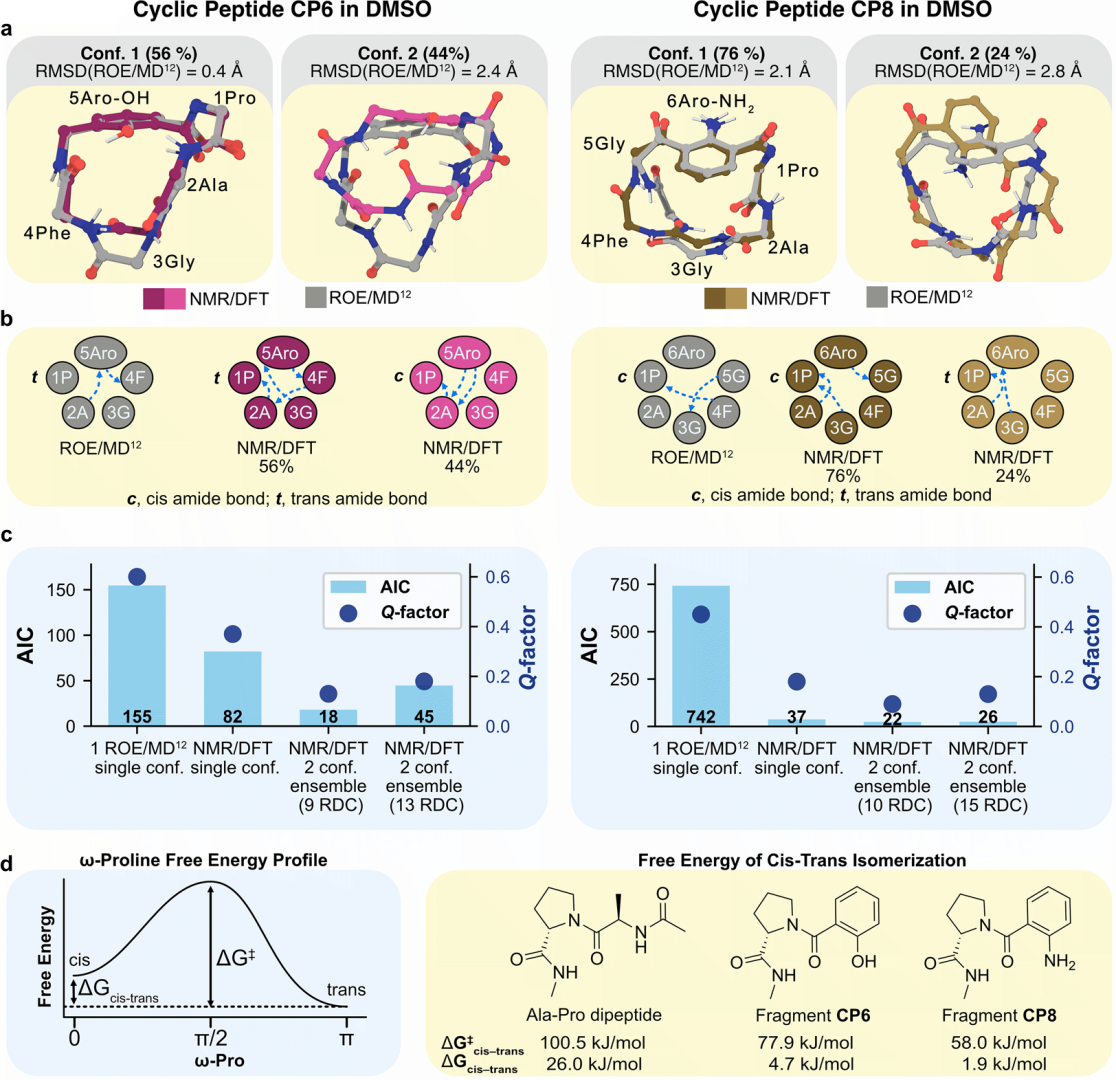
(a) NMR/DFT-derived conformational ensemble for **CP6** (purple) and **CP8** (brown) at 300 K in DMSO, superimposed on the previously reported ROE/MD conformers^12^ (gray). Darker shades indicate higher population in the ensembles. (b) Schematic overview of the internal hydrogen bonds of the ROE/MD and NMR/DFT ensembles. (c) Quality of NMR data fit as a function of ensemble sizes. The impact of including side-chain RDCs is also shown. (d) Schematic free energy profile of the proline ω-angle, together with relative transition state barrier free energies determined using NEB-CI^56^ and energies calculated at the PW6B95-D4/def2-TZVP/SMD­(DMSO) level of theory.

To validate the robustness of the ensemble description, we first incorporated additional side-chain RDCs from rigid moieties into the fitting procedure. Inclusion of these observables did not significantly deteriorate the fitting quality and continued to support the *cis*/*trans* conformer mixture (Figure 3c). Furthermore, the temperature range investigated in our variable-temperature NMR (301–319 K), did not show separation or coalescence of NMR signals, suggesting stable fast-exchange in this temperature range. Substantial lowering of the temperature was hindered by DMSO’s high freezing point. We next examined the Cβ/Cγ chemical shift difference, a parameter typically used to distinguish *cis*- and *trans*-proline in canonical peptides.^60^ However, for **CP6** and **CP8** this metric yielded inconclusive and contradictory results. DFT calculations of the proline chemical shifts within these heteromacrocycles rationalize this observation, revealing that the Cβ/Cγ shift difference is substantially perturbed relative to canonical peptides and therefore loses its diagnostic power in this context (Figure S23).

Finally, DFT calculations of the *cis*/*trans* isomerization pathway for model fragments of **CP6** and **CP8**, benchmarked against a canonical Ala-Pro dipeptide, provide a mechanistic explanation for the observed fast exchange (Figure 3d). These calculations identify the aryl linkage as the key structural element governing the dynamics: its presence substantially lowers the isomerization barrier (Δ*G* ^‡^_trans–cis_) and reduces the thermodynamic free energy gap between the two states (Δ*G*_trans–cis_). The lowering of the transition state can be rationalized in two ways: first, the transition state geometries show that for **CP6** and **CP8** the carbonyl group is rotating into the plane of the benzene ring, allowing larger electronic delocalization (Figure S24). Furthermore, energy decomposition analysis suggests higher steric strain in the ground state of **CP6** and **CP8** effectively lowering the transition barrier compared to the Ala-Pro dipeptide. Together, these effects rationalize the unusually rapid *cis*/*trans* interconversion observed at 300 K in DMSO for **CP6** and **CP8**. Collectively, these results reveal an unexpected mode of conformational dynamics introduced by aryl-linked macrocycles.

### Backbone Linkage Identity Dominates Conformational Preferences

To understand the determinants of the overall conformational space of the five heterobiaryl containing peptides, we systematically compared the determined ensembles using PCA of the backbone coordinates (Figure 4a). This analysis captures the largest structural variations among the peptides and thus reflects differences in their overall backbone shape. For example, principal component (PC) 1 describes the transition between flat and twisted conformations, whereas PC2 captures the transition from a clamshell-like to a more circular conformation (Figure 4a).

**Figure 4:**
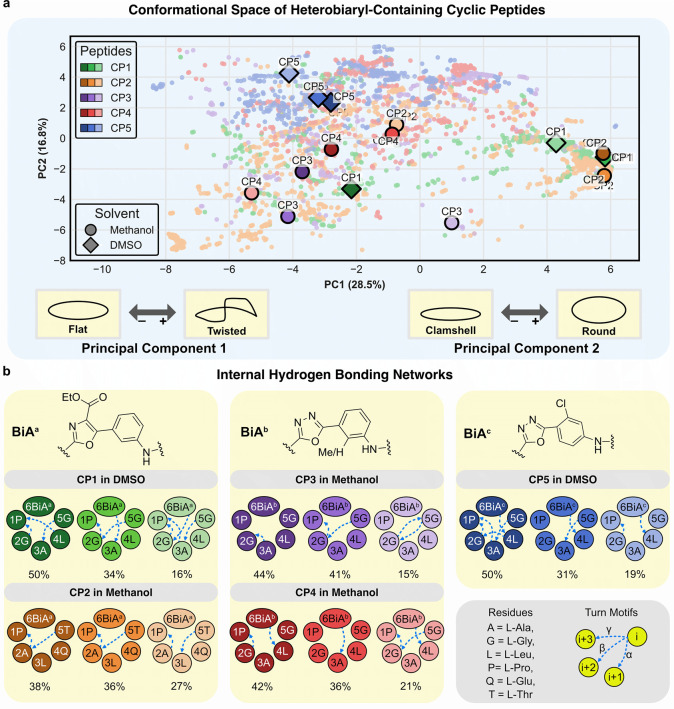
(a) Principal component analysis (PCA) of backbone-atom coordinates from the CREST (GFN2-xTB/ALPB) ensembles for **CP1**–**CP5** (PC1, 28.5%; PC2, 16.8%). Small points represent CREST^25,26^ conformers, and the larger circles (or diamonds if measured in DMSO) indicate the NMR/DFT determined conformers. Colors denote the different CPs investigated, and darker shades reflect higher population in the ensemble. Conformational dynamics described by PC1 and PC2 are schematically illustrated. (b) Schematic overview of the internal hydrogen bonds for the NMR/DFT ensembles of **CP1**–**CP5**.

Notably, peptides containing distinct heterobiaryl linkages (BiA^a^ in **CP1**/**CP2** vs BiA^b^ in **CP3**/**CP4** vs BiA^c^ in **CP5**) occupy mostly discrete, nonoverlapping clusters in the PC1-PC2 projection.^15,61^ In contrast, variations in the amino acid sequence or solvent environment exert only a secondary influence. For instance, **CP1** and **CP2**, which differ both in sequence and solvent (DMSO vs methanol), display similar backbone ensembles with largely overlapping PCA distributions. Despite these global similarities, important differences emerge at the level of local dynamics as reflected in their internal hydrogen bonding networks. (Figure 4b). Both the **CP1** and **CP2** ensembles maintain the endocyclic amine hydrogen bond (2Gly-NH/1Pro-N), yet their secondary turn motifs differ substantially. While the conformational space of **CP2** is largely dominated by closely related conformers, with highly similar IMHBs, the **CP1** ensemble populates conformers that are well separated in PC1, which is additionally accompanied by a greater diversity of turn motifs. The subtle impact of peripheral modifications is further exemplified by the pair **CP3**/**CP4** (Figure 4b). Here, a single methyl substitution on the biaryl linkage preserves the main backbone topology [**CP3** (44%) and **CP4** (42%)], but significantly reshapes the energetic landscape of the minor states. Specifically, methylation shifts the secondary conformer observed in **CP3** (41%) to a less populated third conformer in **CP4** (21%). Moreover, the unique antiparallel β-sheet-like backbone observed as a minor species in **CP3** is completely absent in the methylated variant. Instead, **CP4** populates a distinct conformer (36%) that is structurally similar to the major conformer.

While prior studies assumed stable, constitutive endocyclic amine hydrogen bonds across all members of this series, our ensemble analysis reveals that for **CP3**–**CP5**, these interactions are transient, present in only 41–69% of the population. Thus, the internal hydrogen-bonding networks are far more dynamic than previously assumed, with β- and γ-turn motifs interconverting in equilibrium. Altogether, our results underscore that both global features indicated by the PCA coordinates and local hydrogen bonding dynamics are necessary to fully describe the dynamics of this class of cyclic peptides. The dominant role of the backbone linkage is further supported by the analysis of the aryl-linked series (**CP6**–**CP8**). Here, PCA reveals a generally more dispersed conformational space (Figure S25), consistent with a larger diversity of accessible states. Notably, the first principal component clearly separates the *cis*- and *trans*-proline conformers discussed above, suggesting that proline isomerization strongly influences the overall peptide shape. Within this set, **CP7** represents a particularly complex case, displaying a superposition of both fast and slow conformational exchange on the NMR time scale, which will be discussed more in detail below.

### Resolving Fast and Slow Conformational Dynamics in **CP7**

As mentioned above, slow conformational exchange in **CP7** was evident from the doubling of signals in the ^1^H spectrum, with an approximate population ratio of 87:13. To characterize the conformational landscape underlying these multi-timescale dynamics at atomic resolution, we applied our NMR/DFT workflow to both the major and minor peak sets. This analysis yielded high-confidence structural models with low AIC values for both populated states in the NMR spectra (Table 1): a two-conformer fast-exchanging ensemble for the major peak set, and a single conformer for the minor set (Figure 5a). Notably, the most populated conformer (55%, dark-cyan) exhibits very low RMSD relative to the previously determined ROE/MD single-conformer model and shares key features, including the six-membered pseudo ring (5Aro-OH/5Aro-CO) and the β-turn motif (5Aro-NH/3Gly-CO). However, incorporation of a second conformer was required to quantitatively agree with the full set of NMR data (Figure S28). This second conformer (32%, medium-cyan) introduces an additional hydrogen-bond (2Ala-NH/5Aro-CO), while otherwise retaining the same IMHBs. In contrast, for the minor peak set (13%) a single conformer (light-cyan) was sufficient to describe the NMR data. This conformer is generally similar to the two conformers of the major peak set, but features a different β-turn involving 4Phe-NH and 2Ala-CO (Figure 5c). Given that this structural difference is relatively subtle, the observed slow exchange kinetics were unexpected.

**Figure 5:**
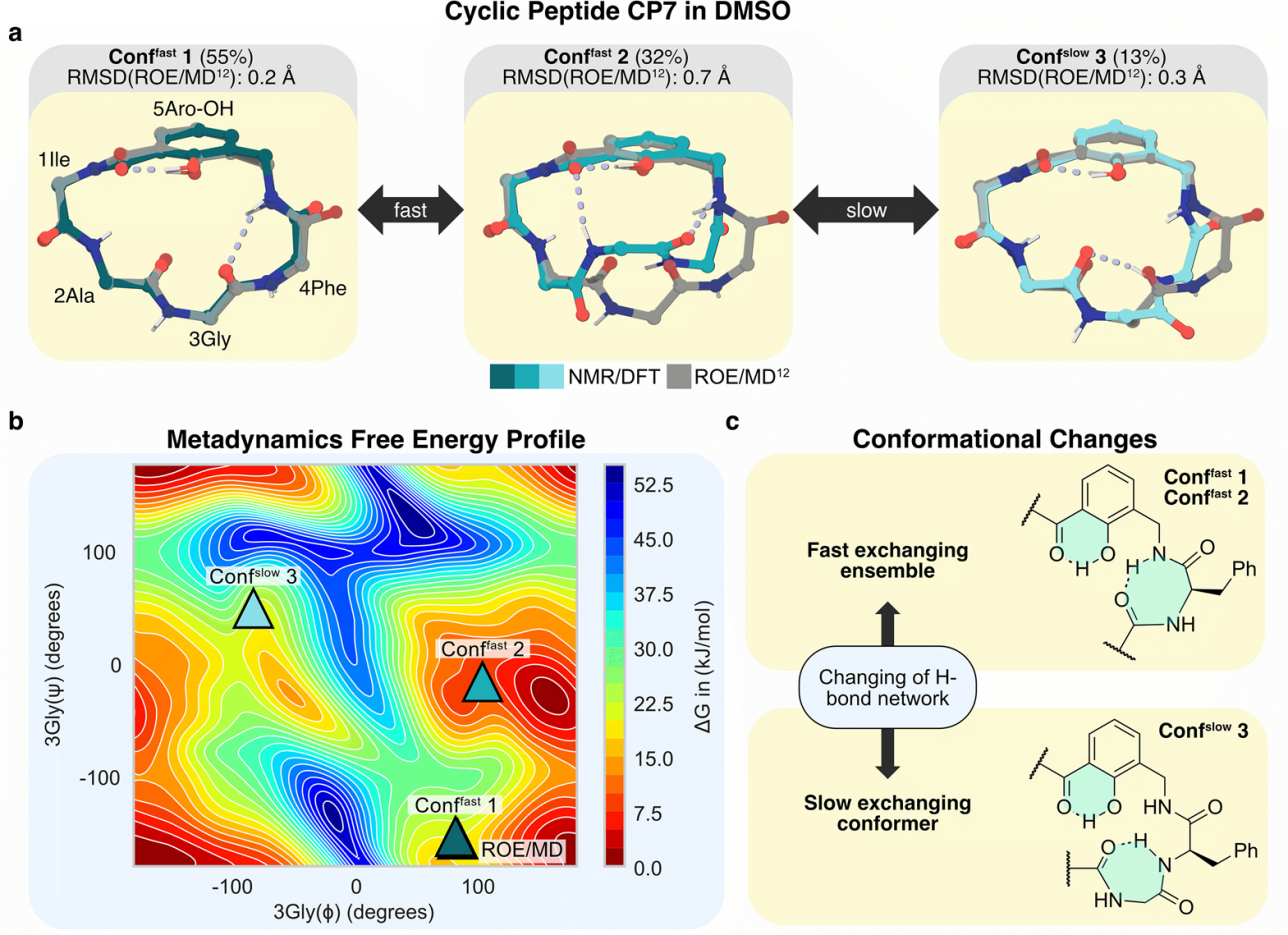
(a) Conformational ensemble determined for **CP7** in DMSO at 300 K using Stereofitter^28^ for the major (dark and medium cyan) and minor (light cyan) set of NMR signals. Internal hydrogen bonds are indicated. (b) Free energy (Δ*G*) landscape of **CP7** calculated by well-tempered metadynamics^62,63^ by biasing the angles of the residue 3Gly, using GROMACS 2022.5 ^64–70^ in conjunction with Plumed 2.9.^71–73^ The shown data are averaged over 4 × 1 μs metadynamics simulations in DMSO using the GAFF^74^ force field at 300 K and 1 bar; see Supporting Information Figure S39 for the convergence of the simulations. The conformers determined by the NMR/DFT as well as the ROE/MD^12^ approaches are indicated. (c) Schematic illustration of the conformational changes between the fast exchanging and slow exchanging conformers.

To rationalize the kinetics of the underlying conformational transitions, we employed well-tempered metadynamics simulations in explicit DMSO solvent, as unbiased MD simulations failed to sample the full conformational space of **CP7**. A bias was applied to the Φ and Ψ dihedral angles of the 3Gly residue, which showed the clearest separation between the fast-exchanging ensemble and the slow-exchanging conformer. The resulting free energy surface (Figure 5b) reveals three distinct energy minima, qualitatively matching the three NMR/DFT conformers.

Notably, the free-energy barrier separating the fast-exchanging conformer (dark- and medium-cyan) is considerably lower than the barrier isolating the slow-exchanging conformer (light-cyan), which agrees very well with the experimental observation. We acknowledge that classical force fields have limitations in the quantitative interpretation of absolute barrier heights, particularly for systems that have not been extensively parametrized or validated.

## Conclusions

Accurate conformational characterization of macrocyclic peptides in solution is critical not only for structure-based drug design but also for understanding and optimizing their physicochemical properties. However, structural descriptions of heterocycle-containing peptides have largely relied on static models derived from conventional NMR restraints in combination with force-field-based simulations. These approaches often fail to capture the full extent of conformational heterogeneity, frequently collapsing highly dynamic systems into artificial time-averaged structures. In this study, we move beyond this traditional paradigm by integrating complementary isotropic and anisotropic NMR observables with enhanced conformational sampling and DFT-based refinement. Our integrative approach resolves previously inaccessible conformational states and yields ensemble descriptions that are substantially more consistent with experimental observables. Application of this framework to eight aryl- and heterobiaryl-linked cyclic peptides reveals that these macrocycles are best described as dynamic ensembles comprising up to three interconverting conformers at the DFT level. We note, however, that its application to considerably larger systems may be constrained by the computational demands of the DFT calculations. Recent advances in machine-learning force fields with near-DFT accuracy,^75–77^ offer a promising opportunity to overcome this limitation. Nevertheless, systematic benchmarking of these new force fields for the conformational analysis of flexible macrocycles remains to be established.

By considering a peptide series, our analysis identifies the backbone linkage motif as a common determinant of the global peptide shape, whereas the internal hydrogen-bonding patterns are largely unique to each cyclic peptide. Importantly, the ensemble description also captures fast interconverting *cis*/*trans* proline isomerization in the aryl-linked peptides, revealing an unexpected mode of conformational dynamics compared with canonical peptides. For one cyclic peptide, we identified the coexistence of both slow- and fast-exchanging conformers on the NMR time scale, a kinetic hierarchy that is consistent with the free-energy landscape obtained from metadynamics simulations. Recent conceptual advances in the field of macrocycles have highlighted the importance of underappreciated conformational interconversions, including homeomorphic switches.^78^ In this context, our findings are particularly relevant as the characterization of such processes relies heavily on NMR-derived structural assignments. On the basis of these examples, we showcase the limitations of relying solely on averaged NOE-derived distances for the conformational analysis of flexible macrocycles, especially when few NOE contacts are experimentally accessible. This underscores the value of complementary descriptors, such as RDCs. By providing these descriptors, our framework should now guide targeted synthetic modification strategies, enabling the evaluation of pharmacological properties by stabilizing conformational states that were previously overlooked.

## Experimental Methods

### Materials

The cyclic peptides investigated in this study have been synthesized following the procedure described previously for **CP1**–**5** ^11^ and **CP6**–**8**.^12^ Deuterated solvents MeOH-*d*_3_, MeOD-*d*_4_, DMSO-*d*_6_ have been used for the NMR measurements.

### NMR Measurements

NMR experiments have been performed on a 600 MHz Bruker AVANCE III spectrometer equipped with a 5 mm QCI cryoprobe. If not stated otherwise, all spectra were measured at 300 K (26.9 °C).

### Isotropic NMR Measurements

Each cyclic peptide (0.9–1.5 mg) was dissolved in 300 μL of DMSO-*d*_6_, MeOD-*d*_4_ or MeOH-*d*_3_. Standard 1D ^1^H- and ^13^C-spectra as well as 2D [^1^H,^1^H]-COSY, [^1^H,^1^H]-TOCSY, [^1^H,^13^C]-HMBC, [^1^H,^13^C]-HSQC, and [^1^H,^15^N]-HSQC experiments have been performed and used for signal assignment. Additionally, [^1^H,^13^C]-CLIP-HSQC^79^ and [^1^H,^15^N]-CLIP-HSQC were measured in the isotropic state as reference to measure the ^1^ *D*_CH_-RDCs. To measure distance restraints, ROESY or EASY-ROESY^80^ spectra have been measured with a mixing time of 80 ms in each case. Peak volumes were integrated and calibrated against the cross-peak of a geminal proton with assumed distance of 1.78 Å (Tables S10–S18). ROESY peaks have been additionally corrected for chemical shift dependence. Temperature coefficients have been measured using 1D ^1^H spectra in a temperature range for methanol of 288–315 K and for DMSO 295–319 K and analyzed using linear regression (Tables S19–S27). The ^3^ *J*_HH_ couplings were extracted from the ^1^H spectra at 300 K. All raw spectra are shown in the Supporting Information Figures S42–S146.

### Anisotropic NMR Measurements

After measurement of the isotropic spectra, alignment medium was added to the same samples to obtain the anisotropic state. Either the oligopeptide AAKLVFF^52^ (28.8 g/L) or the lipopeptide OPA^51^ (C_15_H_31_-CONH-V_4_K_3_–CONH_2_) (30 g/L in methanol and 43 g/L in DMSO) have been used (Table 1). Both form liquid crystalline phases in solution and the formation was monitored by the quadrupolar splitting of ^2^H signals of the solvent peak (Figure S26). ^1^ *D*_CH_- and ^1^ *D*_NH_-RDCs were obtained by measuring the splitting difference between the isotropic and anisotropic signals from the CLIP-HSQC spectra. In all cases, only marginal changes in the chemical shifts of the cyclic peptides were observed under the anisotropic condition compared to the isotropic state (Tables S1–S9), comparable in magnitude to those previously for rigid small molecules.^51^

### Conformational Sampling and DFT Calculations

For each cyclic peptide, conformational search was performed in CREST^25,26^ using GFN2-xTB^81^ and with implicit solvation using the ALPB­(solvent)^82^ model. Subsequent ensemble refinement was done in CENSO^27^ (Table S28) using DFT methods with different levels of accuracy. The final ensembles (Table S29) used for further investigation were geometry optimized using ORCA 5.0.4 ^83–85^ at the PW6B95-D4/def-TZVP/SMD­(solvent)^86–88^ level of theory, with NMR parameters calculated using ORCA 6.0.0 ^89^ at the r^2^SCAN0/pcSseg-2/SMD­(solvent)^90,91^ level of theory. The previously determined ROE/MD conformers were optimized using DFT at the same level of theory for better comparison. The choice of functional and basis set was guided by a small, nonexhaustive benchmark (Tables S30–S33). The chemical shieldings have been converted to chemical shifts, using custom linear scaling parameters (Figure S34 and Table S37).^41,92,93^ More detailed descriptions of the computational methods are provided in the Supporting Information, together with estimated computational times for each step (Table S35).

### Conformational Analysis Using Stereofitter

Conformational deconvolution was performed using MestreLabs Stereofitter 1.1.7 ^28^ based on AIC, which permits simultaneous fitting of a number of different isotropic and anisotropic observables. In contrast to our recent analysis of a canonical cyclic peptide^41^ where the NMR data was fitted separately, in the present study, the experimental data, comprising chemical shift, ROE-derived distances, ^3^ *J*_Hα‑NH_ couplings and ^1^ *D*_CH_-RDCs, were fitted simultaneously using either a single-conformer single tensor (SCST) or multi-conformer single tensor (MCST) approximation. Experimental uncertainties were estimated as 1.2 Hz (RDCs), 2 ppm (^13^C shifts), 0.5 Å (ROEs), and 0.5 Hz (*J*-couplings). The *J*-couplings have been analyzed using the Karplus equation from Pardi et al.,^94^ as also described previously.^37,38^ We noted that these parameters were originally calibrated for globular proteins and have not been specifically parametrized for macrocyclic peptides. In principle, a system-specific parametrization, for example based on DFT calculations, could further improve the agreement between experimental and calculated *J*-couplings.^95,96^ However, given the limited number of unambiguously assignable *J*-couplings available for each **CP1**–**CP8** (0–5), we expect this approximation to have only a minor impact on the resulting conformational ensembles. While ^1^ *D*_NH_-RDCs were initially considered, they were excluded from the final fitting processes due to poor spectral quality leading to significant fit deterioration. Comprehensive input files (Tables S36–S44), ensemble populations (Tables S45–S79), aggregated *Q*-factors (Table S80), correlation plots between experimental and back-calculated NMR data (Figures S10–S20) and final ensembles for each cyclic peptide (Figures S27–S33) are provided in the Supporting Information.

### Transition State Calculation Using DFT

Estimations of the transition state barrier for the *cis*/*trans* isomerization have been performed by DFT calculations using ORCA 6.0.0. Transition state energies of **CP6** and **CP8** including only the aryl-linkage and the proline residue were compared to a canonical Ala-Pro dipeptide. Each system was optimized with an ω dihedral angle of 0 (cis) and 180° (trans) at the GFN2-xTB/ALPB­(DMSO)^81,82^ level of theory. These structures were then employed in nudged elastic band (NEB-CI)^56^ calculations at the same level of theory; see the input in the Table S81. Geometry optimization and frequency calculations were performed at the PW6B95-D4/def2-TZVP/SMD­(DMSO) level of theory (Table S82). The frequencies were manually confirmed to show only a single imaginary frequency for the transition state and none for the relaxed states. Energy decomposition analysis was additionally conducted on the ground state and transition states structures at the PW6B95-D4/def2-TZVP/SMD­(DMSO) level of theory using the ETS-NOCV^97^ module in ORCA 6.1.

### Explicit Solvent MD and Metadynamic Simulations

Parameters for the cyclic peptide **CP7** have been generated for the GAFF^74^ force field. The NMR/DFT determined conformers were geometry optimized, and the RESP charges were calculated in Gaussian16 ^98^ at the HF/6-31*G level of theory (Figures S35–S37). GROMACS 2022.5 ^64–70^ compatible parameter files were generated from the RESP charges using antechamber. The GAFF parameters for DMSO have been generated accordingly. The peptide was solvated in a 3.09 nm × 3.09 nm × 3.09 nm simulation box filled with DMSO molecules. After energy minimization, the system was equilibrated for 1 ns as *NVT* ensemble, another 1 ns as *NPT* ensemble, and a final equilibration of 10 ns *NVT* with LINCS restraints on hydrogen-atoms at 300 K and 1 bar. The equilibrated starting from each NMR/DFT conformer have been used for 4x1 μs unbiased MD simulation (Figures S38–S40). The parameters of the major conformer were then selected for 1 μs well-tempered metadynamics simulations,^62,63^ biasing the 3Gly Φ and Ψ dihedral angles. Plumed v. 2.9 ^71–73^ was used to add a Gaussian bias of 1.2 kJ/mol every 500 steps with a 0.2 radians σ. The bias factor was set to 8. The simulations converged during the 1 μs simulation time (Figure S41) and were analyzed using a python script with MDanalysis.^99^

### Principal Component Analysis

The PCA analyses have been performed using standard python packages. For each peptide the coordinates of the backbone atoms of the CREST ensemble (30 kcal/mol energy window) have been used. The DFT optimized conformers as well as the ROE/MD conformers have been projected using the same principal components vectors. For the shared PCA analysis the same procedure has been carried out, but utilizing the CREST ensembles of each cyclic peptide simultaneously. For the analysis of the dynamics described by the first two principal components PC1 and PC2, backbone conformers have been generated with idealized +5 and −5 PC1 and PC2 separately. Images of these conformers are shown in Figures S147–S150.

## Supplementary Material



## Data Availability

All raw NMR spectra, as well as DFT and MD output files are deposited on Zenodo under the accession link 10.5281/zenodo.19909872.

## Notes

SI.1, full assignment of the cyclic peptides; SI.2, correlation between experimental and back-calculated NMR data; SI.3, impact of different NMR parameters on the analysis of CP1; SI.4, choice of coordinates for the PCA of CP1; SI.5, DFT calculated chemical shift for *cis*/*trans* proline; SI.6, transition state analysis of proline *cis*/*trans* isomerization; SI.7, PCA of aryl-containing cyclic peptides; SI.8, extraction of ROE-derived distances; SI.9, extraction of amide proton temperature coefficients; SI.10, residual dipolar coupling analysis; SI.11, conformational ensembles for CP2, CP3, CP4, CP5, CP6, CP7, CP8; SI.12, computational details for conformational sampling; SI.13, conformational analysis using Stereofitter; SI.14, computational details for predicting proline *cis*/*trans*-isomerization transition barrier; SI.15, molecular dynamics simulations; SI.16, experimental spectra; SI.17, visualization of the principal components; SI.18, additional literature used in the Supporting Information (PDF)

## #

Institute of Medical Sciences, The Second Hospital of Shandong University, Jinan 250033, China

## Abbreviations

AIC: Akaike information criterion
Ala: alanine
Aro: aryl-linkage
ALPB: analytical linearized Poisson–Boltzmann
BiA: biaryl-linkage
CENSO: command-line energetic sorting
CLIP: clean in-phase
Conf: conformer
COSY: correlation spectroscopy
CP: cyclic peptide
CREST: conformer-rotamer ensemble sampling tool
DFG: Deutsche Forschungs Gemeinschaft
DFT: density functional theory
DMSO: dimethyl sulfoxide
EASY-ROESY: efficient adiabatic symmetrized ROESY
FMP: Leibniz-Forschungsinstitut für Molekulare Pharmakologie
GAFF: generalized Amber force field
Gly: glycine
GROMACS: Groningen machine for chemical simulation
HF: Hartree–Fock
HMBC: heteronuclear multiple bond correlation
HPC: high performance cluster
HSQC: heteronuclear single quantum coherence
IMHB: intramolecular hydrogen bond
Leu: leucine
MD: molecular dynamics
MeOD: methanol
NEB-CI: nudged elastic band climbing image
NMR: nuclear magnetic resonance
NOESY: nuclear Overhauser effect spectroscopy
NSRC: National Science and Engineering Research Council of Canada
OPA: oligopeptide amphiphile
PCA: principal component analysis
Phe: phenylalanine
Pro: proline
*Q*(-factor): quality factor
RDC: residual dipolar coupling
RESP: restraint electrostatic potential
RMSD: root-mean square deviation
ROESY: rotating-frame Overhauser effect spectroscopy
RTG: research training group
SMD: solvation model based on density
TOCSY: total correlation spectroscopy
xTB: extended tight binding
