# Supplementary material for: Dynamics of (Hetero)aryl Motifs: An Integrative Approach To Study the Conformational Landscape in Macrocycles
Source: J Am Chem Soc. 2026 Jul 22;148(30):32582–94. doi: 10.1021/jacs.6c09765 (PMC13450417; doi:10.1021/jacs.6c09765)
Supplement: Supplementary file 1 [file ja6c09765_si_001.pdf]

Supporting Information

Dynamics of (Hetero)aryl Motifs: An Integrative Approach to Study the Conformational Landscape in Macrocycles

Anton F. Ketzel‡a,b, Matthew Diamandas‡c, Xiao-Lu Li[a], Yang Daniel Ou[c], Xinxiang Lei[e], Andrei K. Yudin[c*], Han Sun[a,b*]

[a] Research Unit of Structural Chemistry & Computational Biophysics, Leibniz-Forschungsinstitut für Molekulare Pharmakologie, Berlin 13125, Germany.
[b] Institut für Chemie, Strukturelle Chemische Biologie und Cheminformatik, Technische Universität Berlin, Berlin 10623, Germany.
[c] Davenport Research Laboratories, University of Toronto, 80 St. George St, Toronto, Ontario M5S 3H6, Canada.
[d] State Key Laboratory of Natural Product Chemistry, Lanzhou Magnetic Resonance Center, College of Chemistry and Chemical Engineering, Lanzhou University, Lanzhou 730000, China.

* E-Mail: andrei.yudin@utoronto.ca, hsun@fmp-berlin.de
‡ These authors contributed equally

SI.1: Assignments of the cyclic peptides CP1-CP8 3

SI.1.1: Assignment of cyclic peptide CP1 3

SI.1.2: Assignment of cyclic peptide CP2 5

SI.1.3: Assignment of cyclic peptide CP3 7

SI.1.4: Assignment of cyclic peptide CP4 8

SI.1.5: Assignment of cyclic peptide CP5 10

SI.1.6: Assignment of cyclic peptide CP6 12

SI.1.7: Assignment of cyclic peptide CP7 13

SI.1.8: Assignment of cyclic peptide CP8 17

SI.2 Correlation between experimental and back-calculated NMR data 18

SI.3 Impact of different NMR parameters on the analysis of CP1 28

SI.4 Choice of coordinates for the PCA of CP1 30

SI.5 DFT calculated chemical shift for *cis/trans*-proline 31

SI.6 Transition state analysis of proline *cis/trans*-isomerization 32

SI.7 PCA of aryl-containing cyclic peptides CP6–CP8 32

SI.8 Extraction of ROE-derived distances 34

SI.9 Extraction of amide proton temperature coefficients **39**

SI.10 Residual dipolar coupling analysis **44**

SI.11 Determined conformational ensembles for CP2, CP3, CP4, CP5, CP6, CP7, CP8 .... **46**

SI.12 Computational details for conformational sampling **52**

SI.12.1 General procedure of conformational sampling *52*

SI.12.2 DFT benchmark study *66*

SI.12.3 Linear scaling parameter to convert chemical shielding to chemical shift *70*

SI.12.4 Computational times and resources *71*

SI.13 Conformational analysis using Stereofitter **72**

SI.13.1 Input files for Stereofitter *72*

SI.13.2 Results from Stereofitter *84*
SI.13.2.1 Ensemble ranking from Stereofitter 84
SI.13.2.3 *Q*-factors of lowest AIC ensemble 100

SI.14 Computational details for proline *cis/trans*-isomerization transition barrier **101**

SI.15 Molecular dynamics simulations **103**

SI.15.1 General simulation settings *103*

SI.15.2 RESP charges from different conformers *103*

SI.15.3 Unbiased simulations *105*

SI 15.4 Metadynamics simulations *109*

SI.16 Experimental spectra **111**

SI.16.1 Spectra for CP1 .... *111*

SI.16.2 Spectra for CP2 .... *124*

SI.16.3 Spectra for CP3 .... *138*

SI.16.4 Spectra for CP4 .... *151*

SI.16.5 Spectra for CP5 .... *165*

SI.16.6 Spectra for CP6 .... *177*

SI.16.7 Spectra for CP7 .... *189*

SI.16.8 Spectra for CP8 .... *202*

SI.17 Visualization of principal components of shared PCA **215**

SI.18 Additional Literature for the SI **217**

SI.1: Assignments of the cyclic peptides CP1-CP8

The assignment of the cyclic peptides[1,2] with the experimentally determine *J*-couplings and RDCs are shown below. Additionally, the chemical shifts in the anisotropic environment are shown.

SI.1.1: Assignment of cyclic peptide CP1

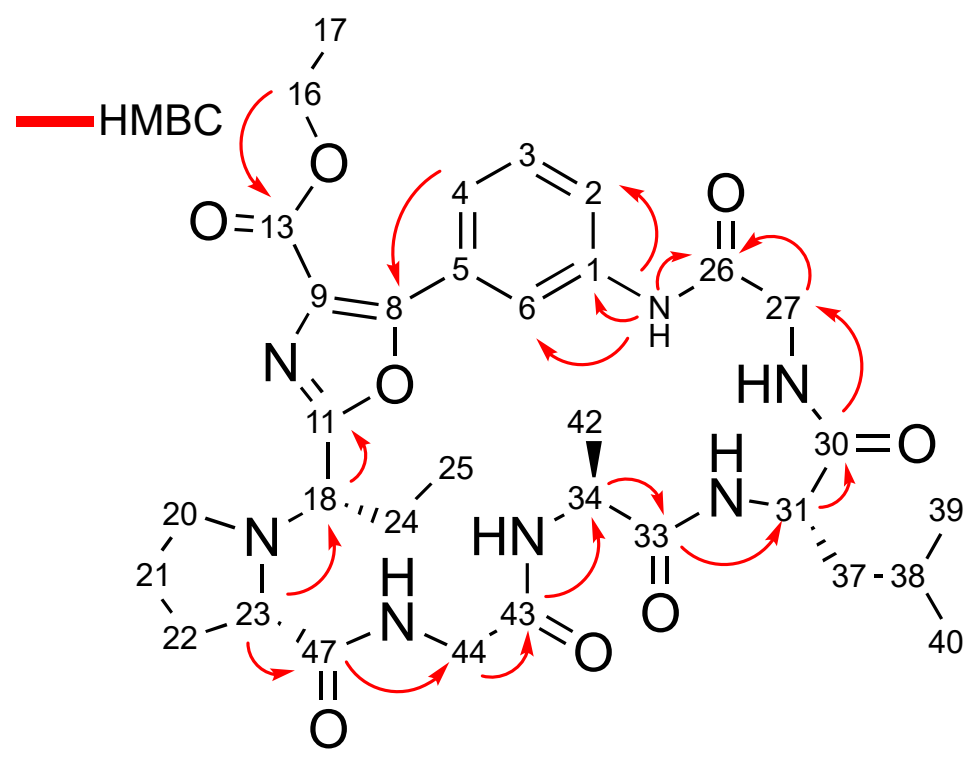

Figure S1. Key HMBC correlations for backbone assignment of cyclic peptide **CP1** in DMSO.

Table S1. Assignment of cyclic peptide **CP1** in DMSO at 300 K. Additionally, the $^3J_{HH}$ coupling constants as well as the RDC measured in DMSO/OPA are shown.

Residue	Atom	Atom-ID carbon/nitrogen	Atom-ID hydrogen	δ_{carbon} in [ppm]	$\delta_{carbon, aniso}$ in [ppm][a]	$\delta_{hydrogen}$ in [ppm]	$\delta_{hydrogen, aniso}$ in [ppm][a]	*J*-coupling of $\delta_{hydrogen}$ in [Hz]	Measured RDC in [Hz]
1Pro	N	19	–			–			
	Cα	23	66	64.0	64.2	3.27	3.45		8.08
	Cβ	22	64,65	29.9	29.7	1.97,1.69	2.00, 1.72		
	Cγ	21	62,63	22.8	22.6	1.68	1.73		
	Cδ	20	60,61	48.2	48.4	2.90,2.58	3.00, 2.71		
	CO	47	–	173.7		–			
2Gly	NH	46	93			8.05	overlap		
	Cα	44	91,92	42.5	42.5	4.13,3.65	4.14, 3.70		13.26, -0.35
	CO	43		169.2					
3Ala	NH	35	78			7.70	overlap	6.9 (d)	-1.00
	Cα	34	77	48.8	49.1	4.32	4.29		-6.15
	Cβ	42	88,89,90	18.0	18.0	1.27	1.27		0.36
	CO	33		173.0					

4Leu	NH	32	76			8.21	8.21	6.5 (d)	-1.55
	Cα	31	75	51.9	51.9	4.18	4.20		2.53
	Cβ	37	79,80	39.9	39.9	1.56	1.55		-6.10
	Cγ	38	81	24.3	24.4	1.57	1.56		-3.60
	Cδ1*	39	82,83,84	22.8	23.0	0.90	0.90	4.7	1.53
	Cδ2*	40	85,86,87	21.6	21.6	0.85	0.85	4.5	-0.33
	CO	30		172.1					
5Gly	NH	28	74			8.13	overlap		4.45
	Cα	27	72,73	43.0	43.0	4.07,3.76	4.07, 3.79		15.44, 5.33
	CO	26		167.8					
6BiA	NH	7	53		–	9.76	9.84		-5.61
	C1	1	–	138.7					
	C2	2	49	121.3	121.5	7.65	7.58	8.0 (d)	-6.86
	C3	3	50	128.8	128.9	7.46	7.46	8.2 (t)	1.25
	C4	4	51	124.3	124.6	7.97	7.94	8.0 (d)	-25.26
	C5	5	–	127.0					
	C6	6	52	118.4	118.7	8.18	8.23		-2.78
	C7	8	–	158.9					
	C8	9	–	126.5					
	C9	11	–	161.7					
	C10	18	59	59.7	59.6	3.76	3.93		-14.33
	C11	24	67,68	25.1	24.8	1.99,1.85	2.02, 1.90		
	C12	25	69,70,71	11.0	10.9	0.82	0.82	7.2	-1.88
	C13	13		161.7					
	C14	16	54,55	61.0	61.0	4.3	4.3		6.90
	C15	17	56,57,58	14.1	14.1	1.28	1.28		-1.22

*can't be unambiguously assigned due to fast chemical exchange. [a] For the chemical shift of carbon and hydrogens bonded to carbon the anisotropic CLIP-HSQC was used, referenced to the solvent peak. The nitrogen bonded hydrogen anisotropic chemical shifts were extracted from the anisotropic 1H spectra referenced to the solvent peak.

SI.1.2: Assignment of cyclic peptide CP2

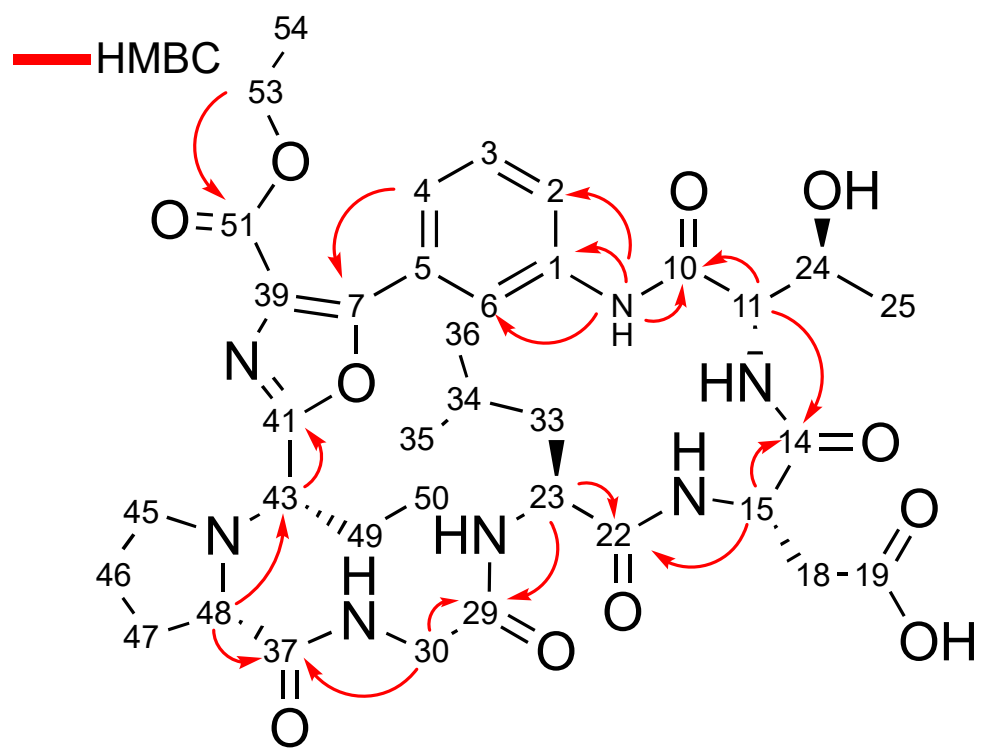

Figure S2. Key HMBC correlations for backbone assignment of cyclic peptide **CP2** in methanol.

Table S2. Assignment of cyclic peptide **CP2** in methanol at 300 K. Additionally, the $^{3}J_{HH}$ coupling constants as well as the RDC measured in methanol/AAKLVFF are shown.

Residue	Atom	Atom-ID carbon/nitrogen	Atom-ID hydrogen	δ_{carbon} in [ppm]	$\delta_{carbon, aniso}$ in [ppm][a]	$\delta_{hydrogen}$ in [ppm]	$\delta_{hydrogen, aniso}$ in [ppm][a]	*J*-coupling of $\delta_{hydrogen}$ in [Hz]	Measured RDC in [Hz]
1Pro	N	44							
	Cα	48	92	65.2	65.9	3.49	3.69		3.87
	Cβ	47	90,91	32.1	31.4	2.22,1.87	2.27, 1.93		-13.91,0.92
	Cγ	46	88,89	24.8	24.3	1.78	1.86		5.42
	Cδ	45	86,87	53.0	52.3	2.89,2.65	3.13, 2.96		
	CO	37	–	178.4		–			
2Gly	NH	31	75			8.47	8.48	8.2, 3.9	-4.45
	Cα	30	73,74	43.5	43.6	4.38,3.79	4.37, 3.85		4.76, 1.69
	CO	29	–	171.7		–			
3Leu	NH	28	72			8.29	8.26	8.7	3.35
	Cα	23	66	54.3	54.2	4.38	4.37		-0.85
	Cβ	33	76,77	41.6	41.5	1.66	1.66		-1.07
	Cγ	34	78	26.0	25.9	1.72	1.70		7.20
	Cδ1*	35	79,80,81	21.8	22.1	0.90	0.89	6.3	-0.19
	Cδ2*	36	82,83,84	22.9	22.8	0.96	0.95	6.4	0.02
	CO	22		175.4					
4Glu	NH	16	62			8.72	8.68	6.9	5.24
	Cα	15	61	53.2	53.0	4.56	4.57		4.29

	Cβ	18	64,65	36.8	–	2.84,2.88	– overlap		
	Cγ	19	–	174.9		–			
	CO	14	–	173.2		–			
5Thr	NH	13	60			7.41	7.38	8.6	6.08
	Cα	11	59	60.5	60.3	4.50	4.52		2.71
	Cβ	24	67	68.8	69.0	4.29	4.28		-2.37
	Cγ	25	68,69,70	20.0	20.0	1.22	1.22	6.2	2.01
	OH	71							
	CO	10		170.7					
6BiA	NH	9	58			9.63	9.81		2.38
	C1	1	–	139.8					
	C2	2	56	123.5	123.4	7.67	7.56	8.0	-1.19
	C3	3	57	128.5	128.5	7.46	7.46	8.0	
	C4	4	8	126.0	126.3	8.29	8.21	8.0	-13.10
	C5	5	–	130.0					
	C6	6	103	120.7	120.6	8.39	8.40		-6.70
	C7	7		156.1					
	C8	39		127.5					
	C9	41		163.8					
	C10	43	85	64.3	63.3	3.77	4.06		-2.69
	C11	49	93,94	26.8	26.3	2.10,1.91	2.20, 1.99		
	C12	50	95,96,97	10.7	10.5	0.82	0.85	7.3	0.24
	C13	51	–	163.2		–			
	C14	53	98,99	62.6	62.6	4.41	4.41	7.1	4.25
	C15	54	100,101,102	14.1	14.1	1.40	1.40	7.2	0.20

*can’t be unambiguously assigned due to fast chemical exchange. [a] For the chemical shift of carbon and hydrogens bonded to carbon the anisotropic CLIP-HSQC was used, referenced to the solvent peak. The nitrogen bonded hydrogen anisotropic chemical shifts were extracted from the anisotropic 1H spectra referenced to the solvent peak.

SI.1.3: Assignment of cyclic peptide CP3

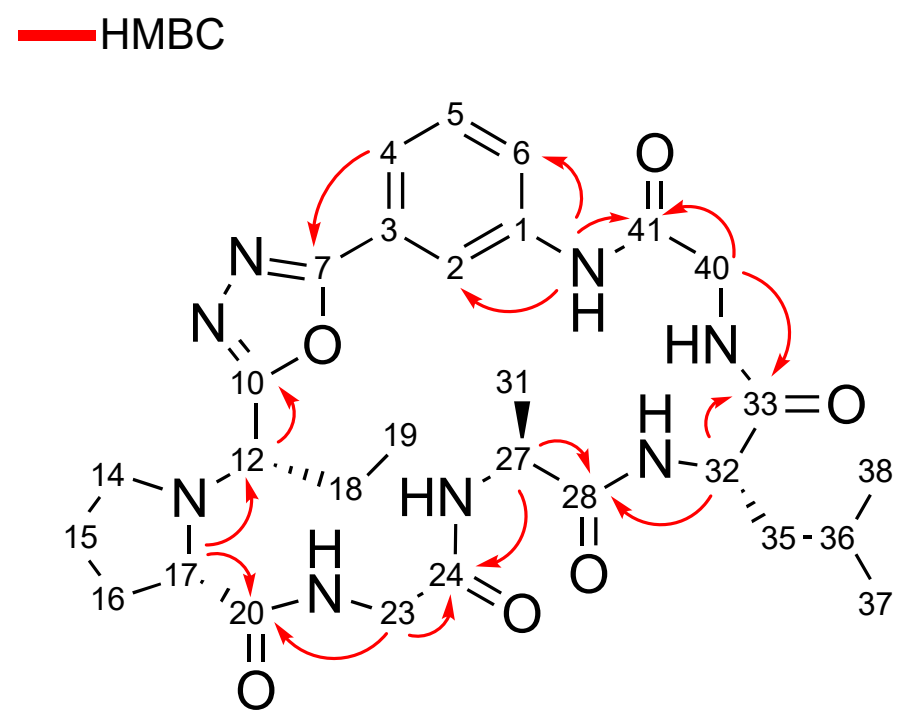

Figure S3. Key HMBC correlations for backbone assignment of cyclic peptide **CP3** in methanol.

Table S3. Assignment of cyclic peptide **CP3** in methanol at 300 K. Additionally, the $^{3}J_{HH}$ coupling constants as well as the RDC measured in methanol/OPA are shown.

Residue	Atom	Atom-ID carbon/nitrogen	Atom-ID hydrogen	δ_{carbon} in [ppm]	$\delta_{carbon, aniso}$ in [ppm][a]	$\delta_{hydrogen}$ in [ppm]	$\delta_{hydrogen, aniso}$ in [ppm][a]	*J*-coupling of $\delta_{hydrogen}$ in [Hz]	Measured RDC in [Hz]
1Pro	N	13	–						
	Cα	17	55	65.5	65.5	3.28	3.32	9.8, 5.6 (dd)	11.10
	Cβ	16	53,54	31.5	31.3	2.13, 1.83	2.12, 1.84		-14.09, 5.75
	Cγ	15	51,52	24.0	23.9	1.82, 1.78	1.80, 1.79		
	Cδ	14	49,50	50.5	50.6	2.99, 2.65	3.00, 2.67		-4.16, 1.30
	CO	20		177.4					
2Gly	NH	21	61		–	8.45	8.46		3.99
	Cα	23	62,63	43.5	43.4	4.30, 3.84	4.30, 3.85	16.5, 7.7, 4.2 (dd)	-6.86, 16.77
	CO	24		171.7					
3Ala	NH	26	64		–	7.94	7.95		5.94
	Cα	27	65	50.6	50.7	4.45	4.45	7.0	16.65
	Cβ	31	67,68,69	18.2	18.1	1.43	1.42	7.2	-0.76
	CO	28		175.3					
4Leu	NH	29	66		–	8.19	8.20	6.5	2.67
	Cα	32	70	53.6	54.5	4.41	4.41		9.34
	Cβ	35	72,73	41.4	41.2	1.66	1.66		

	Cγ	36	74	25.8	25.7	1.66	1.66		-5.16
	Cδ1*	37	75,76,77	22.0	21.9	0.95	0.95	5.6	0.09
	Cδ2*	38	78,79,80	23.2	23.1	0.98	0.97	5.6	-1.05
	CO	33		175.1					
5Gly	NH	34	71		–	8.37	8.36	4.5	5.85
	Cα	40	81,82	44.2	44.1	4.31, 3.77	4.30, 3.77	17.1, 7.1, 4.3	14.61, -11.32
	CO	41		170.0					
6BiA	NH	42	83		–	9.83	9.85		4.41
	C1	1		140.4					
	C2	2	44	124.8	124.7	7.80	7.77	8.1	-18.21
	C3	3	45	130.8	130.7	7.53	7.53	8.0	7.13
	C4	4	46	123.5	123.5	7.93	7.93	8.2	-15.49
	C5	5		125.0					
	C6	6	47	120.0	119.9	8.41	8.42		6.85
	C7	7	–	166.2					
	C8	10	–	167.3					
	C9	12	48	60.8	60.7	3.97	4.00	10.3, 5.2	-15.51
	C10	18	56,57	31.5	31.2	2.21, 1.93	2.21, 1.93		
	C11	19	58,59,60	11.0	10.9	0.87	0.87	7.4	

*can't be unambiguously assigned due to fast chemical exchange. [a] For the chemical shift of carbon and hydrogens bonded to carbon the anisotropic CLIP-HSQC was used, referenced to the solvent peak. The nitrogen bonded hydrogen anisotropic chemical shifts were extracted from the anisotropic 1H spectra referenced to the solvent peak.

SI.1.4: Assignment of cyclic peptide CP4

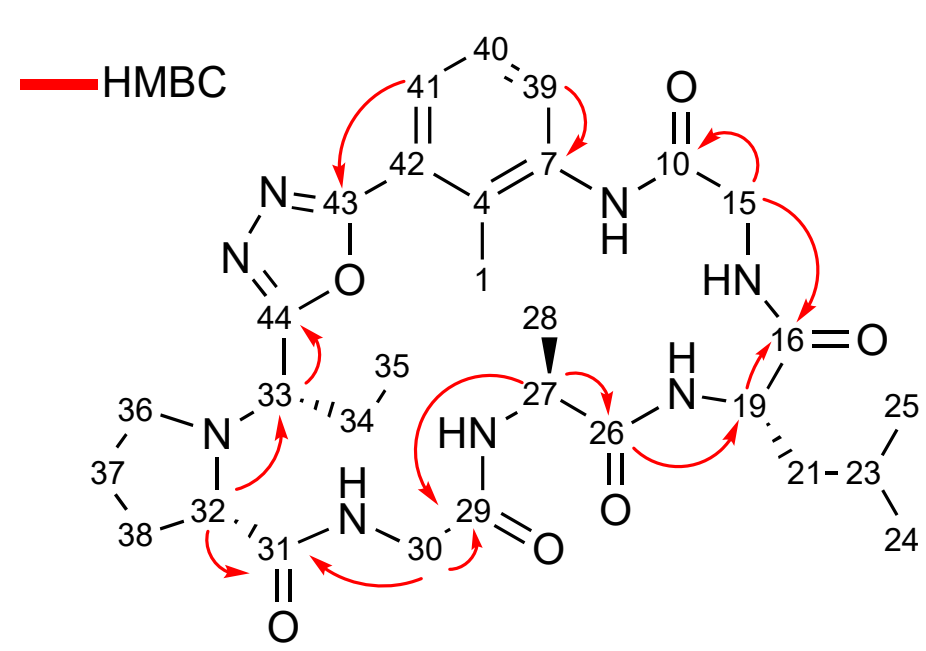

Figure S4. Key HMBC correlations for backbone assignment of cyclic peptide **CP4** in methanol.

Table S4. Assignment of cyclic peptide **CP4** in methanol at 300 K. Additionally, the $^{3}J_{HH}$ coupling constants as well as the RDC measured in methanol/AAKLVFF are shown.

Residue	Atom	Atom-ID carbon/nitrogen	Atom-ID hydrogen	δ_{carbon} in [ppm]	$\delta_{carbon, aniso}$ in [ppm][a]	$\delta_{hydrogen}$ in [ppm]	$\delta_{hydrogen, aniso}$ in [ppm][a]	*J*-coupling of $\delta_{hydrogen}$ in [Hz]	Measured RDC in [Hz]
1Pro	N	17	–						
	Cα	32	47	64.5	64.6	3.54	3.62	10.1, 3.3 (dd)	-0.72
	Cβ	38	82,83	31.7	31.9	2.22, 1.91	2.24, 1.91		-9.05, 4.74
	Cγ	37	80,81	24.4	24.1	1.82, 1.75	1.81		
	Cδ	36	78,79	53.8	53.6	2.88, 2.62	2.98, 2.72		-1.01, -0.75
	CO	31	–	177.7					
2Gly	NH	14	72			8.39	8.41	6.9, 3.3	2.33
	Cα	30	70,71	42.6	42.7	4.24, 3.82	4.21, 3.83	17.0	3.19, 2.24
	CO	29	–	170.7					
3Ala	NH	11	69			8.17	8.15	6.4 (d)	3.21
	Cα	27	46	50.6	50.6	4.37	4.36	7.3 (q)	8.71
	Cβ	28	66,67,68	18.2	18.2	1.38	1.38	7.3 (d)	-0.68
	CO	26	–	175.2					
4Leu	NH	8	65			7.84	7.80	7.8 (d)	-0.07
	Cα	19	45	52.8	52.9	4.51	4.49	8.9, 5.5 (dd)	-1.27
	Cβ	21	57,58	42.0	42.0	1.66	1.66		
	Cγ	23	58	25.9	25.8	1.64	1.62		
	Cδ1*	24	59,60,61	22.0	22.0	0.97	0.96	5.9 (d)	
	Cδ2*	25	62,63,64	23.8	23.8	0.97	0.97	5.9 (d)	
	CO	16	–	175.6					
5Gly	NH	5	55			8.93	8.93		
	Cα	13	53,54	44.6	44.5	4.25, 3.72	4.24, 3.74	16.5	4.13, -10.30
	CO	10	–	171.1					
6BiA	NH	2	52			9.4	9.44		
	C1	7	–	138.3					

C2	39	84	128.8	128.9	7.92	7.92	7.8	-17.04
C3	40	85	127.3	127.3	7.41	7.41	7.85	5.05
C4	41	86	130.8	131.1	7.63	7.59	7.9	-7.05
C5	42	–	125.0					
C6	4	–	135.5					
C7 (Me-thyl)	1	49,50,51	16.2	16.1	2.42	2.41		-1.04
C8	43	–	167.8					
C9	44	–	168.5					
C10	33	48	62.5	62.0	4.00	4.10		2.97
C11	34	73,74	26.1	25.6	2.24, 1.89	2.24, 1.89		
C12	35	75,76,77	10.5	10.4	0.80	0.81	7.3	

* can't be unambiguously assigned due to fast chemical exchange. [a] For the chemical shift of carbon and hydrogens bonded to carbon the anisotropic CLIP-HSQC was used, referenced to the solvent peak. The nitrogen bonded hydrogen anisotropic chemical shifts were extracted from the anisotropic 1H spectra referenced to the solvent peak.

SI.1.5: Assignment of cyclic peptide CP5

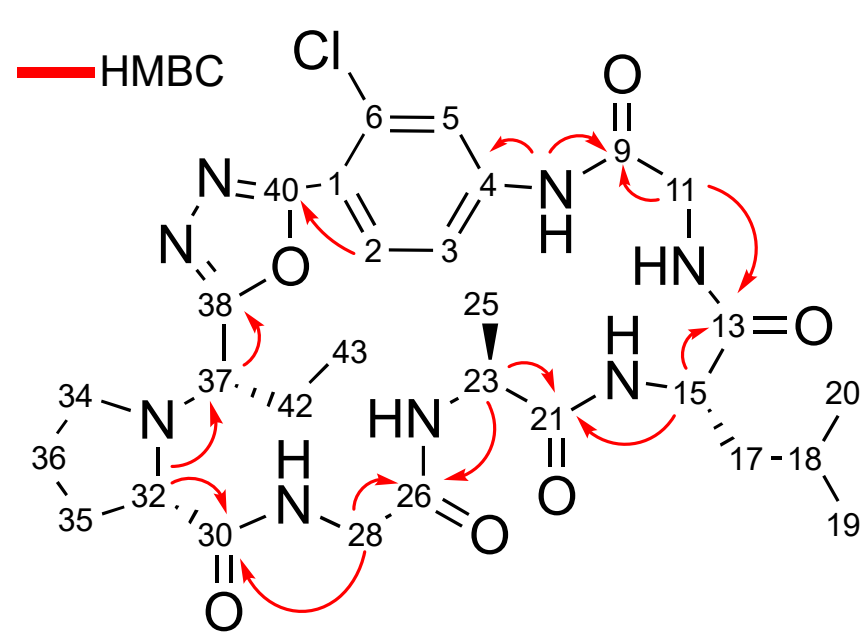

Figure S5. Key HMBC correlations for backbone assignment of cyclic peptide **CP5** in DMSO.

Table S5. Assignment of cyclic peptide **CP5** in DMSO at 300 K. Additionally, the $^3J_{HH}$ coupling constants as well as the RDC measured in DMSO/OPA are shown.

Residue	Atom	Atom-ID carbon/nitrogen	Atom-ID hydrogen	δ_{carbon} in [ppm]	$\delta_{carbon, aniso}$ in [ppm][a]	$\delta_{hydrogen}$ in [ppm]	$\delta_{hydrogen, aniso}$ in [ppm][a]	*J*-coupling of $\delta_{hydrogen}$ in [Hz]	Measured RDC in [Hz]
1Pro	N	33	–			–			
	Cα	32	71	58.5	57.4	3.77	4.12	8.21, 3.6 (dd)	-0.38
	Cβ	35	74,75	31.0	31.0	1.98,1.59	1.69, 1.50		-4.59
	Cγ	36	76,77	22.1	22.2	1.69	1.73		-0.91
	Cδ	34	72,73	50.1	–	3.11,2.75	–	7.8, 6.9	

	CO	30	–	174.1		–			
2Gly	NH	29	70			7.85	– overlap		-6.14
	Cα	28	68,69	42.3	42.3	3.59,3.50	3.60, 3.48	17.7, 17.5, 4.0 (dd)	-1.05, 9.30
	CO	26	–	167.4		–			
3Ala	NH	24	64		–	8.10	8.12	8.2 (d)	5.17
	Cα	23	63	47.1	47.0	4.51	4.51	7.5 (penta)	8.99
	Cβ	25	65,66,67	17.9	17.8	1.24	1.24	7.5 (d)	2.38
	CO	21	–	172.8		–			
4Leu	NH	16	53		–	8.20	8.19		-8.08
	Cα	15	52	53.8	53.8	3.99	4.00	5.2 (q)	-5.65
	Cβ	17	54,55	38.5	38.5	1.65,1.50	1.65, 1.50		0.47
	Cγ	18	56	23.9	23.9	1.60	1.60		1.80
	Cδ1*	19	57,58,59	21.9	21.9	0.89	0.89	6.3	2.71
	Cδ2*	20	60,61,62	22.4	22.4	0.94	0.94	6.3	0.67
	CO	13	–	172.3		–			
5Gly	NH	12	51		–	8.92	8.91	5.68	9.35
	Cα	11	49,50	43.3	43.2	4.12,3.62	4.14, 3.62	16.9, 7.5, 17.1, 4.0	2.32, -11.61
	CO	9	–	169.0		–			
6BiA	NH	8	48		–	9.57	9.61		8.85
	C1	4	–	141.55		–			
	C2	5	47	119.6	119.6	8.40	8.41		8.00
	C3	6	–	132.2		–			
	C4	1	–	116.8		–			
	C5	2	45	130.6	130.4	7.81	7.81	8.7	8.60
	C6	3	46	118.7	118.6	7.62	7.60	7.6	16.54
	C7	40	–	161.1		–			
	C8	38	–	164.4		–			
	C9	37	78	55.5	57.4	4.20	4.17	9.3, 6.7	1.92
	C10	42	79,80	21.1	20.4	1.95	1.98		-0.35
	C11	43	81,82,83	10.6	10.3	0.85	0.85	7.3	6.64

* Can't be unambiguously assigned due to fast chemical exchange. [a] For the chemical shift of carbon and hydrogens bonded to carbon the anisotropic CLIP-HSQC was used, referenced to the solvent peak. The nitrogen bonded hydrogen anisotropic chemical shifts were extracted from the anisotropic 1H spectra referenced to the solvent peak.

SI.1.6: Assignment of cyclic peptide CP6

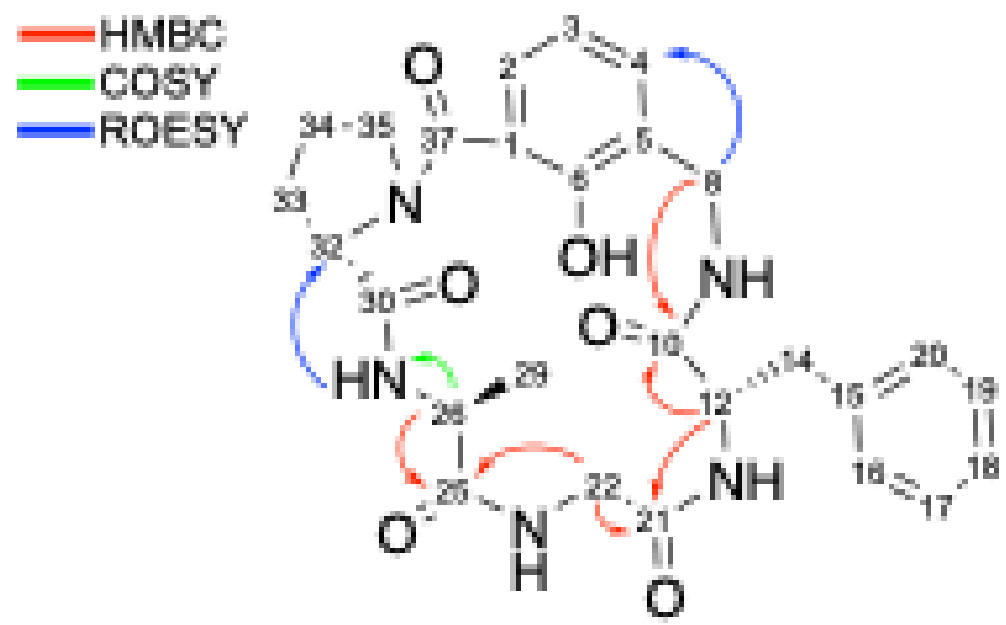

Figure S6. Key HMBC correlations for backbone assignment of cyclic peptide **CP6** in DMSO.

Table S6. Assignment of the major conformer of cyclic peptide **CP6** in DMSO at 300 K. Additionally, the $^{3}J_{HH}$ coupling constants as well as the RDC measured in DMSO/OPA are shown.

Resi-due	Atom	Atom-ID carbon/ni-trogen	Atom-ID hydrogen	δ_{carbon} in [ppm]	$\delta_{carbon, aniso}$ in [ppm][a]	$\delta_{hydrogen}$ in [ppm]	$\delta_{hydrogen, aniso}$ in [ppm][a]	*J*-coupling of $\delta_{hydrogen}$ in [Hz]	Measured RDC in [Hz]
1Pro	N	36	–	–	–	–	–	–	–
	Cα	32	63	60.8	60.8	4.36	4.37		18.07
	Cβ	33	64,65	29.1	29.1	2.26, 1.84	2.26, 1.84		-5.85, -5.23
	Cγ	34	66,67	24.4	24.4	1.87	1.87		-0.60
	Cδ	35	68,69	48.0	48.0	3.72, 3.39	3.73, 3.39		6.23, -12.30
	CO	30	–			–		–	–
2Ala	NH	28	59	–	–	7.42	7.43		5.29
	Cα	26	58	47.8	47.8	4.25	4.26		10.03
	Cβ	29	60,61,62	18.1	18.1	1.27	1.27		-0.36
	CO	25	–	171.84		–		–	–
3Gly	NH	23	57	–	–	8.06	8.05		2.18
	Cα	22	55,56	42.33	42.35	3.80, 3.25	3.79, 3.25		5.0, 7.3
	CO	21	–	169.1		–		–	–
4Phe	NH	13	47	–	–	8.09	8.09		-0.40
	Cα	12	46	54.2	54.3	4.34	4.35		4.49
	Cβ	14	48,49	36.1	36.1	2.95, 2.83	2.95, 2.83		0.90, 3.91
	Cγ	15	–	137.7		–		–	–

	Cδ1	16	50	128.8	128.7	7.24	7.24		1.00
	Cδ2	20	54	128.8	128.7	7.24	7.24		1.00
	Cε1	17	51	127.8	127.9	7.27	7.27		0.45
	Cε2	19	53	127.8	127.9	7.27	7.27		0.45
	Cζ	18	52	127.8	127.9	7.27	7.27		0.45
	CO	10	–	172.8		–		–	–
5Aro	NH	9	45	–	–	8.70	8.70		-2.53
	C1	8	43,44	39.5		4.73, 3.87			0.86, 1.39
	C2	5	–			–		–	–
	C3	6	–			–		–	–
	C4	1	–			–		–	–
	C5	2	39	126.0	126.0	7.20	7.20		(-8.79)
	C6	3	40	118.8	6.86	6.86	118.8		-5.71
	C7	4	41	132.4	132.4	7.28	7.28		-2.35
	OH	7	42	–	–	9.78	9.80		
	CO	37	–			–		–	–

[a] For the chemical shift of carbon and hydrogens bonded to carbon the anisotropic CLIP-HSQC was used, referenced to the solvent peak. The nitrogen bonded hydrogen anisotropic chemical shifts were extracted from the anisotropic 1H spectra referenced to the solvent peak.

SI.1.7: Assignment of cyclic peptide CP7

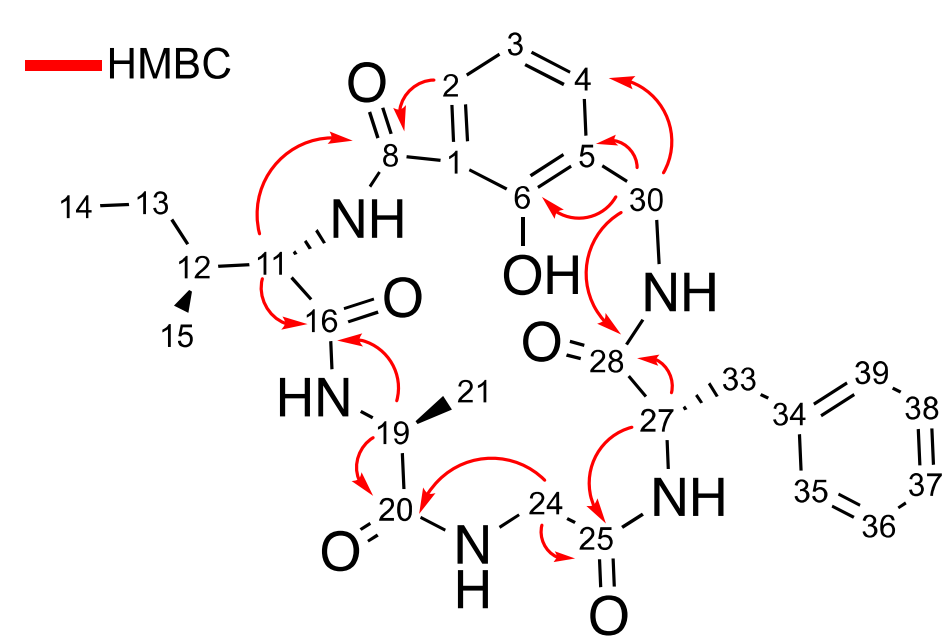

Figure S7. Key HMBC correlations for backbone assignment of the major peak set of cyclic peptide **CP7** in DMSO.

Table S7. Assignment of the major conformer of the major peak set of cyclic peptide **CP7** in DMSO at 300 K. Additionally, the $^3J_{HH}$ coupling constants as well as the RDC measured in DMSO/OPA are shown.

Residue	Atom	Atom-ID carbon/nitrogen	Atom-ID hydrogen	δ_{carbon} in [ppm]	$\delta_{carbon, aniso}$ in [ppm][a]	$\delta_{hydrogen}$ in [ppm]	$\delta_{hydrogen, aniso}$ in [ppm][a]	*J*-coupling of $\delta_{hydrogen}$ in [Hz]	Measured RDC in [Hz]

1Ile	NH	9	44	–	–	8.73	8.75	3.8		6.63
	Cα	11	45	60.9	60.9	3.93	3.93			17.45
	Cβ1	12	46	34.9	34.8	1.99	2.00			11.81
	Cγ1	13	47,48	26.2	26.2	1.59, 1.25	1.59, 1.25			-0.61, –
	Cγ2	15	52,53,54	15.9	15.8	0.90	0.91			-1.70
	Cδ	14	49,50,51	11.2	11.1	0.89	0.89			2.98
	CO	16		170.7						
2Ala	NH	17	55		–	7.69	– (overlap)	7.0		8.81
	Cα	19	56	48.1	48.0	4.30	4.30			11.81
	Cβ	21	57,58,59	19.0	18.9	1.15	1.15			-4.39
	CO	20		172.1						
3Gly	NH	22	60		–	7.73	7.74			6.13
	Cα	24	61,62	41.9	41.8	3.81, 3.24	3.80, 3.24	17.0, 4.3	7.1,	7.58,-2.96
	CO	25		168.9						
4Phe	NH	26	63		–	8.29	8.30	8.5		5.14
	Cα	27	64	54.6	54.5	4.31	4.31			5.19
	Cβ	33	68,69	36.1	36.0	3.02, 2.73	3.02, 2.73			11.27, 8.36
	Cγ	34	–	138.6						
	Cδ1	35	70	128.6	128.5	7.25	7.25			5.32
	Cδ2	39	74	128.6	128.5	7.25	7.25			5.32
	Cε1	36	71	129.5	129.4	7.20	7.20			6.10
	Cε2	38	73	129.5	129.4	7.20	7.20			6.10
	Cζ	37	72	126.7	126.6	7.18	7.17			-7.12
	CO	28		171.1						
5Aro	NH	29	65		–	7.73	7.73			0.62
	C1	30	66,67	39.5	39.5	4.38, 4.05	4.38, 4.06	14.6, 3.5	8.5,	4.49, 19.22
	C2	5		126.9						
	C3	6		160.0						
	C4	1		115.1						
	C5	2	40	127.8	127.7	7.80	7.80			-18.06

	C6	3	41	118.1	118.0	6.83	6.84		-0.07
	C7	4	42	134.8	134.8	7.37	7.38		1.99
	OH	7	43		–	12.33	12.34		
	CO	8	–	171.1					

[a] For the chemical shift of carbon and hydrogens bonded to carbon the anisotropic CLIP-HSQC was used, referenced to the solvent peak. The nitrogen bonded hydrogen anisotropic chemical shifts were extracted from the anisotropic 1H spectra referenced to the solvent peak.

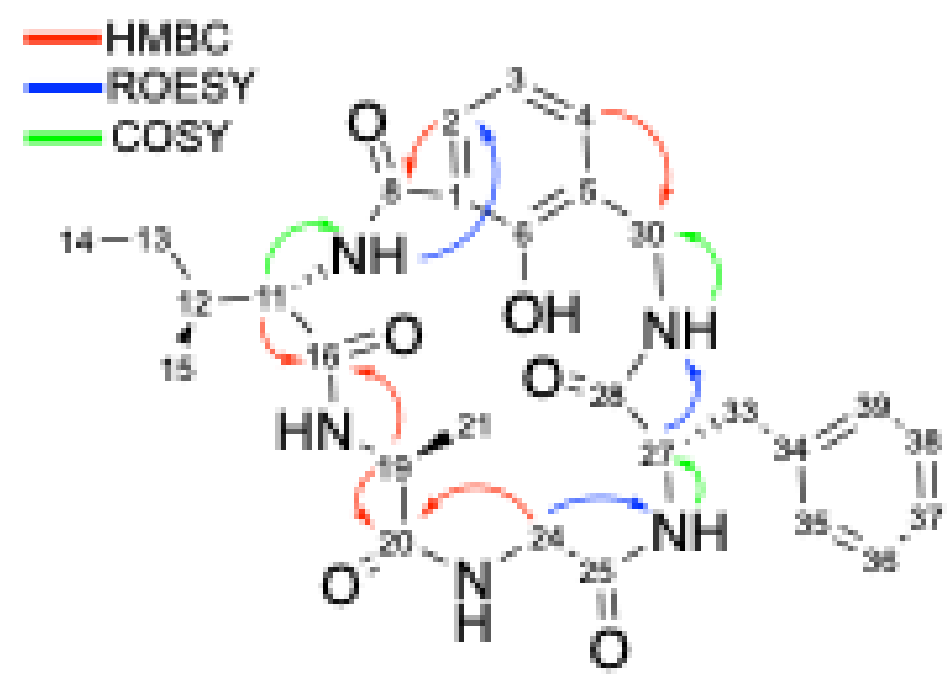

Figure S8. Key HMBC correlations for backbone assignment of the minor peak set of cyclic peptide **CP7** in DMSO.

Table S8. Assignment of the minor peak set of cyclic peptide **CP7** in DMSO at 300 K. Additionally, the $^3J_{HH}$ coupling constants as well as the RDC measured in DMSO/OPA are shown.

Residue	Atom	Atom-ID carbon/nitrogen	Atom-ID hydrogen	δ_{carbon} in [ppm]	$\delta_{carbon, aniso}$ in [ppm][a]	$\delta_{hydrogen}$ in [ppm]	$\delta_{hydrogen, aniso}$ in [ppm][a]	*J*-coupling of $\delta_{hydrogen}$ in [Hz]	Measured RDC in [Hz]
1Ile	NH	9	44	–		9.10	9.10		-1.2
	Cα	11	45	58.6	58.6	4.23	4.23		-1.08
	Cβ1	12	46	34.1	34.1	2.04	2.06		8.53
	Cγ1	13	47,48	25.1	25.2	1.57,1.17	–		
	Cγ2	15	52,53,54	15.7	15.7	0.87	0.85		
	Cδ	14	49,50,51	10.9	11.0	0.84	0.84		
	CO	16		171.6					
2Ala	NH	17	55			8.63	8.67	6.8	0.26
	Cα	19	56	49.6	49.5	4.15	4.15		13.00
	Cβ	21	57,58,59	17.6	17.6	1.24	1.23		
	CO	20		172.5					
3Gly	NH	22	60			6.94	6.95		2.96

	Cα	24	61,62	42.0	41.9	3.67, 3.41	3.68, 3.40	17.2, 4.4	6.1,	17.09, -8.65
	CO	25		168.3						
4Phe	NH	26	63			8.17	8.17	7.8		1.12
	Cα	27	64	55.4	55.4	4.20	4.20			-4.38
	Cβ	33	68,69	35.9	36.0	2.98, 2.80	–			
	Cγ	34	–	138.6						
	Cδ1	35	70	128.6	128.5	7.25	–			
	Cδ2	39	74	128.6	128.5	7.25	–			
	Cε1	36	71	129.5	129.4	7.20	–			
	Cε2	38	73	129.5	129.4	7.20	–			
	Cζ	37	72	126.7	126.6	7.18	–			
	CO	28		171.6						
5Aro	NH	29	65			8.00	7.99			2.59
	C1	30	66,67	40.5	40.05	4.36, 4.12	–			
	C2	5		126.9						
	C3	6		159.0						
	C4	1		115.1						
	C5	2	40	128.1	128.1	7.92	7.90			-13.24
	C6	3	41	118.1	118.0	6.81	6.78			
	C7	4	42	134.8	134.8	7.37	7.33			
	OH	7	43			12.47	12.47			
	CO	8	–	169.9						

[a] For the chemical shift of carbon and hydrogens bonded to carbon the anisotropic CLIP-HSQC was used, referenced to the solvent peak. The nitrogen bonded hydrogen anisotropic chemical shifts were extracted from the anisotropic 1H spectra referenced to the solvent peak.

SI.1.8: Assignment of cyclic peptide CP8

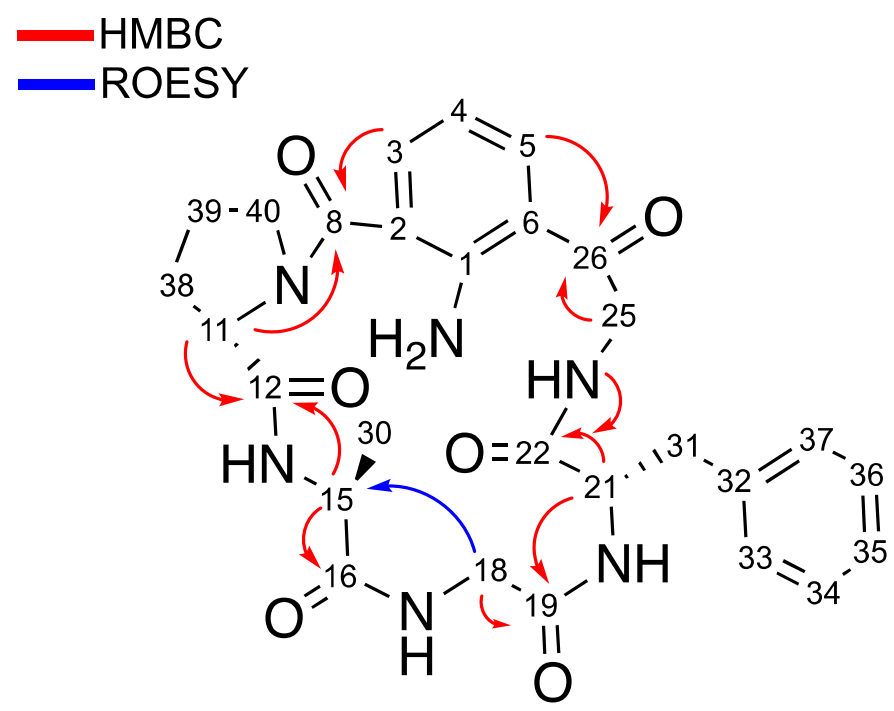

Figure S9. Key HMBC and ROESY correlations for backbone assignment of cyclic peptide **CP8** in DMSO.

Table S9. Assignment of cyclic peptide **CP8** in DMSO at 300 K. Additionally, the $^{3}J_{HH}$ coupling constants as well as the RDC measured in DMSO/OPA are shown.

Residue	Atom	Atom-ID carbon/nitrogen	Atom-ID hydrogen	δ_{carbon} in [ppm]	$\delta_{carbon, aniso}$ in [ppm][a]	$\delta_{hydrogen}$ in [ppm]	$\delta_{hydrogen, aniso}$ in [ppm][a]	*J*-coupling of $\delta_{hydrogen}$ in [Hz]	Measured RDC in [Hz]
1Pro	N	9	—						
	Cα	11	46	60.5	60.6	4.02	4.02		2.74
	Cβ	38	67,68	30.6	30.6	2.14,1.95	2.15, 1.96		3.34, 11.49
	Cγ	39	69,70	22.2	22.3	1.89, 1.65	1.89, 1.66		-13.57, 20.90
	Cδ	40	71,72	45.8	45.8	3.67,3.55	3.67, 3.55		3.95, -0.80
	CO	12	—	171.6		—			
2Ala	NH	13	47	—		8.33	8.33	7.5	4.31
	Cα	15	48	47.3	47.5	4.20	4.21		9.67
	Cβ	30	57,58,59	14.5	14.7	1.20	1.20		0.26
	CO	16	—	170.6		—			
3Gly	NH	17	49	—		6.69	6.69		-2.72
	Cα	18	50,51	41.4	41.5	3.65,3.43	3.65, 3.42		3.02, 1.43
	CO	19	—	166.7		—			
4Phe	NH	20	52	—		8.55	8.55	6.9	-6.40
	Cα	21	53	53.0	53.1	4.43	4.43		-0.33
	Cβ	31	60,61	35.6	35.7	3.01,2.82	3.01, 2.81		4.62, 8.62
	Cγ	32	—	137.5		—			
	Cδ1	33	62	128.6	128.7	7.19	7.18		3.67
	Cδ2	37	66	128.6	128.7	7.19	7.18		3.67

	Cε1	34	63	127.8	128.0	7.26	7.25	3.41
	Cε2	36	65	127.8	128.0	7.26	7.25	3.41
	Cζ	35	64	126.0	126.2	7.18	7.18	3.84
	CO	22	—	169.1		—		
5Gly	NH	23	54	—		8.93	8.92	10.02
	Cα	25	55,56	47.3	47.5	5.25, 3.21	5.25, 3.21	4.76, -2.43
	CO	26		199.9				
6Aro	C1	6		114.8				
	C2	1		145.9				
	C3	2		125.5				
	C4	3	41	133.0	133.2	7.26	7.26	2.62
	C5	4	42	113.3	113.4	6.37	6.37	-10.26
	C6	5	43	132.1	132.2	7.39	7.39	-12.10
	NH2	7	44,45			6.94	6.94	
	CO	8		167.8				

[a] For the chemical shift of carbon and hydrogens bonded to carbon the anisotropic CLIP-HSQC was used, referenced to the solvent peak. The nitrogen bonded hydrogen anisotropic chemical shifts were extracted from the anisotropic 1H spectra referenced to the solvent peak.

SI.2 Correlation between experimental and back-calculated NMR data

In the following Figures **S10–S20** the correlation between the experimental NMR data and the determined ensembles for **CP1–8** using *Stereofitter* are shown below.

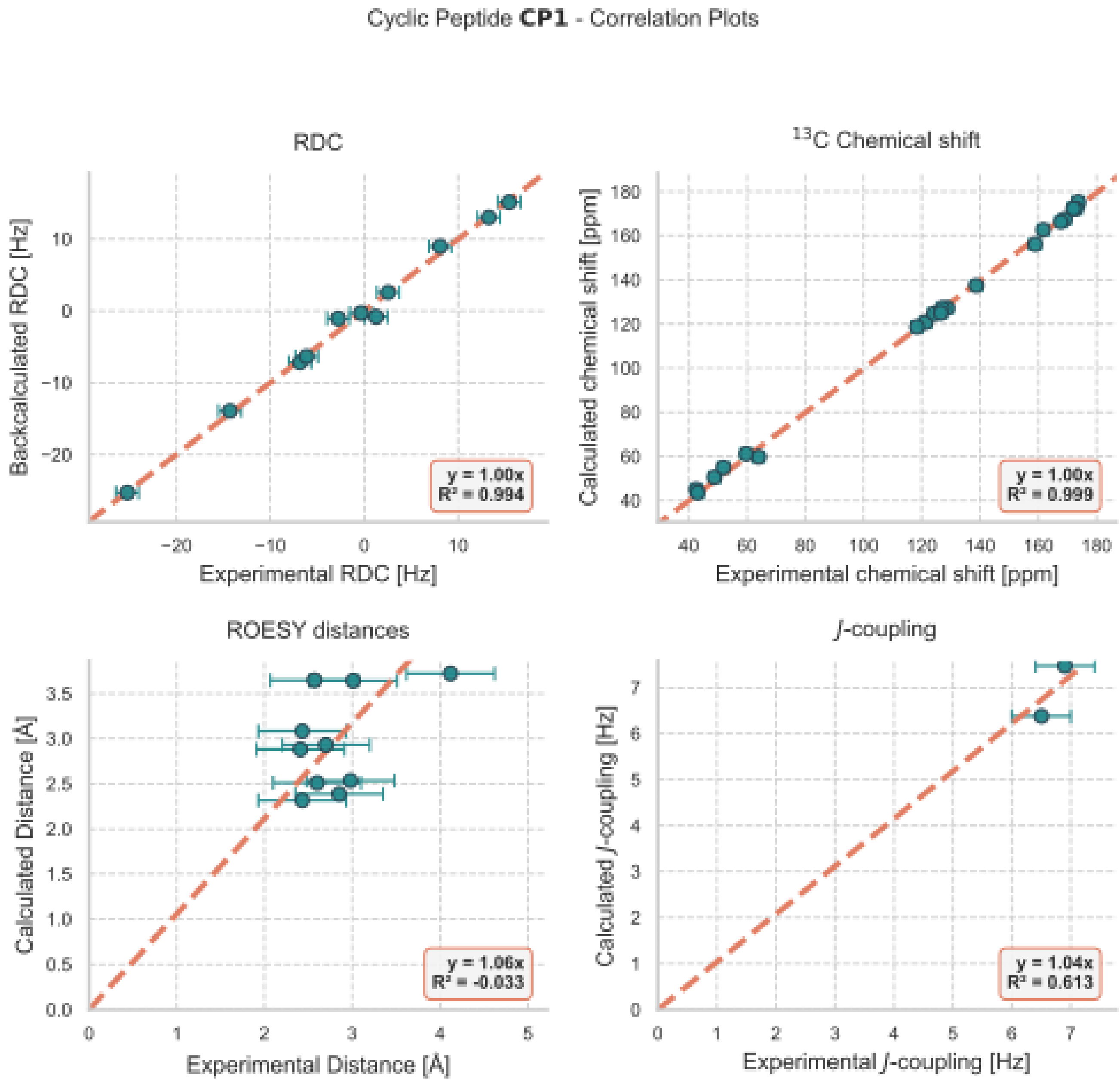

Figure S10. Correlation between experimental NMR data and back-calculated NMR from the conformational ensemble for **CP1** determined using Stereofitter. For each plot a linear regression is shown together with its R^2 values.

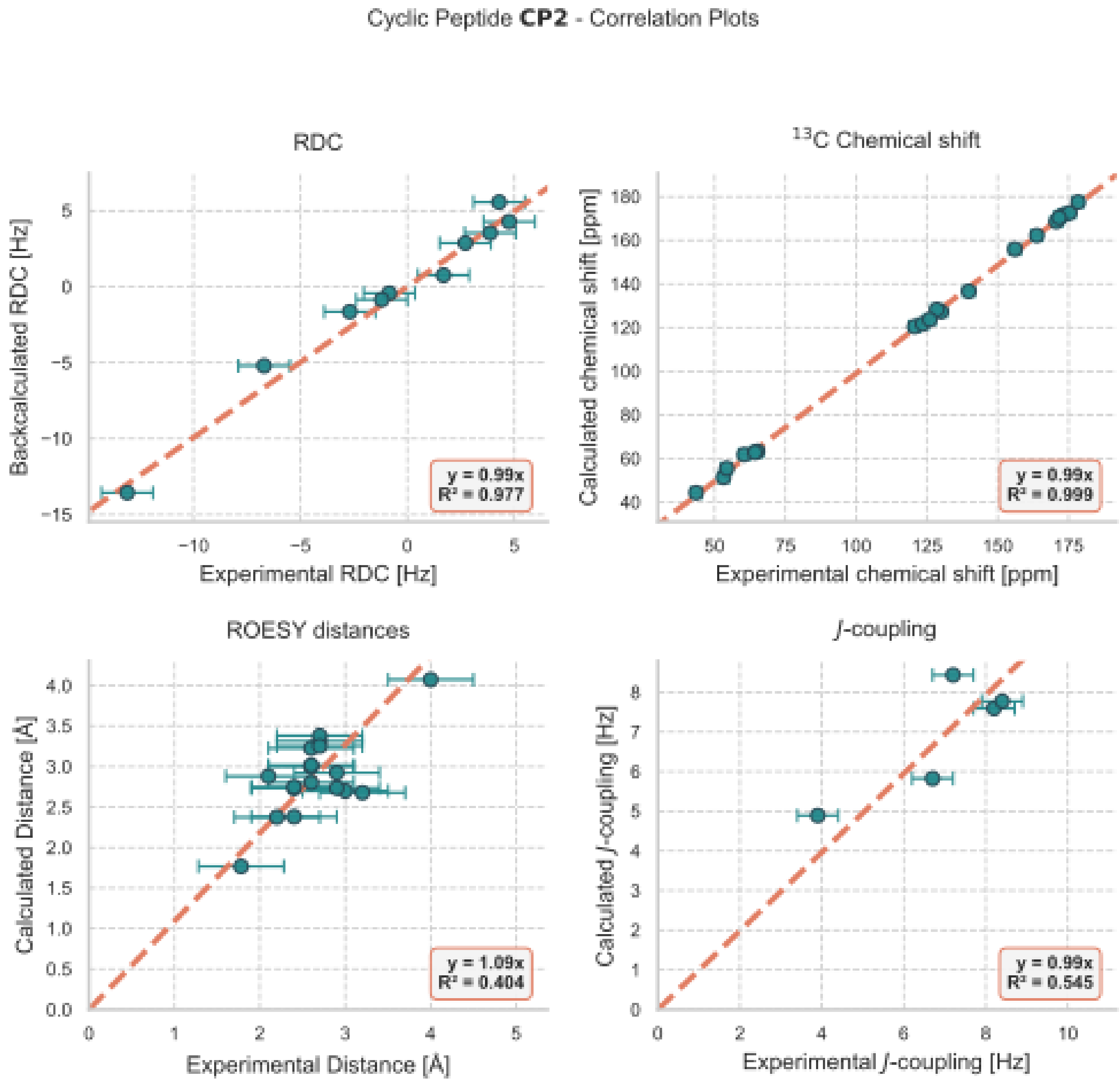

Figure S11. Correlation between experimental NMR data and back-calculated NMR from the conformational ensemble for **CP2** determined using Stereofitter. For each plot a linear regression is shown together with its R^2 values.

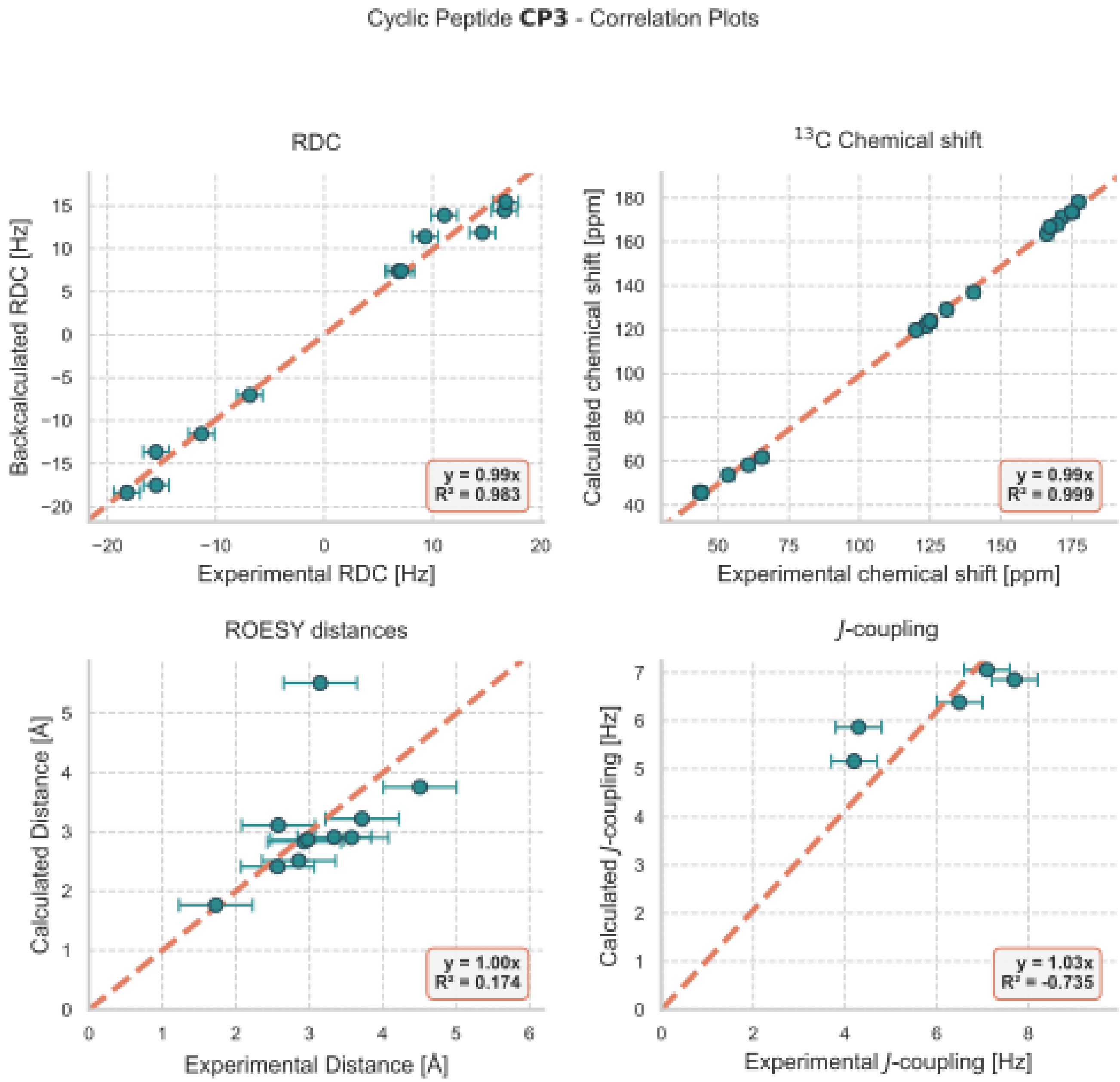

Figure S12. Correlation between experimental NMR data and back-calculated NMR from the conformational ensemble for **CP3** determined using Stereofitter. For each plot a linear regression is shown together with its R^2 values.

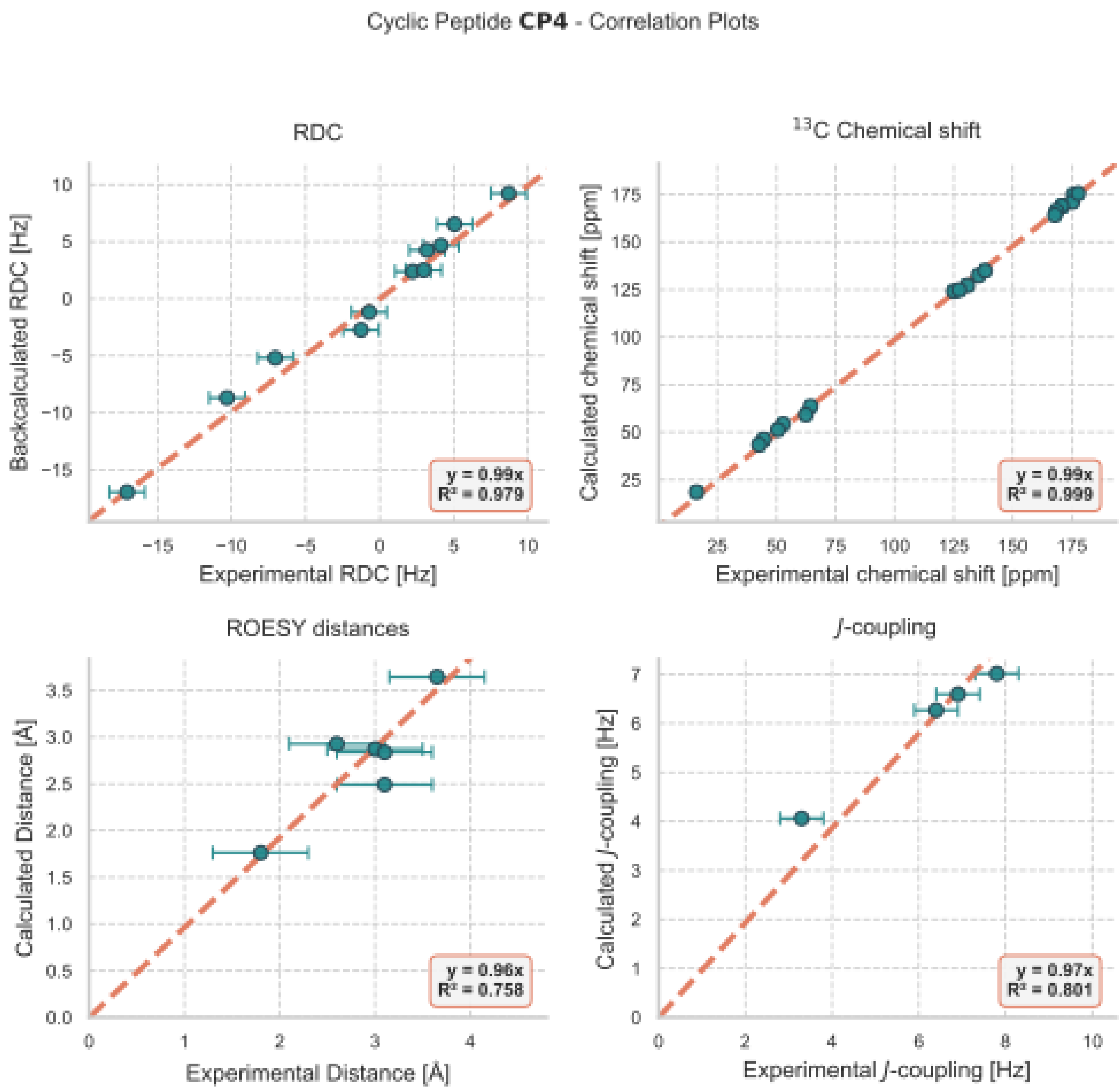

Figure S13. Correlation between experimental NMR data and back-calculated NMR from the conformational ensemble for **CP4** determined using Stereofitter. For each plot a linear regression is shown together with its R^2 values.

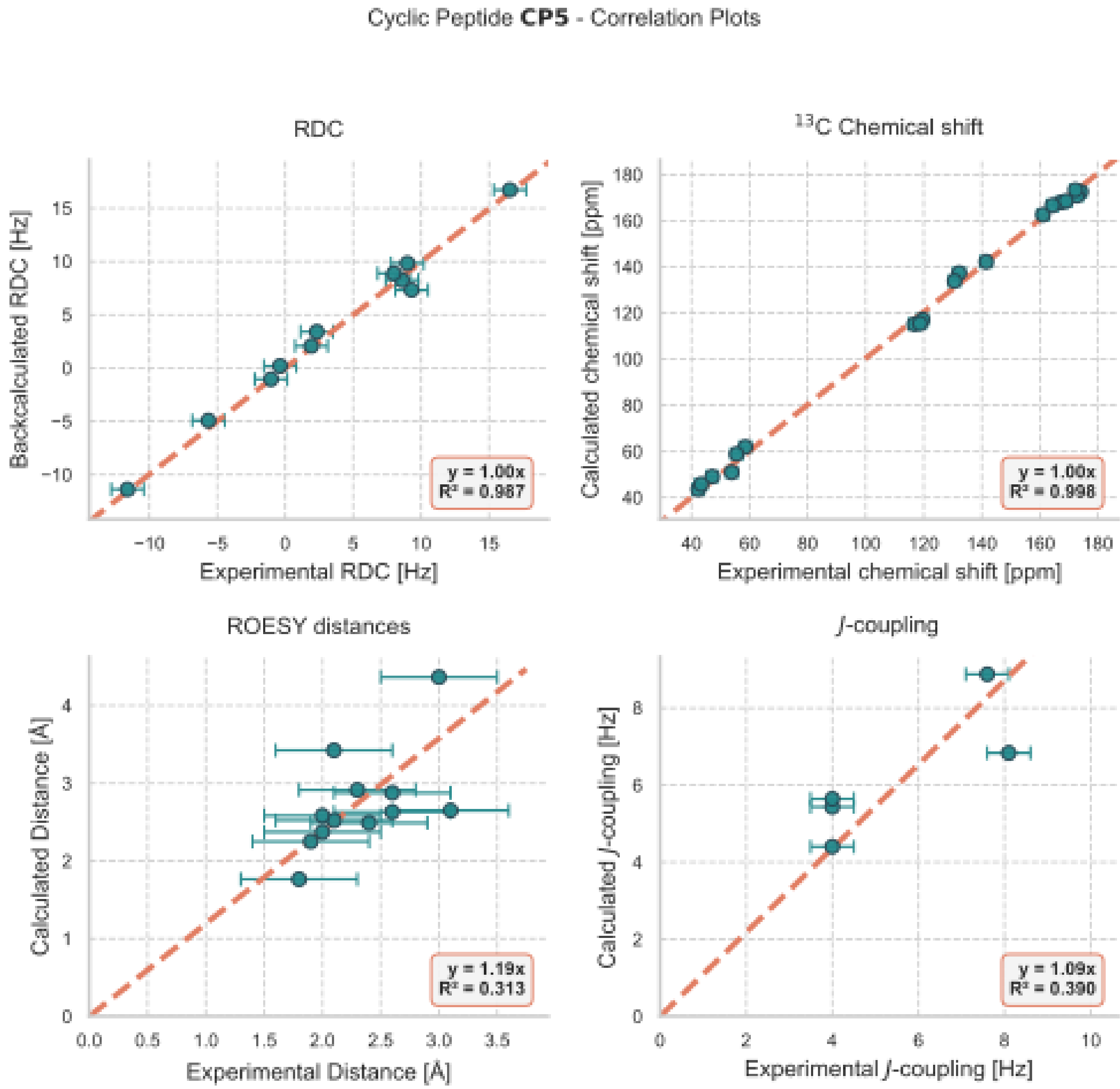

Figure S14. Correlation between experimental NMR data and back-calculated NMR from the conformational ensemble for **CP5** determined using Stereofitter. For each plot a linear regression is shown together with its R^2 values.

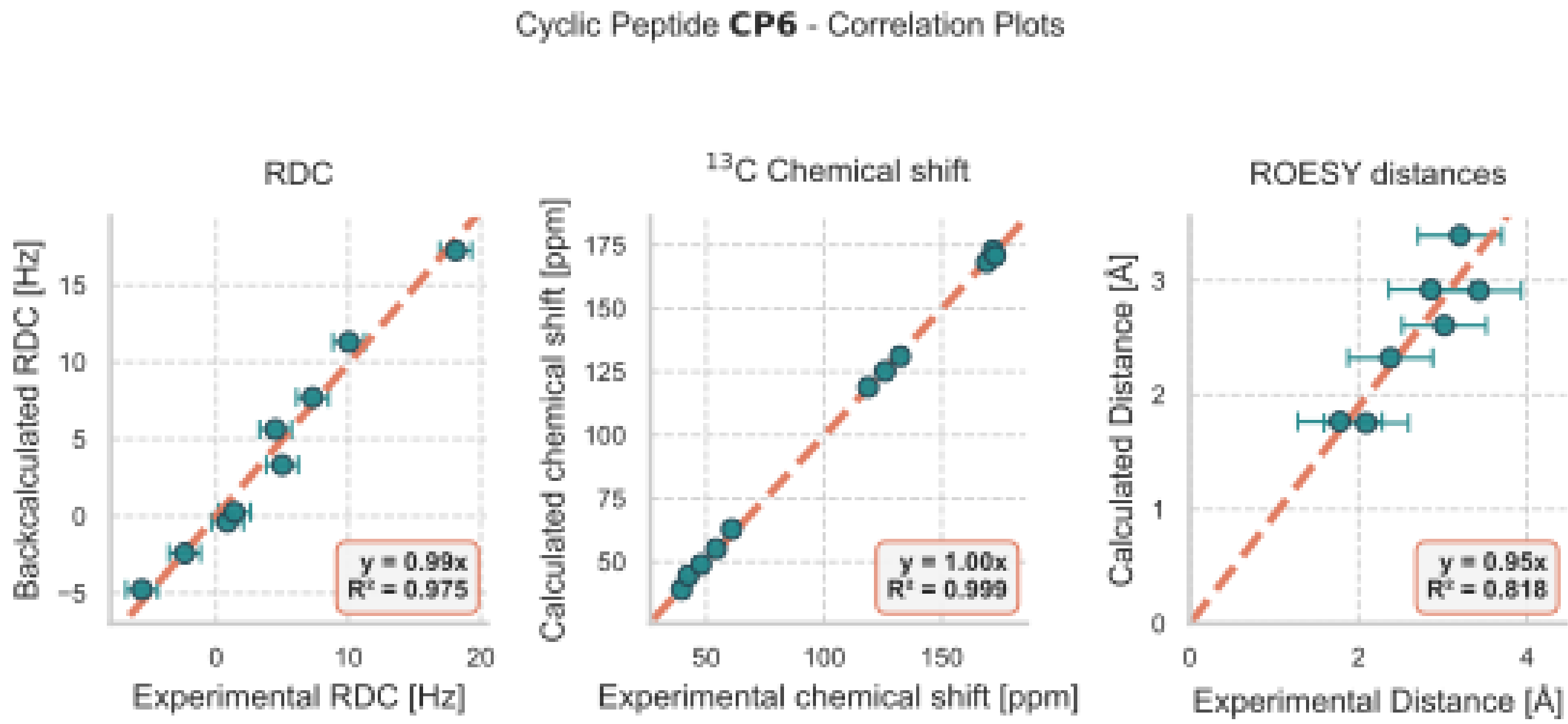

Figure S15. Correlation between experimental NMR data and back-calculated NMR from the conformational ensemble for the first peak set of **CP6** determined using Stereofitter with 9 RDCs. For each plot a linear regression is shown together with its R^2 values.

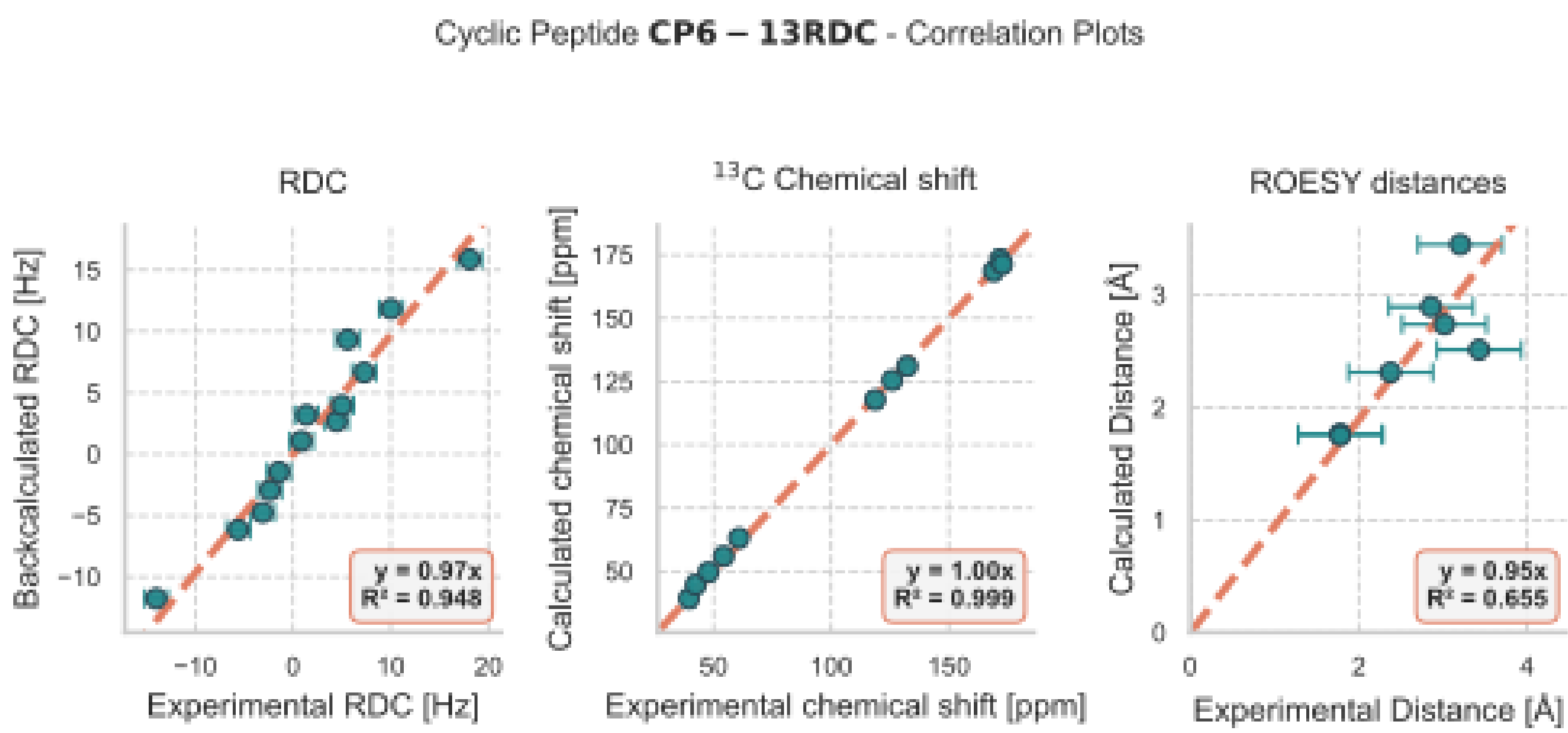

Figure S16. Correlation between experimental NMR data and back-calculated NMR from the conformational ensemble for the first peak set of **CP6** determined using Stereofitter with 13 RDCs. For each plot a linear regression is shown together with its R^2 values.

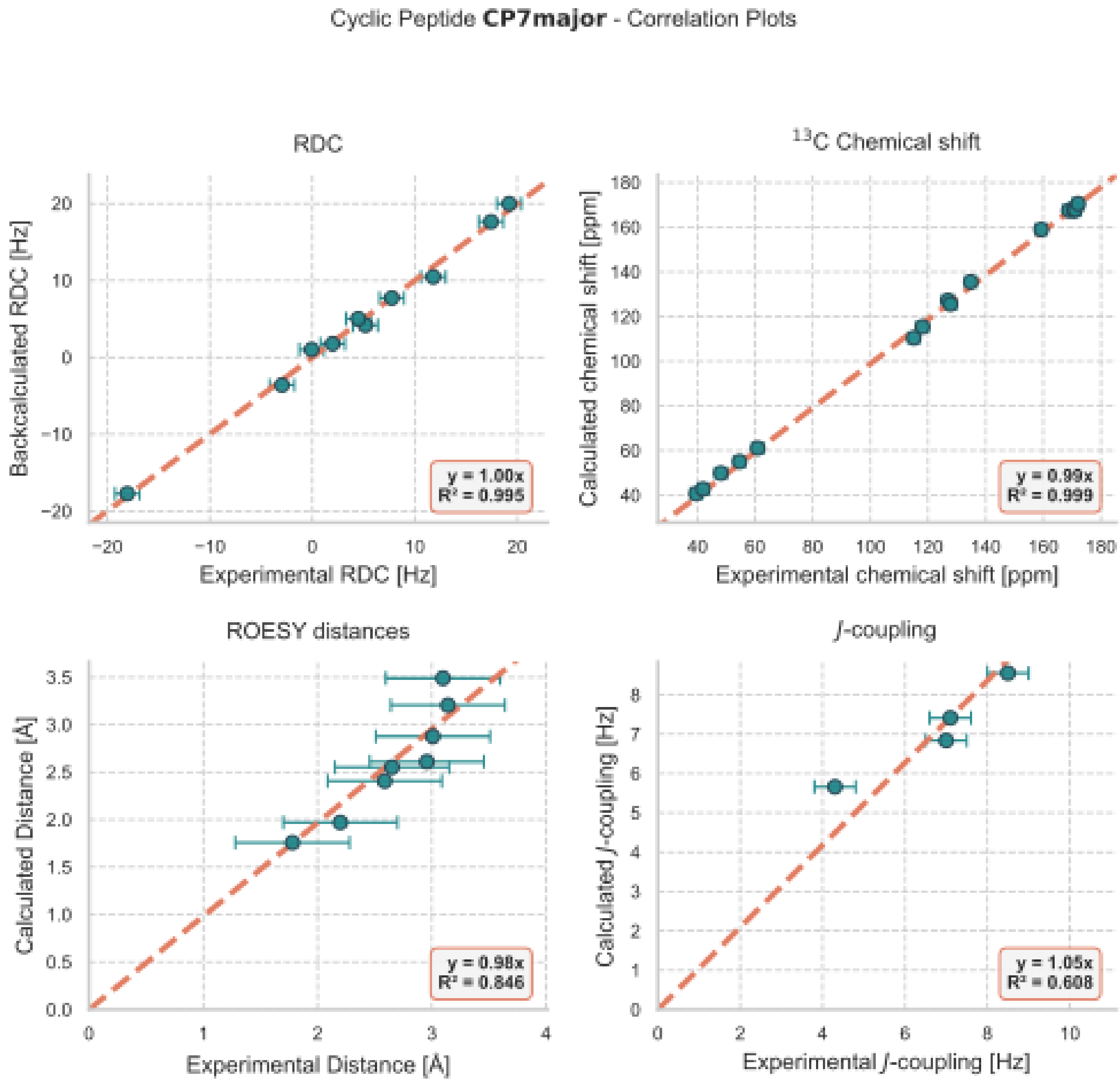

Figure S17. Correlation between experimental NMR data and back-calculated NMR from the conformational ensemble for the first peak set of **CP7.1** determined using Stereofitter. For each plot a linear regression is shown together with its R^2 values.

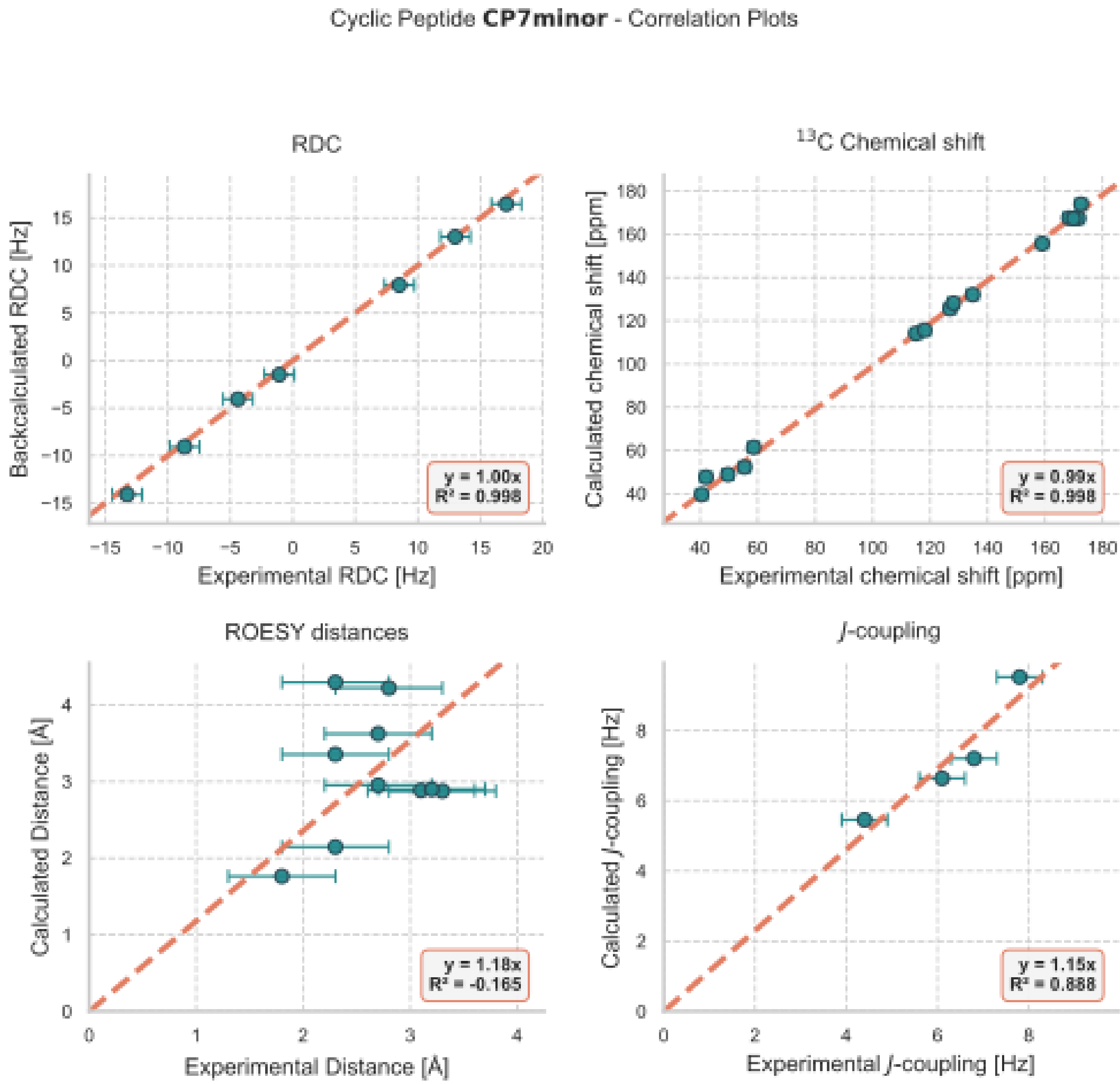

Figure S18. Correlation between experimental NMR data and back-calculated NMR from the conformational ensemble for the first peak set of **CP7.2** determined using Stereofitter. For each plot a linear regression is shown together with its R^2 values.

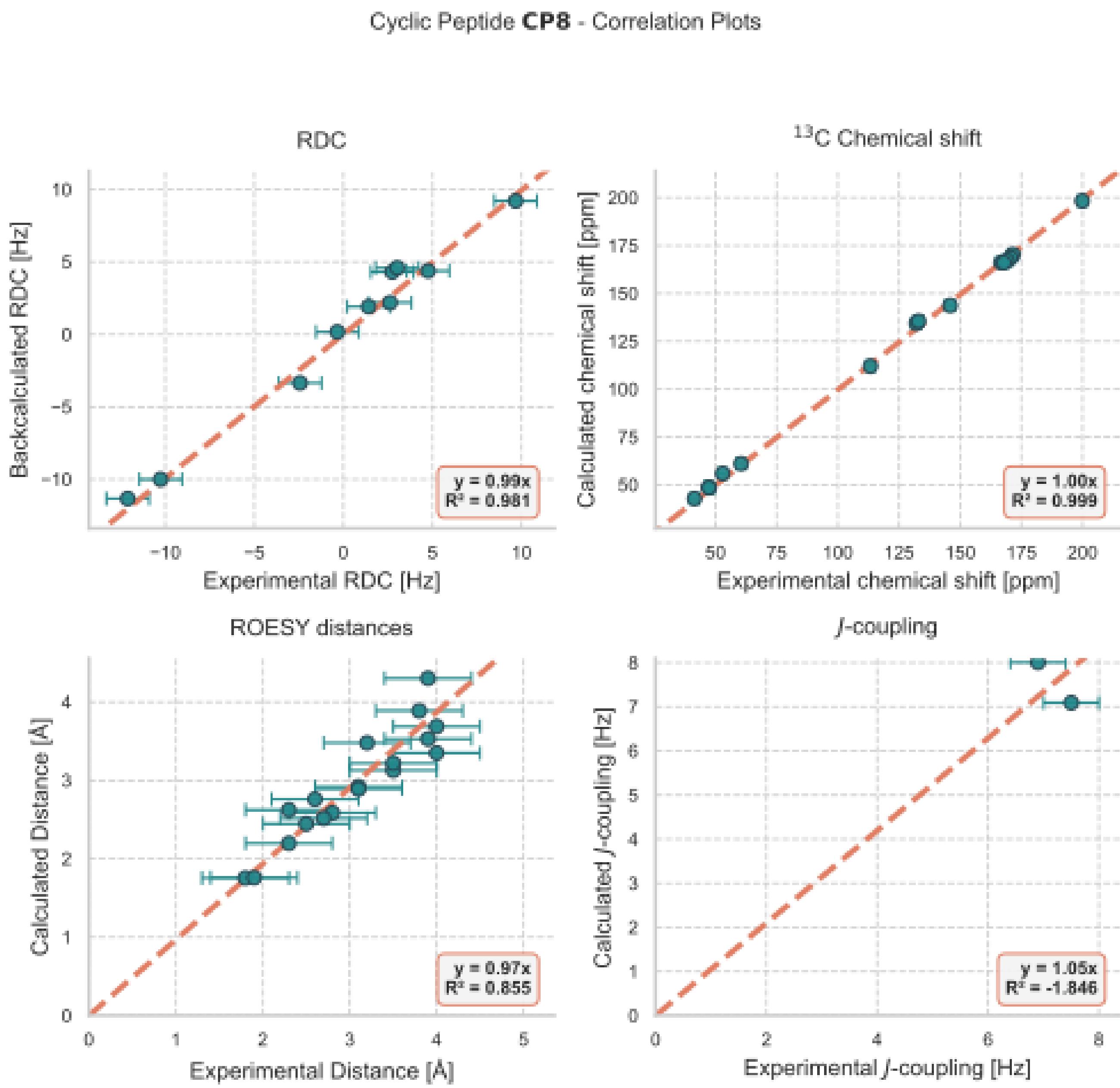

Figure S19. Correlation between experimental NMR data and back-calculated NMR from the conformational ensemble for **CP8** determined using Stereofitter with 10 RDCs. For each plot a linear regression is shown together with its R^2 values.

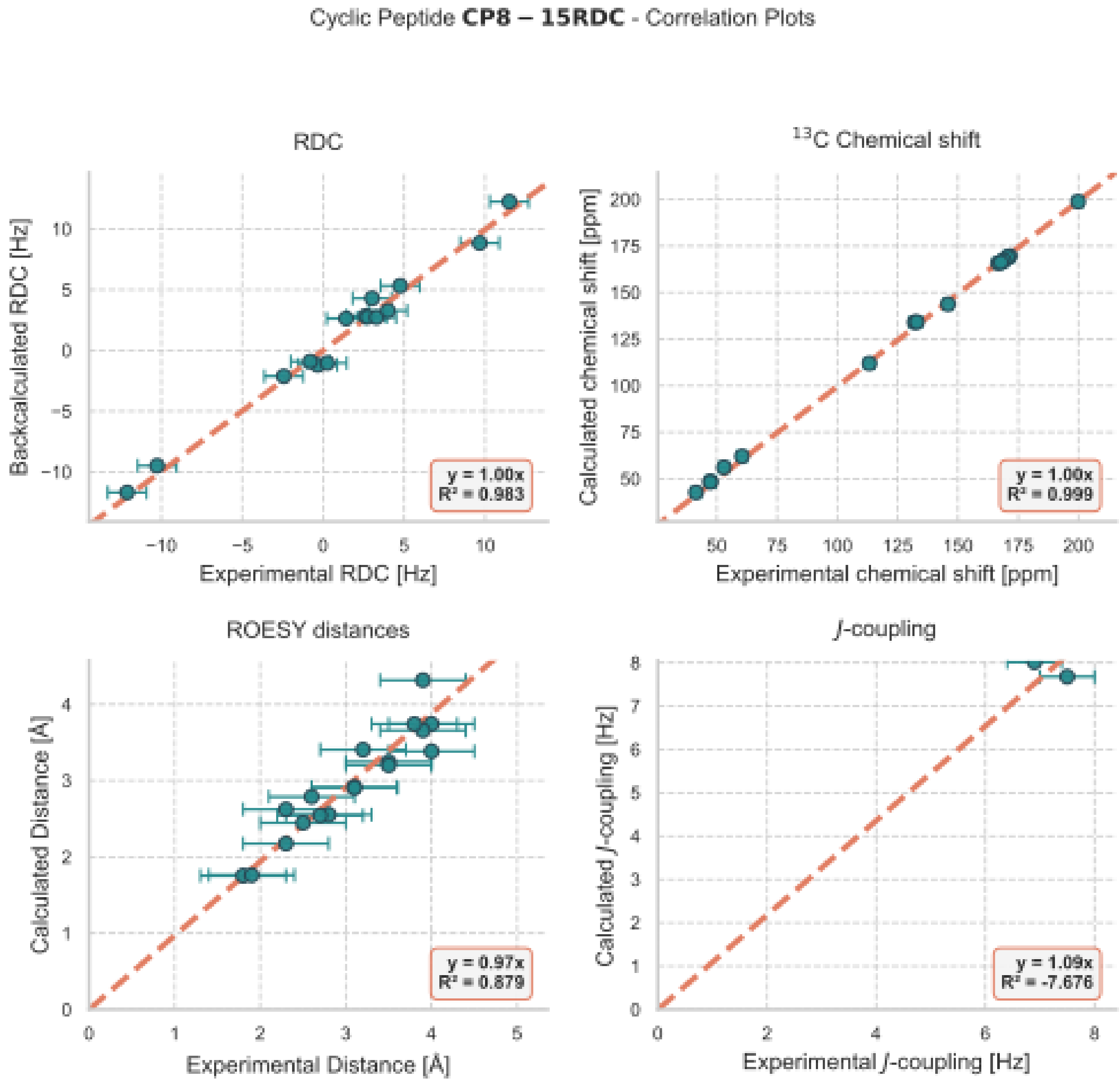

Figure S20. Correlation between experimental NMR data and back-calculated NMR from the conformational ensemble for **CP8** determined using Stereofitter with 15 RDCs. For each plot a linear regression is shown together with its R^2 values.

SI.3 Impact of different NMR parameters on the analysis of CP1

Inspection of the ensembles obtained from different combinations of NMR observables (**Figure S21a**) highlights the dominant influence of the RDC data. In all cases where RDCs were included, the resulting ensembles converged toward similar conformers. Consistent with this observation, the RDC *Q*-factor remained largely unchanged, while the AIC decreased only marginally upon inclusion of additional NMR observables. Notably, the ensemble derived from the combination of ^{13}C chemical shifts, ROEs, and *J*-

couplings resembled the RDC-derived ensemble, exhibiting similar conformational populations in comparable regions of the PCA space. In contrast, the ensemble obtained using only ROEs and *J*-couplings showed a moderate shift. However, the limited number of available restraints (only two *J*-couplings and eight ROE distances) substantially reduces confidence in this result.

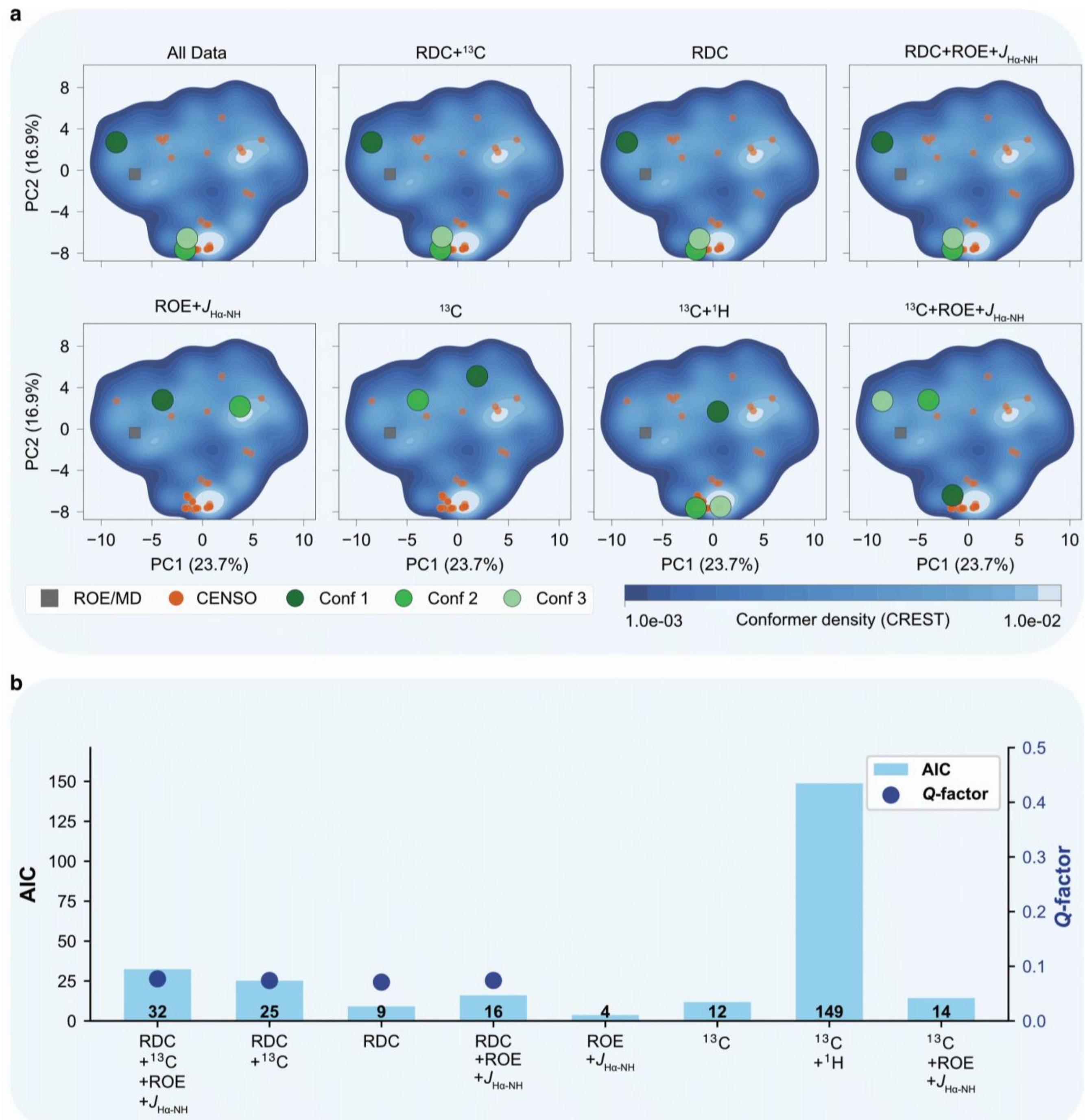

Figure S21. Conformational ensembles and fitting results obtained for **CP1** using different combinations of NMR observables. (**a**) Visualization of the resulting ensembles using the same PCA space as described in **Figure 2**. (**b**) AIC and *Q*-factor for the obtained best-fitting ensembles.

SI.4 Choice of coordinates for the PCA of CP1

Principal component analysis (PCA) can be performed using different coordinate representations. Taking **CP1** as an example, comparison of PCA based on the Cartesian coordinates of the aligned backbone atoms and on the backbone torsion angles reveals notable differences in the resulting conformational landscapes and in the placement of the NMR/DFT conformers within them. In the Cartesian PCA, the most populated CONF1 (50%) is clearly separated, whereas the two minor conformers cluster together. In contrast, the torsion-angle PCA separates the least populated conformer (16%), while the two more populated conformers lie comparably closer to one another. The first two principal components capture less variance in torsional space (25.6%) than in Cartesian space (40.6%). This difference arises because torsion angles represent local, less correlated degrees of freedom, and the sine/cosine embedding distributes the variance across a larger number of components. As a result, no single component dominates the conformational variability to the same extent as in Cartesian PCA, which captures more collective atomic displacements. Although torsion-angle PCA offers the advantage of principal components that are more directly linked to specific structural changes, it is less effective for visualizing overall conformational differences than Cartesian PCA.

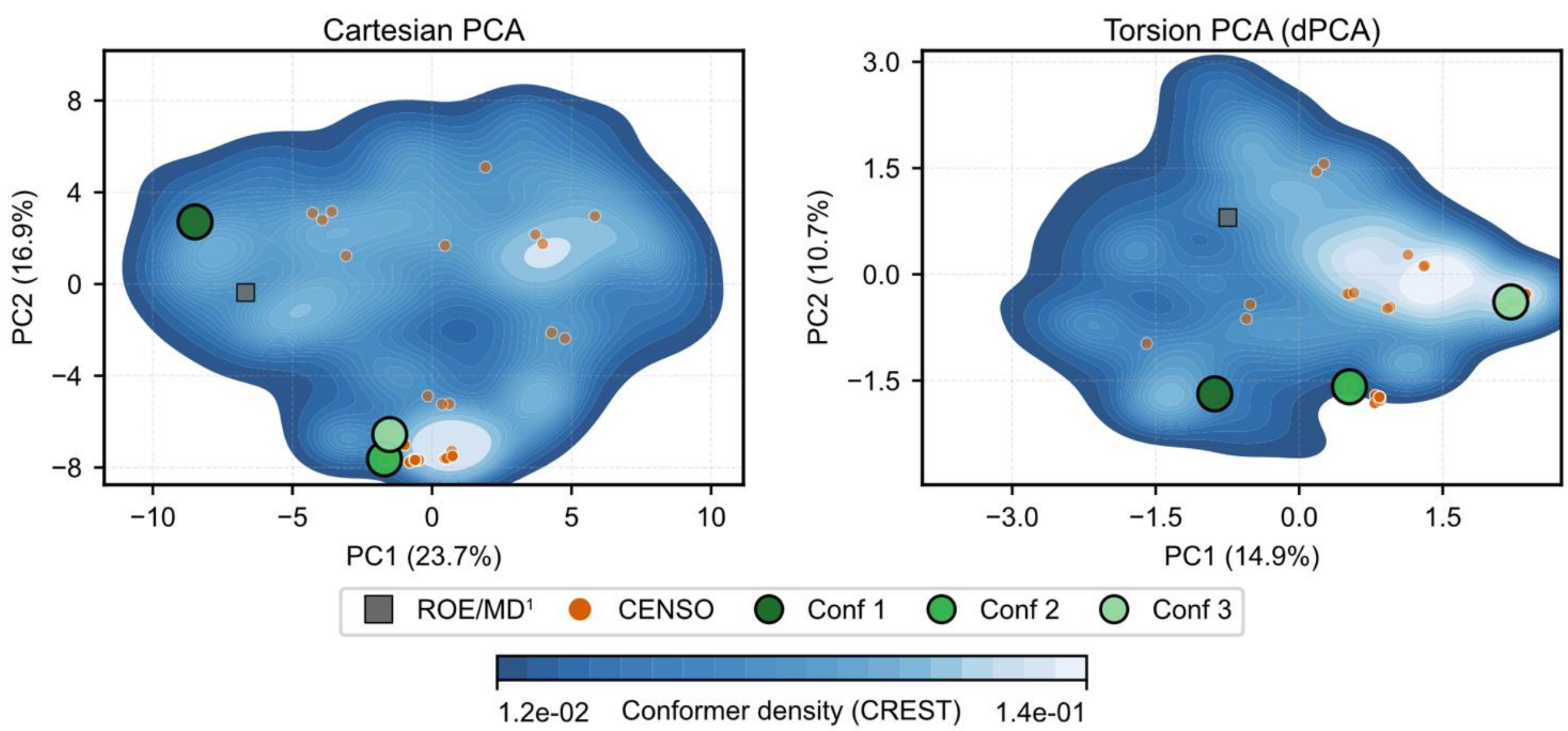

Figure S22. Comparison of the PCA using the Cartesian coordinates of the backbone atoms (left) and the backbone torsion angles (right). The φ, ψ and ω torsions of each residue, together with the endocyclic torsions of the linker, were each defined by four atom indices; prior to PCA the angles were replaced by their sine and cosine components to avoid artifacts from the ±180° periodicity. Green: NMR/DFT conformers; orange: CENSO ensemble; grey square: ROE/MD reference; blue shading: CREST conformer density.

SI.5 DFT calculated chemical shift for *cis*/*trans*-proline

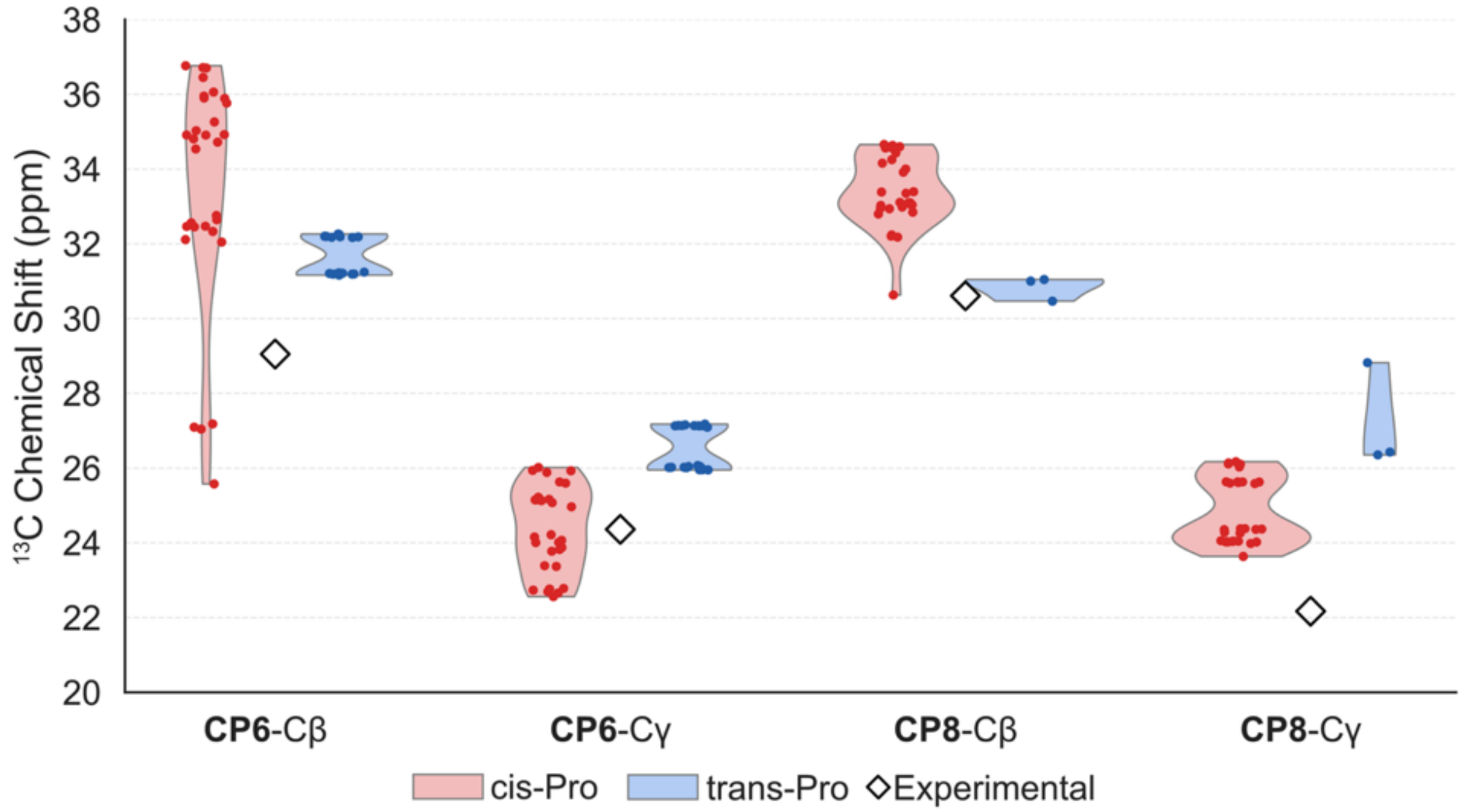

Figure S23. Carbon chemical shift of proline Cβ and Cγ for **CP6** and **CP8**. Experimental shifts were determined in DMSO at 150 MHz (600 MHz ^{1}H) and references to the DMSO solvent peak. DFT chemical shieldings were calculated at the r2SCAN0/pcSseg-2/SMD(DMSO)//PW6B95-*D4*/def2-TZVP/SMD(DMSO) level of theory and converted to shifts using the linear equation parameters from **Table S34**.

SI.6 Transition state analysis of proline *cis/trans*-isomerization

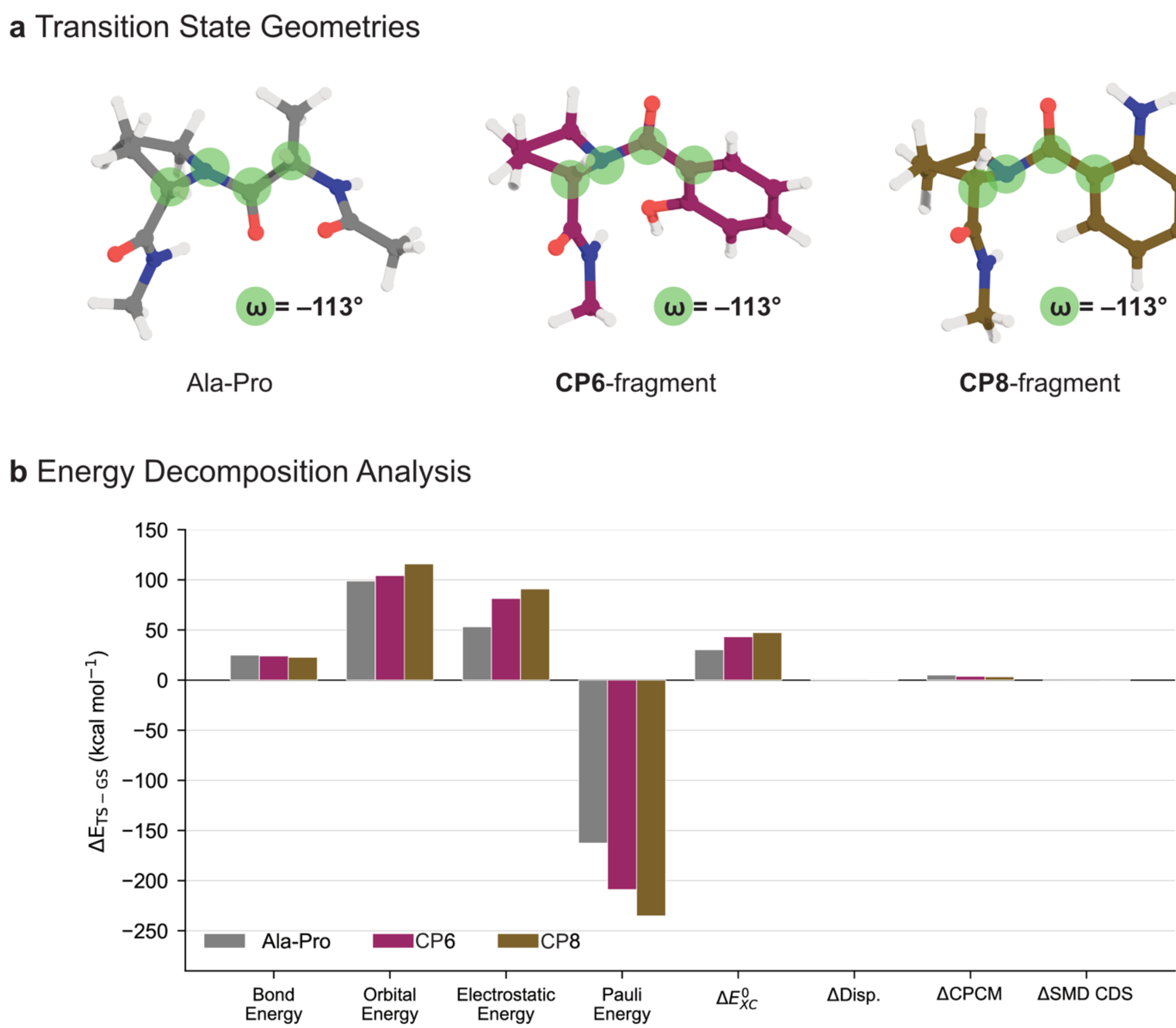

Figure S24. **a**) DFT-calculated transition state geometries for the cis-trans isomerization of proline in the Ala-Pro dipeptide, **CP6** fragment and **CP8** fragment with indicated omega-angles. **b**) Energy decomposition analysis of the transition state DFT-densities in the extended transition state with natural orbital for chemical valency (ETS-NOCV) framework, calculated at the PW6B95-D4/def2-TZVP/SMD(DMSO) level of theory. The depicted energies are the difference between the ground state and transition state.

SI.7 PCA of aryl-containing cyclic peptides CP6–CP8

The most evident result from the comparison of the conformational ensembles of **CP6–CP8** in **Figure S25**, is the clear separation between the *cis*-proline conformers (major conformer of **CP8** and minor conformer of **CP6**) to the *trans*-proline conformers (minor conformers of **CP8** and major conformer of **CP6**). Accordingly, the conformers of **CP7** do lie between them, as it does not contain a proline residue and the substitute Ile residue does not show *cis-trans* isomerization. Interestingly, the two *trans*-proline conformers of **CP8** and **CP6** are very close in the PCA space, suggesting similar overall shape, despite containing either a -NH_2 or -OH group connected to the benzene ring. **CP6** shows higher similarity between the two

conformers in the first principal component than **CP8**, but lower similarity in the second principal competent. This suggests that while **CP8** changes between two rather symmetric states from a down-flip to an up-flip state, while **CP6** remains more towards the up-flip state but changes between a tilted and a symmetric state. The shared PCA analysis shows the disadvantage, that while for **CP6** and **CP8** strong difference in the conformer can be detected, the changes for **CP7** are much less pronounced, as the PCA space is heavily dominated by the contrasting behavior of the *cis*-*trans* isomerization.

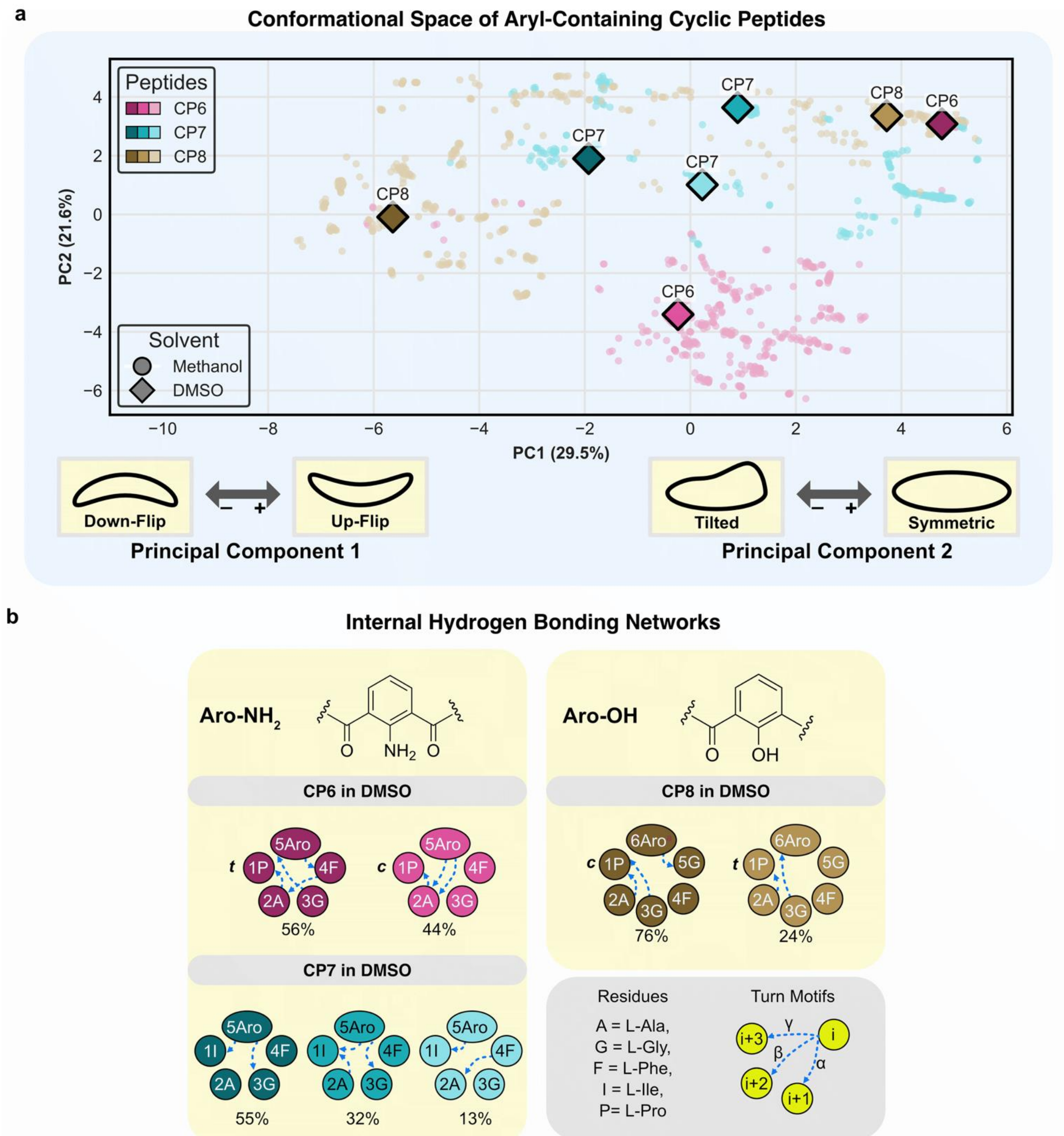

Figure S25. a Principal component analysis (PCA) of backbone-atom coordinates from the CREST (xTB-GFN2/ALPB) ensembles for **CP6–CP8** (PC1, 29.0%; PC2, 21.5%). Small points denote all CREST conformers and the larger circles mark the

NMR/DFT selected conformers. Colors indicate peptides, and darker shades reflect higher population in the *Stereofitter* ensemble. Additionally, rough conformational changes are illustrated for PC1 and PC2. **b** Schematic overview of the internal hydrogen bonds for the NMR/DFT ensembles of **CP6**–**CP8**.

SI.8 Extraction of ROE-derived distances

ROESY spectra have an inherent offset dependency, which results in distorted distances when converting intensities to distances. This dependency was mitigated by taking the dependency into account in the following equation.[3]

$$r_{ij} = r_{ref}\left(\frac{a_{ref}c_{ref}}{a_{ij}c_{ij}}\right)^{1/6},$$

where r_{ij} is the distances between two hydrogens, r_{ref} is the distances of a reference proton pair, a is the ROESY peak volume and c is a correction factor. The correction factors are calculated for each nuclei pair by,

$$c_{ij} = \frac{1}{\sin^2\theta_i \sin^2\theta_j},$$

with

$$\tan\theta_i = \frac{\gamma B_1}{(\omega_i - \omega_0)},$$

where γB_1 is the field strength of the ROESY spin-lock, ω_i is the frequency of nucleus i and ω_0 is the frequency of the offset. For **CP2**, **CP4** and **CP5**, the distances have been calculated accordingly. For **CP1**, **CP3** and **CP6–CP8** EASY-ROESY[4] experiments have been used, which are not as affected by offset dependency and the volumes have been converted to distances without the correction factors. In both cases the peak intensities for the mirror symmetric cross-peaks have been averaged, if both were well separated.

Table S10. EASY-ROESY peak integral from MNOVA 15.1 and distances for cyclic peptide **CP1** in DMSO at 300K. The orange highlighted peak has been used as reference and set to 1.78 Å for a methylene group.

Hydrogen 1	Hydrogen 1 Chemical Shift in [ppm]	Hydrogen 2	Hydrogen 2 Chemical Shift in [ppm]	Absolute Peak Intensity (a_{ij})	Distances in [Å]
53	9.77	49	7.66	-118.16	2.57
53	9.77	52	8.17	-162.87	2.43
53	9.77	75	4.18	-6.85	4.12
76	8.23	77	4.33	-162.98	2.43
76	8.23	75	4.18	-63.61	2.85

74	8.14	75	4.18	-171.78	2.41
93	8.06	59	3.77	-45.02	3.01
93	8.06	91,92	3.65/4.12	-166.26/-66.81	2.6
78	7.71	77	4.33	-48.15	2.98
78	7.71	91,92	3.65/4.12	-63.52/-126.87	2.7
60	2.91	61	2.60	-1060.66	1.78

Table S11. ROESY peak integral from TopSpin 4.3 and offset corrected distances for cyclic peptide **CP2** in methanol at 300K. The orange highlighted peak has been used as reference and set to 1.78 Å for a methylene group.

Hydrogen 1	Hydrogen 1 Chemical Shift in [ppm]	Hydrogen 2	Hydrogen 2 Chemical Shift in [ppm]	Absolute Peak Intensity (a_{ij})	Correction factor (c_{ij})	Distances in [Å]
58	9.65	103	8.39	-23203000	4.23	2.2
58	9.65	56	7.66	-17353000	3.55	2.4
58	9.65	60	7.41	-19201500	3.35	2.4
58	9.65	59	4.50	-15655200	2.42	2.6
58	9.65	67	4.27	-5729950	2.46	3.0
62	8.74	72	8.32	-12400600	3.31	2.6
62	8.74	60	7.42	-23973000	2.66	2.4
62	8.74	61	4.57	-10598400	1.93	2.9
62	8.74	66	4.35	-16041500	1.95	2.7
75	8.48	92	3.46	-14728500	2.03	2.7
72	8.27	78	1.68	-40534500	2.82	2.1
60	7.41	73	3.78	-2872200	1.50	4.0
60	7.42	(68,69,70)	1.20	-10136950	2.59	2.7
59	4.49	(68,69,70)	1.20	-18352500	1.88	2.6
85	3.78	92	3.47	-15929500	1.22	2.9
85	3.78	(86,87)	2.87/2.64	-3274300/ -28307500	1.36/1.43	3.2
85	3.78	(95,96,97)	0.79	-18741500	2.23	2.6
86	2.87	87	2.63	-212900000	1.67	1.78

Table S12. EASY-ROESY peak integral from MNOVA 15.1 and distances for cyclic peptide **CP3** in methanol at 300K. The orange highlighted peak has been used as reference and set to 1.78 Å for a methylene group.

Hydrogen 1	Hydrogen 1 Chemical Shift in [ppm]	Hydrogen 2	Hydrogen 2 Chemical Shift in [ppm]	Absolute Peak Intensity (a_{ij})	Distances in [Å]
83	9.84	71	8.37	-24.55	3.6
83	9.84	44	7.79	-82.44	2.9
83	9.84	70	4.41	-6.2	4.5
83	9.84	47	8.41	-95.18	2.9
66	8.19	70	4.39	-73.68	3.0
66	8.19	65	4.45	-182.04	2.6
66	8.19	64	7.95	-19.63	3.7
64	7.96	70	4.43	-53.46	3.2
64	7.96	48	3.97	-37.86	3.3
48	3.97	55	3.30	-176.7	2.6
49	2.99	50	2.65	-1943.75	1.78

Table S13. ROESY peak integral from TopSpin 4.3 and offset corrected distances for cyclic peptide **CP4** in methanol at 300K. The orange highlighted peak has been used as reference and set to 1.78 Å for a methylene group.

Hydrogen 1	Hydrogen 1 Chemical Shift in [ppm]	Hydrogen 2	Hydrogen 2 Chemical Shift in [ppm]	Absolute Peak Intensity (a_{ij})	Correction factor (c_{ij})	Distances in [Å]
72	8.39	47	3.55	-3630650	1.94	3.1
48	3.99	(75,76,77)	0.79	-10158000	2.24	2.6
48	3.99	47	3.54	-7694650	1.20	3.0
48	3.99	(78,79)	2.86/2.61	-2081000/-13155000	1.37/1.44	3.1
47	3.55	(78,79)	2.85/2.61	-3185850/-1131700	1.53/1.46	3.7
78	2.85	79	2.61	-114215000	1.74	1.78

Table S14. ROESY peak integral from TopSpin 4.3 and offset corrected distances for cyclic peptide **CP5** in DMSO at 300K. The orange highlighted peak has been used as reference and set to 1.78 Å for a methylene group.

Hydrogen 1	Hydrogen 1 Chemical Shift in [ppm]	Hydrogen 2	Hydrogen 2 Chemical Shift in [ppm]	Absolute Peak Intensity (a_{ij})	Correction factor (c_{ij})	Distances in [Å]
46	7.63	48	9.58	-66200000	3.30	2.0
47	8.41	48	9.58	-4000000	3.99	3.1

48	9.58	(50,49)	4.12/3.65	-19900000/ -21600000	2.42/2.57	2.6
48	9.58	52	4.00	-74600000	2.45	3.0
51	8.92	52	4.00	-136000000	2.08	1.9
52	4.00	53	8.21	-49500000	2.35	2.3
53	8.21	63	4.51	-43200000	1.67	2.4
63	4.51	64	8.11	-27000000	1.63	2.6
64	8.11	(68,69)	3.51/3.59	-83400000/- 76000000	1.83/1.80	2.1
70	7.85	71	3.78	-145000000	1.65	2.0
72	3.12	73	2.76	-285000000	1.60	1.78
51	8.92	48	9.58	-40400000	4.52	2.1

Table S15. EASY-ROESY peak integral from MNOVA 15.1 and distances of cyclic peptide **CP6** in DMSO at 300K. The orange highlighted peak has been used as reference and set to 1.78 Å for a methylene group.

Hydrogen 1	Hydrogen 1 Chemical Shift in [ppm]	Hydrogen 2	Hydrogen 2 Chemical Shift in [ppm]	Absolute Peak Intensity (a_{ij})	Distances in [Å]
42	9.78	59	7.42	-48.315	3.0
42	9.78	45	8.68	-22.435	3.4
45	8.68	46	4.35	-201.9	2.4
59	7.42	58	4.26	-67.025	2.9
59	7.42	63	4.36	-33.695	3.2
55	3.79	56	3.25	-1146.255	1.78
48	2.97	49	2.83	-435.08	2.1

Table S16. EASY-ROESY peak integral from MNOVA 15.1 and distances for the first peak set of cyclic peptide **CP7** in DMSO at 300K. The orange highlighted peak has been used as reference and set to 1.78 Å for a methylene group.

Hydrogen 1	Hydrogen 1 Chemical Shift in [ppm]	Hydrogen 2	Hydrogen 2 Chemical Shift in [ppm]	Absolute Peak Intensity (a_{ij})	Distances in [Å]
44	8.78	46	1.99	-305.69	2.6
44	8.78	45	3.93	-123.52	3.0
44	8.78	55	7.68	-264.08	2.7
44	8.78	40	7.79	-809.86	2.2

45	3.93	46	1.99	-137.265	3.0
55	7.68	46	1.99	-95.565	3.1
55	7.68	45	3.93	-103.715	3.1
67	4.38	66	4.04	-2879.65	1.78

Table S17. EASY-ROESY peak integral from MNOVA 15.1 and distances for the second peak set of cyclic peptide **CP7** in DMSO at 300K. The orange highlighted peak has been used as reference and set to 1.78 Å for a methylene group.

Hydrogen 1	Hydrogen 1 Chemical Shift in [ppm]	Hydrogen 2	Hydrogen 2 Chemical Shift in [ppm]	Absolute Peak Intensity (a_{ij})	Distances in [Å]
44	9.10	40	7.93	-102.37	2.3
44	9.09	46	2.04	-33.93	2.7
55	8.71	40	7.92	-85.555	2.3
55	8.70	60	6.95	-26.665	2.8
55	8.70	56	4.14	-11.32	3.3
55	8.70	45	4.23	-87.69	2.3
63	8.16	64	4.19	-36.74	2.7
60	6.95	56	4.15	-14.49	3.1
45	4.23	44	9.10	-11.465	3.2
61	3.67	62	3.40	-420.295	1.78

Table S18. EASY-ROESY peak integral from MNOVA 15.1 and distances for cyclic peptide **CP8** in DMSO at 300K. The orange highlighted peak has been used as reference and set to 1.78 Å for a methylene group.

Hydrogen 1	Hydrogen 1 Chemical Shift in [ppm]	Hydrogen 2	Hydrogen 2 Chemical Shift in [ppm]	Absolute Peak Intensity (a_{ij})	Distances in [Å]
54	8.93	55	3.21	-172.865	2.8
54	8.93	43	7.37	-44.265	3.5
54	8.93	53	4.44	-538.11	2.3
52	8.55	42	6.36	-19.025	4.0
52	8.55	49	6.68	-18.665	4.0
52	8.55	53	4.44	-87.435	3.1
52	8.55	43	7.37	-23.445	3.9
47	8.32	(57,58,59)	1.18	-240.27	2.6

47	8.32	46	3.99	-72.66	3.2
47	8.32	49	6.68	-40.625	3.5
47	8.32	48	4.21	-95.895	3.1
43	7.37	42	6.36	-336.73	2.5
49	6.68	48	4.21	-211.38	2.7
49	6.68	(57,58,59)	1.18	-22.00	3.9
49	6.68	46	3.99	-28.055	3.8
48	4.21	(57,58,59)	1.18	-487.52	2.3
60	3.01	61	2.81	-2460.895	1.78
67	2.14	68	1.96	-1765.185	1.9

SI.9 Extraction of amide proton temperature coefficients

Amide proton temperature coefficients have been obtained by measuring ^1H spectra at different temperatures. For methanol a temperature range of 288–315 K was chosen. For DMSO the temperature range of 295-319 was chosen, to avoid freezing of the sample. For most CPs steps of 2 K were measured, for **CP3** 5 K steps were measured. The shifts at different temperature are listed in the **Tables S19-S27** below.

Table S19. Amide proton temperature coefficients of cyclic peptide **CP1** in DMSO.

Temperature in [K]	2Gly-NH	3Ala-NH	4Leu-NH	5Gly-NH	6BiA-NH
295	8.0714	7.7364	8.241	8.1476	9.7896
297	8.0672	7.7276	8.2359	8.1426	9.7786
299	8.0628	7.7189	8.2311	8.1374	9.7692
301	8.0594	7.7107	8.2263	8.1315	9.7591
303	8.0545	7.7023	8.2194	8.1264	9.7493
305	8.0497	7.694	8.2146	8.1209	9.74
307	8.0449			8.1145	9.7294
309	8.0408		8.2028	8.1096	9.7204
311	8.037		8.1938	8.1045	9.7115
313	8.0323			8.0977	9.7009
315	8.028			8.0929	9.692
317	8.02	7.65		8.09	9.68

319	8.0139	7.6387		8.0812	9.6737
$\Delta\delta_{NH}$ in (ppb/K)	-2.3	-4.1	-2.9	-2.8	-4.8

Table S20. Amide proton temperature coefficients of cyclic peptide **CP2** in methanol.

Temperature in [K]	3Leu-NH	4Glu-NH	2Gly-NH	5Thr-NH	6BiA-NH
288	8.380	8.682	8.504	7.413	9.707
290	8.367	8.675	8.497	7.413	9.695
292	8.352	8.667	8.490	7.413	9.683
294	8.339	8.660	8.483	7.411	9.671
296	8.325	8.653	8.477	7.411	9.659
298	8.311	8.646	8.469	7.410	9.648
300	8.297	8.638	8.463	7.410	9.636
302	8.286	8.632	8.456	7.409	9.625
304	8.268	8.624	8.449	7.409	9.613
306		8.617	8.443	7.408	9.602
308	8.241	8.610	8.436	7.407	9.590
310	8.229	8.603	8.429	7.407	9.579
312	8.214	8.595	8.422	7.406	9.568
314	8.202	8.588	8.415	7.406	9.557
315	8.195	8.584	8.411	7.405	9.552
$\Delta\delta_{NH}$ in (ppb/K)	-6.90	-3.61	-3.42	-0.28	-5.74

Table S21. Amide proton temperature coefficients of cyclic peptide **CP3** in methanol.

Temperature in [K]	2Gly-NH	3Ala-NH	4Leu-NH	5Gly-NH	6BiA-NH
290	8.514	8.053	8.253	8.419	9.919
295	8.494	8.011	8.230	8.408	9.882
300	8.474	7.970	8.206	8.396	9.846
305	8.453	7.931	8.182	8.382	9.811
310	8.433	7.893	8.157	8.367	9.776
315	8.411	7.853	8.131	8.350	9.742
$\Delta\delta_{NH}$ in (ppb/K)	-4.12	-7.95	-4.85	-2.75	-7.06

Table S22. Amide proton temperature coefficients of cyclic peptide **CP4** in methanol.

Temperature in [K]	2Gly-NH	3Ala-NH	4Leu-NH	5Gly-NH	6BiA-NH
288	8.427	8.258	7.896	8.996	9.500
290	8.417	8.243	7.887	8.985	9.483
292	8.414	8.230	7.878	8.974	9.469
294	8.407	8.216	7.869	8.962	9.454
296	8.401	8.203	7.860	8.951	9.441
298					
300	8.389	8.177	7.842	8.929	9.410
302	8.383	8.164	7.833	8.919	9.396
304	8.377	8.151	7.824	8.905	9.389
306	8.371	8.138	7.816	8.895	9.372
308	8.364	8.125	7.807	8.882	9.361
310	8.358	8.112	7.798	8.870	9.343
312	8.349	8.099	7.789	8.858	9.330
314	8.344	8.087	7.781	8.848	9.315
315	8.340	8.081	7.777	8.840	9.312
$\Delta\delta_{NH}$ in (ppb/K)	-5.76	-3.14	-6.55	-4.43	-6.90

Table S23. Amide proton temperature coefficients of cyclic peptide **CP5** in DMSO.

Temperature in [K]	2Gly-NH	3Ala-NH	4Leu-NH	5Gly-NH	6BiA-NH
295	7.862	8.119	8.214	8.919	9.570
297	7.852	8.109	8.206	8.911	9.564
299	7.842	8.098	8.198	8.903	9.559
301	7.832	8.087	8.187	8.895	9.553
303	7.822	8.076	8.179	8.887	9.548
305	7.812	8.065	8.171	8.878	9.542
307		8.0540	8.163	8.870	9.536
309		8.0429	8.155	8.862	9.530
311		8.0317	8.1465	8.853	9.525
313		8.021	8.138	8.845	9.519
315	7.763	8.009	8.130	8.837	9.513

317	7.754	7.998	8.122	8.828	9.508
319	7.744	7.987	8.114	8.820	9.502
$\Delta\delta_{NH}$ in (ppb/K)	-4.90	-4.13	-4.15	-5.52	-2.84

Table S24. Amide proton temperature coefficients of the first peak set of cyclic peptide **CP6** in DMSO.

Temperature in [K]	1Pro-NH	2Ala-NH	3Gly-NH	4Phe-NH	5Aro-NH	5Aro-OH
295	–	7.4130	8.0926	8.1346	8.7256	9.7404
297	–	7.4172	8.0765	8.1172	8.715	9.7574
299	–	7.4249	8.0615	8.1003	8.7017	9.774
301	–	7.4295	8.0496	8.0832	8.6881	9.7901
303	–	7.4337	8.0340	8.0663	8.6753	9.8056
305	–			8.0472	8.663	9.8203
307	–			8.0298	8.6507	9.8345
309	–				8.6367	9.8479
311	–			7.9839	8.629	9.8614
313	–			7.9697	8.6146	9.8729
315	–			7.9576	8.6024	9.8843
317	–			7.9452	8.5915	9.895
319	–			7.9331	8.5808	9.9017
$\Delta\delta_{NH}$ in (ppb/K)	–	2.7	-7.2	-8.7	-6.1	6.8

Table S25. Amide proton temperature coefficients of the major peak set of cyclic peptide **CP7** in DMSO.

Temperature in [K]	1Ile-NH	2Ala-NH	3Gly-NH	4Phe-NH	5Aro-NH	5Aro-OH
295	8.8	7.6988		8.3233		12.351
297	8.7955	7.6993		8.3128		12.347
299	8.788	7.6985		8.3034		12.343
301	8.781	7.697		8.295		12.3388
303	8.775	7.6968		8.2867		12.3349
305	8.767			8.2761		12.3307
307	8.76			8.2667		12.3267
309	8.753			8.2584		12.3211

311	8.746			8.2498		12.3166
313	8.738	7.6931		8.2381		12.3111
315	8.731	7.6917		8.2301		12.3064
317	8.724	7.6889		8.2207		12.3003
319	8.715	7.6864		8.2107		12.2959
$\Delta\delta_{NH}$ in (ppb/K)	-3.56	-0.48		-4.65		-2.31

Table S26. Amide proton temperature coefficients of the minor peak set of cyclic peptide **CP7** in DMSO.

Temperature in [K]	1Ile-NH	2Ala-NH	3Gly-NH	4Phe-NH	5Aro-NH	5Aro-OH
295	9.12	8.733	8.014	8.1854	6.951	12.5058
297	9.11	8.722	8.009	8.179	6.952	12.4975
299	9.1067	8.709	8.004	8.1729	6.951	12.4895
301	9.1	8.696	7.997	8.1651	6.951	12.4796
303	9.092	8.685	7.992	8.15787	6.951	12.4707
305	9.086	8.663	7.988	8.1526	6.951	12.4623
307	9.08	8.66	7.982	8.1445	6.951	12.4528
309	9.072	8.648	7.975	8.1379	6.95	12.4437
311	9.066	8.635	7.97	8.1309	6.949	12.4344
313	9.06	8.621	7.966	8.1243	6.949	12.4253
315	9.051	8.607	7.961	8.1159	6.948	12.416
317	9.043	8.596	7.955	8.1096	6.948	12.4061
319	9.037	8.583	7.952	8.1037	6.947	12.3958
$\Delta\delta_{NH}$ in (ppb/K)	-3.40	-6.26	-2.65	-3.45	-0.18	-4.57

Table S27. Amide proton temperature coefficients of cyclic peptide **CP8** in DMSO.

Temperature in [K]	2Ala-Nh	3Gly-NH	4Phe-NH	5Aro-NH	5Aro-NH_2
301	8.332	6.697	8.553	8.928	6.948
303	8.314	6.702	8.54	8.91	6.94
305	8.299	6.703	8.525	8.897	6.929
307	8.283	6.705	8.508	8.879	6.916
309	8.267	6.706	8.489	8.866	6.906
311	8.252	6.708	8.48	8.849	6.896

313	8.236	6.711	8.461	8.832	6.888
315	8.226	6.713	8.448	8.818	6.881
317	8.204	6.715	8.436	8.802	6.872
319	8.191	6.718	8.417	8.785	6.863
$\Delta\delta_{NH}$ in (ppb/K)	-7.76	1.06	-7.54	-7.87	-4.78

SI.10 Residual dipolar coupling analysis

RDCs have been calculated by the difference between the one-bond carbon-hydrogen coupling constant, as described in the following equation,

$$^{1}D_{CH} = {}^{1}T_{CH} - {}^{1}J_{CH},$$

where *D* is the dipolar coupling component, *J* the scalar coupling component and *T* the total coupling constant. Experimentally, *J* was measured by isotropic F2-coupled CLIP-HSQC spectra. Afterwards, the alignment media AAKLVFF[5] or OPA[6] have been added to the sample. Both media form liquid crystalline phases. AAKLVFF has been used in methanol at a concentration of 28.8 g/L, while OPA has been used in DMSO at 43 g/L and in methanol at 30 g/L concentration. The degree of alignment was monitored, by monitoring the splitting of the ^{2}H-signal of the solvent signal. In all cases good splitting of the ^{2}H-signal indicated successful generation of a stable but weak anisotropic phases, see **Figure S26**. It should be noted that for the cases **CP2** and **CP4** in methanol-*d3* the splitting of the -OH signal cannot be monitored in the ^{2}H-spectrum, therefore the procedure has been done in the same way in methanol-*d4* and the degree of alignment has been assumed to be similar. The final RDC's were in both cases extracted from the methanol-*d3* measurements. Finally, by measuring again the coupling constant from F2-coupled CLIP-HSQC spectra, *T* was determined from which the RDC restraints *D* used later in Stereofitter could be calculated.

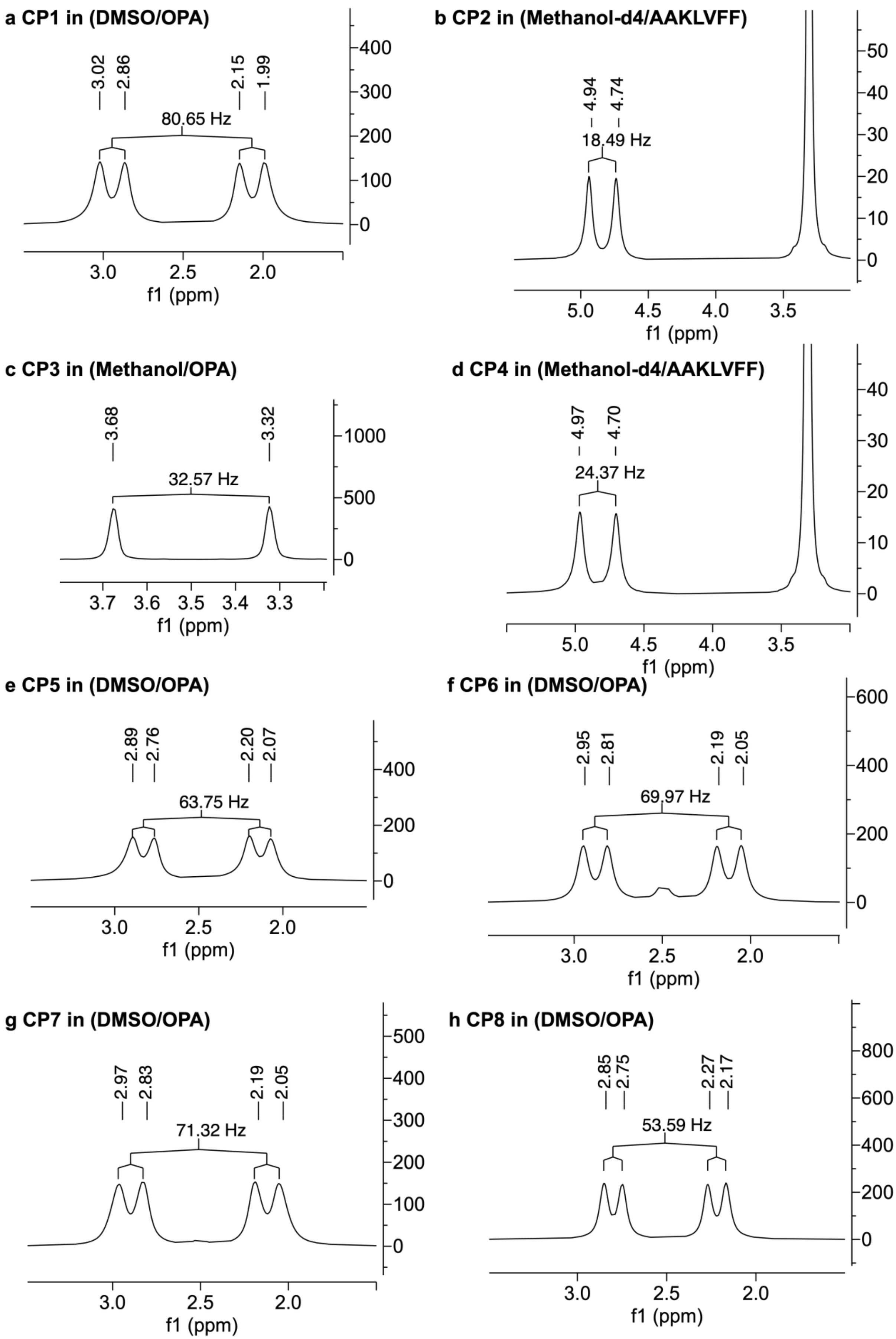

Figure S26. ^{2}H-spectra of the fully anisotropic phase using AAKLVFF or OPA as alignment media for cyclic peptides **CP1-**

CP8.

SI.11 Determined conformational ensembles for CP2, CP3, CP4, CP5, CP6, CP7, CP8

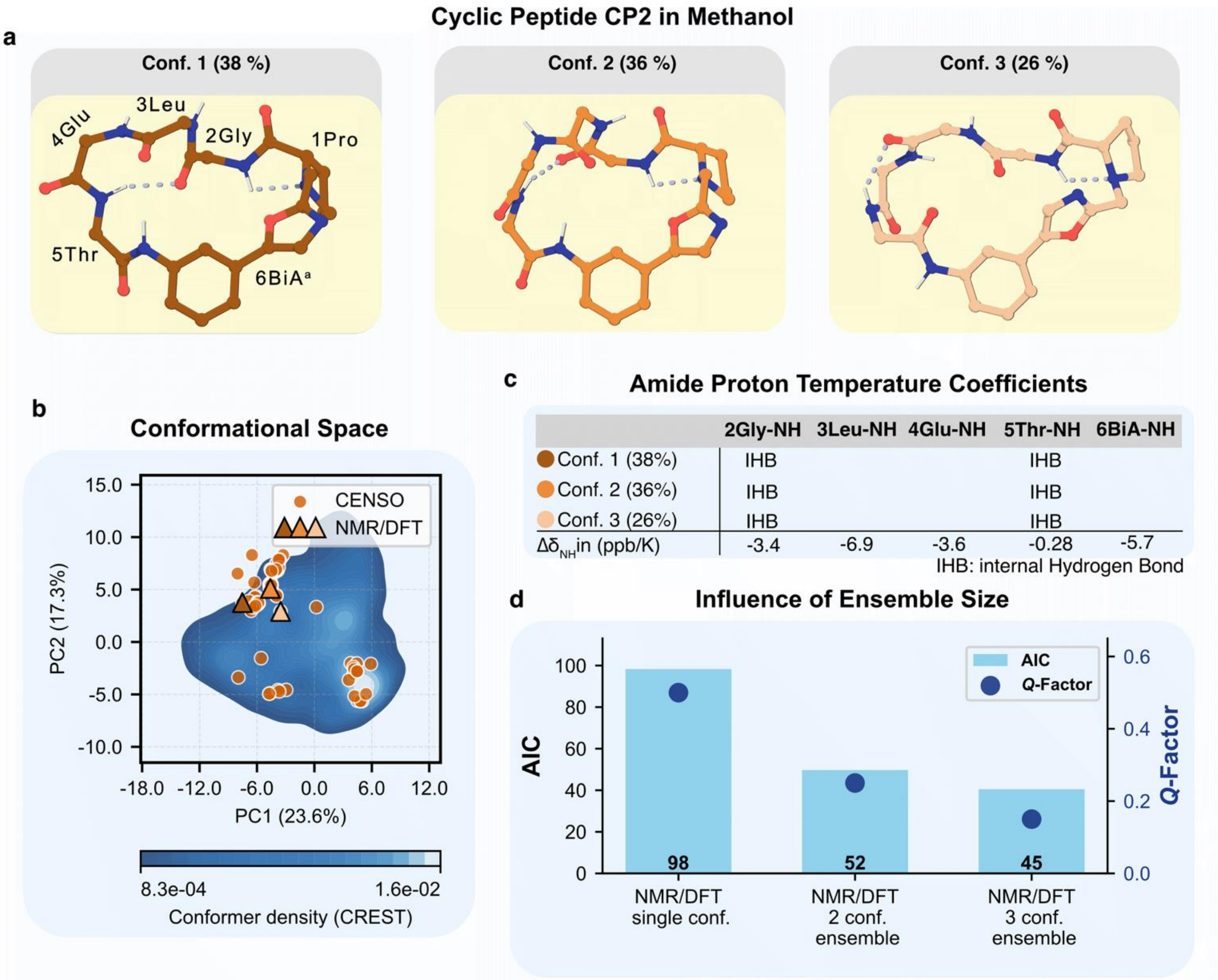

Figure S27. Stereofitter ensemble obtained for **CP2** in methanol. **a** Structures of the three conformers with indicated internal hydrogen bonds and population in the Stereofitter ensemble. **b** Conformational space of **CP5** in methanol spanned by the CREST sampling, CENSO refinement and the selected conformers. **c** Amide proton temperature coefficient measured together with indications which amide proton of the determined conformer is likely to be involved in internal hydrogen bonds. **d** Statistical data from Stereofitter when increasing the ensemble size.

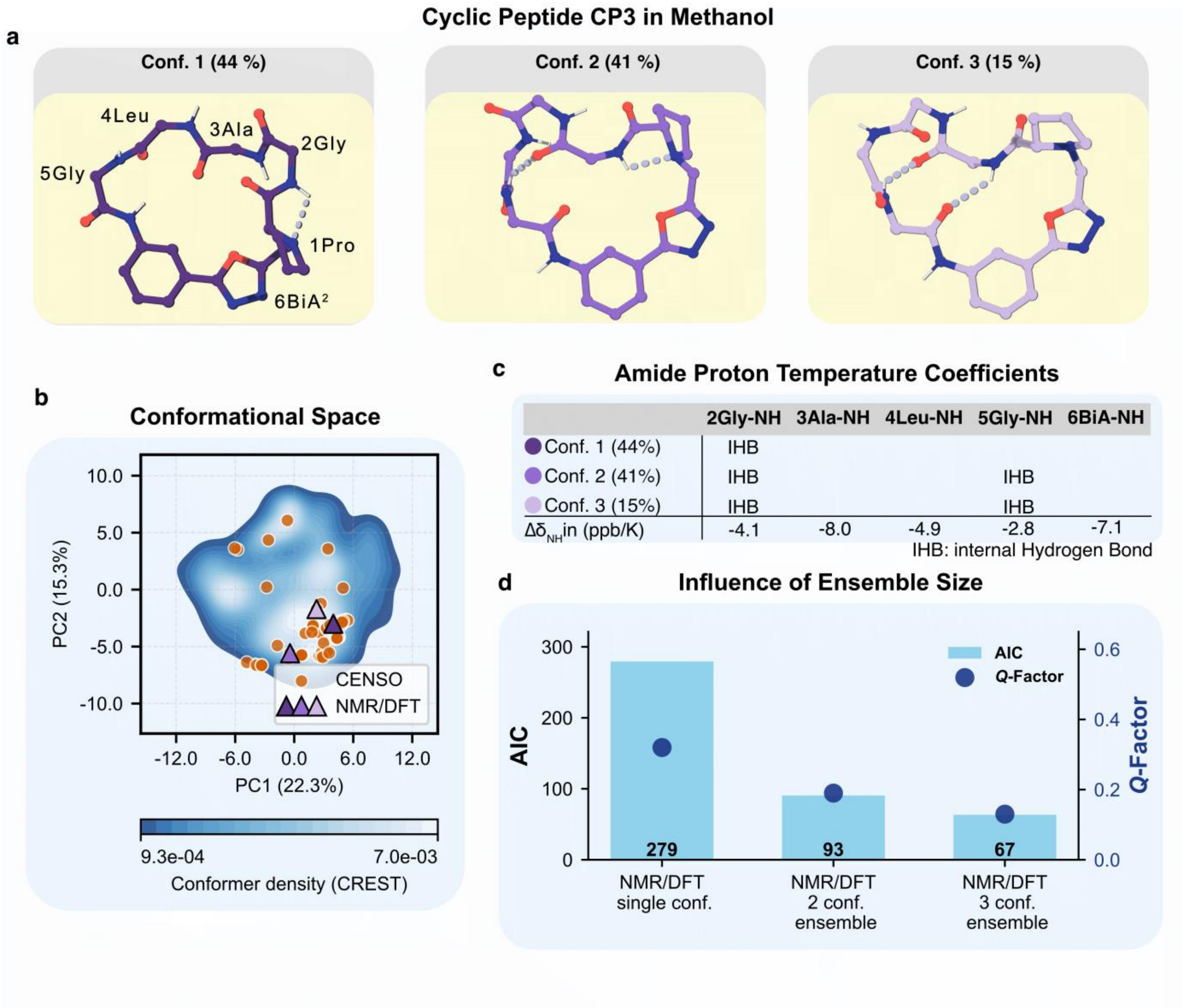

Figure S28. Stereofitter ensemble obtained for **CP3** in Methanol. **a** Structures of the three conformers with indicated internal hydrogen bonds and population in the Stereofitter ensemble. **b** Conformational space of **CP3** in methanol spanned by the CREST sampling, CENSO refinement and the selected conformers. **c** Amide proton temperature coefficient measured together with indications which amide proton of the determined conformer is likely to be involved in internal hydrogen bonds. **d** Statistical data from Stereofitter when increasing the ensemble size.

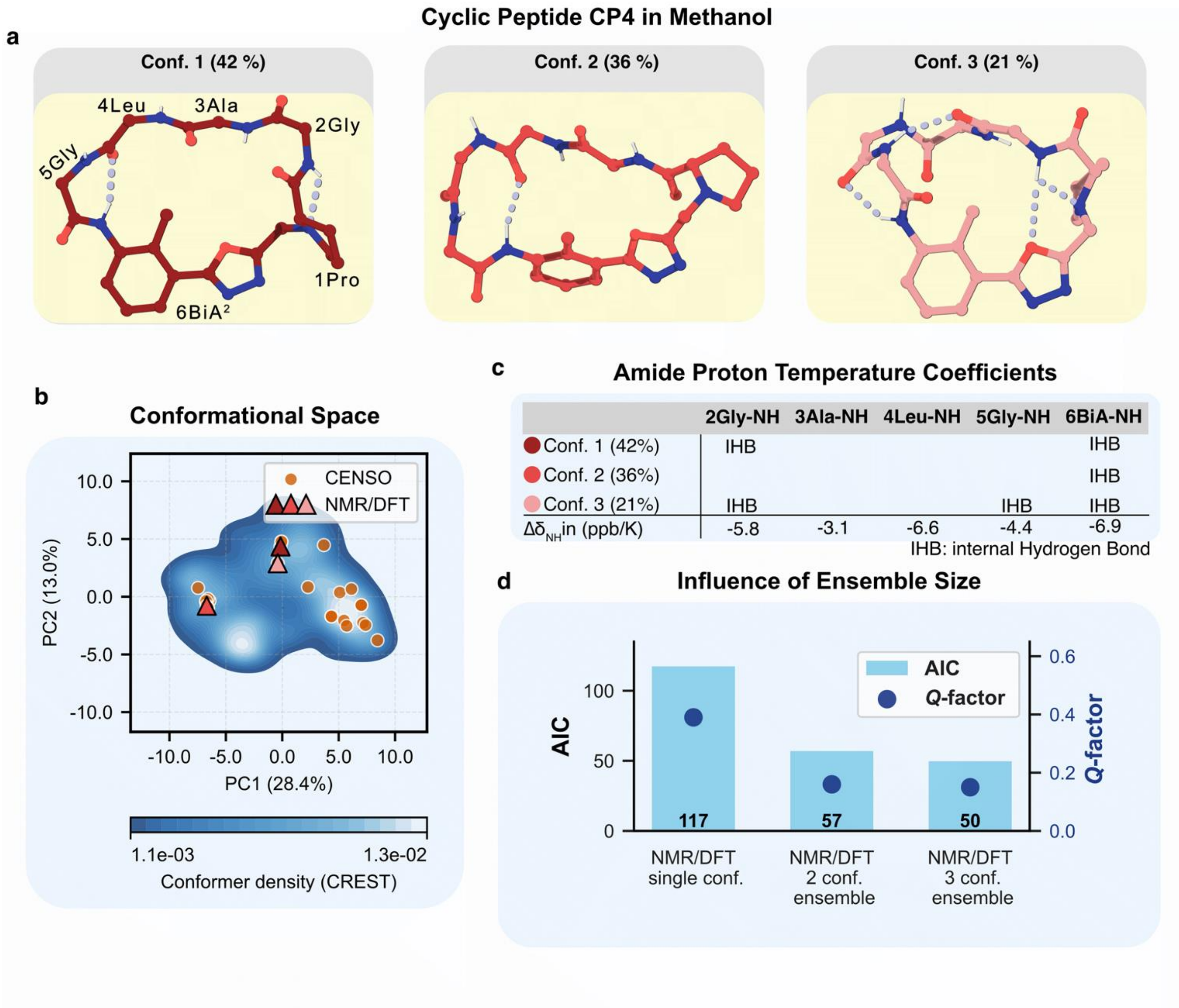

Figure S29. Stereofitter ensemble obtained for **CP4** in Methanol. **a** Structures of the three conformers with indicated internal hydrogen bonds and population in the Stereofitter ensemble. **b** Conformational space of **CP4** in methanol spanned by the CREST sampling, CENSO refinement and the selected conformers. **c** Amide proton temperature coefficient measured together with indications which amide proton of the determined conformer is likely to be involved in internal hydrogen bonds. **d** Statistical data from Stereofitter when increasing the ensemble size.

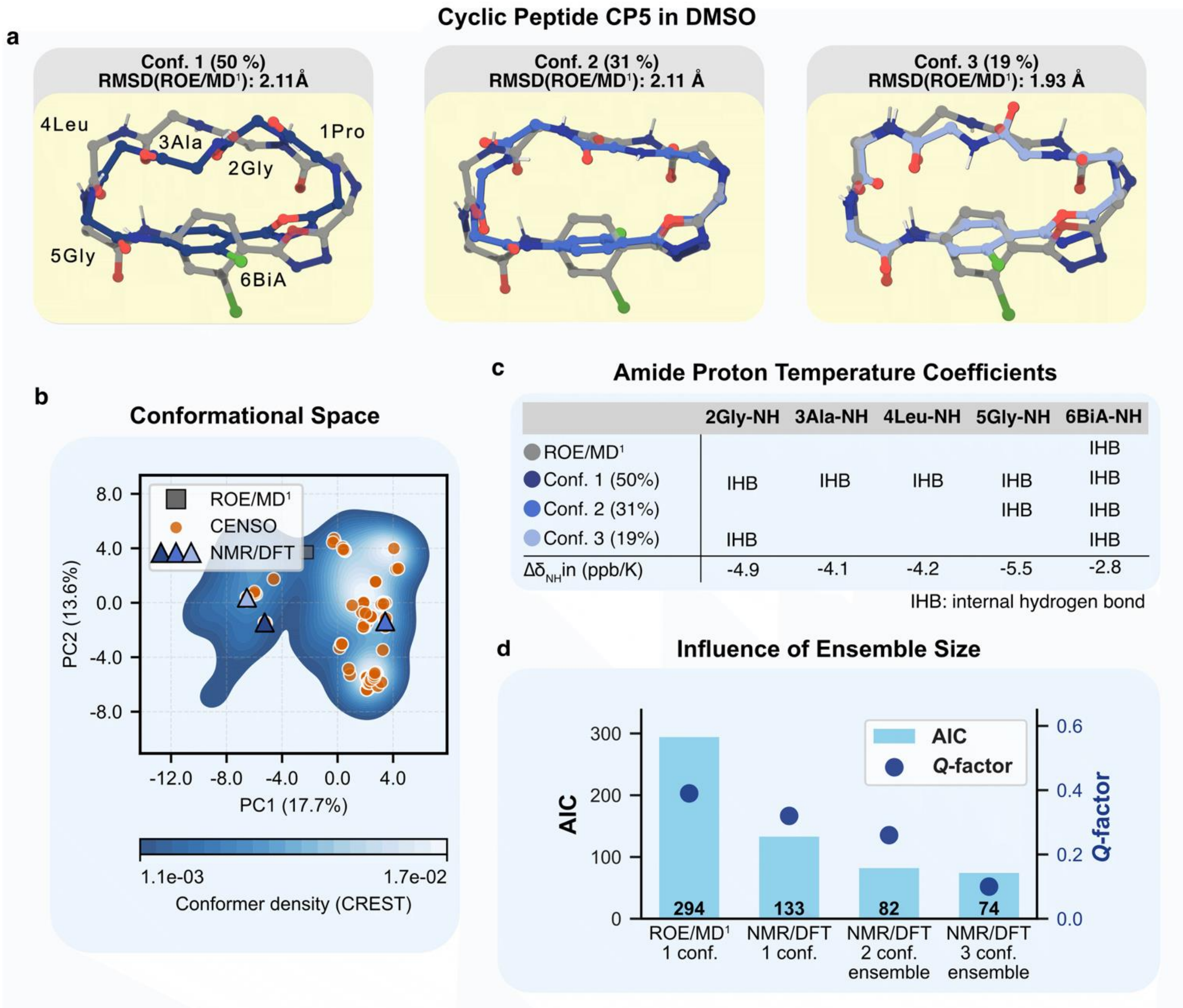

Figure S30. Stereofitter ensemble obtained for **CP5** in DMSO. **a** Structures of the three conformers and population in the Stereofitter ensemble overlayed with ROE/MD[1] conformer. **b** Conformational space of **CP5** in DMSO spanned by the CREST sampling, CENSO refinement and the selected conformers. **c** Amide proton temperature coefficient measured together with indications which amide proton of the determined conformer is likely to be involved in internal hydrogen bonds. **d** Statistical data from Stereofitter when increasing the ensemble size.

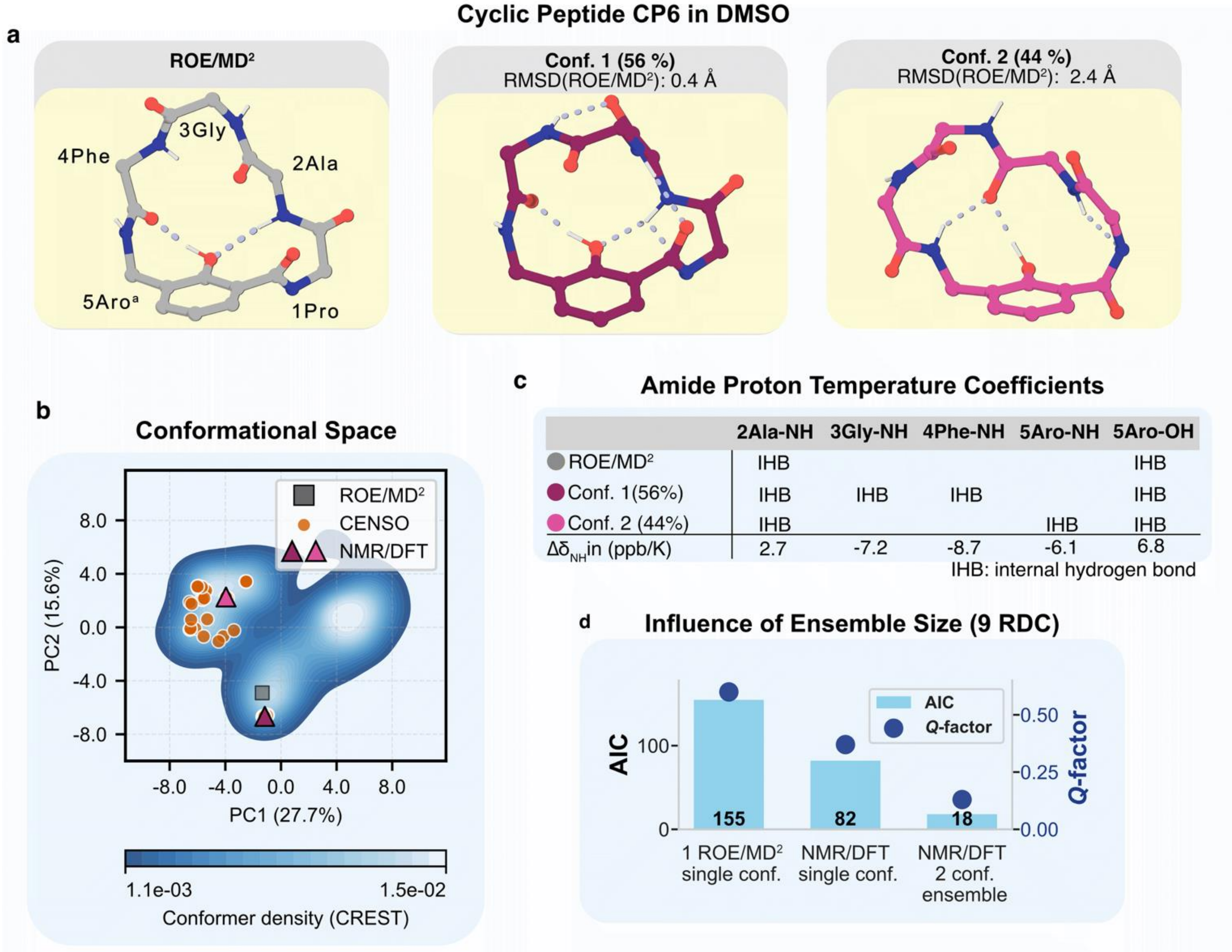

	2Ala-NH	3Gly-NH	4Phe-NH	5Aro-NH	5Aro-OH
ROE/MD²	IHB				IHB
Conf. 1(56%)	IHB	IHB	IHB		IHB
Conf. 2 (44%)	IHB			IHB	IHB
$\Delta\delta_{NH}$ in (ppb/K)	2.7	-7.2	-8.7	-6.1	6.8

IHB: internal hydrogen bond

Figure S31. Stereofitter ensemble obtained for **CP6** in DMSO. **a** Structures of the ROE/MD^2 conformer and the two conformers population in the Stereofitter ensemble. **b** Conformational space of **CP6** in DMSO spanned by the CREST sampling, CENSO refinement and the selected conformers. **c** Amide proton temperature coefficient measured together with indications which amide proton of the determined conformer is likely to be involved in internal hydrogen bonds. **d** Statistical data from Stereofitter when increasing the ensemble size.

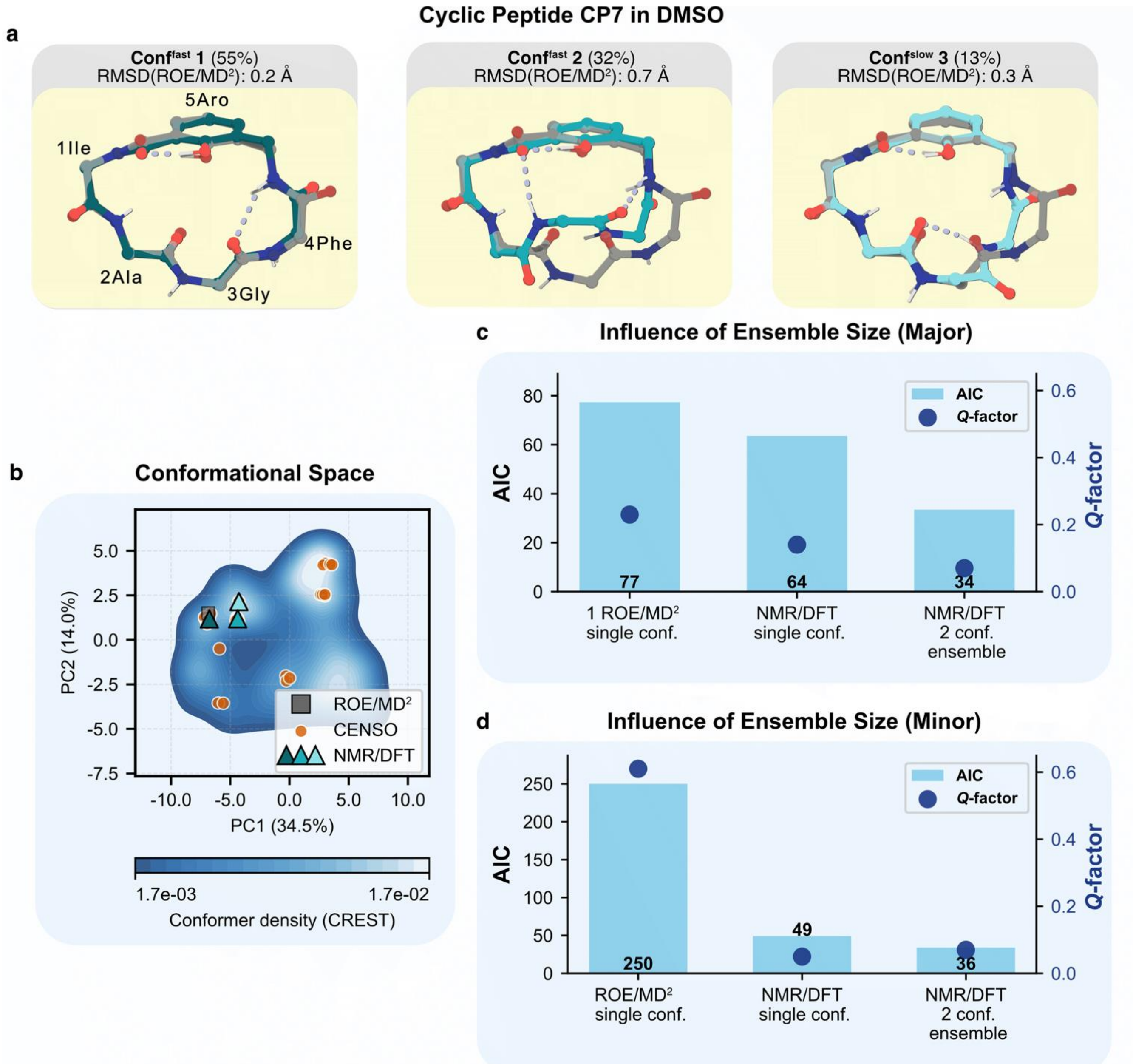

e Amide Proton Temperature Coefficients

Major

	1Ile-NH	2Ala-NH	3Gly-NH	4Phe-NH	5Aro-NH	5Aro-OH
ROE/MD^2						IHB
Conf^{fast} 1 (63%)					IHB	IHB
Conf^{fast} 2 (37%)			IHB		IHB	IHB
$\Delta\delta_{NH}$ in (ppb/K)	-3.6	-0.5	–	-4.7	–	-2.3

Minor

	1Ile-NH	2Ala-NH	3Gly-NH	4Phe-NH	5Aro-NH	5Aro-OH
ROE/MD^2						IHB
Conf^{slow} 3				IHB		IHB
$\Delta\delta_{NH}$ in (ppb/K)	-3.4	-6.3	-2.7	-3.5	-0.2	-4.6

IHB: internal hydrogen bond

Figure S32. Stereofitter ensemble obtained for **CP7** in DMSO. **a** Structures of the three conformers and population in the Stereofitter ensemble overlayed over the ROE/MD^2 conformer. **b** Conformational space of **CP7** in DMSO spanned by the CREST sampling, CENSO refinement and the selected conformers. **c** Statistical data of the Stereofitter ensemble for the major peak set. **d** Statistical data of the Stereofitter ensemble for the minor peak set. **e** Amide proton temperature coefficient measured together with indications which amide proton of the determined conformer is likely to be involved in internal hydrogen bonds.

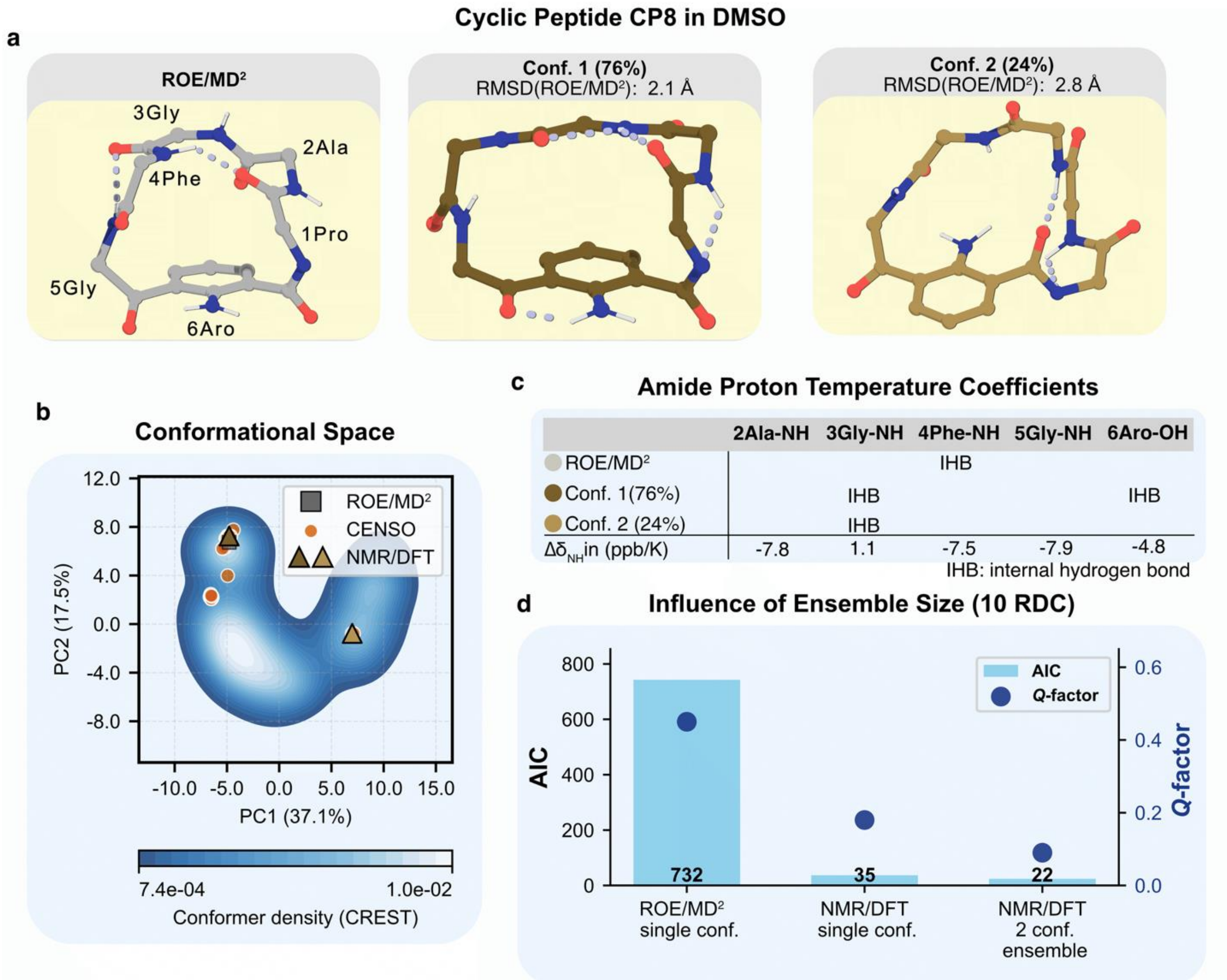

Figure S33. Stereofitter ensemble obtained for **CP8** in DMSO. **a** Structures of the ROE/MD^2 conformer and the two Stereofitter conformers with indicated internal hydrogen bonds and population in the Stereofitter ensemble. **b** Conformational space of **CP8** in DMSO spanned by the CREST sampling, CENSO refinement and the selected conformers. **c** Amide proton temperature coefficient measured together with indications which amide proton of the determined conformer is likely to be involved in internal hydrogen bonds. **d** Statistical data from Stereofitter when increasing the ensemble size.

SI.12 Computational details for conformational sampling

SI.12.1 General procedure of conformational sampling

For every cyclic peptide **CP1–CP8** the structure has been drawn in MAESTRO v. 2023 by Schrödinger[7] and converted to an initial 3D conformer. The resulting conformers have been used as the starting structures for conformational sampling using the CREST software version 2.12[8,9] in conjunction with xTB version 6.6.1[10,11]. Each time the GFN2-xTB[11] tight binding method has been used with ALPB[12] solvation in methanol or DMSO respectively. Ensembles of conformers in an energy window of 30 kcal/mol above the lowest energy conformer were obtained.

The conformers in an energy window of 6 kcal/mol have been used for further ensemble refinement using the CENSO software version 1.2[13] together with either ORCA 5.0.4 or ORCA 6.0.0[14–17]. The CENSO approach includes a multistep procedure using computationally more demanding methods in each consecutive step. Overall, the initial ensembles were filtered down in each case to an DFT optimized ensemble within a chosen energy window. The initial ensemble was filtered first by B3LYP[18]/def2-SV(P)[19]/ALPB(solvent) using an energy window of 6 kcal/mol. Afterwards, a 5 kcal/mol energy window was used for filtering based on free energies calculated at B3LYP-*D4*[20]/def2-TZVP[19](-f)/ALPB(solvent) with vibrational energy correction using the GFN2-xTB/ALPB method. All conformers in a 4 kcal/mol energy window were used in a DFT geometry optimization at the B3LYP-*D4*/def2-TZVP(-f)/SMD[21](solvent) level of theory, using again GFN2-xTB/ALPB for the vibrational corrections. See **Table S28** for the number of conformers obtained in each step. All conformers for which the geometry optimization was successful were used in the further analysis of the experimental NMR data in Stereofitter. It should be noted that for **CP6–CP8** the DFT functional PW6B95[22]-*D4* has been used in CENSO instead of B3LYP-*D4*, it is assumed that this does not have a large impact on the refined ensemble. Ensembles sizes after the different steps are shown in **Table S28** and DFT calculated free energies are shown in **Table S29**.

However, before the conformers were used for the conformational selection using the NMR data, all conformers were reoptimized in ORCA 5.0.4 or 6.0.0 at the PW6B95-*D4*/def2-TZVP/SMD level of theory using the "TIGHTOPT" option using the standard grid "DEFGRID2". "DEFGRID3" integration grids were used for structures with convergence issues. The resulting geometries have been used in GIAO-DFT calculation to calculate the isotropic chemical shielding constants as well as spin-spin coupling constant. For these calculations the r2SCAN0[23]/pcSseg-2[24]/SMD method has been used with the "VeryTightSCF" and "DEFGRID3" keywords and the DOBSON method[25] has been employed for gauge correction in the kinetic energy density τ of r2SCAN0. All GIAO-DFT calculations have been done in ORCA 6.0.0 The obtained ORCA output files were used then used directly as input files for *Stereofitter*.

Table S28. Conformational ensembles for cyclic peptides, after each sampling/refinement step.

	**CP1**	**CP2**	**CP3**	**CP4**	**CP5**	**CP6**[a]	**CP7**	**CP8**
Solvent	DMSO	Methanol	Methanol	Methanol	DMSO	DMSO	DMSO	DMSO
CREST (30 kcal/mol)	5539	11334	3553	3741	2284	448/670	1877	441
CREST (6 kcal/mol)	865	1561	395	576	559	375/22	297	441
Ensemble after CENSO	483/93[b]	103	63	26	117	30/21	34	32

[a] Two sampling starting from the two initial structures, where performed, one with the proline in cis- and the other in trans-conformation.
[b] A smaller set of 92 conformer was chosen, still representing 95% of the overall Boltzmann population.

Table S29. Gibbs free energy for the conformers of the cyclic peptides **CP1–CP5** at the B3LYP-*D4*/def-TZVP(-f)/SMD(solvent) and **CP6–CP8** PW6B95-*D4*/def2-TZVP/SMD(solvent)level of theory with zero-point vibration correction calculated at the GFN2-xTB level of theory.

Cyclic Peptide and Solvent	CONF Index	CONF Name	G_{Conf} in (kcal/mol)	ΔG_{Conf} in (kcal/mol)	Boltzmann weight at 298.15 K
**CP1 in DMSO**	1	CONF10	-2268.8794	3.2	0.1
	2	CONF11	-2268.8791	3.4	0.1
	3	CONF15	-2268.8794	3.2	0.1
	4	CONF36	-2268.8792	3.3	0.1
	5	CONF39	-2268.8799	2.9	0.2
	6	CONF40	-2268.88	2.9	0.2
	7	CONF43	-2268.8827	1.2	3.5
	8	CONF44	-2268.8825	1.3	3
	9	CONF45	-2268.8825	1.3	2.8
	10	CONF46	-2268.8827	1.1	3.6
	11	CONF47	-2268.8826	1.2	3.1
	12	CONF49	-2268.8829	1	4.6
	13	CONF56	-2268.881	2.2	0.6
	14	CONF57	-2268.8805	2.6	0.3
	15	CONF58	-2268.881	2.2	0.6
	16	CONF59	-2268.8805	2.5	0.4
	17	CONF60	-2268.8806	2.5	0.4
	18	CONF67	-2268.882	1.6	1.7
	19	CONF69	-2268.8802	2.7	0.2
	20	CONF73	-2268.8793	3.3	0.1
	21	CONF74	-2268.8795	3.2	0.1
	22	CONF79	-2268.8796	3.1	0.1
	23	CONF80	-2268.8811	2.1	0.7
	24	CONF81	-2268.8797	3.1	0.1
	25	CONF82	-2268.8812	2.1	0.7
	26	CONF86	-2268.8835	0.7	8.1
	27	CONF119	-2268.8797	3	0.2
	28	CONF131	-2268.8818	1.7	1.3
	29	CONF134	-2268.8815	1.9	1
	30	CONF140	-2268.8814	2	0.9
	31	CONF142	-2268.8797	3	0.1
	32	CONF147	-2268.8797	3	0.1
	33	CONF162	-2268.879	3.4	0.1
	34	CONF168	-2268.8794	3.2	0.1
	35	CONF171	-2268.8792	3.4	0.1
	36	CONF172	-2268.8791	3.4	0.1
	37	CONF173	-2268.879	3.4	0.1
	38	CONF177	-2268.8791	3.4	0.1

39	CONF178	-2268.8792	3.3	0.1
40	CONF179	-2268.8792	3.3	0.1
41	CONF187	-2268.8803	2.7	0.3
42	CONF188	-2268.8804	2.6	0.3
43	CONF189	-2268.8803	2.6	0.3
44	CONF191	-2268.8803	2.6	0.3
45	CONF194	-2268.8812	2.1	0.8
46	CONF195	-2268.8809	2.3	0.5
47	CONF197	-2268.8809	2.3	0.5
48	CONF203	-2268.881	2.2	0.6
49	CONF205	-2268.8809	2.3	0.5
50	CONF206	-2268.8811	2.2	0.6
51	CONF207	-2268.8811	2.1	0.7
52	CONF208	-2268.881	2.2	0.6
53	CONF210	-2268.881	2.2	0.6
54	CONF211	-2268.8816	1.8	1.2
55	CONF216	-2268.8792	3.3	0.1
56	CONF219	-2268.8811	2.1	0.7
57	CONF226	-2268.88	2.8	0.2
58	CONF227	-2268.8809	2.3	0.5
59	CONF232	-2268.8805	2.5	0.3
60	CONF236	-2268.8793	3.3	0.1
61	CONF237	-2268.8799	2.9	0.2
62	CONF250	-2268.881	2.2	0.6
63	CONF254	-2268.8821	1.5	1.9
64	CONF260	-2268.8828	1.1	3.9
65	CONF261	-2268.8825	1.3	2.9
66	CONF266	-2268.8824	1.3	2.5
67	CONF270	-2268.8816	1.8	1.2
68	CONF272	-2268.8806	2.5	0.4
69	CONF295	-2268.8792	3.3	0.1
70	CONF304	-2268.8791	3.4	0.1
71	CONF307	-2268.8791	3.4	0.1
72	CONF313	-2268.8791	3.4	0.1
73	CONF315	-2268.8791	3.4	0.1
74	CONF323	-2268.8791	3.4	0.1
75	CONF324	-2268.8794	3.2	0.1
76	CONF325	-2268.8794	3.2	0.1
77	CONF337	-2268.8792	3.4	0.1
78	CONF350	-2268.8799	2.9	0.2
79	CONF356	-2268.8801	2.8	0.2
80	CONF358	-2268.88	2.8	0.2
81	CONF382	-2268.8799	2.9	0.2

	82	CONF385	-2268.88	2.9	0.2
	83	CONF391	-2268.8806	2.5	0.4
	84	CONF396	-2268.8798	3	0.2
	85	CONF454	-2268.8797	3.1	0.1
	86	CONF499	-2268.8804	2.6	0.3
	87	CONF500	-2268.8795	3.2	0.1
	88	CONF596	-2268.8835	0.6	8.4
	89	CONF612	-2268.8791	3.4	0.1
	90	CONF693	-2268.8791	3.4	0.1
	91	CONF742	-2268.8794	3.2	0.1
	92	CONF743	-2268.88	2.8	0.2
	93	CONF859	-2268.8845	0	24.8
**CP2 in methanol**	1	CONF18	-2611.2526	2	1.3
	2	CONF25	-2611.2497	3.8	0.1
	3	CONF27	-2611.2491	4.1	0
	4	CONF28	-2611.2495	3.9	0.1
	5	CONF29	-2611.2488	4.3	0
	6	CONF30	-2611.2487	4.4	0
	7	CONF34	-2611.2535	1.4	3.3
	8	CONF35	-2611.2503	3.4	0.1
	9	CONF36	-2611.2506	3.2	0.2
	10	CONF40	-2611.2508	3.1	0.2
	11	CONF41	-2611.2497	3.8	0.1
	12	CONF46	-2611.2494	4	0
	13	CONF49	-2611.2503	3.4	0.1
	14	CONF50	-2611.2496	3.8	0.1
	15	CONF51	-2611.2469	5.6	0
	16	CONF53	-2611.2507	3.1	0.2
	17	CONF54	-2611.2508	3.1	0.2
	18	CONF57	-2611.2523	2.2	1
	19	CONF58	-2611.2506	3.2	0.2
	20	CONF62	-2611.2518	2.5	0.6
	21	CONF64	-2611.2486	4.5	0
	22	CONF65	-2611.2525	2	1.2
	23	CONF68	-2611.248	4.8	0
	24	CONF69	-2611.247	5.5	0
	25	CONF74	-2611.2466	5.7	0
	26	CONF75	-2611.2481	4.8	0
	27	CONF80	-2611.254	1.1	5.8
	28	CONF81	-2611.2516	2.6	0.5
	29	CONF82	-2611.2516	2.6	0.4
	30	CONF85	-2611.2523	2.2	0.9
	31	CONF89	-2611.2495	3.9	0.1

32	CONF92	-2611.2526	2	1.3
33	CONF95	-2611.2505	3.3	0.1
34	CONF96	-2611.2551	0.4	18.9
35	CONF110	-2611.2534	1.5	2.9
36	CONF113	-2611.2533	1.5	2.8
37	CONF114	-2611.252	2.3	0.7
38	CONF122	-2611.2466	5.7	0
39	CONF126	-2611.2459	6.2	0
40	CONF129	-2611.2471	5.4	0
41	CONF136	-2611.2461	6	0
42	CONF144	-2611.2497	3.8	0.1
43	CONF145	-2611.2476	5.1	0
44	CONF147	-2611.2474	5.2	0
45	CONF148	-2611.248	4.9	0
46	CONF150	-2611.2477	5	0
47	CONF151	-2611.248	4.8	0
48	CONF153	-2611.248	4.9	0
49	CONF154	-2611.2497	3.8	0.1
50	CONF156	-2611.2501	3.5	0.1
51	CONF158	-2611.2506	3.2	0.2
52	CONF161	-2611.2517	2.5	0.5
53	CONF167	-2611.247	5.5	0
54	CONF169	-2611.2471	5.4	0
55	CONF174	-2611.2489	4.3	0
56	CONF175	-2611.2488	4.3	0
57	CONF176	-2611.2471	5.4	0
58	CONF183	-2611.2513	2.8	0.3
59	CONF193	-2611.252	2.3	0.7
60	CONF196	-2611.2451	6.7	0
61	CONF207	-2611.2474	5.2	0
62	CONF227	-2611.2488	4.4	0
63	CONF259	-2611.2462	6	0
64	CONF268	-2611.25	3.6	0.1
65	CONF269	-2611.2517	2.5	0.5
66	CONF274	-2611.2469	5.6	0
67	CONF281	-2611.2502	3.4	0.1
68	CONF286	-2611.2495	3.9	0.1
69	CONF288	-2611.2457	6.3	0
70	CONF353	-2611.2512	2.9	0.3
71	CONF421	-2611.2489	4.3	0
72	CONF451	-2611.2505	3.3	0.1
73	CONF471	-2611.2501	3.6	0.1
74	CONF526	-2611.2502	3.5	0.1

	75	CONF543	-2611.25	3.6	0.1
	76	CONF544	-2611.2499	3.7	0.1
	77	CONF575	-2611.2482	4.7	0
	78	CONF576	-2611.2481	4.8	0
	79	CONF601	-2611.2494	4	0
	80	CONF615	-2611.2494	4	0
	81	CONF619	-2611.2513	2.8	0.3
	82	CONF631	-2611.247	5.5	0
	83	CONF661	-2611.2487	4.4	0
	84	CONF673	-2611.2483	4.7	0
	85	CONF714	-2611.2447	6.9	0
	86	CONF717	-2611.2485	4.6	0
	87	CONF722	-2611.248	4.9	0
	88	CONF761	-2611.2466	5.7	0
	89	CONF841	-2611.2474	5.2	0
	90	CONF922	-2611.2474	5.2	0
	91	CONF923	-2611.2469	5.6	0
	92	CONF957	-2611.2488	4.4	0
	93	CONF977	-2611.2523	2.2	0.9
	94	CONF1007	-2611.2471	5.4	0
	95	CONF1025	-2611.2473	5.3	0
	96	CONF1028	-2611.2472	5.3	0
	97	CONF1066	-2611.2541	1	6.5
	98	CONF1089	-2611.2543	0.9	7.7
	99	CONF1260	-2611.2529	1.8	1.7
	100	CONF1363	-2611.2506	3.2	0.2
	101	CONF1375	-2611.248	4.8	0
	102	CONF1461	-2611.2557	0	35.6
	103	CONF1486	-2611.2454	6.5	0
**CP3 in methanol**	1	CONF35	-2017.8269	0.6	5.7
	2	CONF37	-2017.8269	0.6	5.4
	3	CONF44	-2017.825	1.8	0.7
	4	CONF49	-2017.8248	1.9	0.6
	5	CONF66	-2017.8261	1.1	2.4
	6	CONF70	-2017.8256	1.4	1.4
	7	CONF77	-2017.8277	0.1	13.3
	8	CONF80	-2017.8275	0.2	10.7
	9	CONF85	-2017.8226	3.3	0.1
	10	CONF104	-2017.8207	4.5	0
	11	CONF105	-2017.8212	4.2	0
	12	CONF110	-2017.8209	4.4	0
	13	CONF112	-2017.8242	2.3	0.3
	14	CONF121	-2017.8224	3.4	0.1

15	CONF137	-2017.8253	1.6	1
16	CONF138	-2017.8255	1.5	1.2
17	CONF149	-2017.82	4.9	0
18	CONF150	-2017.82	4.9	0
19	CONF152	-2017.8203	4.7	0
20	CONF155	-2017.824	2.4	0.3
21	CONF156	-2017.8202	4.8	0
22	CONF157	-2017.8228	3.1	0.1
23	CONF160	-2017.8201	4.8	0
24	CONF166	-2017.8227	3.2	0.1
25	CONF167	-2017.8244	2.1	0.4
26	CONF173	-2017.8213	4.1	0
27	CONF174	-2017.8213	4.1	0
28	CONF178	-2017.8207	4.5	0
29	CONF186	-2017.8213	4.1	0
30	CONF187	-2017.8207	4.5	0
31	CONF199	-2017.8257	1.3	1.5
32	CONF204	-2017.8254	1.5	1.1
33	CONF209	-2017.8253	1.6	1
34	CONF239	-2017.8232	2.9	0.1
35	CONF245	-2017.8226	3.3	0.1
36	CONF248	-2017.8201	4.8	0
37	CONF263	-2017.8213	4.1	0
38	CONF265	-2017.8222	3.6	0
39	CONF266	-2017.8199	5	0
40	CONF267	-2017.8235	2.7	0.1
41	CONF269	-2017.8234	2.8	0.1
42	CONF275	-2017.8233	2.8	0.1
43	CONF276	-2017.8248	1.9	0.6
44	CONF279	-2017.821	4.3	0
45	CONF280	-2017.8211	4.3	0
46	CONF283	-2017.8198	5.1	0
47	CONF284	-2017.8198	5	0
48	CONF289	-2017.8221	3.6	0
49	CONF291	-2017.8208	4.4	0
50	CONF293	-2017.8227	3.2	0.1
51	CONF295	-2017.823	3	0.1
52	CONF301	-2017.8278	0	14.6
53	CONF302	-2017.8249	1.8	0.7
54	CONF308	-2017.8238	2.5	0.2
55	CONF326	-2017.824	2.4	0.2
56	CONF334	-2017.8189	5.6	0
57	CONF335	-2017.8218	3.8	0

	58	CONF363	-2017.8226	3.3	0.1
	59	CONF366	-2017.8191	5.5	0
	60	CONF369	-2017.8272	0.4	7.7
	61	CONF370	-2017.8273	0.3	8.5
	62	CONF371	-2017.8275	0.2	10.9
	63	CONF374	-2017.8273	0.3	8.4
**CP4 in metha-nol**	1	CONF31	-2057.1007	2.5	0.6
	2	CONF76	-2057.1048	0	43.9
	3	CONF77	-2057.1039	0.5	17.9
	4	CONF78	-2057.0975	4.6	0
	5	CONF83	-2057.1026	1.4	4.4
	6	CONF87	-2057.1027	1.3	4.7
	7	CONF92	-2057.103	1.1	6.8
	8	CONF148	-2057.0991	3.6	0.1
	9	CONF149	-2057.1017	2	1.6
	10	CONF151	-2057.102	1.7	2.3
	11	CONF152	-2057.102	1.7	2.4
	12	CONF155	-2057.0981	4.2	0
	13	CONF180	-2057.0997	3.2	0.2
	14	CONF224	-2057.0966	5.1	0
	15	CONF228	-2057.0963	5.3	0
	16	CONF239	-2057.1022	1.6	2.8
	17	CONF242	-2057.0987	3.8	0.1
	18	CONF243	-2057.0993	3.4	0.1
	19	CONF258	-2057.0964	5.3	0
	20	CONF285	-2057.0998	3.1	0.2
	21	CONF305	-2057.0997	3.2	0.2
	22	CONF330	-2057.0984	4	0.1
	23	CONF333	-2057.0985	3.9	0.1
	24	CONF447	-2057.1019	1.8	2.1
	25	CONF454	-2057.1031	1.1	7.4
	26	CONF562	-2057.1018	1.9	1.9
**CP5 in DMSO**	1	CONF2	-2477.3762	3.9	0
	2	CONF3	-2477.3765	3.7	0.1
	3	CONF4	-2477.3741	5.2	0
	4	CONF5	-2477.373	5.9	0
	5	CONF6	-2477.3727	6.1	0
	6	CONF8	-2477.3759	4.1	0
	7	CONF9	-2477.3807	1	4.4
	8	CONF10	-2477.3814	0.6	8.7
	9	CONF13	-2477.3824	0	25.6
	10	CONF14	-2477.3811	0.8	6.9
	11	CONF15	-2477.382	0.3	16.4

12	CONF16	-2477.381	0.9	6.1
13	CONF17	-2477.3786	2.4	0.5
14	CONF22	-2477.3803	1.3	2.8
15	CONF26	-2477.3716	6.8	0
16	CONF29	-2477.3765	3.7	0.1
17	CONF30	-2477.3779	2.8	0.2
18	CONF31	-2477.3783	2.5	0.4
19	CONF32	-2477.3793	2	0.9
20	CONF34	-2477.3766	3.6	0.1
21	CONF40	-2477.3774	3.1	0.1
22	CONF41	-2477.3778	2.9	0.2
23	CONF43	-2477.3774	3.2	0.1
24	CONF46	-2477.374	5.3	0
25	CONF49	-2477.38	1.5	2.1
26	CONF50	-2477.3768	3.5	0.1
27	CONF51	-2477.3758	4.1	0
28	CONF52	-2477.3759	4.1	0
29	CONF53	-2477.3761	3.9	0
30	CONF54	-2477.3761	3.9	0
31	CONF56	-2477.3781	2.7	0.3
32	CONF59	-2477.3728	6	0
33	CONF60	-2477.3729	6	0
34	CONF62	-2477.372	6.5	0
35	CONF63	-2477.3789	2.2	0.7
36	CONF64	-2477.3718	6.6	0
37	CONF65	-2477.3784	2.5	0.4
38	CONF66	-2477.3784	2.5	0.4
39	CONF68	-2477.3724	6.3	0
40	CONF69	-2477.3737	5.5	0
41	CONF70	-2477.3735	5.6	0
42	CONF71	-2477.3736	5.5	0
43	CONF72	-2477.3763	3.8	0
44	CONF74	-2477.3731	5.8	0
45	CONF75	-2477.3753	4.4	0
46	CONF79	-2477.3752	4.5	0
47	CONF80	-2477.376	4	0
48	CONF83	-2477.3717	6.7	0
49	CONF86	-2477.375	4.6	0
50	CONF89	-2477.3751	4.6	0
51	CONF90	-2477.3794	1.9	1.1
52	CONF93	-2477.3714	6.9	0
53	CONF99	-2477.3722	6.4	0
54	CONF100	-2477.3723	6.4	0

55	CONF101	-2477.3772	3.3	0.1
56	CONF109	-2477.3773	3.2	0.1
57	CONF129	-2477.3707	7.3	0
58	CONF133	-2477.3794	1.9	1.1
59	CONF141	-2477.3715	6.9	0
60	CONF142	-2477.3713	7	0
61	CONF149	-2477.3766	3.6	0.1
62	CONF154	-2477.3789	2.2	0.6
63	CONF156	-2477.3788	2.3	0.6
64	CONF165	-2477.3783	2.6	0.3
65	CONF166	-2477.3776	3	0.2
66	CONF168	-2477.3777	2.9	0.2
67	CONF171	-2477.3786	2.4	0.5
68	CONF180	-2477.3727	6.1	0
69	CONF182	-2477.3771	3.3	0.1
70	CONF187	-2477.3771	3.3	0.1
71	CONF195	-2477.3734	5.6	0
72	CONF200	-2477.3728	6	0
73	CONF201	-2477.3729	6	0
74	CONF202	-2477.3771	3.3	0.1
75	CONF206	-2477.3764	3.8	0.1
76	CONF220	-2477.3715	6.9	0
77	CONF236	-2477.3752	4.5	0
78	CONF238	-2477.372	6.5	0
79	CONF239	-2477.372	6.5	0
80	CONF240	-2477.372	6.5	0
81	CONF243	-2477.3703	7.6	0
82	CONF246	-2477.3754	4.4	0
83	CONF247	-2477.3761	3.9	0
84	CONF259	-2477.376	4	0
85	CONF260	-2477.3754	4.4	0
86	CONF261	-2477.3752	4.5	0
87	CONF266	-2477.3751	4.6	0
88	CONF267	-2477.3745	4.9	0
89	CONF268	-2477.3747	4.8	0
90	CONF269	-2477.3745	5	0
91	CONF273	-2477.3749	4.7	0
92	CONF274	-2477.3747	4.8	0
93	CONF275	-2477.3742	5.2	0
94	CONF276	-2477.3751	4.5	0
95	CONF296	-2477.3756	4.2	0
96	CONF299	-2477.376	4	0
97	CONF315	-2477.3756	4.2	0

	98	CONF325	-2477.3732	5.8	0
	99	CONF333	-2477.3731	5.8	0
	100	CONF359	-2477.375	4.6	0
	101	CONF373	-2477.3738	5.4	0
	102	CONF388	-2477.3741	5.2	0
	103	CONF389	-2477.3733	5.7	0
	104	CONF391	-2477.3734	5.6	0
	105	CONF406	-2477.3772	3.2	0.1
	106	CONF409	-2477.3719	6.6	0
	107	CONF411	-2477.3713	7	0
	108	CONF412	-2477.3725	6.2	0
	109	CONF413	-2477.3724	6.2	0
	110	CONF415	-2477.3789	2.2	0.7
	111	CONF422	-2477.3819	0.3	15.8
	112	CONF485	-2477.3717	6.7	0
	113	CONF489	-2477.3703	7.6	0
	114	CONF493	-2477.375	4.6	0
	115	CONF527	-2477.3772	3.3	0.1
	116	CONF533	-2477.3729	5.9	0
	117	CONF535	-2477.3729	6	0
**CP6 in DMSO**	1	CONF2	-1775.2923	4.2	0
	2	CONF26	-1775.2967	1.4	2.7
	3	CONF33	-1775.2962	1.7	1.5
	4	CONF34	-1775.2963	1.6	1.8
	5	CONF38	-1775.295	2.5	0.4
	6	CONF42	-1775.2917	4.5	0
	7	CONF51	-1775.2942	3	0.2
	8	CONF53	-1775.2947	2.7	0.3
	9	CONF58	-1775.294	3.1	0.2
	10	CONF76	-1775.296	1.9	1.2
	11	CONF81	-1775.2961	1.7	1.5
	12	CONF91	-1775.2956	2.1	0.8
	13	CONF92	-1775.2953	2.3	0.6
	14	CONF102	-1775.2933	3.5	0.1
	15	CONF109	-1775.295	2.5	0.4
	16	CONF113	-1775.2953	2.3	0.6
	17	CONF116	-1775.2954	2.2	0.7
	18	CONF153	-1775.2949	2.5	0.4
	19	CONF154	-1775.2948	2.6	0.4
	20	CONF162	-1775.2968	1.3	3.1
	21	CONF174	-1775.2969	1.2	3.4
	22	CONF185	-1775.2965	1.5	2.2
	23	CONF191	-1775.2987	0.1	23.2

	24	CONF193	-1775.2987	0.2	21.2
	25	CONF205	-1775.2962	1.7	1.6
	26	CONF232	-1775.2936	3.3	0.1
	27	CONF236	-1775.2989	0	27.9
	28	CONF244	-1775.2962	1.7	1.5
	29	CONF279	-1775.2934	3.4	0.1
	30	CONF302	-1775.2964	1.6	1.9
**CP6 in DMSO (trans proline)***	31	CONF99001	-1775.784	0.1	10
	32	CONF99002	-1775.7837	0.3	6.3
	33	CONF99003	-1775.7839	0.2	8
	34	CONF99004	-1775.7837	0.3	6.2
	35	CONF99005	-1775.7824	0.9	2.2
	36	CONF99006	-1775.7839	0.1	9.7
	37	CONF99007	-1775.7839	0.1	9.6
	38	CONF99008	-1775.7838	0.2	8
	39	CONF99009	-1775.7831	0.5	4.9
	40	CONF99010	-1775.7839	0.1	10
	41	CONF99011	-1775.7825	0.9	2.4
	42	CONF99012	-1775.7827	0.8	3
	43	CONF99013	-1775.7825	1	2
	44	CONF99014	-1775.7831	0.6	3.9
	45	CONF99015	-1775.784	0	10.9
	46	CONF99016	-1775.7811	2	0.4
	47	CONF99017	-1775.7812	1.9	0.4
	48	CONF99018	-1775.7815	1.8	0.5
	49	CONF99019	-1775.7815	1.8	0.5
	50	CONF99020	-1775.7815	1.8	0.5
	51	CONF99021	-1775.7815	1.8	0.6
**CP7 in DMSO**	1	CONF4	-1815.8437	5.5	0
	2	CONF10	-1815.8453	4.6	0
	3	CONF14	-1815.8433	5.8	0
	4	CONF16	-1815.843	6	0
	5	CONF23	-1815.8432	5.9	0
	6	CONF42	-1815.8459	4.2	0.1
	7	CONF43	-1815.846	4.1	0.1
	8	CONF46	-1815.8454	4.5	0
	9	CONF49	-1815.8456	4.4	0
	10	CONF54	-1815.8459	4.2	0.1
	11	CONF59	-1815.8463	3.9	0.1
	12	CONF60	-1815.8457	4.3	0
	13	CONF81	-1815.8525	0	61.8
	14	CONF89	-1815.8518	0.5	28
	15	CONF92	-1815.849	2.2	1.4

	16	CONF104	-1815.8477	3	0.4
	17	CONF106	-1815.8461	4.1	0.1
	18	CONF107	-1815.8477	3	0.4
	19	CONF115	-1815.847	3.5	0.2
	20	CONF120	-1815.8488	2.4	1.2
	21	CONF121	-1815.8488	2.3	1.2
	22	CONF148	-1815.8466	3.7	0.1
	23	CONF160	-1815.8467	3.7	0.1
	24	CONF166	-1815.8489	2.3	1.3
	25	CONF180	-1815.846	4.1	0.1
	26	CONF181	-1815.8462	4	0.1
	27	CONF190	-1815.849	2.2	1.4
	28	CONF197	-1815.8469	3.5	0.2
	29	CONF223	-1815.846	4.1	0.1
	30	CONF230	-1815.8468	3.6	0.2
	31	CONF241	-1815.8485	2.6	0.8
	32	CONF256	-1815.8456	4.3	0
	33	CONF266	-1815.8443	5.2	0
	34	CONF284	-1815.8483	2.7	0.7
**CP8 in DMSO**	1	CONF1	-1868.9039	1.1	5.9
	2	CONF2	-1868.9047	0.5	14.4
	3	CONF3	-1868.9016	2.5	0.6
	4	CONF4	-1868.8997	3.7	0.1
	5	CONF6	-1868.9022	2.1	1.1
	6	CONF8	-1868.9028	1.7	2
	7	CONF10	-1868.9028	1.7	2
	8	CONF11	-1868.9024	2	1.2
	9	CONF13	-1868.9012	2.7	0.4
	10	CONF14	-1868.9008	3	0.2
	11	CONF22	-1868.9034	1.3	3.7
	12	CONF23	-1868.9031	1.6	2.5
	13	CONF26	-1868.9034	1.3	3.8
	14	CONF27	-1868.9033	1.4	3.4
	15	CONF28	-1868.9055	0	35.2
	16	CONF29	-1868.9033	1.4	3.2
	17	CONF36	-1868.9026	1.8	1.6
	18	CONF37	-1868.9028	1.7	2
	19	CONF43	-1868.9024	2	1.2
	20	CONF44	-1868.9046	0.6	12.3
	21	CONF45	-1868.9024	2	1.3
	22	CONF136	-1868.8965	5.7	0
	23	CONF165	-1868.8993	3.9	0.1
	24	CONF171	-1868.9009	2.9	0.3

25	CONF181	-1868.8997	3.7	0.1
26	CONF202	-1868.8997	3.7	0.1
27	CONF236	-1868.9005	3.2	0.2
28	CONF242	-1868.9005	3.2	0.2
29	CONF246	-1868.8984	4.5	0
30	CONF250	-1868.9005	3.2	0.2
31	CONF283	-1868.9001	3.4	0.1
32	CONF326	-1868.9021	2.2	0.9

* The index count has been continued from the cis-proline conformers of **CP6** and 9900 has been added to the indexes of the trans-proline conformers of **CP6** to distinguish from the cis-proline conformers.

SI.12.2 DFT benchmark study

In order to determine an adequate DFT functional for the non-canonical cyclic peptides at hand, different DFT functionals have been benchmark for geometry optimization. For this the lowest energy conformer, according to the CREST sampling, of **CP4** at GFN2-xTB level of theory has been used as the starting structure. For the geometry optimization benchmark the following settings in ORCA 5.0.4 have been used:

Table S30. Example Input file for B3LYP for the GIAO-DFT benchmark study

```
! B3LYP
! def2-TZVP
! D4
! TightSCF
! CPCM(methanol)
! OPT
! FREQ
! RIJCOSX

* xyzFILE 0 1 inp.xyz
```

The comparison has been done by calculating the RMSD to the structure optimized at RI-MP2[26]/def2-TZVP/CPCM[27] level of theory, see **Table S31**.

Table S31. DFT benchmark study of different DFT functionals for the geometry optimization of cyclic peptide **CP4** CONF1 using implicit solvation in methanol. The functionals have been tested against RI-MP2/def2-TZVP.

Method	Level of theory [a]	RMSD to RI-MP2 reference in (Å)
M06-2X-*D3*zero	DFT (mGGA-Hyb)	0.08
MN15-*D3*BJ	DFT (mGGA-Hyb)	0.11
M06L-*D3*zero	DFT (mGGA)	0.11
M06-*D3*zero	DFT (mGGA-Hyb)	0.13

PW6B95-*D4*	DFT (mGGA-Hyb)	0.14
BP-*D4*	DFT (GGA)	0.15
r2SCAN-*D4*	DFT (mGGA)	0.15
wB97X-V	DFT (RS-Hyb)	0.16
mPW1PW-*D4*	DFT (GGA-Hyb)	0.16
wB97-*D4*	DFT (RS-Hyb)	0.16
TPSSh-*D4*	DFT (mGGA-Hyb)	0.17
B2PLYP-*D4*	DFT (Double Hyb)	0.17
B3LYP-*D4*	DFT (GGA-Hyb)	0.17
PBE0-*D4*	DFT (GGA-Hyb)	0.17
wB97M-*D4*	DFT (RS- Hyb)	0.17
wB97X-*D3*BJ	DFT (RS-Hyb)	0.18
TPSS-*D4*	DFT (mGGA)	0.18
r2scan-3c	DFT (GGA)	0.19
wB97X-*D4*	DFT (RS-Hyb)	0.19
mPW1LYP-*D4*	DFT (GGA-Hyb)	0.21
MN15L	DFT (mGGA)	0.22
GFN2-xTB	Tight Binding	0.42

[a] Classification using common abbreviations: [meta] Generalized Gradient Approximation ([m]GGA) and [Range Separated] Hybrid functional ([RS-]Hyb)

A small non-exhaustive benchmark study for the DFT shielding calculation has been conducted. For the structure for this benchmark, we reoptimized the structure used above at the RI-MP2/def2-TZVP level of theory in vacuum. Isotropic shielding constants have been calculated using ORCA 6.0.0 using the key-words in **Table S32**. As the reference values we used the shielding constant calculated at the double DSD-PBEPB86[28]/pcSseg-2 level of theory. Mean signed errors have been calculated for ^{13}C and ^{1}H shielding constants individually.

Table S32. Example Input file for B3LYP for the settings used in the GIAO-DFT benchmark study.

```
! B3LYP
! pcSseg-2
! RIJCOSX
! VeryTightSCF
! smallprint printgap noloewdin
! NoSOSCF
! DEFGRID3

%basis
    AuxJ "AutoAux"
    AuxC "AutoAux"
end

%output
    print[P_BondOrder_M] 1
    print[P_Mayer] 1
    print[P_basis] 2
end

* xyzFILE 0 1 inp.xyz

%eprnmr
    Nuclei = all H { shift }
    Nuclei = all C { shift }
    origin giao
    giao_2el giao_2el_same_as_scf
    giao_1el giao_1el_analytic
    TAU=DOBSON
end
```

Table S33. GIAO-DFT benchmark study for the calculation of NMR shielding constants using various DFT methods against DSD-PBEP86 shielding constants.

**Method**	**Level of theory[a]**	**MSE relative to DSD-PBEP86 in [ppm] of ^{13}C shielding constant**	**MSE relative to DSD-PBEP86 in [ppm] of ^{1}H shielding constant**
MN15-L	DFT(mGGA)	6.8	0.27
KT1	DFT(GGA)	-4.1	-0.16
ωB97	DFT(RS-Hyb)	-4.2	0.09
r2SCAN0	DFT(mGGA-Hyb)	-5.4	0.00
KT2	DFT(GGA)	-6.1	-0.19
KT3	DFT(GGA)	-6.2	0.03
r2SCANh	DFT(mGGA-Hyb)	-6.3	-0.03

LC_PBE	DFT(RS-Hyb)	-6.3	-0.21
TPSS0	DFT(mGGA-Hyb)	-6.3	0.17
r2SCAN	DFT(mGGA)	-6.9	-0.05
ωB97M-V	DFT(RS-Hyb)	-7.1	0.02
ωB97X	DFT(RS-Hyb)	-7.8	0.06
TPSSh	DFT(mGGA-Hyb)	-8.2	0.16
M06-L	DFT(mGGA)	-9.0	0.36
PBE0	DFT(GGA-Hyb)	-9.0	-0.07
TPSS	DFT(mGGA)	-9.5	0.15
mPW1PW	DFT(GGA-Hyb)	-9.8	-0.02
BHandH-LYP	DFT(GGA-Hyb)	-9.9	0.19
MN15	DFT(mGGA-Hyb)	-10.4	0.18
PW6B95	DFT(mGGA-Hyb)	-11.4	0.02
CAM-B3LYP	DFT(RS-Hyb)	-12.7	0.03
M06-2X	DFT(mGGA-Hyb)	-12.8	0.00
PBE	DFT(GGA)	-13.2	-0.20
BP	DFT(GGA)	-13.4	-0.15
mPWPW	DFT(GGA)	-14.0	-0.13
LSD	DFT	-14.8	-0.67
B3LYP	DFT(GGA-Hyb)	-14.8	0.07
BLYP	DFT(GGA)	-18.5	0.01
M06	DFT(mGGA-Hyb)	-20.0	0.20

[a] Classification using common abbreviations: [meta] Generalized Gradient Approximation ([m]GGA) and [Range Separated] Hybrid functional ([RS-]Hyb)

From these two benchmark studies, it was concluded that for the cyclic peptides in our study the DFT functional had only a small influence on the geometry optimization but a larger influence on the ^{1}H and ^{13}C chemical shielding calculations. For our study presented here, we chose the PW6B95-*D4*/def2-TZVP/SMD(solvent) level of theory for geometry optimization and the r2SCAN0/pcSseg-2/SMD(solvent) level of theory for the chemical shielding calculations. The first method we choose for its small RMSD to RI-MP2 here in our study and because it was reported to be particular well suited for reproducing non-covalent interaction based on the large-scale benchmark set GMTKN55[29] study. We chose r2SCAN0 for the NMR shielding calculation for its low MSE against DSD-PBEP86 for ^{13}C as well as ^{1}H, suggesting a well-balanced functional for shielding calculations of these cyclic peptides.

SI.12.3 Linear scaling parameter to convert chemical shielding to chemical shift

For the conversion of chemical shielding constants, obtained in DFT calculation, to chemical shift, which are obtained in experimental NMR measurements, linear scaling parameter have been determined, similar to our previous procedure.[30] Here, for the level of theory r2SCAN0/pcSseg-2/SMD(solvent)//PW6B95-*D4*/def2-TZVP/SMD(solvent) for methanol and DMSO. As described previously, a subset of the DELTA50 dataset[31] has been chosen, based on the availability of their experimental shifts in either methanol or DMSO.[32] The chemical shieldings are then converted to chemical shifts in *Stereofitter* internally, based on the following equation:

$$\delta_{13C} = a + b \cdot \sigma_{13C},$$

where δ is the chemical shift, σ is the chemical shielding, *a* is the slope and *b* the y-intercept of the linear equation.

Table S34. Linear scaling parameters for the conversion of shielding to shifts for the method r2SCAN0/pcSseg-2/SMD//PW6B95-*D4*/def2-TZVP/SMD.

Solvent	Nucleus	Slope (a)	y-intercept (b)	R^2
DMSO	^{1}H	-0.918	29.142	0.998
DMSO	^{13}C	-0.930	177.710	0.998
Methanol	^{1}H	-0.906	28.844	0.997
Methanol	^{13}C	-0.912	176.32	0.994

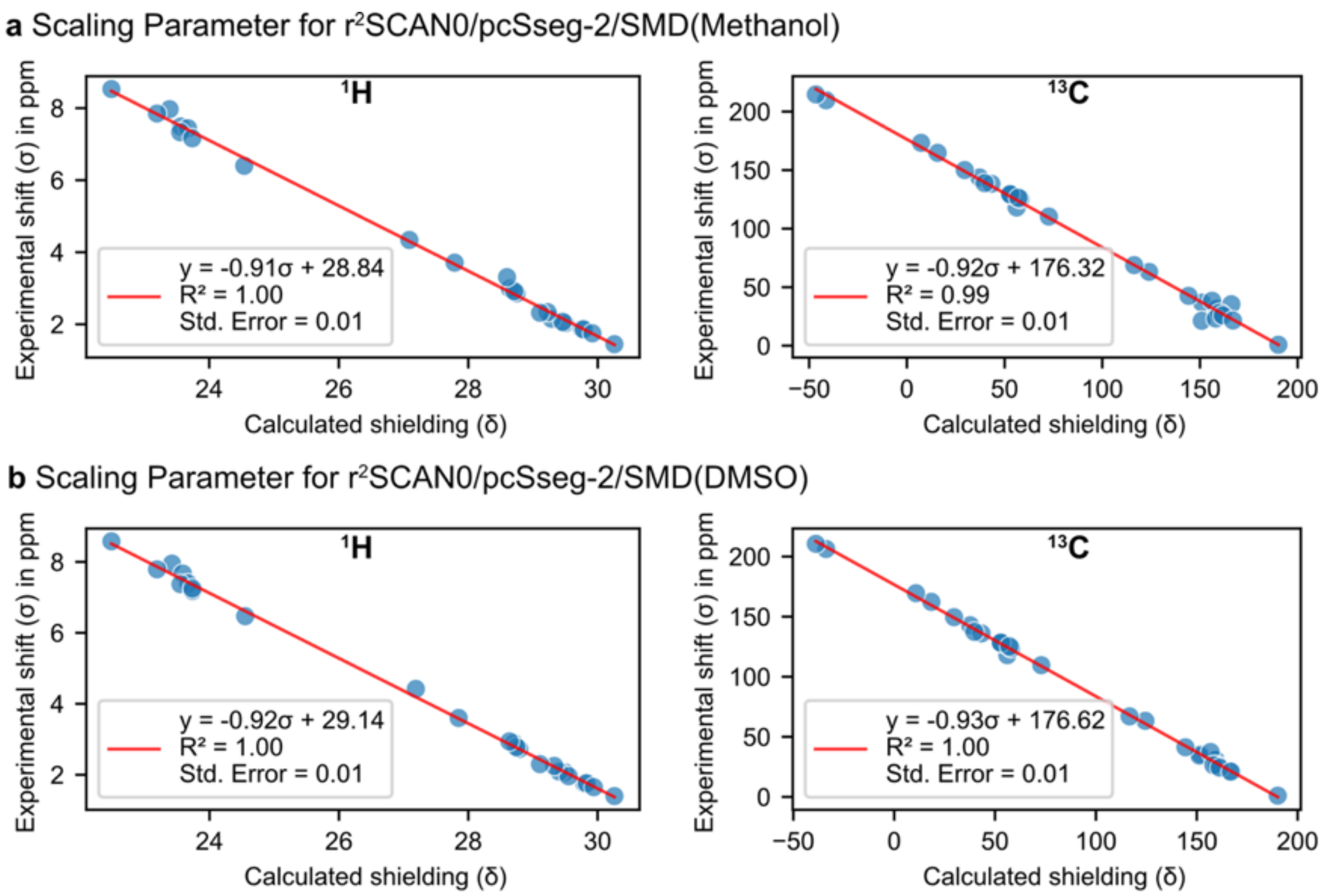

Figure S34. Determination of linear scaling parameters for the conversion of shielding to shifts for the method r2SCAN0/pcSseg-2/SMD//PW6B95-*D4*/def2-TZVP/SMD for the solvents **a** methanol and **b** DMSO .

SI.12.4 Computational times and resources

Table S35. Timings and CPU resources for each step. Most calculations were conducted on a HPC server with 40 core Intel Xenon Gold 6230 CPUs with a clock speed of 2.1 GHz.

	CP1	CP2	CP3	CP4	CP5	CP6[a]	CP7	CP8
Time of CREST	4 h / 40 cpu cores	5 h / 40 cpu cores	2.5 h / 40 cpu cores	2.75 h / 40 cpu cores	2 h / 40 cpu cores	1 h + 0.5 h / 40 cpu cores	1.25 h / 40 cpu cores	1 h / 40 cpu cores
Time for CENSO	12 days/ 40 cpu cores	10 days / 40 cpu cores	4 days / 40 cpu cores	3.5 days / 40 cpur cores	12 days/ 40 cpu cores	8 days + 2 days / 40 cpu cores	7 days / 40 cpur cores	4 days / 40 cpu cores
Example time for ORCA optimization[a]	12 hours / 4 cpu cores	27 hours / 4 cpu cores	16 hours / 4 cpu cores	3 hours / 4 cpu cores	8 hours / 4 cpu cores	19 hours / 4 cpu cores	23 hours / 4 cpu cores	29 hours / 4 cpu cores
Example time for ORCA NMR calculation[a]	14 hours / 8 cpu cores	19 hours / 8 cpu cores	11 hours / 8 cpu cores	14 hours / 8 cpu cores	12 hours / 8 cpu cores	7 hours / 8 cpu cores	9 hours / 8 cpu cores	12 hours / 8 cpu cores

[a] These calculations were done in parallel on a HPC server, however multiple different CPU models were used, therefore this can only be considered as a rough estimation. Additionally, the calculation step for the geometry optimization depends heavily on the number of steps of convergence, which vary considerably.

SI.13 Conformational analysis using Stereofitter

Conformational analysis of the cyclic peptide has been done using Stereofitter[33] 1.17 in MNOVA 15.1. For each cyclic peptide the conformers where superimposed based in their backbone atoms. Standard errors have been assumed for RDC (1.2 Hz), NOE distances (0.5 Å), ^{13}C chemical shifts (2 ppm) and *J*-couplings (0.5 Hz). *J*-couplings have been compared to predicted values based in the Karplus equation parameter published by Pardi et al.[34].

SI.13.1 Input files for Stereofitter

In the following tables the exact input files used for the conformational analysis of the **CPs 1-8** in Stereofitter are shown.

Table S36. Stereofitter Input file for cyclic peptide **CP1** in DMSO.

```
rdc_data {
6   52  -2.781
4   51  -25.2648
2   49  -6.8565
3   50  1.2524
34  77  -6.1471
31  75  2.5269
18  59  -14.3275
23  66  8.0798
44  91  -0.3477
44  92  13.2642
27  73  15.4459
27  72  5.3338}

rdc_standard_error {1.2}

jcoupling_data {
77 78 6.9 0.5 Pardi-[H-N-Ca-H]
75 76 6.5 0.5 Pardi-[H-N-Ca-H}

chemicalshift_data {
23 64.0
47 173.7
44 42.5
43 169.2
34 48.8
33 173.0
31 51.9
30 172.1
27 43.0
26 167.8
1 138.7
```

```
2 121.3
3 128.8
4 124.3
5 127.0
6 118.4
8 158.9
9 126.5
11 161.7
18 59.7}

chemicalshift_references {
6  176.480531 -0.928898}

chemicalshift_standard_error {
6 2.0}

noedistance_data {
53  49  2.57
53  52  2.43
53  75  4.12
76  77  2.43
76  75  2.85
74  75  2.41
93  59  3.01
93  (91,92) 2.6
78  77  2.98
78  92,92   2.7}

noedistance_standard_error {0.5}
aic {TRUE}
superimpose {TRUE}
superimpose_atoms {
1,7,26,27,28,30,31,32,33,34,35,43,44,46,47,23,19,18,11,8,5
}
number_of_threads {8}
max_conformations {3}
```

Table S37. Stereofitter Input file for cyclic peptide **CP2** in methanol.

```
rdc_data {
11  59  2.71
15  61  4.29
23  66  -0.85
30  74  4.76
30  73  1.69
48  92  3.87
43  85  -2.69
6   103 -6.7
4   8   -13.1
2   56  -1.2}
```

```
rdc_standard_error {1.2}

chemicalshift_data {
10  170.7
11  60.5
14  173.2
15  53.2
22  175.4
23  54.3
29  171.7
30  43.5
37  178.4
48  65.2
43  64.3
41  163.8
5   130
7   156.1
6   120.7
1   139.8
2   123.5
3   128.5
4   126}

chemicalshift_standard_error {6 2.0}
chemicalshift_references {
6 176.317375 -0.922787}

aic {TRUE}
superimpose {TRUE}
superimpose_atoms {10,11,14,15,22,23,29,30,37,48,43}

noedistance_data {
58  103 2.2
58  56  2.4
58  60  2.4
58  59  2.6
58  67  3
62  72  2.6
62  60  2.4
62  61  2.9
62  66  2.7
75  92  2.7
72  78  2.1
60  73  4
60  (68,69,70)  2.7
59  (68,69,70)  2.6
85  92  2.9
85  (86,87) 3.2
85  (95,96,97)  2.6
86  87  1.78}
```

```
noedistance_standard_error {0.5}

jcoupling_data {
75 74   8.2 0.5 Pardi-[H-N-Ca-H]
75 73   3.9 0.5 Pardi-[H-N-Ca-H]
66 72   8.7 0.5 Pardi-[H-N-Ca-H]
62 61   6.9 0.5 Pardi-[H-N-Ca-H]
59 60   8.6 0.5 Pardi-[H-N-Ca-H]}

number_of_threads {8}
max_conformations {3}
```

Table S38. Stereofitter Input file for cyclic peptide **CP3** in methanol.

```
rdc_data {
6 47 6.8517
4 46 -15.4917
2 44 -18.2097
3 45 7.1337
27 65 16.651
32 70 9.3362
#CH2
23 62 -6.8584
23 63 16.7673
#CH2
40 82 14.6117
40 81 -11.3202
12 48 -15.5123
17 55 11.0982}
jcoupling_data {
61 63 7.7 0.5 Pardi-[H-N-Ca-H]
61 62 4.2 0.5 Pardi-[H-N-Ca-H]
66 70 6.5 0.5 Pardi-[H-N-Ca-H]
71 81 4.3 0.5 Pardi-[H-N-Ca-H]
71 82 7.1 0.5 Pardi-[H-N-Ca-H]}
chemicalshift_data {
17 65.5
20 177.4
23 43.5
24 171.7
28 175.3
32 53.6
33 175.1
40 44.2
41 170
1 140.4
2 124.8
3 130.8
4 123.5
5 125
6 120
```

```
7 166.2
10 167.3
12 60.8}
chemicalshift_standard_error {
6 2.0}

chemicalshift_references {
6 176.317375 -0.922787}

noedistance_data {
83 71 3.58 0.5
83 44 2.93 0.5
83 70 4.51 0.5
83 47 2.86 0.5
66 70 2.98 0.5
66 65 2.57 0.5
66 64 3.72 0.5
64 70 3.15 0.5
64 48 3.34 0.5
48 55 2.58 0.5
49 50 1.73 0.5}

rdc_standard_error {1.2}
aic {TRUE}
superimpose {TRUE}
superimpose_atoms {42,41,40,34,33,32,29,28,27,26,24,23,21,20,17,12}
number_of_threads {8}
max_conformations {3}
```

Table S39. Stereofitter Input file for cyclic peptide **CP4** in methanol.

```
rdc_data {
   13 54 4.13
   13 53 -10.3
   19 45 -1.27
   27 46 8.71
   30 70 3.19
    30 71 2.24
    32 47 -0.72
    33 48 2.97
    39 84 -17.04
    40 85 5.05
    41 86 -7.05
}
rdc_standard_error {  1.2  }
 chemicalshift_data {
   #gly5
   10 171.1
   13 44.6
    #4leu
   16 175.6
```

```
    19 52.8
    #3ala
    26 175.2
    27 50.6
    #2gly
    29 170.7
    30 42.6
    #1pro
    31 177.7
    32 64.50
    #6Het
    33 62.5
    44 168.5
    43 167.8
    4 135.5
    42 125
    1 16.22
    7 138.3
    39 130.83
    40 127.27
    41 128.75}
chemicalshift_standard_error {
    6 2.0}
chemicalshift_references {
6 176.317375 -0.922787}
jcoupling_data {
    46 69 6.4 0.5 Pardi-[H-N-Ca-H]
    45 65 7.8 0.5 Pardi-[H-N-Ca-H]
    72 71 6.9 0.5 Pardi-[H-N-Ca-H]
    72 70 3.3 0.5 Pardi-[H-N-Ca-H]
}
noedistance_data {
    72  47    3.1  0.5
    48  (75,76,77)    2.6  0.5
    48  47    3  0.5
    48  (78,79) 3.1  0.5
    47  (78,79) 3.65  0.5
    78  79    1.8  0.5}
aic {TRUE}
superimpose {TRUE}
superimpose_atoms {10,13,16,19,26,27,29,30,31,33,44,43}
number_of_threads {8}
max_conformations {2}
```

Table S40. Stereofitter Input file for cyclic peptide **CP5** in DMSO.

```
rdc_data {
28 68 9.3
28 69 -1.05
11 50 -11.61
11 49 2.32
32 71 -0.38
15 52 -5.65
37 78 1.92
23 63 8.99
3 46 16.54
2 45 8.60
5 47 8.00}
rdc_standard_error {1.2}

chemicalshift_data {
32  58.5
30  174.1
28  42.3
26  167.4
23  47.1
21  172.8
15  53.8
13  172.3
11  43.3
9   169
4   141.55
5   119.6
6   132.2
1   116.8
2   130.6
3   118.7
40  161.1
38  164.4
37  55.5}

chemicalshift_references {
6  176.480531 -0.928898}
chemicalshift_standard_error {
6 2.0}

noedistance_data {
46  48  2.0 0.5
47  48  3.1 0.5
48  (50,49) 2.6 0.5
48  52  3.0 0.5
51  52  1.9 0.5
52  53  2.3 0.5
53  63  2.4 0.5
63  64  2.6 0.5
64  (68,69) 2.1 0.5
```

```
70  71  2.0 0.5
72  73  1.8 0.5
51  48  2.1 0.5}

jcoupling_data {
63 64 8.1 0.5 Pardi-[H-N-Ca-H]
70 68 4.0 0.5 Pardi-[H-N-Ca-H]
70 69 4.0 0.5 Pardi-[H-N-Ca-H]
51 49 7.6 0.5 Pardi-[H-N-Ca-H]
51 50 4.0 0.5 Pardi-[H-N-Ca-H]}
aic {TRUE}
superimpose {TRUE}
superimpose_atoms {37,33,32,30,29,28,26,24,23,21,16,8}
max_conformations {3}
number_of_threads {8}
```

Table S41. Stereofitter Input file for cyclic peptide **CP6** in DMSO.

```
rdc_data {
4 41 -2.35
#2 39 -8.8
3 40 -5.59
8 44 0.86
8 43 1.39
32 63 18.1
12 46 4.49
26 58 10.03
22 55 5.0
22 56 7.3
#Proline and Alanine Side-Chain
#35 68 5.6
#35 69 -14.0
#(34,67) (34,66) -1.43
#(29,60) (29,61) (29,62) -3.1 }

rdc_standard_error {
1.2}

chemicalshift_data {
8 39.52
22 42.33
26 47.8
12 54.21
32 60.78
3 118.77
2 125.96
4 132.37
21 169.12
25 171.84
10 172.79}
```

```
chemicalshift_standard_error {
6 2.0}

chemicalshift_references {
6 176.480531 -0.928898}

aic {
TRUE}

superimpose {
TRUE}

superimpose_atoms {
1,2,3,4,5,6,8,9,10,12,13,21,22,23,25,26,28,30,32,36,37}

noedistance_data {
42 59 3.0173
42 45 3.4288
45 46 2.3774
59 58 2.8571
59 63 3.204
55 56 1.78
48 49 2.091}
noedistance_standard_error {
0.5}
number_of_threads {
8}

max_conformations {
3}
```

Table S42. Stereofitter Input file for the major peak set of cyclic peptide **CP7.1** in DMSO.

```
rdc_data {
2   40  -18.06
4   42  1.987
3   41  -0.0665
27  64  5.1876
19  56  11.811
11  45  17.4468
30  66  4.4948
30  67  19.2199
24  62  7.75776
24  61  -2.9562}

rdc_standard_error {1.2}

chemicalshift_data {
30 39.5
24 41.9
```

```
19 48.1
27 54.6
11 60.9
1 115.1
3 118.1
5 126.9
2 127.8
4 134.8
6 159.3
25 168.9
16 170.7
28 171.1
8 171.1
20 172.1}

chemicalshift_standard_error {
6 2.0}

chemicalshift_references {
6  176.480531 -0.928898}

noedistance_data {
44  46  2.6 0.5
44  45  3.0 0.5
44  55  2.7 0.5
44  40  2.2 0.5
45  46  3.0 0.5
55  46  3.1 0.5
55  45  3.1 0.5
67  66  1.8 0.5}

jcoupling_data  {
55 56   7   0.5 Pardi-[H-N-Ca-H]
60 62   7.1 0.5 Pardi-[H-N-Ca-H]
60 61   4.3 0.5 Pardi-[H-N-Ca-H]
63 64   8.5 0.5 Pardi-[H-N-Ca-H]}

aic {TRUE}
superimpose {TRUE}
superimpose_atoms {1,6,5,30,29,28,27,26,25,24,22,20,19,17,16,11,9,8}
number_of_threads {8}
max_conformations {2}
```

Table S43. Stereofitter Input file for the minor peak set of cyclic peptide **CP7.2** in DMSO.

```
rdc_data {
2 40 -13.2448
27 64 -4.382
19 56 12.998
11 45 -1.0752
12 46 8.53
24 62 17.09
24 61 -8.652
}
```

```
rdc_standard_error {
1.2
}

chemicalshift_data {
#carbon shifts
30 40.5
24 42
19 49.6
27 55.4
11 58.6
1 115.1
3 118.1
2 126.9
5 128.13
4 134.8
6 159.0
25 168.3
16 171.6
8 169.9
20 172.5}

chemicalshift_standard_error {
6 2.0
1 0.2
}

chemicalshift_references {
#reference
6 176.480531 -0.928898
}

noedistance_data {
44 40 2.3 0.5
44 46 2.7 0.5
55 40 2.3 0.5
55 60 2.8 0.5
55 56 3.3 0.5
55 45 2.3 0.5
63 64 2.7 0.5
60 56 3.1 0.5
45 44 3.2 0.5
61 62 1.8 0.5
}

aic {
TRUE
}

jcoupling_data {
55 56 6.8 0.5 Pardi-[H-N-Ca-H]
60 62 6.1 0.5 Pardi-[H-N-Ca-H]
60 61 4.4 0.5 Pardi-[H-N-Ca-H]
63 64 7.8 0.5 Pardi-[H-N-Ca-H]
}

superimpose {
TRUE
}

superimpose_atoms {
1,6,5,30,29,28,27,26,25,24,22,20,19,17,16,11,9,8
}

number_of_threads {
8
}

max_conformations {
1
}
```

Table S44. Stereofitter Input file for cyclic peptide **CP8** in DMSO.

```
rdc_data {
5   43  -12.1026
3   41  2.6231
4   42  -10.258
15  48  9.673
21  53  -0.3348
11  46  2.7441
25  55  4.757
25  56  -2.43
18  51  3.0217
18  50  1.42
#Proline
#38 68 3.3
#38 67 11.5
#40 71 4.0
#40 70 -0.8
# Alanine CH3
#(30,57) (30,58) (30,59) 0.26}

rdc_standard_error {1.2}

chemicalshift_data {
11  60.47
12  171.56
15  47.34
16  170.62
18  41.4
19  166.7
21  52.96
22  169.13
25  47.34
26  199.88
1   145.91
5   132.06
4   113.3
3   133
8   167.8}

chemicalshift_standard_error {
6 2.0}

chemicalshift_references {
6  176.480531 -0.928898}

noedistance_data {
54  55  2.8 0.5
54  43  3.5 0.5
54  53  2.3 0.5
52  42  4.0 0.5
```

```
52  49  4.0 0.5
52  53  3.1 0.5
52  43  3.9 0.5
47  (57,58,59)  2.6 0.5
47  46  3.2 0.5
47  49  3.5 0.5
47  48  3.1 0.5
43  42  2.5 0.5
49  48  2.7 0.5
49  (57,58,59)  3.9 0.5
49  46  3.8 0.5
48  (57,58,59)  2.3 0.5
60  61  1.8 0.5
67  68  1.9 0.5
}

jcoupling_data {
47 48 7.5 0.5 Pardi-[H-N-Ca-H]
53 52 6.9 0.5 Pardi-[H-N-Ca-H]}

aic {TRUE}
superimpose {TRUE}
superimpose_atoms {1,2,8,9,11,12,13,15,16,17,18,19,20,21,22,23,25,26,6}
number_of_threads {8}
max_conformations {3}
```

SI.13.2 Results from Stereofitter

SI.13.2.1 Ensemble ranking from Stereofitter

In the following the ensemble ranking as given by *Stereofitter* are shown for all cyclic peptides. In all cases a maximum number of conformers for one ensemble has been set to “3”, because in all cases sufficiently low *Q*-factors were found and in most cases the change from 2 to 3 had already reduced effect, compared to from a single conformer to 2. The top 10 ensembles for a maximum number of a single, two-conformer and three-conformer ensembles are shown. Furthermore, for the minor conformer of **CP7** a single-conformer was selected, due to the low number of experimental constraints, which still resulted in good agreement with the experimental data. In fact, when including more conformers the *Q*-factor rose (**Table S80**) and the AIC did only improve marginally, which we therefore concluded to be an artifact. As a final note, for cyclic peptide **CP1, CP5**, **CP6**, **CP7**, **CP8**, the conformer determined using ROE/MD[1,2] is also shown.

Table S45. Stereofitter ensemble ranking as obtained through the input file above for cyclic peptide **CP1** with 1 conformer. Additionally, the conformer from our previous study[1] is shown and highlighted in green.

Conf1 Index	$\chi^{(2/n)}$	χ^2	AIC	rel. prob.
85	2.625	112.88	112.88	1
81	3.169	136.275	136.275	0
82	3.192	137.241	137.241	0
73	3.193	137.281	137.281	0
74	3.194	137.349	137.349	0
27	3.209	137.983	137.983	0
34	3.582	154.035	154.035	0
17	3.618	155.56	155.56	0
16	3.69	158.685	158.685	0
ROE/MD[1]	4.997	214.880	214.880	0

Table S46. Stereofitter ensemble ranking as obtained through the input file above for cyclic peptide **CP1** with 2 conformers.

Conf1 Index	Conf1 %	Conf2 Index	Conf2 %	$\chi^{(2/n)}$	χ^2	AIC	rel. prob.
4	34.10%	85	65.90%	0.864	37.153	39.153	1
6	33.95%	85	66.05%	0.865	37.196	39.196	0.979
5	33.97%	85	66.03%	0.865	37.216	39.216	0.969
31	34.48%	85	65.52%	0.87	37.415	39.415	0.877
35	34.05%	85	65.95%	0.873	37.554	39.554	0.818
41	33.91%	85	66.09%	0.874	37.564	39.564	0.814
32	34.41%	85	65.60%	0.874	37.57	39.57	0.812
36	34.02%	85	65.98%	0.874	37.597	39.597	0.801
37	33.98%	85	66.02%	0.875	37.618	39.618	0.793
2	33.11%	85	66.89%	0.878	37.734	39.734	0.748

Table S47. Stereofitter ensemble ranking as obtained through the input file above for cyclic peptide **CP1** with 3 conformers.

Conf1 Index	Conf1 %	Conf2 Index	Conf2 %	Conf3 Index	Conf3 %	$\chi^{(2/n)}$	χ^2	AIC	rel. prob.
34	33.80%	80	16.19%	85	50.01%	0.664	28.539	32.539	0.954
21	15.67%	34	34.55%	85	49.78%	0.667	28.693	32.693	0.883
20	15.68%	34	34.51%	85	49.82%	0.667	28.699	32.699	0.88
73	33.88%	76	17.43%	85	48.69%	0.668	28.706	32.706	0.878

74	33.73%	76	17.46%	85	48.81%	0.668	28.73	32.73	0.867
34	34.37%	55	15.83%	85	49.81%	0.669	28.75	32.75	0.859
73	33.76%	75	17.42%	85	48.83%	0.669	28.759	32.759	0.855
72	33.80%	76	17.41%	85	48.80%	0.669	28.764	32.764	0.852
34	33.80%	80	16.19%	85	50.01%	0.664	28.539	32.539	0.954
21	15.67%	34	34.55%	85	49.78%	0.667	28.693	32.693	0.883

Table S48. Stereofitter ensemble ranking as obtained through the input file above for cyclic peptide **CP2** with 1 conformer.

Conf1 Index	$\chi^{(2/n)}$	χ^2	AIC	rel. prob.
1	1.891	98.338	98.338	1
22	2.149	111.744	111.744	0.001
36	2.407	125.151	125.151	0
35	2.416	125.628	125.628	0
7	2.451	127.472	127.472	0
49	2.46	127.915	127.915	0
30	2.478	128.868	128.868	0
37	2.49	129.475	129.475	0
23	2.501	130.075	130.075	0
18	2.521	131.109	131.109	0

Table S49. Stereofitter ensemble ranking as obtained through the input file above for cyclic peptide **CP2** with 2 conformers.

Conf1 Index	Conf1 %	Conf2 Index	Conf2 %	$\chi^{(2/n)}$	χ^2	AIC	rel. prob.
1	67.09	58	32.91	0.955	49.681	51.681	1
22	65.24	58	34.76	1.042	54.193	56.193	0.105
37	60.65	58	39.35	1.049	54.533	56.533	0.088
7	61.20	58	38.80	1.055	54.883	56.883	0.074
1	67.14	43	32.86	1.068	55.556	57.556	0.053
1	67.37	56	32.63	1.082	56.261	58.261	0.037
1	67.40	55	32.60	1.082	56.277	58.277	0.037
33	37.26	58	62.74	1.105	57.449	59.449	0.021
49	61.31	58	38.69	1.105	57.471	59.471	0.02

36	61.48	58	38.52	1.108	57.616	59.616	0.019

Table S50. Stereofitter ensemble ranking as obtained through the input file above for cyclic peptide **CP2**.

Conf1 Index	Conf1 %	Conf2 Index	Conf2 %	Conf3 Index	Conf3 %	$\chi^{(2/n)}$	χ^2	AIC	rel. prob.
33	26.64	58	37.59	99	35.77	0.779	40.505	44.505	1
7	38.13	33	25.65	58	36.22	0.781	40.618	44.618	0.945
33	27.01	37	34.33	58	38.66	0.791	41.108	45.108	0.74
18	33.31	33	27.36	58	39.33	0.796	41.415	45.415	0.634
33	28.10	58	41.95	67	29.95	0.797	41.463	45.463	0.619
30	33.69	33	27.84	58	38.48	0.797	41.465	45.465	0.619
33	28.20	58	41.67	70	30.13	0.801	41.648	45.648	0.565
33	27.28	36	34.67	58	38.05	0.802	41.701	45.701	0.55
33	27.91	35	34.36	58	37.73	0.804	41.794	45.794	0.525
33	28.01	58	49.42	98	22.57	0.809	42.084	46.084	0.454

Table S51. Stereofitter ensemble ranking as obtained through the input file above for cyclic peptide **CP3** with 1 conformer.

Conf1 Index	$\chi^{(2/n)}$	χ^2	AIC	rel. prob.
54	6.074	279.389	279.389	1.00
16	7.440	342.23	342.230	0.00
56	7.888	362.869	362.869	0.00
37	8.990	413.525	413.525	0.00
52	9.083	417.817	417.817	0.00
39	9.224	424.307	424.307	0.00
27	9.339	429.575	429.575	0.00
14	9.759	448.927	448.927	0.00
15	10.932	502.884	502.884	0.00
21	11.403	524.531	524.531	0.00

Table S52. Stereofitter ensemble ranking as obtained through the input file above for cyclic peptide **CP3** with 2 conformers.

Conf1 Index	Conf1 %	Conf2 Index	Conf2 %	$\chi^{(2/n)}$	χ^2	AIC	rel. prob.
11	56.87%	53	43.13%	1.968	90.518	92.518	1

35	25.47%	54	74.53%	2.61	120.056	122.056	0
11	57.92%	48	42.08%	3.086	141.943	143.943	0
32	23.28%	54	76.72%	3.186	146.571	148.571	0
38	23.07%	54	76.93%	3.288	151.226	153.226	0
11	65.15%	34	34.85%	3.342	153.732	155.732	0
11	58.93%	43	41.07%	3.364	154.728	156.728	0
34	36.76%	39	63.24%	3.584	164.881	166.881	0
50	21.83%	54	78.17%	3.736	171.851	173.851	0
51	21.78%	54	78.22%	3.747	172.362	174.362	0

Table S53. Stereofitter ensemble ranking as obtained through the input file above for cyclic peptide **CP3** with 3 conforms.

Conf1 Index	Conf1 %	Conf2 Index	Conf2 %	Conf3 Index	Conf3 %	χ(2/n)	χ2	AIC	rel. prob.
11	44.04	16	15.26	53	40.70	1.375	63.27	67.27	1
11	46.12	37	11.52	53	42.36	1.396	64.234	68.234	0.618
11	45.79	27	11.51	53	42.70	1.401	64.426	68.426	0.561
11	44.08	15	13.72	53	42.20	1.435	66.03	70.03	0.252
10	13.22	11	42.88	53	43.90	1.44	66.248	70.248	0.226
11	44.67	52	14.28	53	41.04	1.456	66.966	70.966	0.158
11	56.30	53	32.77	59	10.93	1.458	67.079	71.079	0.149
11	49.51	26	9.46	53	41.03	1.497	68.881	72.881	0.06
11	43.18	21	12.14	53	44.68	1.508	69.372	73.372	0.047
11	42.78	12	12.21	53	45.01	1.509	69.42	73.42	0.046

Table S54. Stereofitter ensemble ranking as obtained through the input file above for cyclic peptide **CP4** with 1 conformer.

Conf1 Index	$\chi^{(2/n)}$	χ^2	AIC	rel. prob.
23	2.934	117.354	117.354	1.000
22	2.946	117.859	117.859	0.777
12	3.458	138.309	138.309	0.000
2	4.397	175.882	175.882	0.000
11	4.657	186.290	186.290	0.000
3	4.678	187.127	187.127	0.000

10	4.729	189.177	189.177	0.000
5	4.829	193.154	193.154	0.000
9	5.051	202.037	202.037	0.000
7	5.602	224.064	224.064	0.000

Table S55. Stereofitter ensemble ranking as obtained through the input file above for cyclic peptide **CP4** with 2 conformers.

Conf1 Index	Conf1 %	Conf2 Index	Conf2 %	$\chi^{(2/n)}$	χ^2	AIC	rel. prob.
2	49.06	12	50.94	1.375	54.986	56.986	1.00
3	48.41	12	51.59	1.560	62.387	64.387	0.025
9	47.13	12	52.87	1.560	62.398	64.398	0.025
5	47.93	12	52.07	1.568	62.735	64.735	0.021
2	40.81	23	59.19	1.605	64.198	66.198	0.010
5	40.27	23	59.73	1.606	64.250	66.250	0.010
2	40.87	22	59.14	1.610	64.394	66.394	0.009
5	40.33	22	59.67	1.614	64.545	66.545	0.008
11	47.88	12	52.12	1.620	64.792	66.792	0.007
10	48.21	12	51.79	1.638	65.534	67.534	0.005

Table S56. Stereofitter ensemble ranking as obtained through the input file above for cyclic peptide **CP4** with 3 conformers.

Conf1 Index	Conf1 %	Conf2 Index	Conf2 %	Conf3 Index	Conf3 %	$\chi(2/n)$	$\chi 2$	AIC	rel. prob.
5	36.32	12	42.39	13	21.29	1.142	46.670	49.670	1.00
5	35.40	12	41.58	16	23.02	1.164	46.571	50.571	0.637
2	39.20	12	43.71	13	17.09	1.170	46.793	50.793	0.570
11	30.24	12	41.31	16	28.45	1.197	47.866	51.866	0.333
2	39.04	12	43.19	16	17.77	1.197	47.880	51.880	0.331
11	33.01	12	42.09	13	23.65	1.198	47.939	51.939	0.322
10	34.62	12	41.73	13	23.65	1.217	48.681	52.681	0.222
3	36.47	12	42.73	13	20.81	1.219	48.762	52.762	0.213
10	31.94	12	40.83	16	27.24	1.228	49.107	53.107	0.179
7	34.03	12	42.52	13	34.03	1.230	49.185	53.185	0.172

Table S57. Stereofitter ensemble ranking as obtained through the input file above for cyclic peptide **CP5** with 1 conformer. Additionally, the conformer from our previous study[1] is shown and highlighted in green.

Conf1 Index	$\chi^{(2/n)}$	χ^2	AIC	rel. prob.
18	2.830	133.017	133.017	1.00
50	2.839	133.423	133.423	0.816
61	2.845	133.704	133.704	0.709
49	3.069	144.224	144.224	0.004
27	3.077	144.632	144.632	0.003
28	3.103	145.857	145.857	0.002
95	3.160	138.513	138.513	0.000
47	3.197	150.279	150.279	0.000
100	5.333	250.665	250.665	0.000
ROE/MD[1]	6.259	294.151	294.151	–

Table S58. Stereofitter ensemble ranking as obtained through the input file above for cyclic peptide **CP5** with 2 conformers.

Conf1 Index	Conf1 %	Conf2 Index	Conf2 %	$\chi^{(2/n)}$	χ^2	AIC	rel. prob.
18	75.28	36	24.74	1.702	79.994	81.994	1.00
36	24.49	50	75.51	1.718	80.746	82.746	0.687
36	26.48	47	73.52	1.797	84.480	86.480	0.106
18	74.67	34	25.33	1.838	86.370	88.370	0.041
34	25.03	50	74.97	1.846	86.748	88.748	0.034
36	20.31	61	79.69	1.858	87.345	89.345	0.025
27	74.78	36	25.22	1.887	88.677	90.677	0.013
36	24.78	49	75.22	1.896	89.102	91.102	0.011
28	74.69	36	25.31	1.903	89.437	91.437	0.009
61	77.35	77	22.65	1.944	91.364	93.364	0.003

Table S59. Stereofitter ensemble ranking as obtained through the input file above for cyclic peptide **CP5** with 3 conformers.

Conf1 Index	Conf1 %	Conf2 Index	Conf2 %	Conf3 Index	Conf3 %	χ(2/n)	χ2	AIC	rel. prob.
2	49.78	7	19.01	109	31.22	1.494	70.212	74.212	1.00
2	50.36	7	18.52	108	31.12	1.505	70.745	74745	0.766

36	22.17	47	62.66	94	15.17	1.513	71.097	75.097	0.643
36	22.23	47	62.54	87	15.23	1.515	71.198	75.198	0.611
6	52.72	7	16.27	109	31.01	1.517	71.310	75.310	0.578
1	52.96	7	15.69	109	31.36	1.520	71.423	75.423	0.546
6	53.38	7	15.65	108	30.97	1.526	71.701	75.701	0.475
1	51.34	7	18.35	117	30.31	1.529	71.853	75.853	0.440
1	53.65	7	15.01	108	31.33	1.533	72.028	76.028	0.403
2	50.10	20	19.78	109	30.12	15.33	72.066	76.066	0.396

Table S60. Stereofitter ensemble ranking as obtained through the input file above for cyclic peptide **CP6** with 1 conformer. Additionally, the conformer from our previous study[2] is shown and highlighted in green. The conformers from the *cis*- and *trans*-sampling have been combined. 9 RDCs were used.

**Conf1 Index**	$\chi^{(2/n)}$	χ^2	**AIC**	**rel. prob.**
15	3.040	82.089	82.089	1.000
14	3.138	84.734	84.734	0.266
13	3.292	88.889	88.889	0.033
16	3.297	89.016	89.016	0.031
12	3.392	91.586	91.586	0.009
26	3.399	91.766	91.766	0.008
7	3.847	103.872	103.872	0.000
9	3.908	105.523	105.523	0.000
10	4.108	110.910	110.910	0.000
11	4.142	111.821	111.821	0.000
ROE/MD[2]	5.728	154.666	154.666	–

Table S61. Stereofitter ensemble ranking as obtained through the input file above for cyclic peptide **CP6** with 1 conformer. Additionally, the conformer from our previous study[2] is shown and highlighted in green. The conformers from the *cis*- and *trans*-sampling have been combined. 13 RDCs were used.

**Conf1 Index**	$\chi^{(2/n)}$	χ^2	**AIC**	**rel. prob.**
49	5.626	174.395	174.395	1
50	5.626	174.418	174.418	0.989
51	5.628	174.454	174.454	0.971
48	5.638	174.776	174.776	0.827

44	6.499	201.476	201.476	0
41	6.526	202.306	202.306	0
33	6.612	204.959	204.959	0
42	6.666	206.64	206.64	0
45	6.668	206.694	206.694	0
43	6.669	206.744	206.744	0
ROE/MD[2]	6.861	212.689	212.689	–

Table S62. Stereofitter ensemble ranking as obtained through the input file above for cyclic peptide **CP6** with 2 conformers. The conformers from the *cis*- and *trans*-sampling have been combined. 9 RDCs were used.

Conf1 Index	Conf1 %	Conf2 Index	Conf2 %	$\chi^{(2/n)}$	χ^2	AIC	rel. prob.
4	43.61	50	56.39	0.595	16.067	18.067	1
4	43.61	49	56.39	0.598	16.152	18.152	0.958
4	43.61	48	56.39	0.599	16.182	18.182	0.944
3	42.63	49	57.37	0.605	16.339	18.339	0.872
4	43.60	51	56.40	0.605	16.344	18.344	0.87
3	42.63	50	57.37	0.613	16.559	18.559	0.782
3	42.62	48	57.38	0.62	16.751	18.751	0.71
3	42.61	51	57.39	0.627	16.938	18.938	0.647
3	42.61	46	57.39	0.64	17.283	19.283	0.544
4	43.48	46	56.52	0.681	18.385	20.385	0.314

Table S63. Stereofitter ensemble ranking as obtained through the input file above for cyclic peptide **CP6** with 2 conformers. The conformers from the *cis*- and *trans*-sampling have been combined. 13 RDCs were used.

Conf1 Index	Conf1 %	Conf2 Index	Conf2 %	$\chi^{(2/n)}$	χ^2	AIC	rel. prob.
19	42.67%	48	57.33%	1.508	46.741	48.741	1
19	42.66%	51	57.34%	1.521	47.165	49.165	0.809
18	42.80%	48	57.20%	1.524	47.237	49.237	0.78
19	42.67%	50	57.33%	1.525	47.276	49.276	0.765
19	42.69%	49	57.31%	1.535	47.586	49.586	0.655
18	42.80%	51	57.20%	1.538	47.669	49.669	0.629
18	42.81%	50	57.19%	1.542	47.792	49.792	0.591

18	42.82%	49	57.18%	1.552	48.123	50.123	0.501
19	43.53%	41	56.47%	2.044	63.349	65.349	0
19	43.60%	44	56.40%	2.058	63.808	65.808	0

Table S64. Stereofitter ensemble ranking as obtained through the input file above for cyclic peptide **CP6** with 3 conformers. The conformers from the *cis*- and *trans*-sampling have been combined. 9 RDCs were used.

Conf1 Index	Conf1 %	Conf2 Index	Conf2 %	Conf3 Index	Conf3 %	$\chi^{(2/n)}$	χ^2	AIC	rel. prob.
4	43.61	50	56.39	–	–	0.595	16.067	18.067	1
4	43.61	49	56.39	–	–	0.598	16.152	18.152	0.958
4	43.61	48	56.39	–	–	0.599	16.182	18.182	0.944
3	40.02	9	5.12	50	54.86	0.528	14.244	18.244	0.915
3	40.97	10	4.13	50	54.90	0.529	14.272	18.272	0.902
3	39.88	9	5.32	48	54.80	0.53	14.312	18.312	0.885
3	40.91	10	4.25	48	54.83	0.531	14.338	18.338	0.873
3	42.63	49	57.37	–	–	0.605	16.339	18.339	0.872
4	43.60	51	56.40	–	–	0.605	16.344	18.344	0.87
3	40.21	7	4.86	50	54.93	0.532	14.365	18.365	0.861

Table S65. Stereofitter ensemble ranking as obtained through the input file above for cyclic peptide **CP6** with 3 conformers. 13 RDCs used.

Conf1 Index	Conf1 %	Conf2 Index	Conf2 %	Conf3 Index	Conf3 %	$\chi^{(2/n)}$	χ^2	AIC	rel. prob.
19	42.67%	48	57.33%	–	–	1.508	46.741	48.741	1
19	42.66%	51	57.34%	–	–	1.521	47.165	49.165	0.809
18	42.81%	48	57.19%	–	–	1.524	47.237	49.237	0.78
19	42.68%	50	57.32%	–	–	1.525	47.276	49.276	0.765
17	8.44%	19	36.65%	48	54.91%	1.468	45.502	49.502	0.683
19	42.68%	49	57.32%	–	–	1.535	47.586	49.586	0.655
18	42.79%	51	57.21%	–	–	1.538	47.669	49.669	0.629
18	42.81%	50	57.19%	–	–	1.542	47.792	49.792	0.591
17	8.05%	19	36.94%	51	55.00%	1.48	45.893	49.893	0.562
17	7.91%	19	37.04%	50	55.05%	1.483	45.987	49.987	0.536

Table S66. Stereofitter ensemble ranking as obtained through the input file above for cyclic peptide **CP6** with 3 conformers. Using the (*trans*-proline conformers and *cis*-proline conformers separate) with 9 RDCs.

Conf1 Index	Conf1 %	Conf2 Index	Conf2 %	Conf3 Index	Conf3 %	$\chi^{(2/n)}$	χ^2	AIC	rel. prob.
15	78.21	24	21.79	–	–	2.756	74.401	76.401	1
15	78.31	23	21.69	–	–	2.758	74.475	76.475	0.964
15	78.24	20	21.76	–	–	2.77	74.781	76.781	0.827
5	3.56	15	76.54	24	19.90	2.754	74.368	78.368	0.374
5	4.81	15	76.03	23	19.16	2.756	74.417	78.417	0.365
15	78.28	20	1.36	23	20.36	2.759	74.484	78.484	0.353
5	5.14	15	75.91	20	18.94	2.767	74.714	78.714	0.315
5	32.41	16	67.59	–	–	2.847	76.858	78.858	0.293
5	30.51	15	69.49	–	–	2.85	76.956	78.956	0.279
16	76.14	24	23.86	–	–	2.899	78.272	80.272	0.144
50	100	–	–	–	–	4.928	133.052	133.052	1
48	100	–	–	–	–	4.933	133.199	133.199	0.929
51	100	–	–	–	–	4.945	133.524	133.524	0.79
49	100	–	–	–	–	4.961	133.937	133.937	0.642
44	100	–	–	–	–	5.913	159.654	159.654	0
44	75.76	47	24.24	–	–	5.903	159.368	161.368	0
44	89.95	46	10.05	–	–	5.911	159.588	161.588	0
47	100	–	–	–	–	6.016	162.431	162.431	0
41	43.87	47	56.13	–	–	5.959	160.892	162.892	0
41	100	–	–	–	–	6.046	163.24	163.24	0

Table S67. Stereofitter ensemble ranking as obtained through the input file above for cyclic peptide **CP6** with 3 conformers. Using the (*trans*-proline conformers and *cis*-proline conformers separate) with 13 RDCs.

Conf1 Index	Conf1 %	Conf2 Index	Conf2 %	Conf3 Index	Conf3 %	$\chi^{(2/n)}$	χ^2	AIC	rel. prob.
19	100%	–	–	–	–	5.626	174.395	174.395	1
20	100%	–	–	–	–	5.626	174.418	174.418	0.989
21	100%	–	–	–	–	5.628	174.454	174.454	0.971
18	100%	–	–	–	–	5.638	174.776	174.776	0.827
17	11.18%	19	88.82%	–	–	5.592	173.358	175.358	0.618

17	11.48%	21	88.52%	–	–	5.592	173.365	175.365	0.616
17	11.21%	20	88.79%	–	–	5.593	173.384	175.384	0.61
17	11.47%	18	88.53%	–	–	5.603	173.689	175.689	0.524
16	9.33%	19	90.67%	–	–	5.604	173.737	175.737	0.511
16	9.55%	21	90.45%	–	–	5.605	173.767	175.767	0.504
4	43.57%	18	22.07%	29	34.36%	8.439	261.603	265.603	1
4	44.35%	19	21.65%	29	33.99%	8.451	261.969	265.969	0.833
3	44.18%	18	20.26%	29	35.57%	8.462	262.317	266.317	0.7
3	45.11%	19	19.75%	29	35.14%	8.472	262.63	266.63	0.599
1	34.60%	4	43.00%	18	22.40%	8.567	265.566	269.566	0.138
1	34.45%	4	43.44%	19	22.11%	8.576	265.855	269.855	0.119
3	76.15%	29	23.85%	–	–	8.641	267.869	269.869	0.118
1	35.99%	3	42.61%	18	21.41%	8.606	266.775	270.775	0.075
1	35.76%	3	43.18%	19	21.06%	8.614	267.023	271.023	0.067
4	78.02%	29	21.98%	–	–	8.701	269.725	271.725	0.047

Table S68. Stereofitter ensemble ranking as obtained through the input file above for the major peak set of cyclic peptide **CP7.1** with 1 conformer. Additionally, the conformer from our previous study[2] is shown and highlighted in green.

**Conf1 Index**	$\chi^{(2/n)}$	χ^2	**AIC**	**rel. prob.**
34	1.674	63.598	63.598	1
ROE/MD[2]	2.038	77.44	77.44	–
18	2.317	88.041	88.041	0
16	2.759	104.844	104.844	0
15	2.765	105.062	105.062	0
30	2.881	109.496	109.496	0
19	2.963	112.587	112.587	0
22	3.195	121.402	121.402	0
32	3.281	124.67	124.67	0
33	3.281	124.679	124.679	0
29	3.297	125.29	125.29	0

Table S69. Stereofitter ensemble ranking as obtained through the input file above for the major peak set of cyclic peptide **CP7.1** with 2 conformers.

Conf1 Index	Conf1 %	Conf2 Index	Conf2 %	$\chi^{(2/n)}$	χ^2	AIC	rel. prob.
18	62.68	31	37.32	0.829	31.5	33.5	1
14	37.31	18	62.69	0.87	33.053	35.053	0.46
18	63.27	27	36.73	0.884	33.591	35.591	0.352
13	35.92	18	64.08	0.894	33.973	35.973	0.29
14	37.68	16	62.32	0.915	34.772	36.772	0.195
13	36.13	16	63.87	0.92	34.961	36.961	0.177
16	62.33	31	37.67	0.931	35.375	37.375	0.144
18	63.18	24	36.82	0.944	35.887	37.887	0.112
15	61.32	31	38.68	0.946	35.956	37.956	0.108
16	62.95	27	37.05	0.983	37.361	39.361	0.053

Table S70. Stereofitter ensemble ranking as obtained through the input file above for the major peak set of cyclic peptide **CP7.1** with 3 conformers.

Conf1 Index	Conf1 %	Conf2 Index	Conf2 %	Conf3 Index	Conf3 %	$\chi^{(2/n)}$	χ^2	AIC	rel. prob.
18	62.68	31	37.32	—	—	0.829	31.5	33.5	1
7	7.47	18	61.16	31	31.37	0.783	29.747	33.747	0.884
6	7.02	18	61.47	31	31.51	0.788	29.948	33.948	0.799
11	6.79	18	61.79	31	31.42	0.793	30.141	34.141	0.726
12	6.50	18	62.02	31	31.48	0.795	30.222	34.222	0.697
5	6.40	18	62.03	31	31.56	0.802	30.477	34.477	0.614
4	5.82	18	62.14	31	32.04	0.806	30.636	34.636	0.567
2	5.66	18	61.90	31	32.44	0.807	30.651	34.651	0.562
3	5.35	18	62.26	31	32.39	0.809	30.737	34.737	0.539
1	5.00	18	62.24	31	32.76	0.813	30.88	34.88	0.502

Table S71. Stereofitter ensemble ranking as obtained through the input file above for the minor peak set of cyclic peptide **CP7.2**. Additionally, the conformer from our previous study[2] is shown and highlighted in green.

Conf1 Index	$\chi^{(2/n)}$	χ^2	AIC	rel. prob.
23	1.366	49.17	49.17	1
24	1.594	57.384	57.384	0.016

27	1.82	65.516	65.516	0
14	1.908	68.69	68.69	0
13	2.161	77.803	77.803	0
31	2.579	92.841	92.841	0
33	3.099	111.581	111.581	0
4	3.186	114.68	114.68	0
7	3.192	114.929	114.929	0
17	3.286	118.28	118.28	0
ROE/MD[2]	6.948	250.141	250.1441	–

Table S72. Stereofitter ensemble ranking as obtained through the input file above for the minor peak set of cyclic peptide **CP7.2** with 2 conformers.

Conf1 Index	Conf1 %	Conf2 Index	Conf2 %	$\chi^{(2/n)}$	χ^2	AIC	rel. prob.
17	31.72%	23	68.28%	0.944	33.966	35.966	1
23	50.79%	31	49.21%	1.021	36.748	38.748	0.249
4	21.96%	16	78.04%	1.044	37.59	39.59	0.163
5	27.22%	34	72.78%	1.044	37.595	39.595	0.163
6	21.33%	34	78.67%	1.054	37.942	39.942	0.137
12	19.95%	34	80.05%	1.073	38.628	40.628	0.097
11	19.94%	34	80.06%	1.073	38.632	40.632	0.097
13	34.21%	23	65.79%	1.089	39.206	41.206	0.073
1	28.41%	34	71.59%	1.09	39.244	41.244	0.071
14	35.89%	23	64.11%	1.091	39.291	41.291	0.07

Table S73. Stereofitter ensemble ranking as obtained through the input file above for the minor peak set of cyclic peptide **CP7.2** with 3 conformers.

Conf1 Index	Conf1 %	Conf2 Index	Conf2 %	Conf3 Index	Conf3 %	$\chi^{(2/n)}$	χ^2	AIC	rel. prob.
4	23.29%	16	29.66%	31	47.05%	0.81	29.165	33.165	1
4	21.42%	30	27.15%	31	51.43%	0.818	29.444	33.444	0.87
7	36.42%	13	42.99%	25	20.59%	0.821	29.541	33.541	0.829
4	21.44%	15	26.97%	31	51.59%	0.83	29.871	33.871	0.703
5	15.84%	30	29.55%	31	54.60%	0.848	30.539	34.539	0.503

1	19.13%	30	26.81%	31	54.06%	0.851	30.65	34.65	0.476
4	23.87%	29	27.17%	31	48.96%	0.852	30.671	34.671	0.471
7	37.43%	13	42.77%	26	19.80%	0.853	30.693	34.693	0.466
11	35.44%	13	44.31%	25	20.25%	0.854	30.746	34.746	0.454
3	24.79%	30	19.94%	31	55.27%	0.857	30.844	34.844	0.432

Table S74. Stereofitter ensemble ranking as obtained through the input file above for cyclic peptide **CP8** with 1 conformer and 10 RDCs. Additionally, the conformer from our previous study[2] is shown and highlighted in green.

**Conf1 Index**	$\chi^{(2/n)}$	χ^2	**AIC**	**rel. prob.**
7	0.78	35.107	35.107	1
6	0.781	35.153	35.153	0.977
9	0.833	37.495	37.495	0.303
10	0.933	41.989	41.989	0.032
8	0.948	42.664	42.664	0.023
26	1.723	77.531	77.531	0
29	1.947	87.628	87.628	0
12	4.191	188.596	188.596	0
16	4.2	189.01	189.01	0
13	4.201	189.054	189.054	0
ROE/MD[2]	16.498	742.407	742.407	–

Table S75. Stereofitter ensemble ranking as obtained through the input file above for cyclic peptide **CP8** with 1 conformer and 15 RDCs. Additionally, the conformer from our previous study[2] is shown and highlighted in green.

**Conf1 Index**	$\chi^{(2/n)}$	χ^2	**AIC**	**rel. prob.**
7	0.736	36.824	36.824	1
6	0.737	36.854	36.854	0.985
8	0.889	44.439	44.439	0.022
9	1.315	65.725	65.725	0
10	1.335	66.760	66.760	0
26	1.626	81.319	81.319	0
29	2.273	113.654	113.654	0
12	4.053	202.633	202.633	0

16	4.063	203.143	203.143	0
14	4.063	203.169	203.169	0
ROE/MD[2]	14.650	732.507	732.507	–

Table S76. Stereofitter ensemble ranking as obtained through the input file above for cyclic peptide **CP8** with 2 conformers and 10 RDCs.

Conf1 Index	Conf1 %	Conf2 Index	Conf2 %	$\chi^{(2/n)}$	χ^2	AIC	rel. prob.
6	76.36%	23	23.64%	0.446	20.068	22.068	1
7	76.37%	23	23.63%	0.448	20.154	22.154	0.958
6	76.31%	25	23.69%	0.47	21.132	23.132	0.587
7	76.32%	25	23.68%	0.471	21.199	23.199	0.568
9	79.57%	23	20.43%	0.557	25.073	27.073	0.082
9	79.13%	25	20.87%	0.57	25.671	27.671	0.061
8	75.91%	23	24.09%	0.583	26.248	28.248	0.045
8	75.61%	25	24.39%	0.603	27.126	29.126	0.029
10	78.96%	23	21.04%	0.625	28.109	30.109	0.018
10	78.37%	25	21.63%	0.636	28.602	30.602	0.014

Table S77. Stereofitter ensemble ranking as obtained through the input file above for cyclic peptide **CP8** with 2 conformers and 15 RDCs.

Conf1 Index	Conf1 %	Conf2 Index	Conf2 %	$\chi^{(2/n)}$	χ^2	AIC	rel. prob.
6	79.77%	23	20.23%	0.49	24.497	26.497	1
7	79.90%	23	20.10%	0.493	24.642	26.642	0.93
6	79.76%	25	20.24%	0.502	25.088	27.088	0.744
7	79.87%	25	20.13%	0.504	25.203	27.203	0.703
8	80.41%	23	19.59%	0.645	32.228	34.228	0.021
8	80.42%	25	19.58%	0.655	32.748	34.748	0.016
6	89.24%	24	10.76%	0.663	33.152	35.152	0.013
7	89.64%	24	10.36%	0.668	33.389	35.389	0.012
5	10.16%	6	89.84%	0.679	33.969	35.969	0.009
5	10.11%	7	89.89%	0.68	33.977	35.977	0.009

Table S78. Stereofitter ensemble ranking as obtained through the input file above for cyclic peptide **CP8** with 3 conformers and 10 RDCs.

**Conf1 Index**	**Conf1 %**	**Conf2 Index**	**Conf2 %**	**Conf3 Index**	**Conf3 %**	$\chi^{(2/n)}$	χ^2	**AIC**	**rel. prob.**
6	76.36	23	23.64	—	—	0.446	20.068	22.068	1
7	76.37	23	23.63	—	—	0.448	20.154	22.154	0.958
6	71.16	23	23.07	32	5.78	0.419	18.847	22.847	0.677
7	71.24	23	23.05	32	5.72	0.422	18.969	22.969	0.637
6	76.31	25	23.69	—	—	0.47	21.132	23.132	0.587
7	76.32	25	23.68	—	—	0.471	21.199	23.199	0.568
6	73.10	23	22.00	31	4.90	0.436	19.628	23.628	0.458
6	73.49	23	22.02	24	4.49	0.437	19.648	23.648	0.454
6	70.49	25	23.24	32	6.27	0.437	19.677	23.677	0.447
7	73.17	23	22.02	31	4.81	0.439	19.751	23.751	0.431

Table S79. Stereofitter ensemble ranking as obtained through the input file above for cyclic peptide **CP8** with 3 conformers and 15 RDCs.

**Conf1 Index**	**Conf1 %**	**Conf2 Index**	**Conf2 %**	**Conf3 Index**	**Conf3 %**	$\chi^{(2/n)}$	χ^2	**AIC**	**rel. prob.**
6	79.77%	23	20.23%	–	–	0.49	24.497	26.497	1
7	79.89%	23	20.11%	–	–	0.493	24.642	26.642	0.93
6	79.77%	25	20.23%	–	–	0.502	25.088	27.088	0.744
7	79.87%	25	20.13%	–	–	0.504	25.203	27.203	0.703
6	71.56%	23	20.37%	29	8.071%	0.479	23.927	27.927	0.489
7	71.15%	23	20.28%	29	8.565%	0.48	24.001	28.001	0.471
6	76.53%	23	19.62%	32	3.845%	0.48	24.002	28.002	0.471
6	63.90%	9	16.02%	23	20.08%	0.481	24.042	28.042	0.462
7	61.98%	9	17.92%	23	20.10%	0.481	24.043	28.043	0.462
6	75.32%	15	5.027%	23	19.65%	0.481	24.047	28.047	0.461

SI.13.2.3 *Q*-factors of lowest AIC ensemble

In the following **Table S80** the *Q*-factors of the lowest AIC ensembles for each cyclic peptide are shown. For **CP1** and **CP5–CP8** the *Q*-factors for the previously determined conformer are shown as well. Upon addition of the $^{1}D_{NH}$ restraint extracted, shown in **Tables S1–S9**, the *Q*-factors deteriorated drastically

and were therefore excluded from the conformational analysis. While in most cases we were able to measure the RDCs, due to strong line-broadening the extracted restraints were often of poor quality, which might explain the inferior performance.

Table S80. Q-factors for the ensemble with the lowest AIC with and without -NH RDC data.

	***Q*-factor of backbone RDCs**							
	**1 Conf. Ensemble**		**2 Conf. Ensemble**		**3 Conf. Ensemble**		**ROE/MD**[1,2]	
	$^{1}D_{CH}$	$^{1}D_{CH}+{}^{1}D_{NH}$	$^{1}D_{CH}$	$^{1}D_{CH}+{}^{1}D_{NH}$	$^{1}D_{CH}$	$^{1}D_{CH}+{}^{1}D_{NH}$	$^{1}D_{CH}$	$^{1}D_{CH}+{}^{1}D_{NH}$
**CP1**	0.21	0.48	0.09	0.30	0.08	0.28	0.40	0.52
**CP2**	0.50	0.69	0.25	0.63	0.15	0.53	–	
**CP3**	0.32	0.35	0.19	0.23	0.13	0.19	–	
**CP4**	0.39	0.53	0.16	0.33	0.15	0.31	–	
**CP5**	0.32	0.70	0.26	0.61	0.10	0.58	0.39	0.66
**CP6 (cis+trans)**	0.37	0.41	0.13[a]	0.23[a]	0.13[a]	0.23[a]	0.60	0.62
**CP6 13 RDCs (cis+trans)**	0.55	–	0.22	–	0.22	–	0.60	–
**CP7.1**	0.14	0.47	0.07[a]	0.43[a]	0.07[a]	0.43[a]	0.23	0.58
**CP7.2**	0.05	0.41	0.08	0.27	–	–	0.61	0.68
**CP8**	0.18	0.65	0.09[a]	0.49[a]	0.09	0.48	0.45	0.56
**CP8 15 RDCs**	0.17	–	0.13	–	0.13	–	0.40	–

[a] Always the same two conformer ensemble selected by Stereofitter.

SI.14 Computational details for proline *cis*/*trans*-isomerization transition barrier

To determine the barrier height of the *cis*-*trans*-isomerization of proline, fragments of the aryl-proline motive of **CP6** and **CP8** have been used. The C-termini have been methylated. For comparison a small dipeptide Ala-Pro has been used as reference. For each system the *cis*- and *trans*-conformer have been used in ORCA NEB-TS[35] calculation using the following settings.

Table S81. Example Input file for xTB-GFN2/ALPB(DMSO) NEB-CI calculation in ORCA.

```
!XTB2 ALPB(DMSO) NEB-TS
%pal
 nprocs 40
end

%NEB
 NEB_END_XYZFILE  "end.xyz"
 Nimages        20
 MaxIter        1000
 SIDPP         TRUE
 preopt      TRUE
END

* XYZFILE 0 1 inp.xyz
```

After successful converges of the NEB-TS calculation the educt, product and transition state have been reoptimized at the PW6B95-*D4*/def2-TZVP/SMD(DMSO) level of theory. Thereby no imaginary frequencies were observed for all educt and product structures and a single imaginary frequency for the transition states. The estimated free energies for each system and structure are shown below. For the obtained transition states as well as ground state structures energy decomposition analysis in the extended transition state with natural orbitals for valency (ETS-NOCV) framework have been conducted in ORCA 6.1 at the PW6B95-D4/def2-TZVP/SMD(DMSO) level of theory. The differences (ground state and transition state) between the different energy contributions are depicted in **Figure S24**. All optimized structures are deposited at: [10.5281/zenodo.19909872](https://doi.org/10.5281/zenodo.19909872)

Table S82. Calculated Gibbs Free Energies at the PW6B95-*D4*/def2-TZVP/SMD(DMSO) level of theory.

System	Structure	Gibbs Free Energy in [E_h]	Imaginary Frequency
CP6	Educt	-841.41593905	–
CP6	Product	-841.41415509	–
CP6	Transition State	-841.38627793	1
CP8	Educt	-821.51385593	–
CP8	Product	-821.51314432	–
CP8	Transition State	-821.49176944	1
Ala-Pro	Educt	-821.71922353	–
Ala-Pro	Product	-821.70932855	–
Ala-Pro	Transition State	-821.68094938	1

SI.15 Molecular dynamics simulations

SI.15.1 General simulation settings

All molecular dynamics simulation have been done using the GROMACS[36–42] simulation package. GAFF[43] force field has been parametrized for DMSO starting from a PW6B95/def2-TZVP optimized structure and for the **CP7** conformers determined using our NMR/DFT approach. For all structures geometry optimization and subsequent RESP-charge calculations have been done at the HF/6-31G* level of theory with the “tight” SCF criterion set in Gaussian16[44]. The gaussian output files have been used in *antechamber* for the generation of the GAFF force field files.[45,46]

System have been build using the GROMACS *editconf* and *solvate* commands. In each case one **CP7** molecule has been inserted into a solvent box of 3.09 nm x 3.09 nm x 3.09 nm DMSO molecules. The systems have been minimized and equilibrated. For equilibration, NVT simulations at 300 K have been conducted for 1 ns, followed by 1 ns of NPT simulations at 300 K and 1 bar, both without bond restraints and 10 ns of NPT at 300 K and 1 bar with LINCS restraints for all bonds to hydrogen-atoms. For the temperature coupling the *V-rescale* with a τ_T of 1 ps was used and for the pressure coupling the *C-rescale* with a τ_P of 2 ps. For the VdW-interactions a simple Cut-Off of 1.0 nm was used and for the Coulomb-interactions the Particle-mesh Ewald (PME) method with a radius of 1.0 nm was used. The PME-order was set to 4. Using a timestep of 2 fs, energy values and coordinates were saved every 2 ps.

SI.15.2 RESP charges from different conformers

Using the three different conformers determined in our NMR/DFT approach, different RESP charges were obtained, when calculated using Gaussian16. In the following the charges are shown for all atoms besides hydrogens. Unbiased simulation with each force field parameters are shown as well.

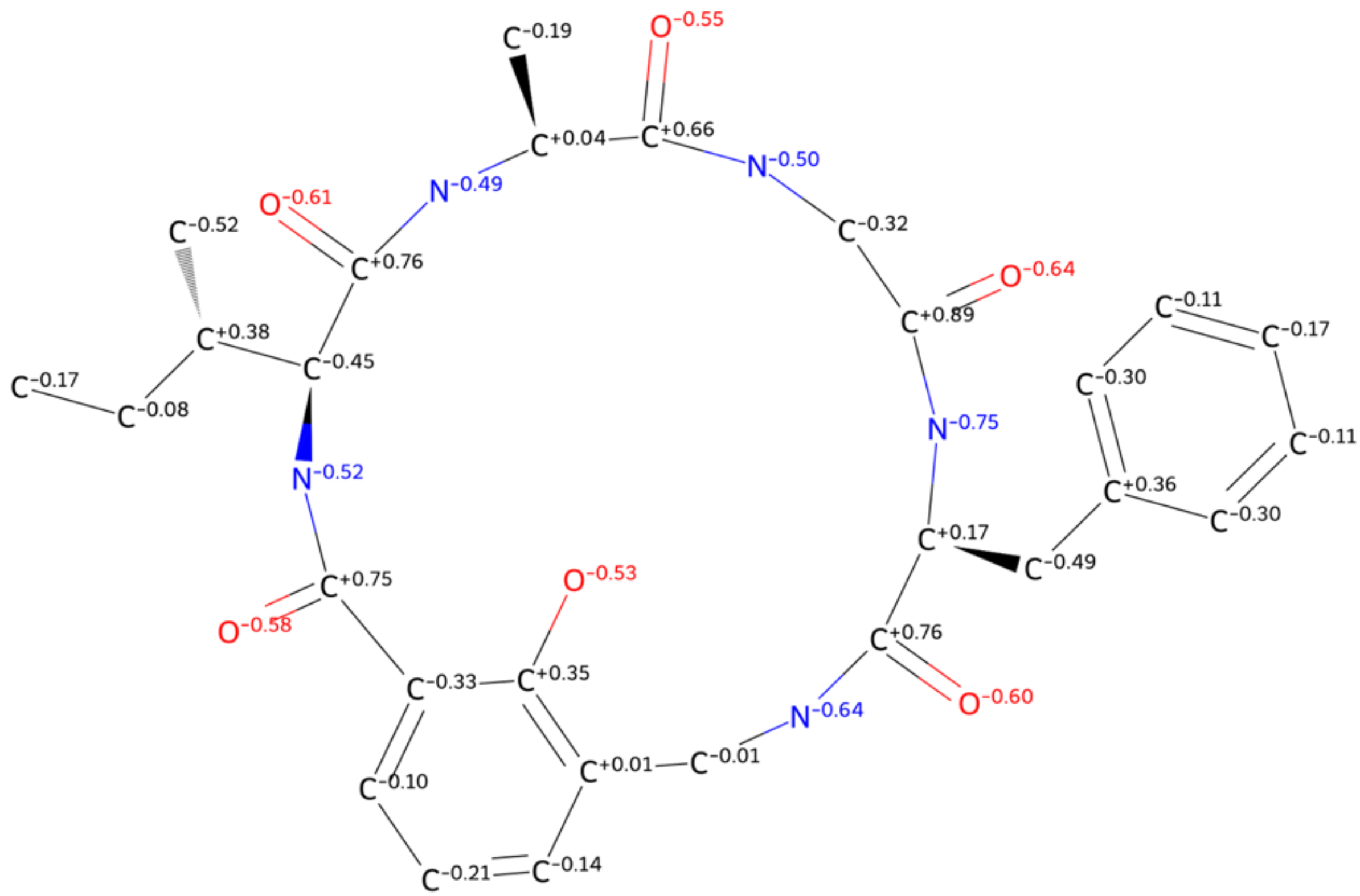

Figure S35. Lewis-structure of **CP7** with indicated RESP charges, calculated for the conformer (Conf.^{fast} 1, dark cyan in the main text) using Gaussian16 at the HF/6-31G* level of theory.

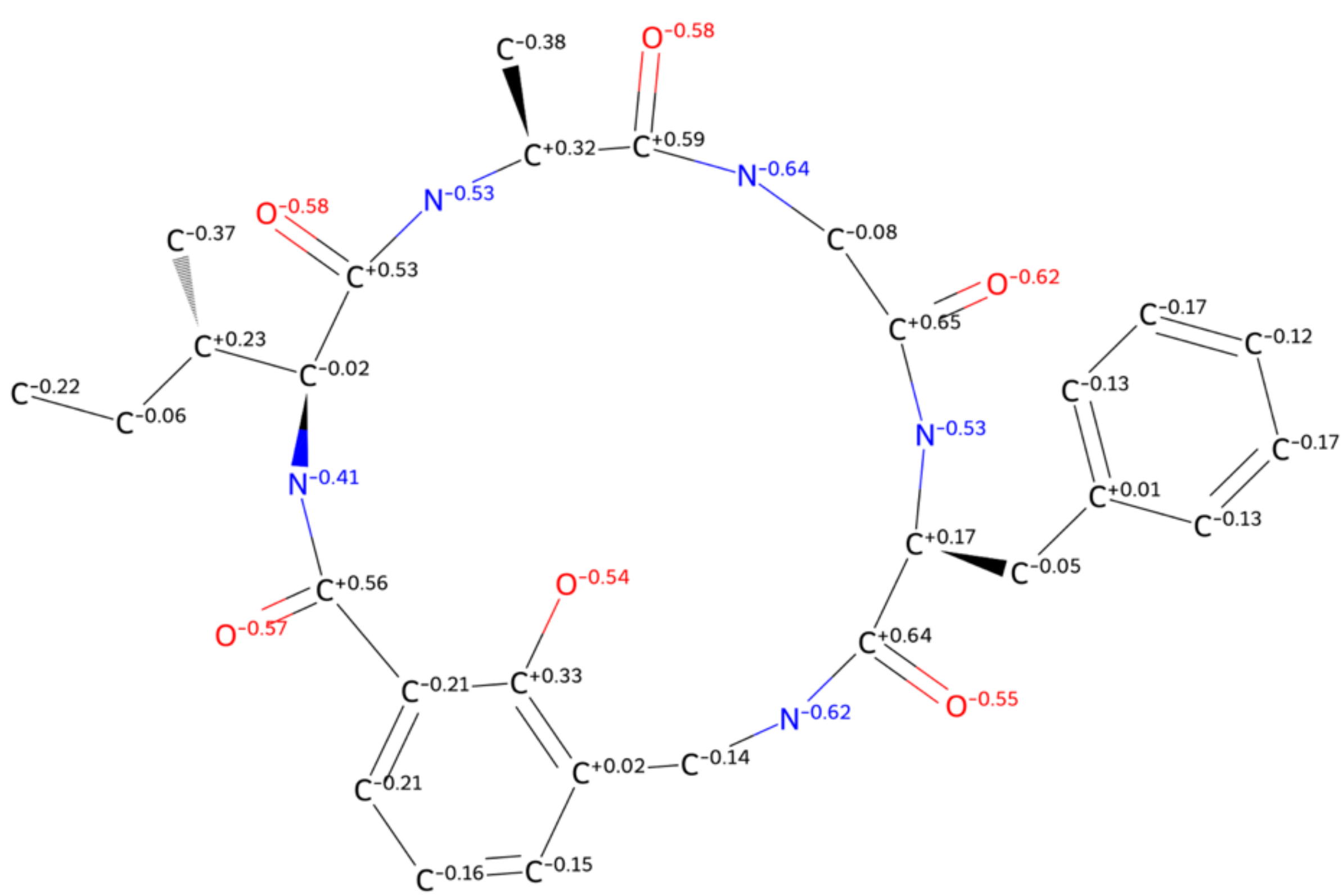

Figure S36. Lewis-structure of **CP7** with indicated RESP charges, calculated for the conformer (Conf.^{fast} 2, medium cyan in the main text) using Gaussian16 at the HF/6-31G* level of theory.

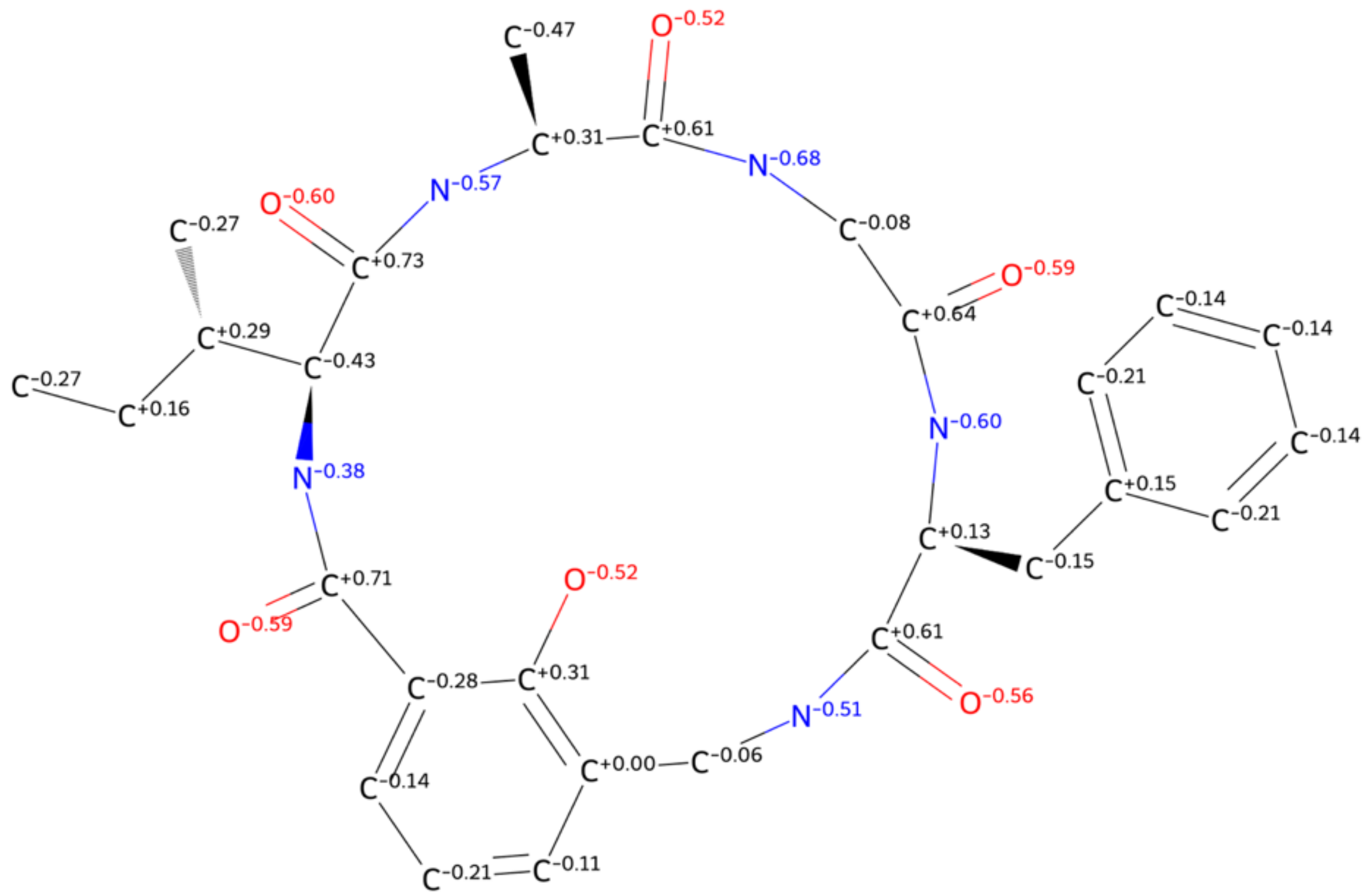

Figure S37. Lewis-structure of **CP7** with indicated RESP charges, calculated for the conformer (Conf.^{slow} 3, light cyan in the main text) using Gaussian16 at the HF/6-31G* level of theory.

SI.15.3 Unbiased simulations

The systems have been prepared all according to the procedure described above, using the force field parameters derived for **CP7** of the different conformers as described above. In each case 1 µs, 5µs for the parameter from Conf.^{fast} 1, of total simulation time in DMSO at 300 K and 1 bar were taken and the ϕ and ψ angles of the residues 1Ile, 2Ala, 3Gly and 4Phe have been plotted in Ramachandran plots. From this one can see that the differences in the angle distribution are small for the parameters derived from Conf.^{fast} 1 (dark cyan) and Conf.^{fast} 2 (medium cyan). Both show overall similar populations of the angles. In contrast the angles obtained from the simulation with the parameters derived from Conf.^{slow} 3 (light cyan) show clearly a different population distribution at least for 1Ile and 3Gly. Notably we see that the angles of the Conf.^{slow} 3 (light cyan) for 3Gly are not sampled in any of the simulations, not even when taking the conformer as the starting structure and for the parametrization.

We therefore decided to take the RESP-charges derived from the conformer with the highest population in our NMR/DFT approach Conf.^{fast} 1. Furthermore, we concluded that the reason for the lack of 3Gly angles of Conf.^{slow} 3 was due to limited simulation time and not due to the force field parameters.

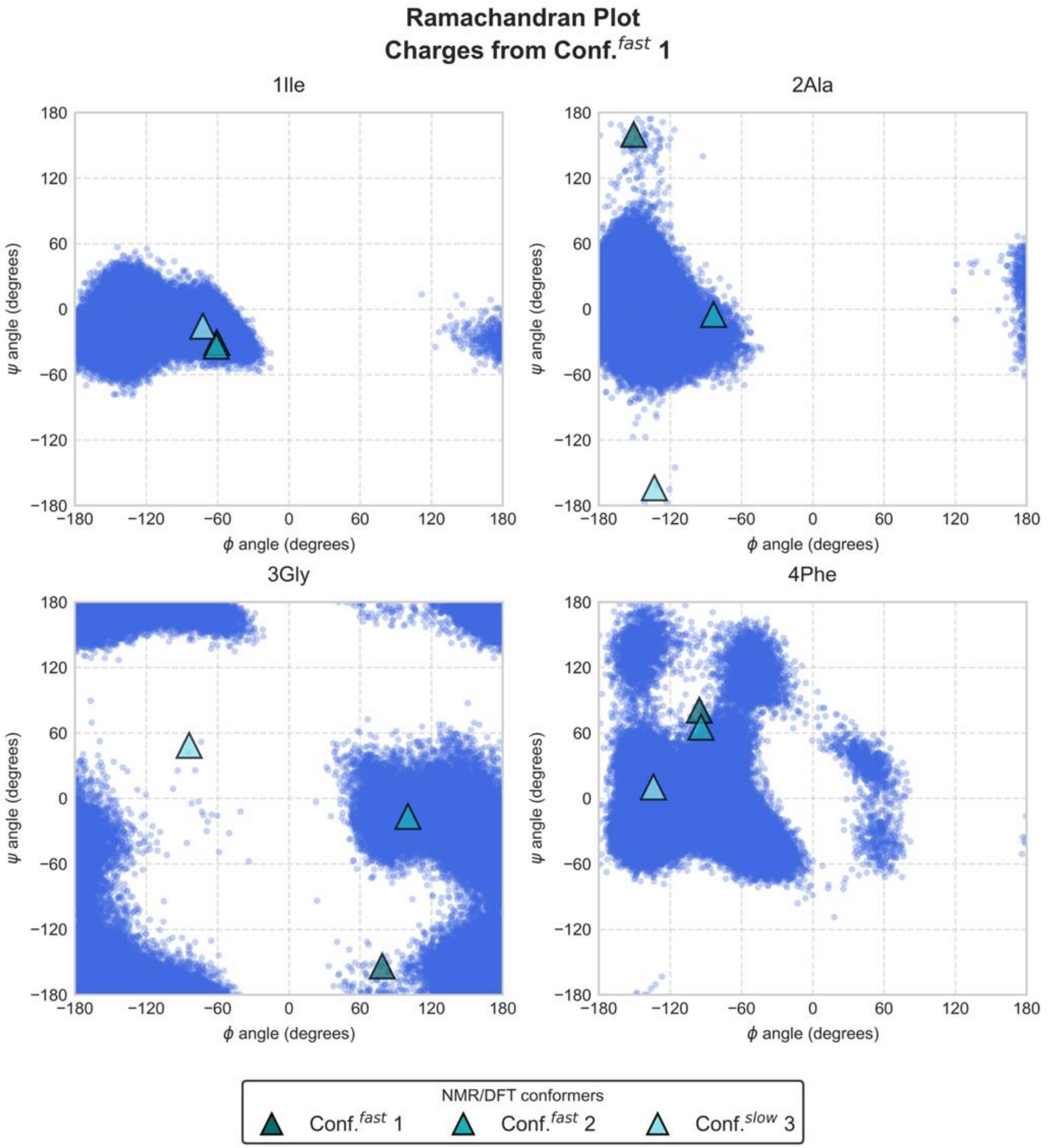

Figure S38. Ramachandran plot of the residues 1Ile, 2Ala, 3Gly and 4Phe from an unbiased MD simulation of **CP7** in DMSO at 300 K and 1 bar using the GAFF force field. Parameters for **CP7** have been calculated based on the structure of the major conformer Conf.^{fast} 1 (dark cyan). The data was taken from 5x1µs simulations.

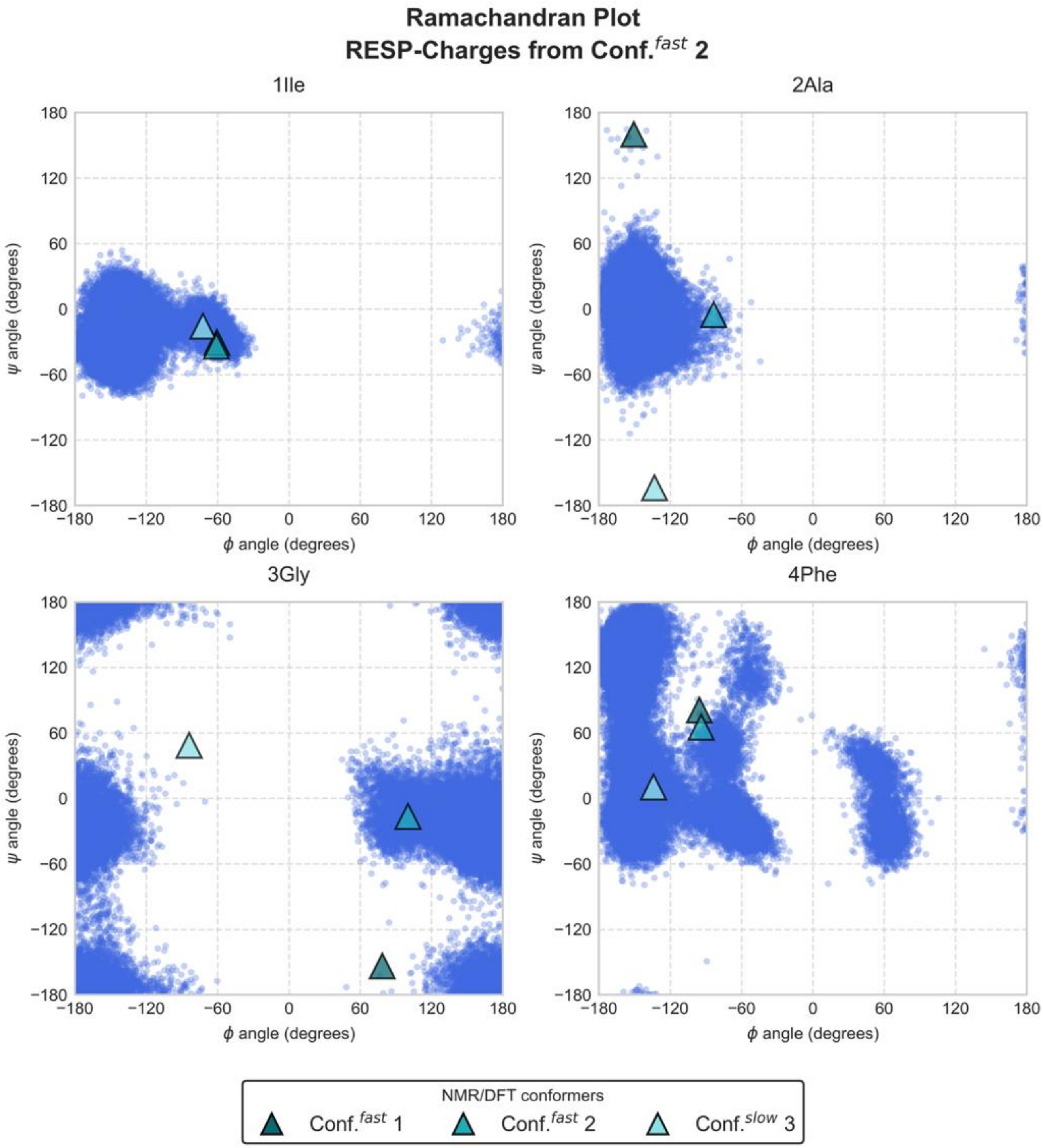

Figure S39. Ramachandran plot of the residues 1Ile, 2Ala, 3Gly and 4Phe from an unbiased MD simulation of **CP7** in DMSO at 300 K and 1 bar using the GAFF force field. Parameters for **CP7** have been calculated based on the structure of the major conformer Conf.^{fast} 2 (medium cyan).

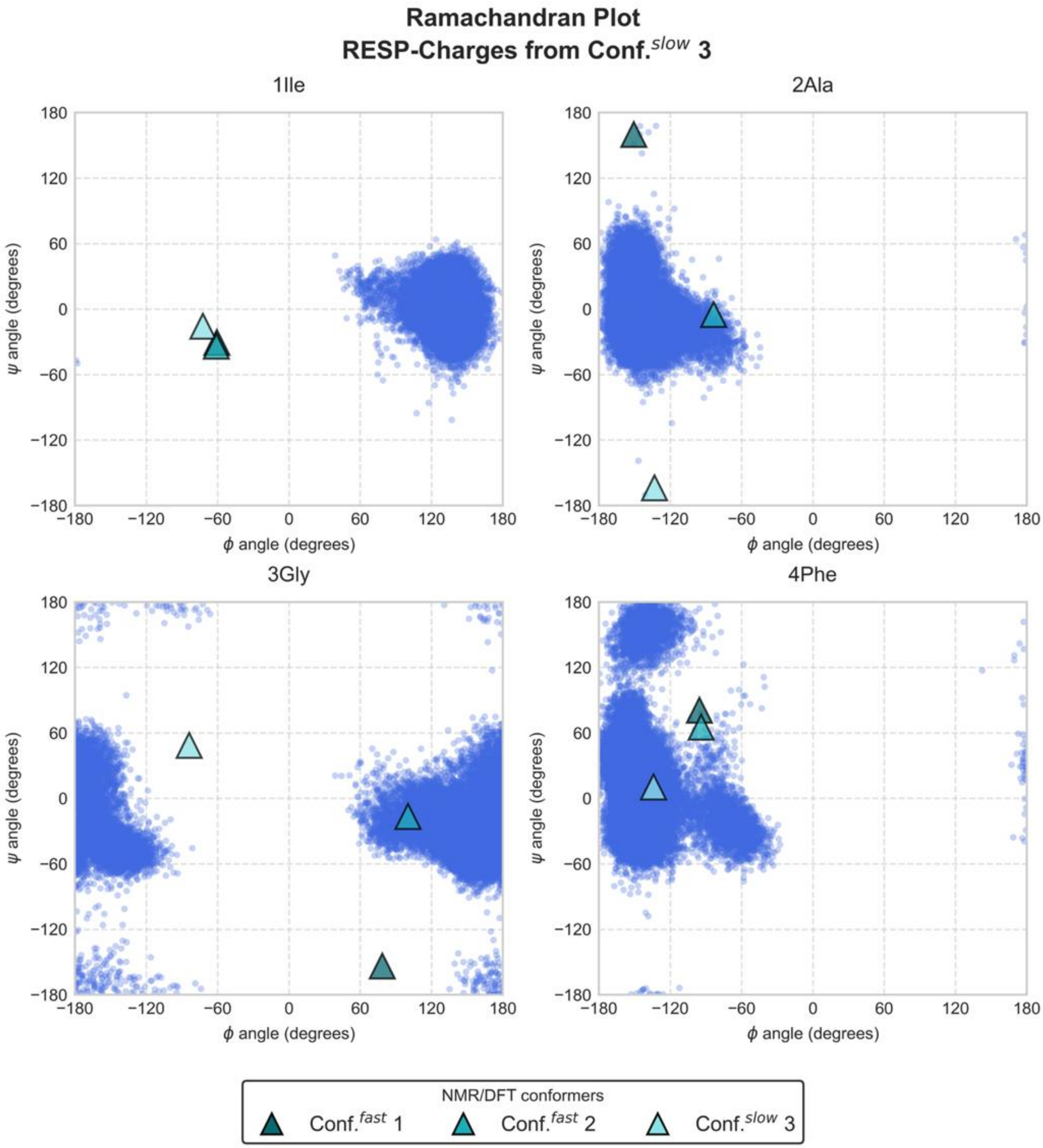

Figure S40. Ramachandran plot of the residues 1Ile, 2Ala, 3Gly and 4Phe from an unbiased MD simulation of **CP7** in DMSO at 300 K and 1 bar using the GAFF force field. Parameters for **CP7** have been calculated based on the structure of the minor conformer Conf.^{slow} 3 (light cyan).

SI 15.4 Metadynamics simulations

Because we were unable to identify 3Gly dihedral angles similar to $Conf.^{slow}$ 3(light cyan) in any of the unbiased simulation we decided to turn to well-tempered metadynamics simulations by biasing the 3Gly dihedrals to investigate whether this pair of angles belongs to an energy minimum. For that we conducted similar simulations as described above, however we used plumed v. 2.9[47–49] to add biases to the dihedral angles. The bias was added every 500 steps (1 ps), with an initial gaussian height of 1.2 kJ/mol and an angle sigma of 0.2 radians. The bias factor was set to 8. The angles were output every 200 fs. The simulations were conducted four times with each 1 µs. Free energy plots have been generated using the plumed *sum_hills* command.

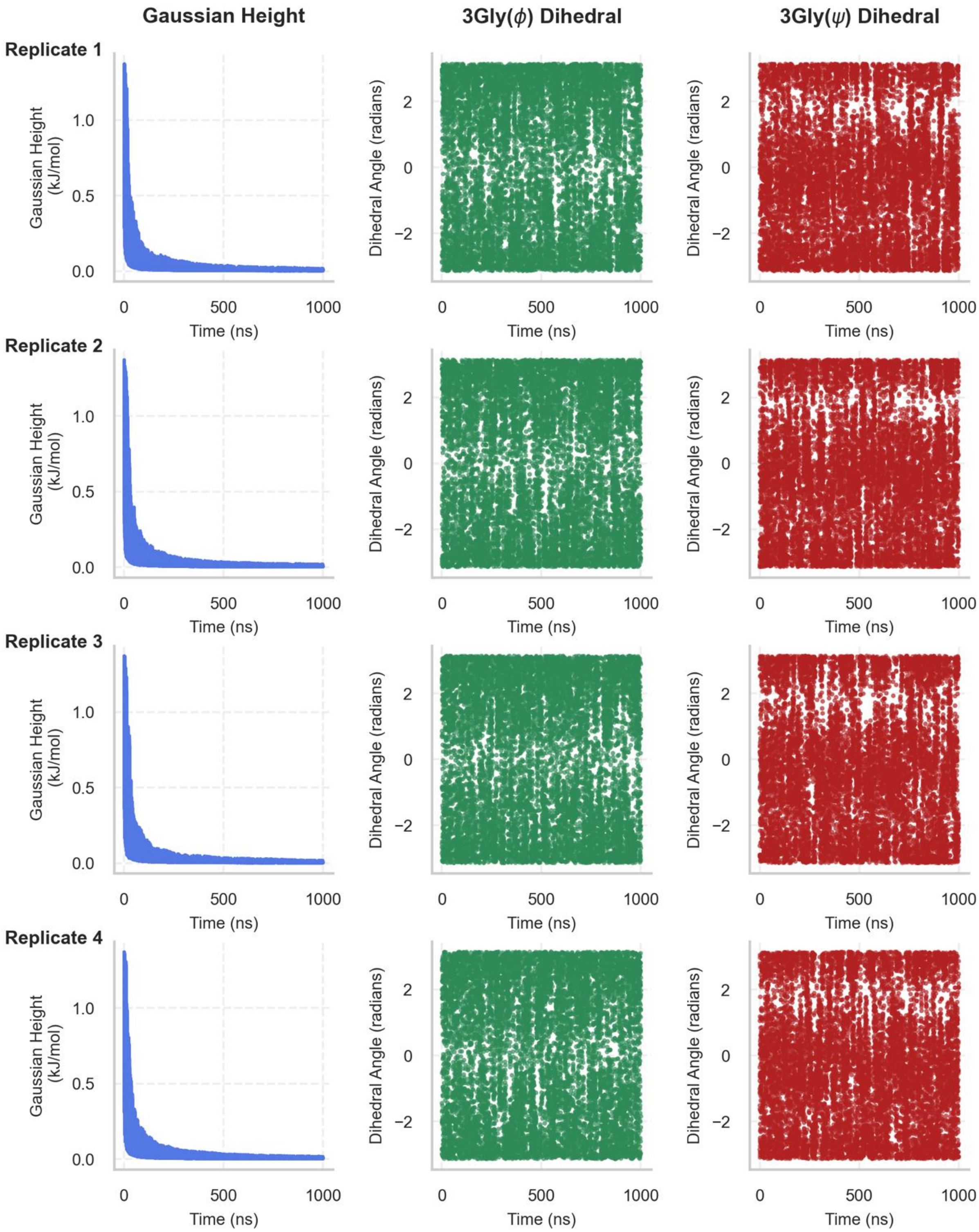

Figure S41. Distribution of added gaussian biases, 3Gly(ϕ) and 3Gly(ψ) of **CP7** in DMSO for the 4x1 μs metadynamic simulation at 300 K and 1 bar using the GAFF force field.

SI.16 Experimental spectra

All raw spectra are deposited on Zenodo in Bruker format: 10.5281/zenodo.19909872

SI.16.1 Spectra for CP1

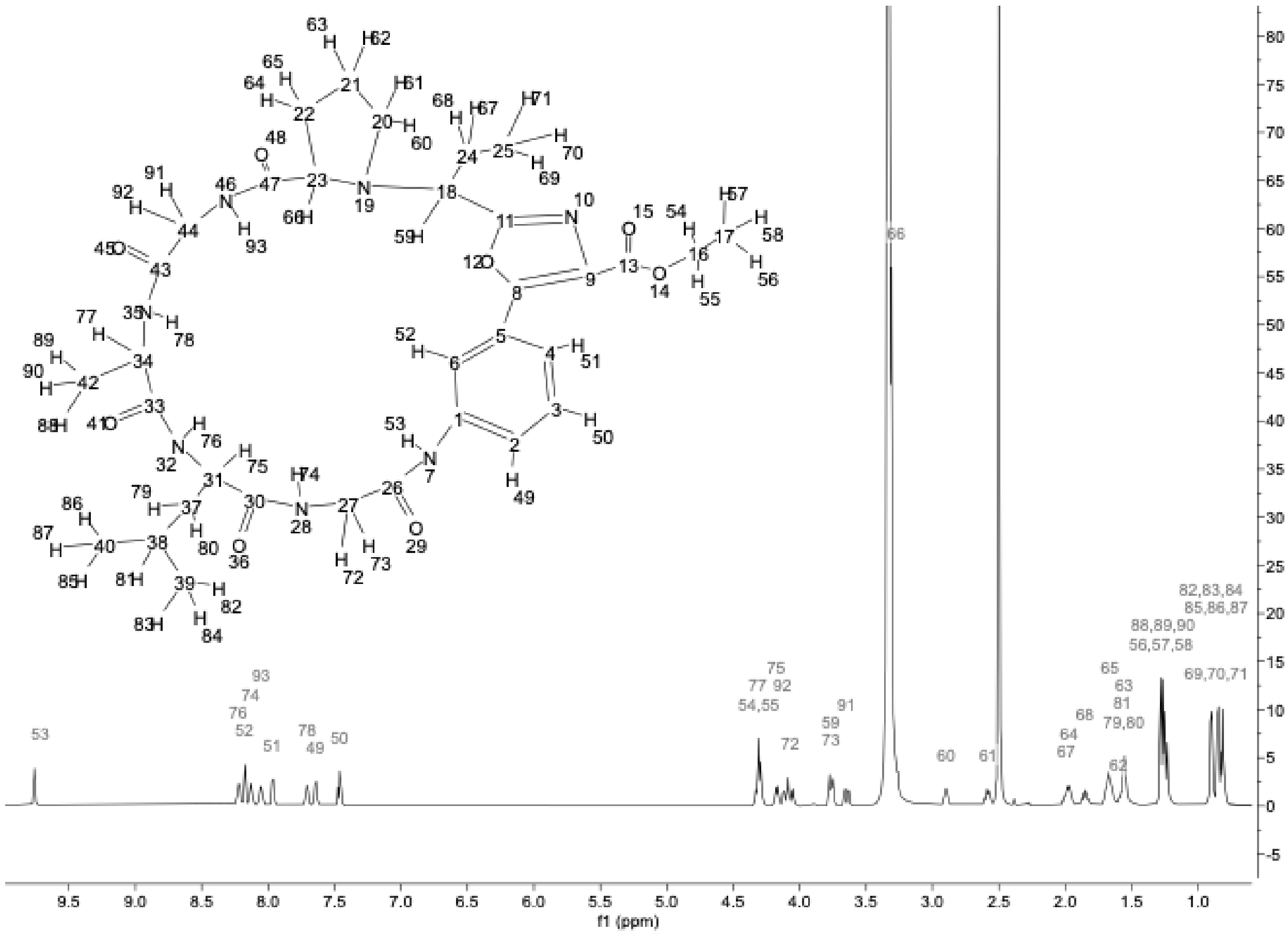

Figure S42. Proton spectrum of cyclic peptide **CP1** at 300 K in DMSO-*d6* at 600 MHz. NS =8, d1= 5 s, AQ = 1.6 s, SW = 16.6 ppm, TD = 32k.

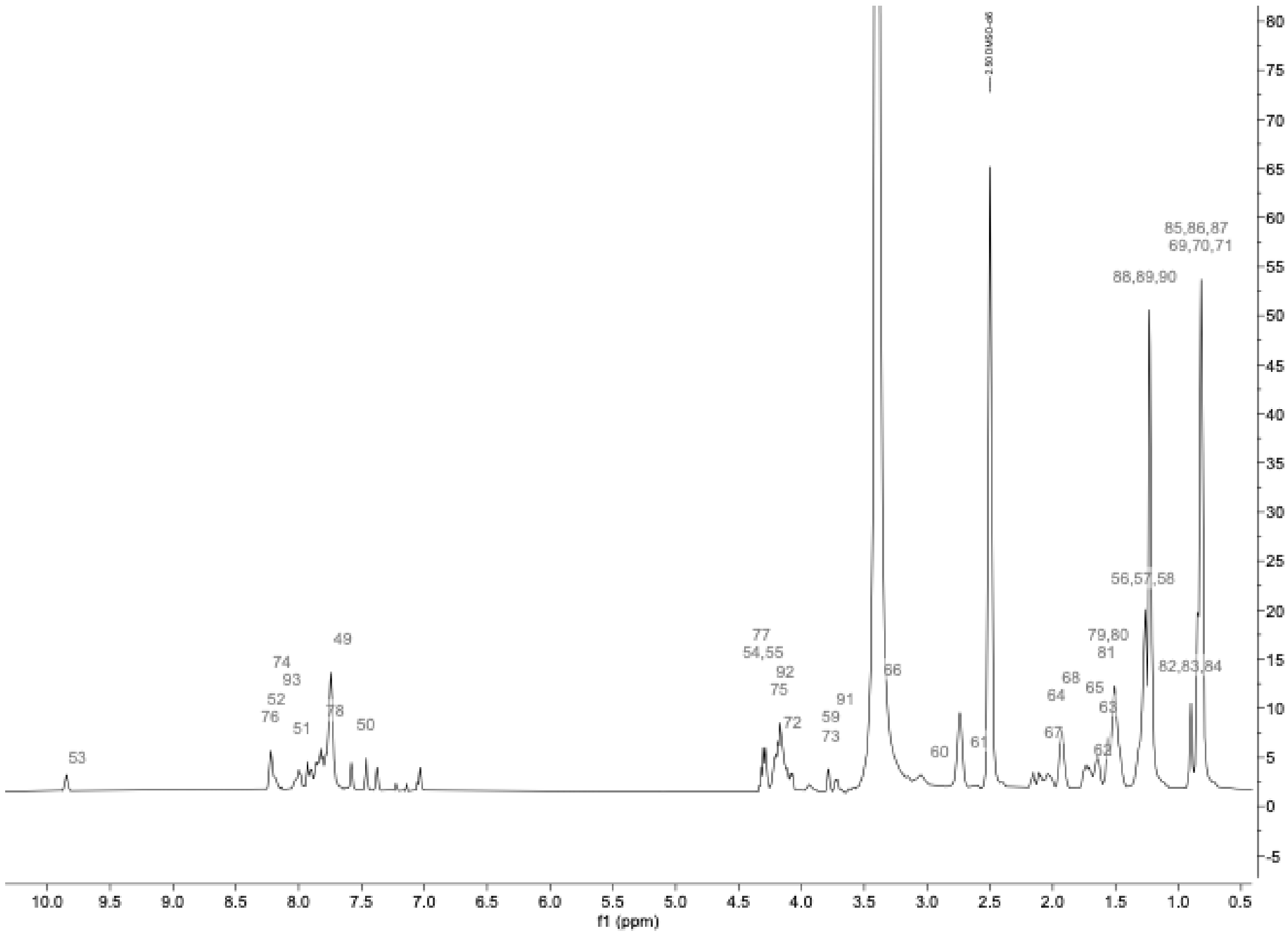

Figure S43. Proton spectrum of cyclic peptide **CP1** at 300 K in the anisotropic phase DMSO-*d6*/OPA at 600 MHz. NS =8, d1= 5 s, AQ = 1.6 s, SW = 16.6 ppm, TD = 32k.

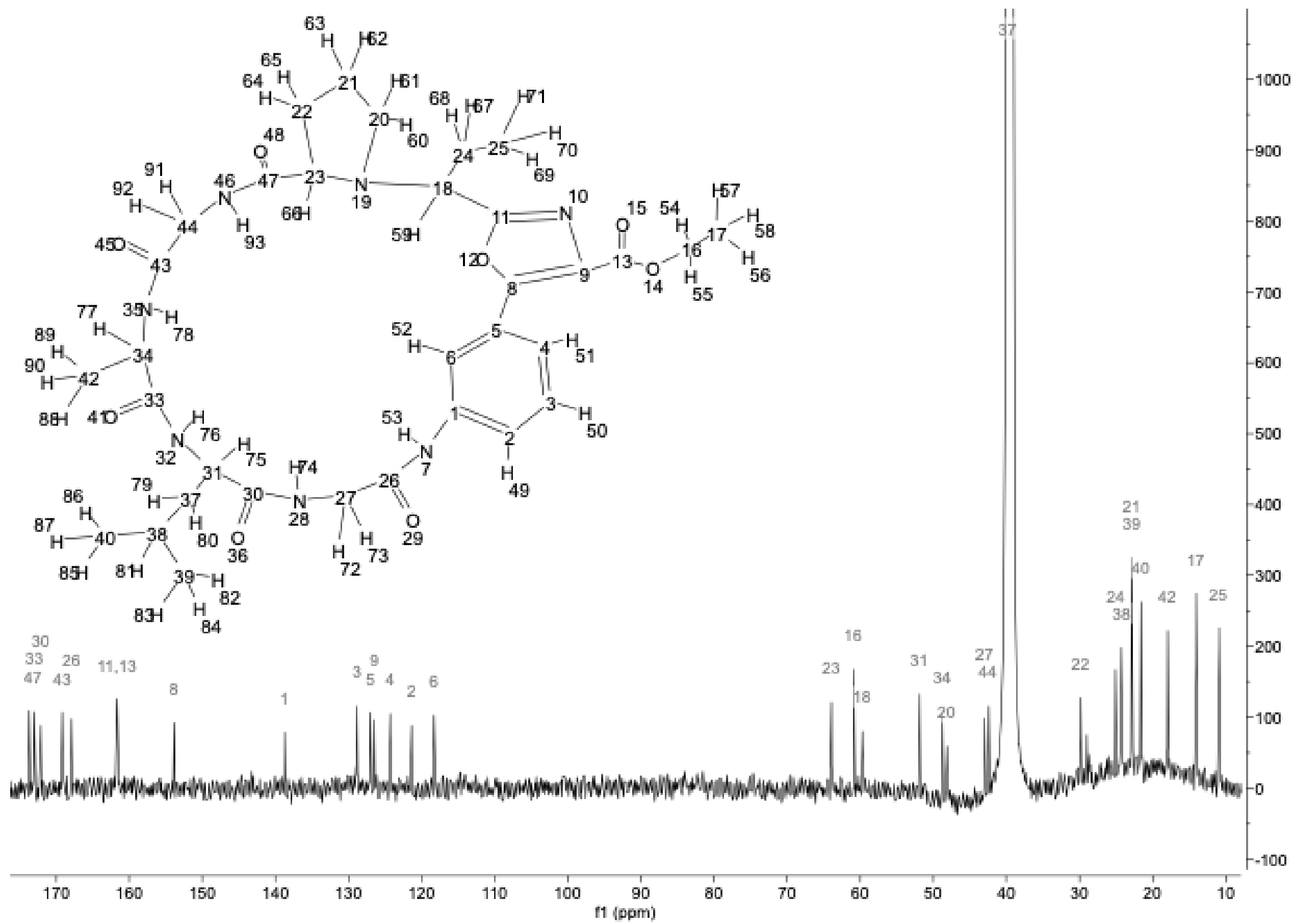

Figure S44. Carbon spectrum of cyclic peptide **CP1** at 300 K in *DMSO-d6* at 600 MHz. NS =1024 (TD0 =32), d1= 2.5 s, AQ = 2.0 s, SW = 212.4 ppm, TD = 128k, 5.0 Hz LB.

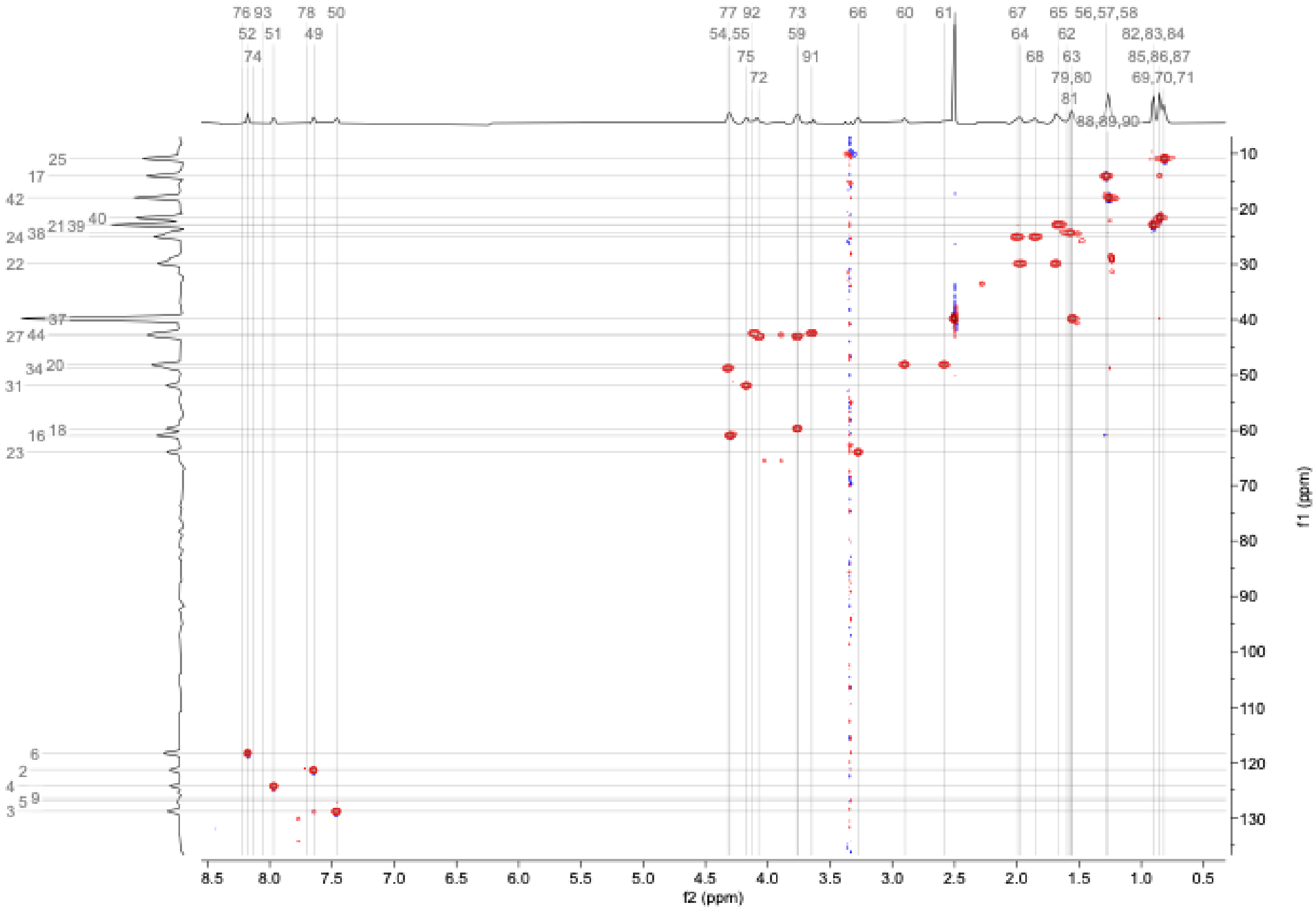

Figure S45. HSQC spectrum of cyclic peptide **CP1** at 300 K in DMSO-*d6* at 600 MHz. NS=32, d1 = 1.3 s, SW 12 (F2) x 160 (F1), TD 2k x 512, echo-antiecho.

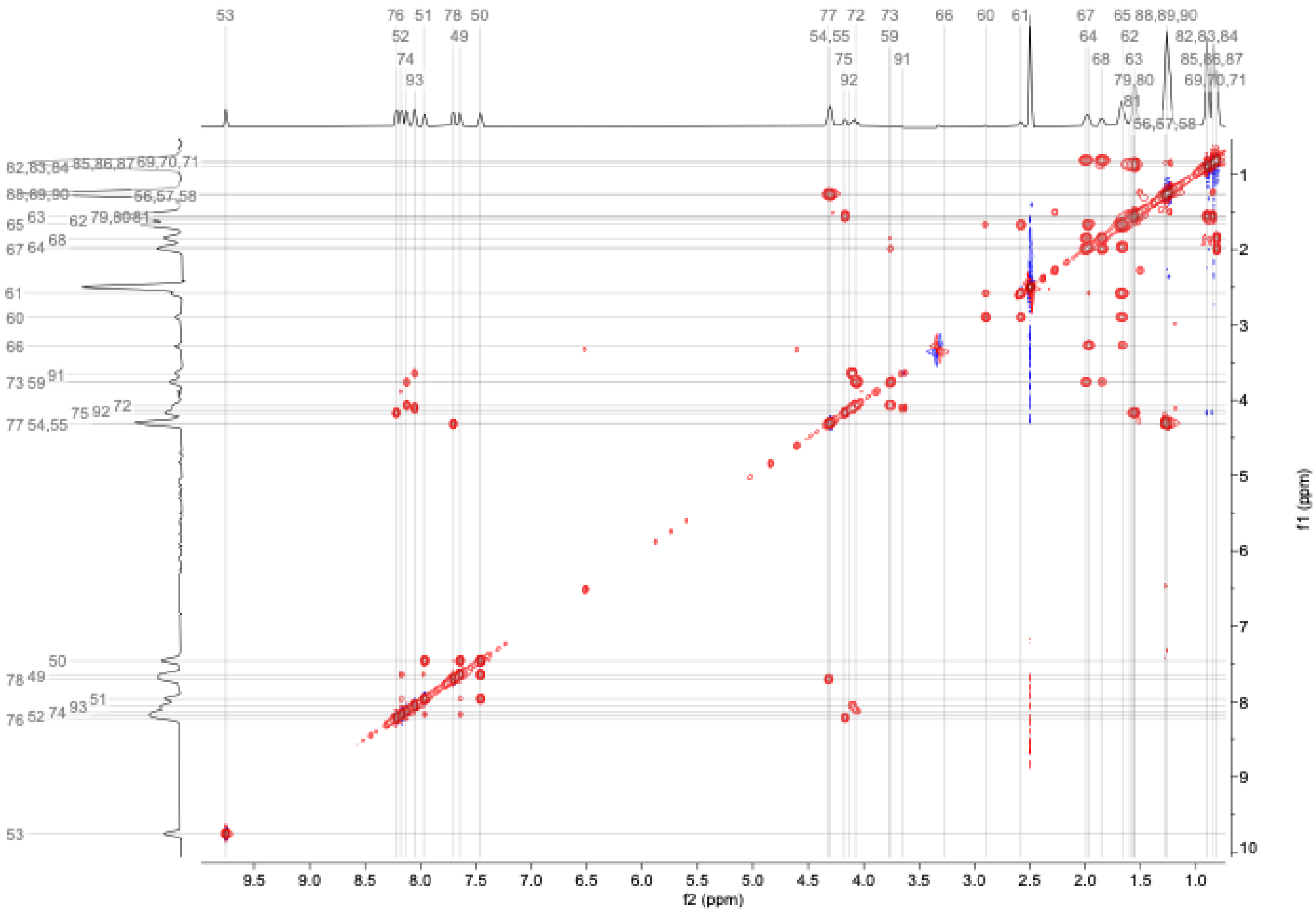

Figure S46. CLIP-COSY spectrum of cyclic peptide **CP1** at 300 K in DMSO-*d6* at 600 MHz.NS=16, d1= 1.3s, SW 16 (F2) x 16 (F1), TD 2k x 512, States-TPPI.

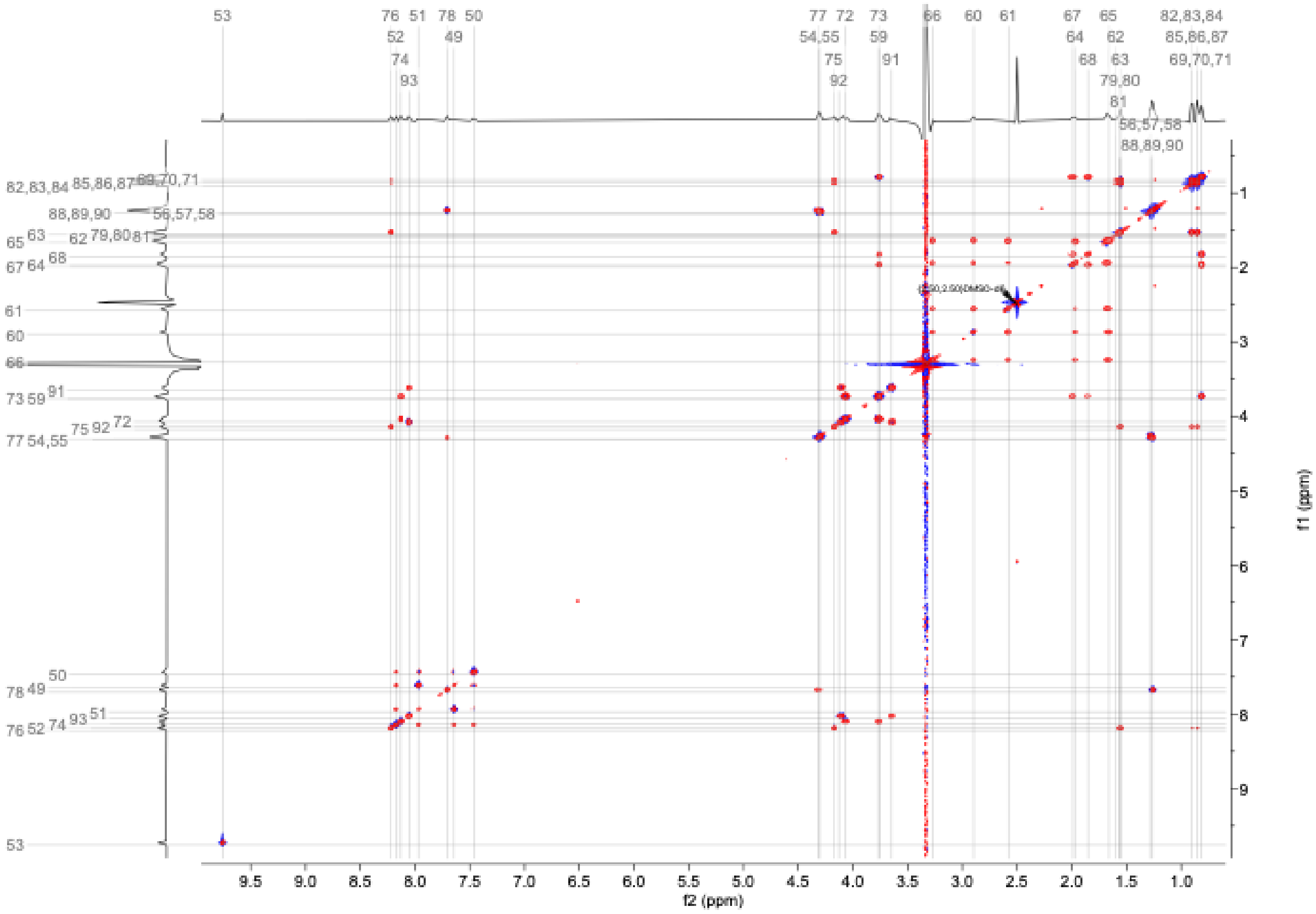

Figure S47. TOCSY spectrum of cyclic peptide **CP1** at 300 K in DMSO-*d6* at 600 MHz. NS=16, d1= 1.3s, SW 14 (F2) x 14 (F1), TD 2k x 1k, States-TPPI, 80 ms mixing time.

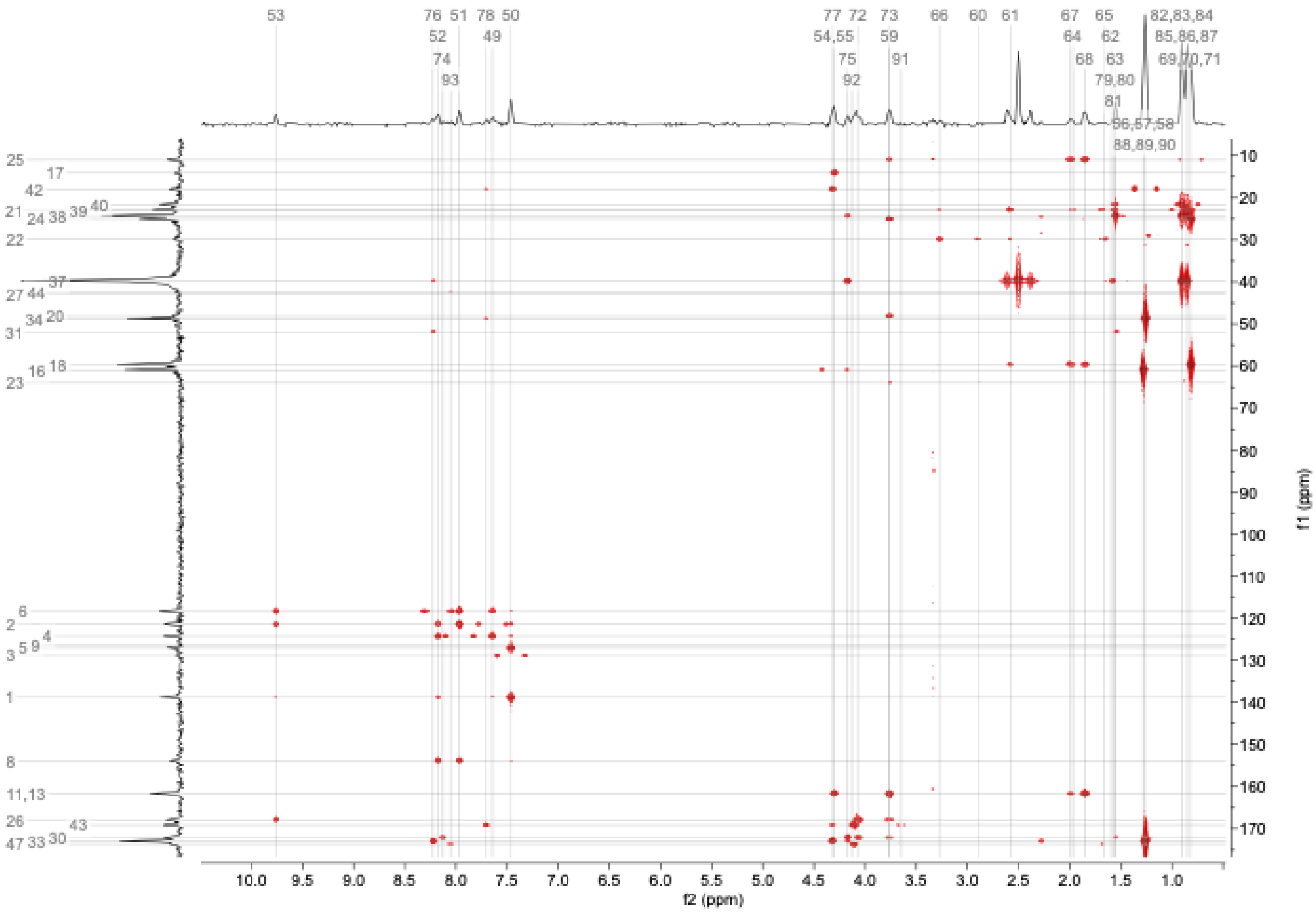

Figure S48. HMBC spectrum of cyclic peptide **CP1** at 300 K in DMSO-*d6* at 600 MHz. NS=32, d1= 1.3s, SW 16.6 (F2) x 207 (F1), TD 2k x 1k, delay for long range coupling 70 ms, echo-antiecho.

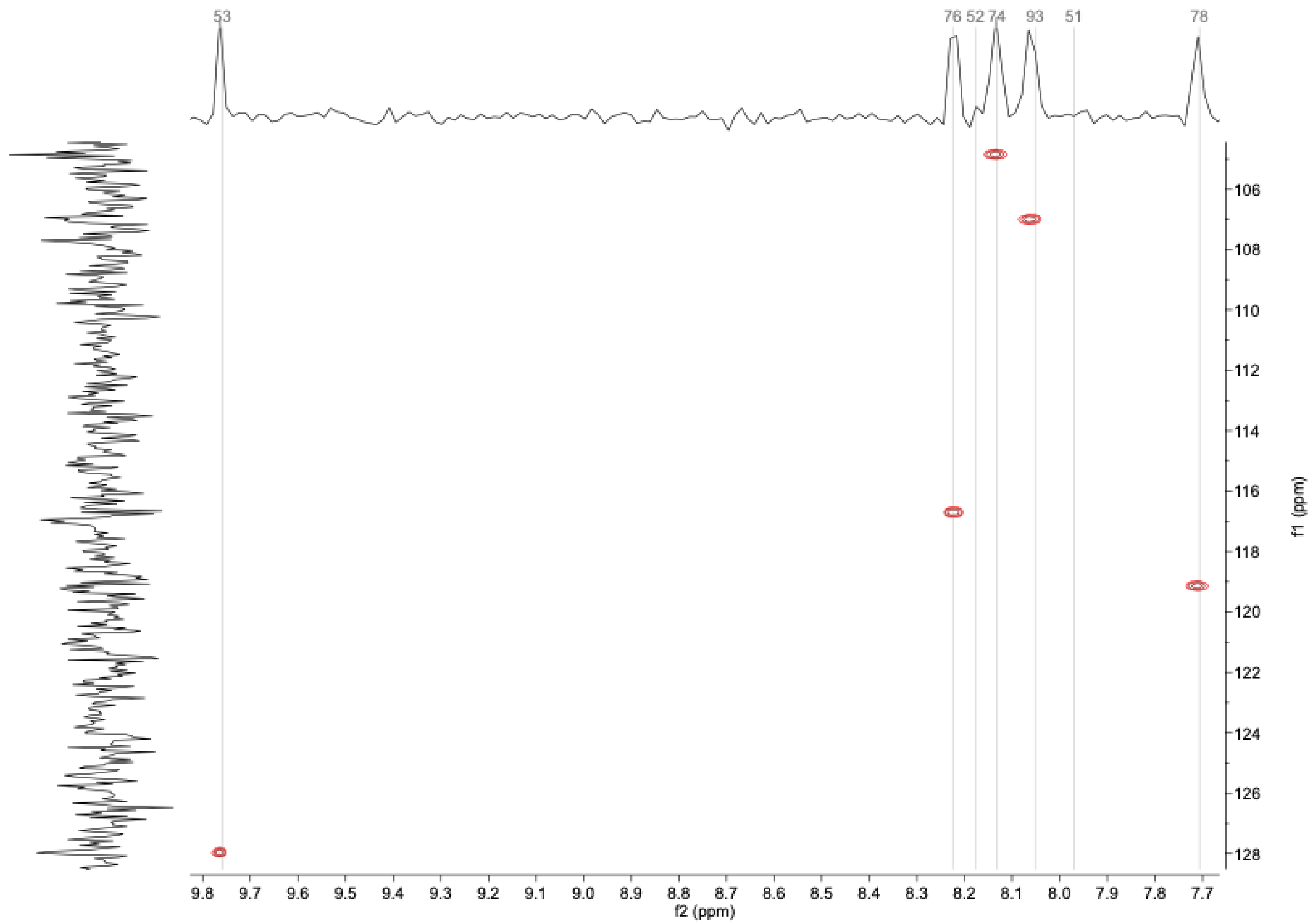

Figure S49. ^{15}N-HSQC spectrum of cyclic peptide **CP1** at 300 K in DMSO-*d6* at 600 MHz. NS=32, d1= 1.3s, SW 14 (F2) x 49 (F1), TD 2k x 512, States-TPPI, WaterGATE.

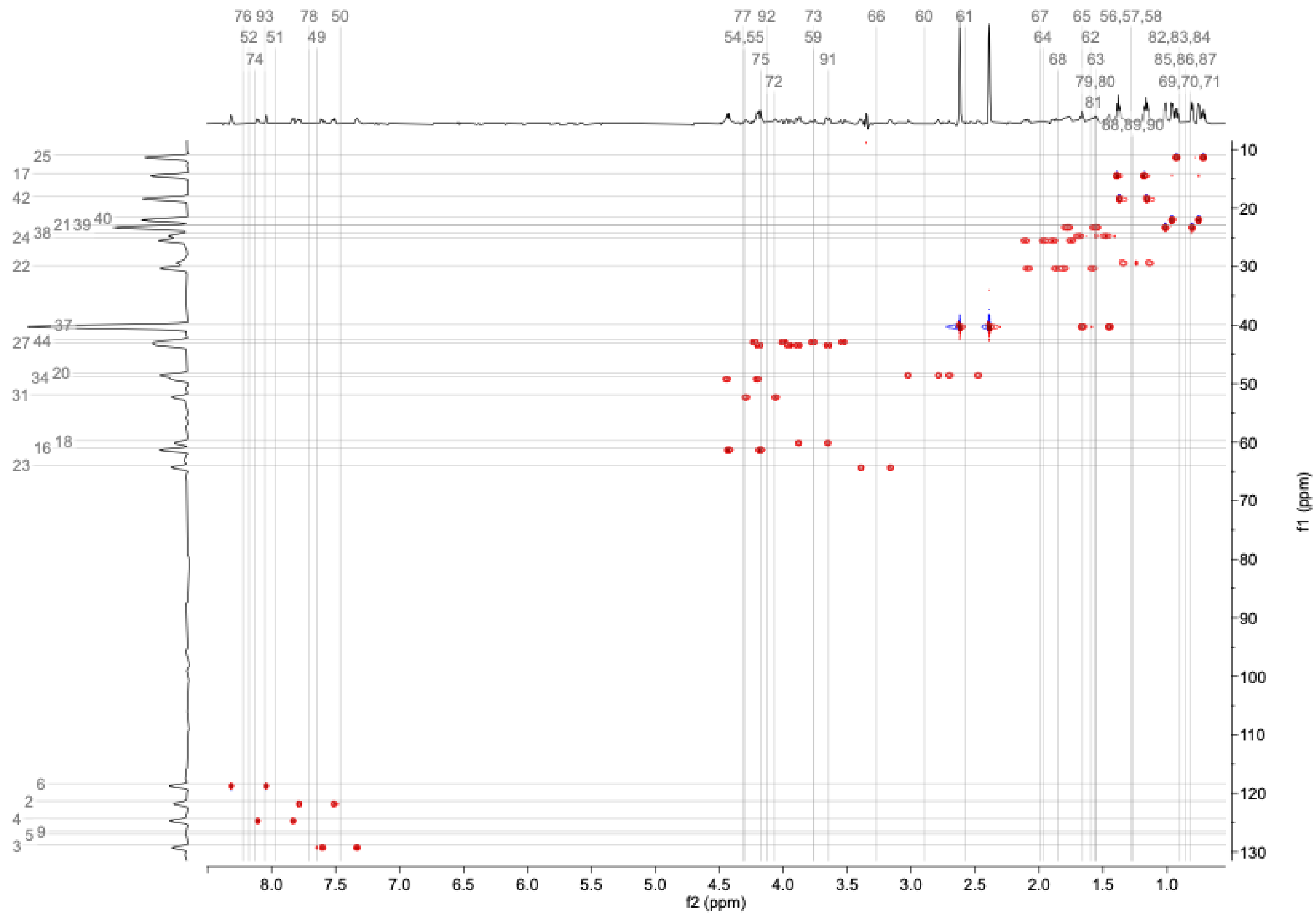

Figure S50. CLIP-HSQC spectrum of cyclic peptide **CP1** at 300 K in DMSO-*d6* at 600 MHz. NS=72, d1 = 1.3 s, SW 11 (F2) x 130 (F1), TD 4k x 512, echo-antiecho, coupling in F2.

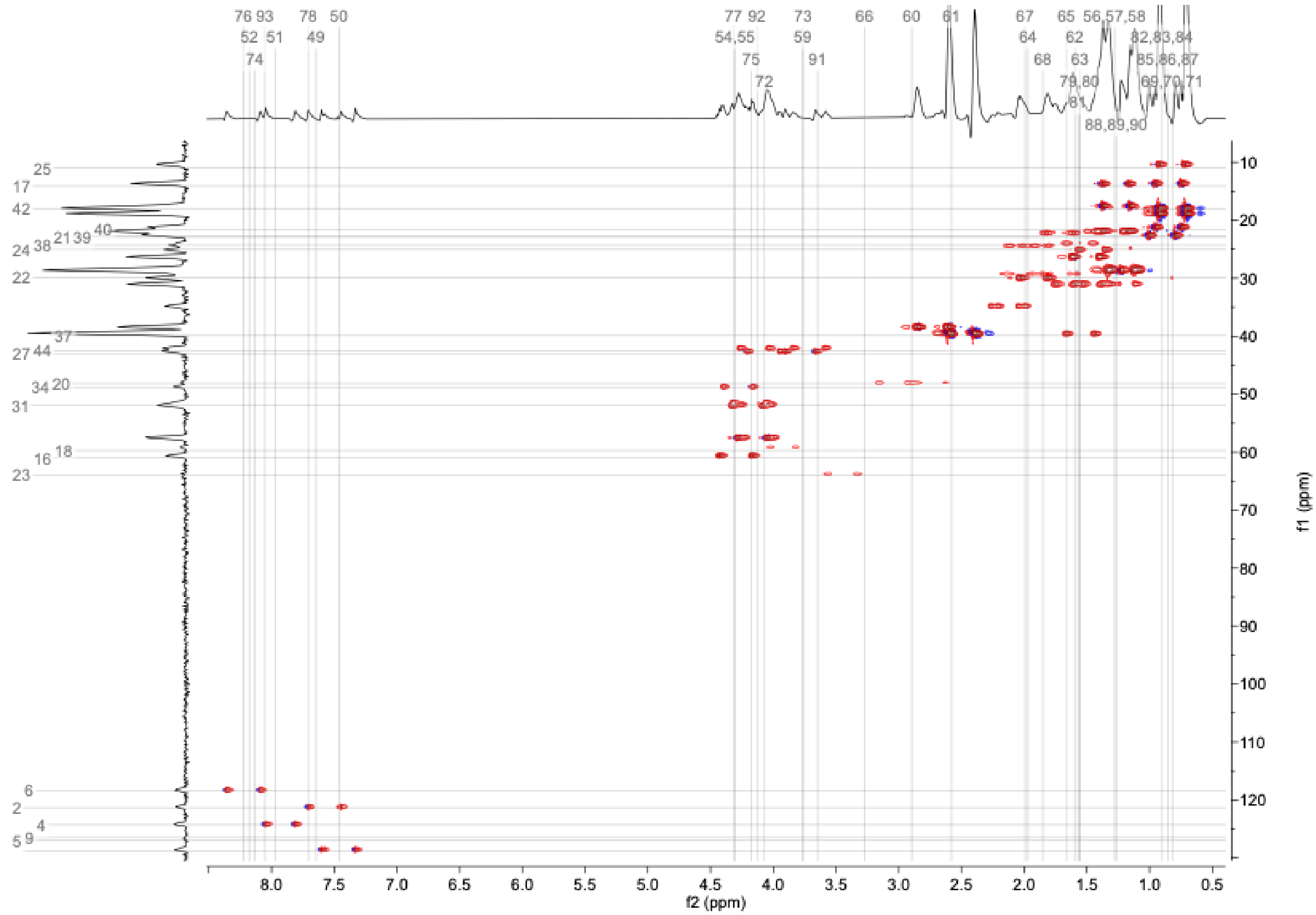

Figure S51. CLIP-HSQC spectrum of cyclic peptide **CP1** at 300 K in DMSO-*d6*/OPA at 600 MHz. NS=72, d1 = 1.3 s, SW 11 (F2) x 130 (F1), TD 4k x 512, echo-antiecho, coupling in F2.

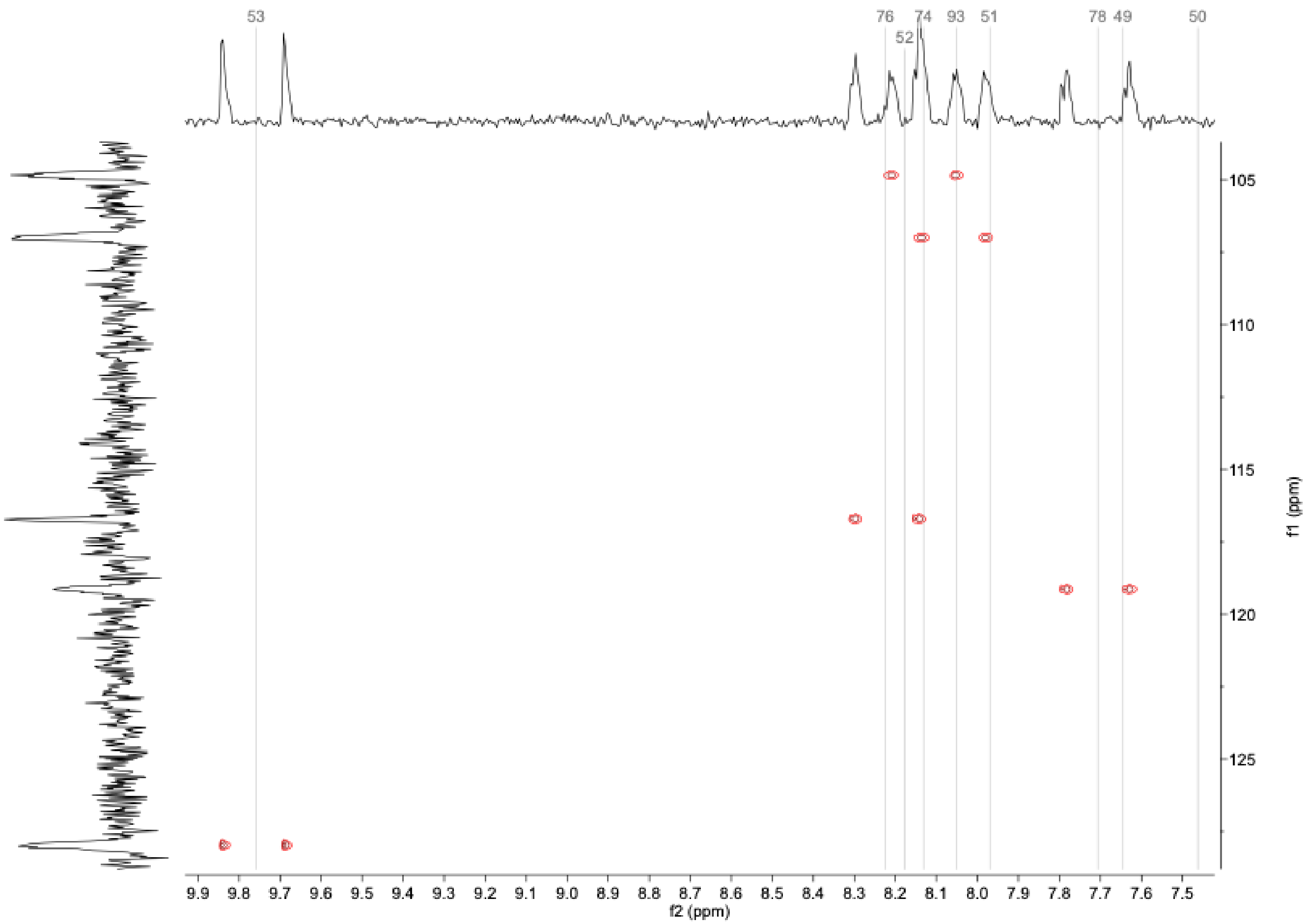

Figure S52. ^{15}N-CLIP-HSQC spectrum of cyclic peptide **CP1** at 300 K in DMSO-*d6* at 600 MHz. NS=72, d1 = 1.3 s, SW 4 (F2) x 30 (F1), TD 2k x 256, echo-antiecho, coupling in F2. WaterGATE.

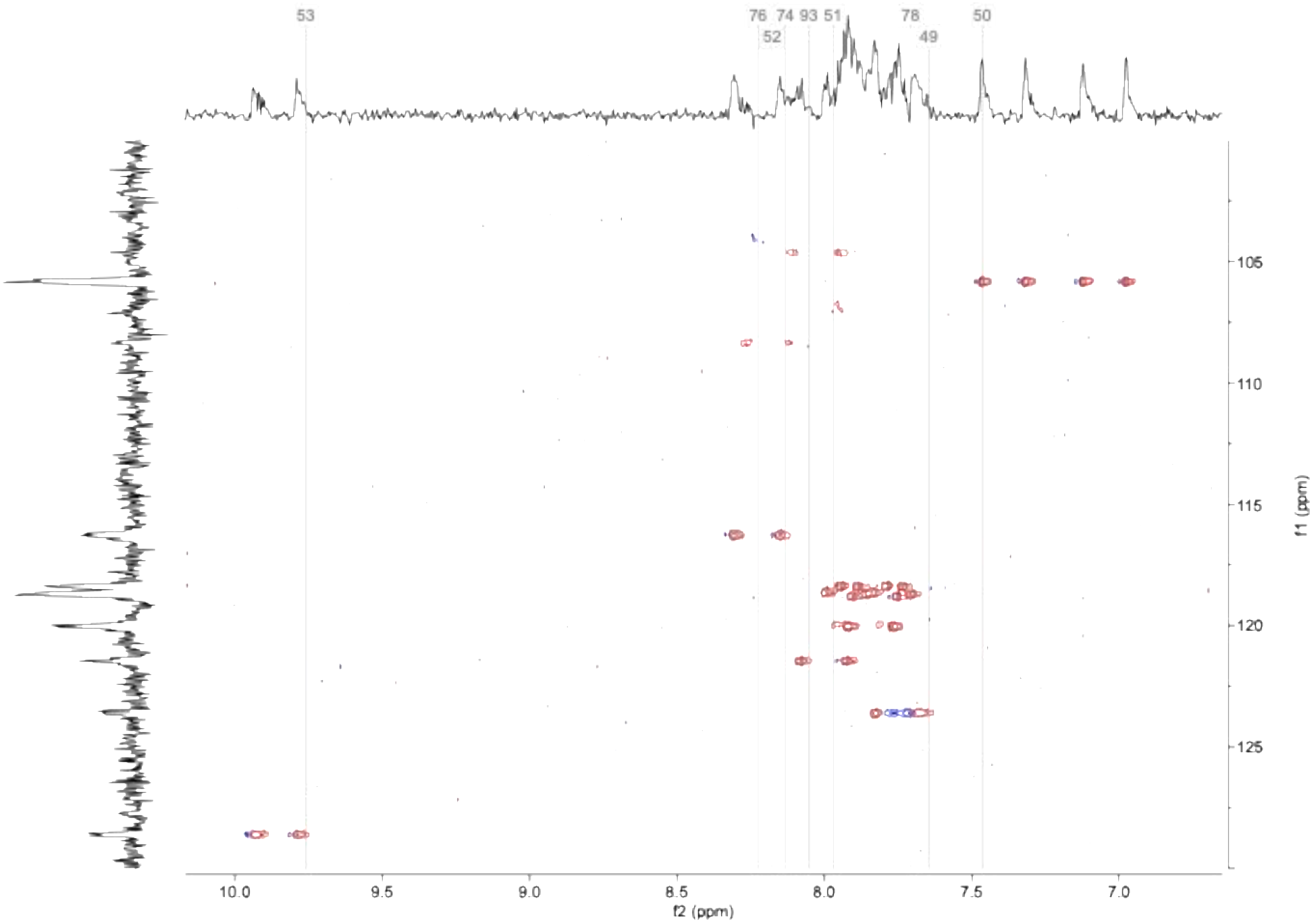

Figure S53. ^{15}N-CLIP-HSQC spectrum of cyclic peptide **CP1** at 300 K in DMSO-*d6*/OPA at 600 MHz. NS=72, d1 = 1.3 s, SW 11 (F2) x 130 (F1), TD 4k x 512, echo-antiecho, coupling in F2.

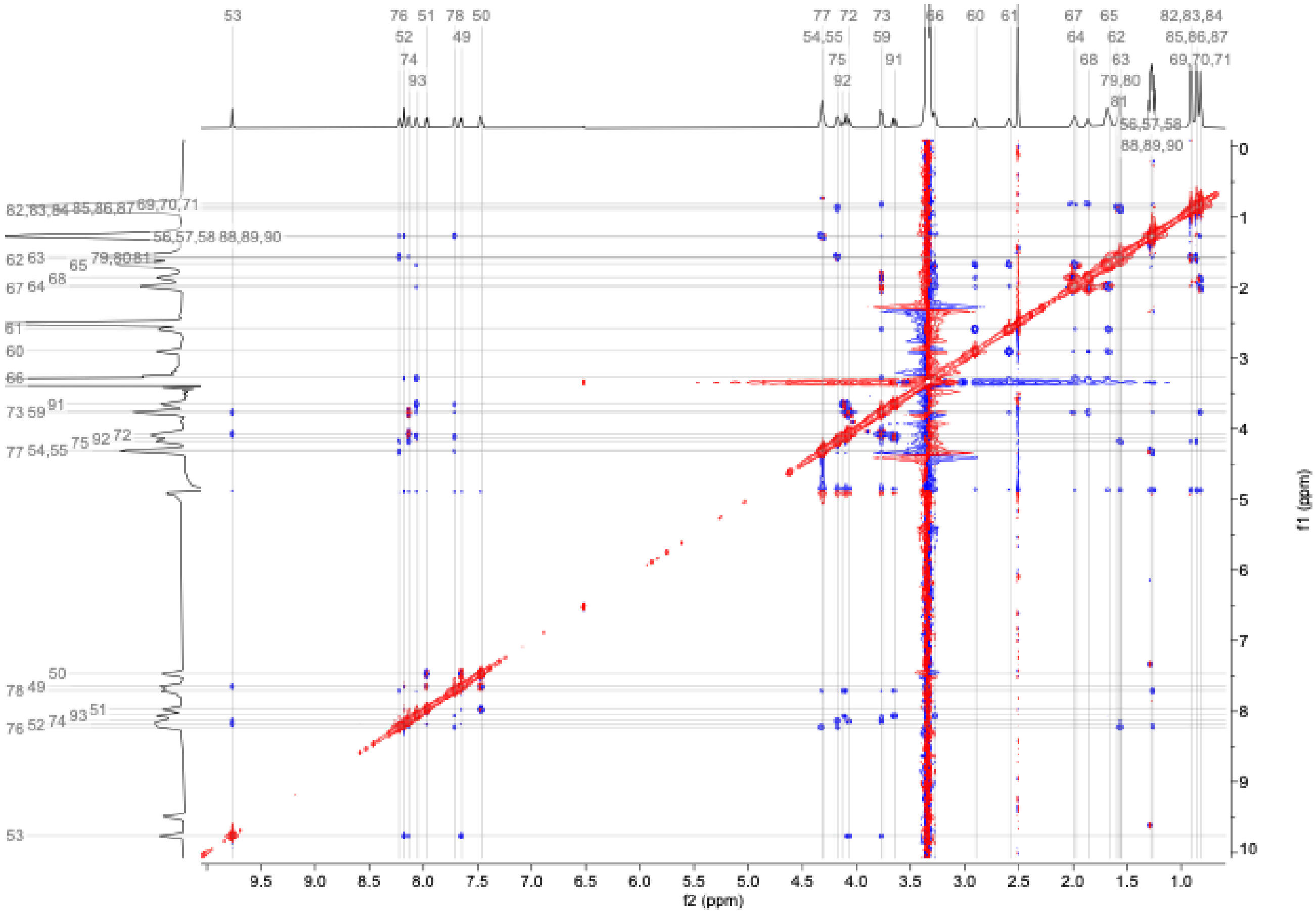

Figure S54. EASY-ROESY spectrum of cyclic peptide **CP1** at 300 K in DMSO-*d6* at 600 MHz. NS=24, d1 = 1.3 s, SW 11.9 (F2) x 11.9 (F1), TD 4k x 512, States-TPPI, mixing time = 80 ms..

SI.16.2 Spectra for CP2

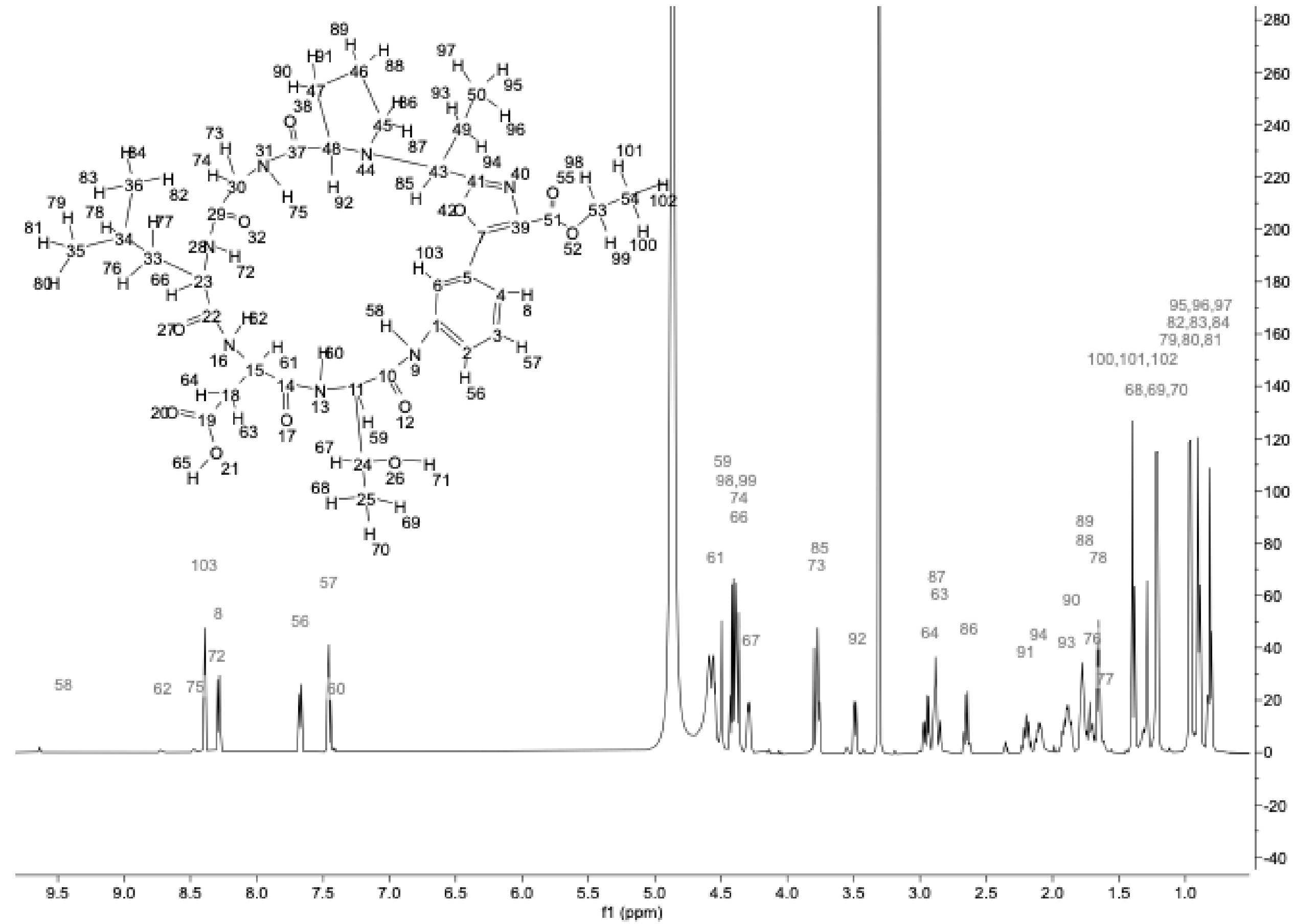

Figure S55. Proton spectrum of cyclic peptide **CP2** at 300 K in methanol-*d4* at 600 MHz. NS =16, d1= 5 s, AQ = 0.4 s, SW = 16.6 ppm, TD = 8k.

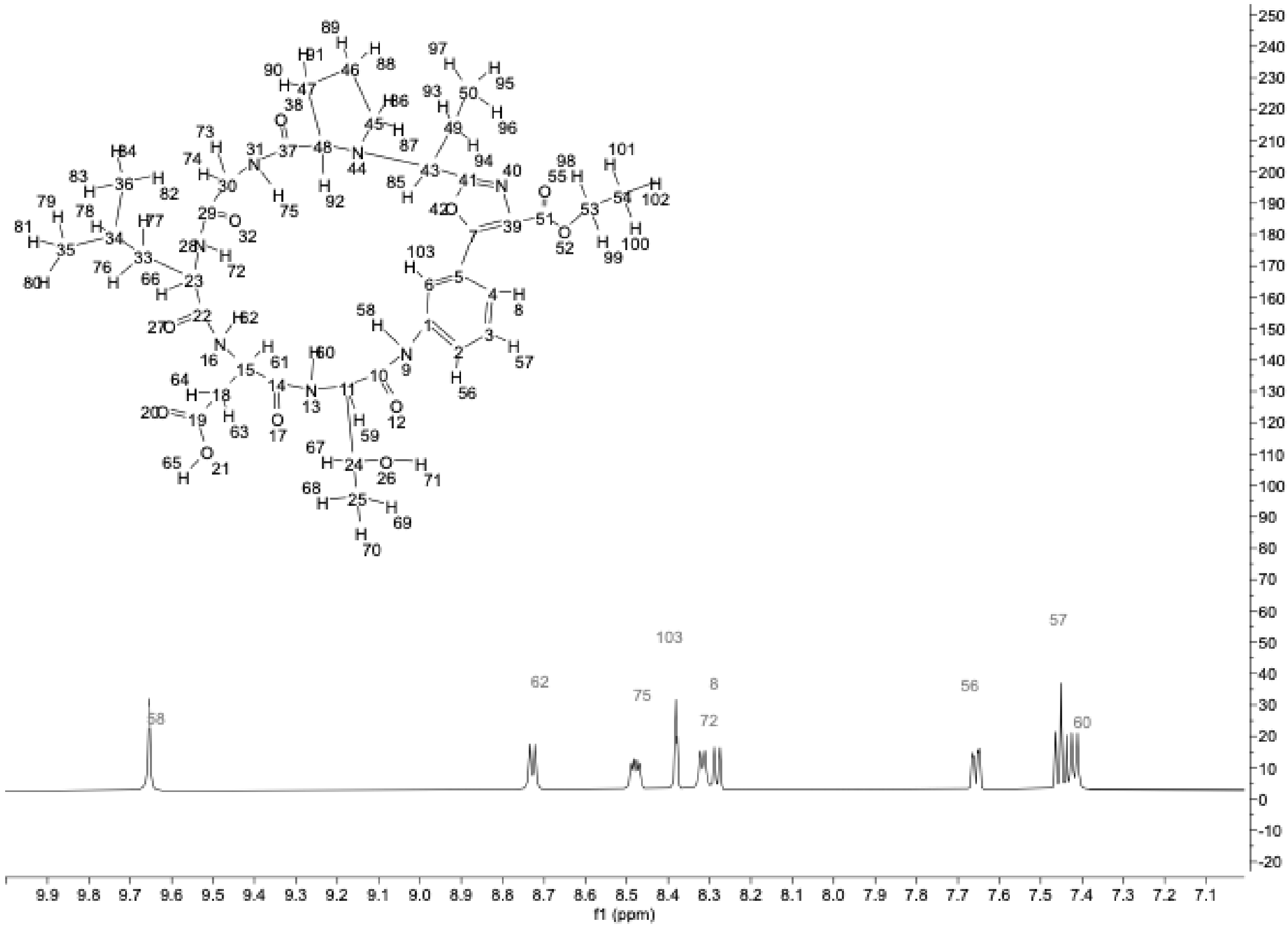

Figure S56. Excerpt of proton spectrum of amide proton region [7.00 ppm – 10.00 ppm] of cyclic peptide **CP2** at 300 K in methanol-*d3* at 600 MHz. NS =16, d1= 5 s, AQ = 0.4 s, SW = 16.6 ppm, TD = 8k.

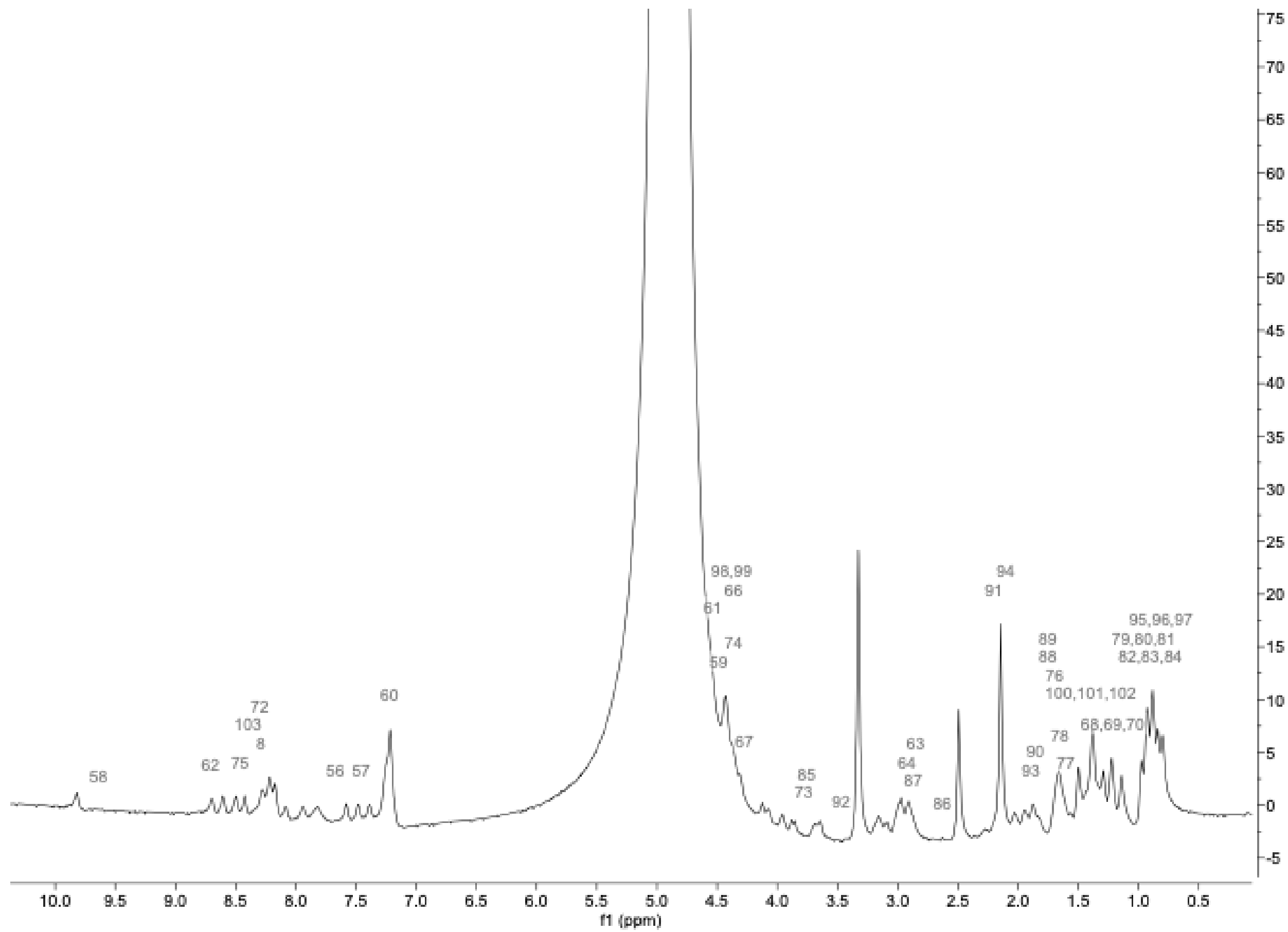

Figure S7. Proton spectrum of cyclic peptide **CP2** at 300 K in anisotropic methanol-*d3*/AAKLVFF at 600 MHz. NS =16, d1= 5 s, AQ = 0.4 s, SW = 16.6 ppm, TD = 8k.

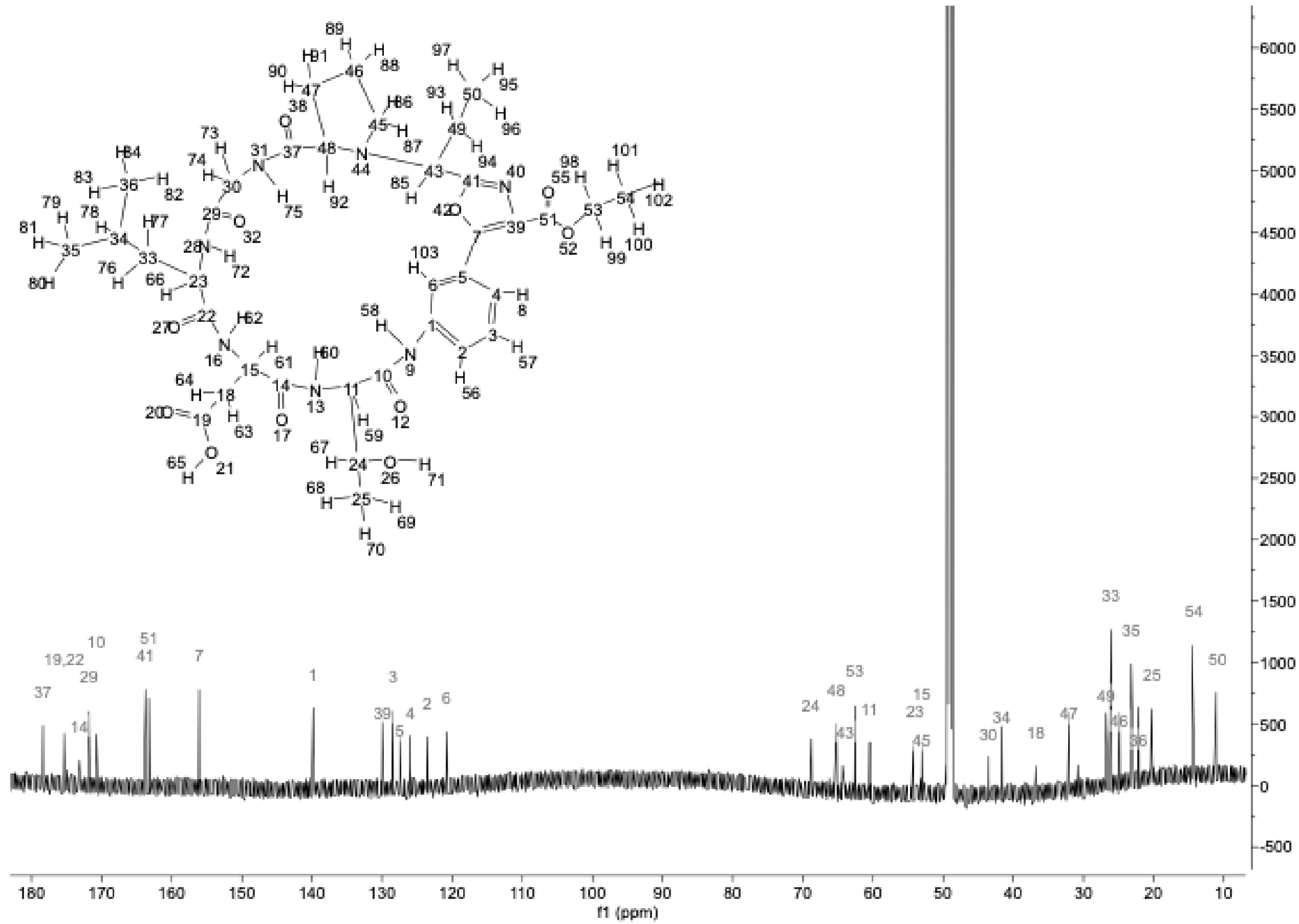

Figure S58. Carbon spectrum of cyclic peptide **CP2** at 300 K in methanol-*d4* at 600 MHz. NS =1024 (TD0 =14), d1= 2.5 s, AQ = 2.1 s, SW = 207 ppm, TD = 128k, 5.0 Hz LB.

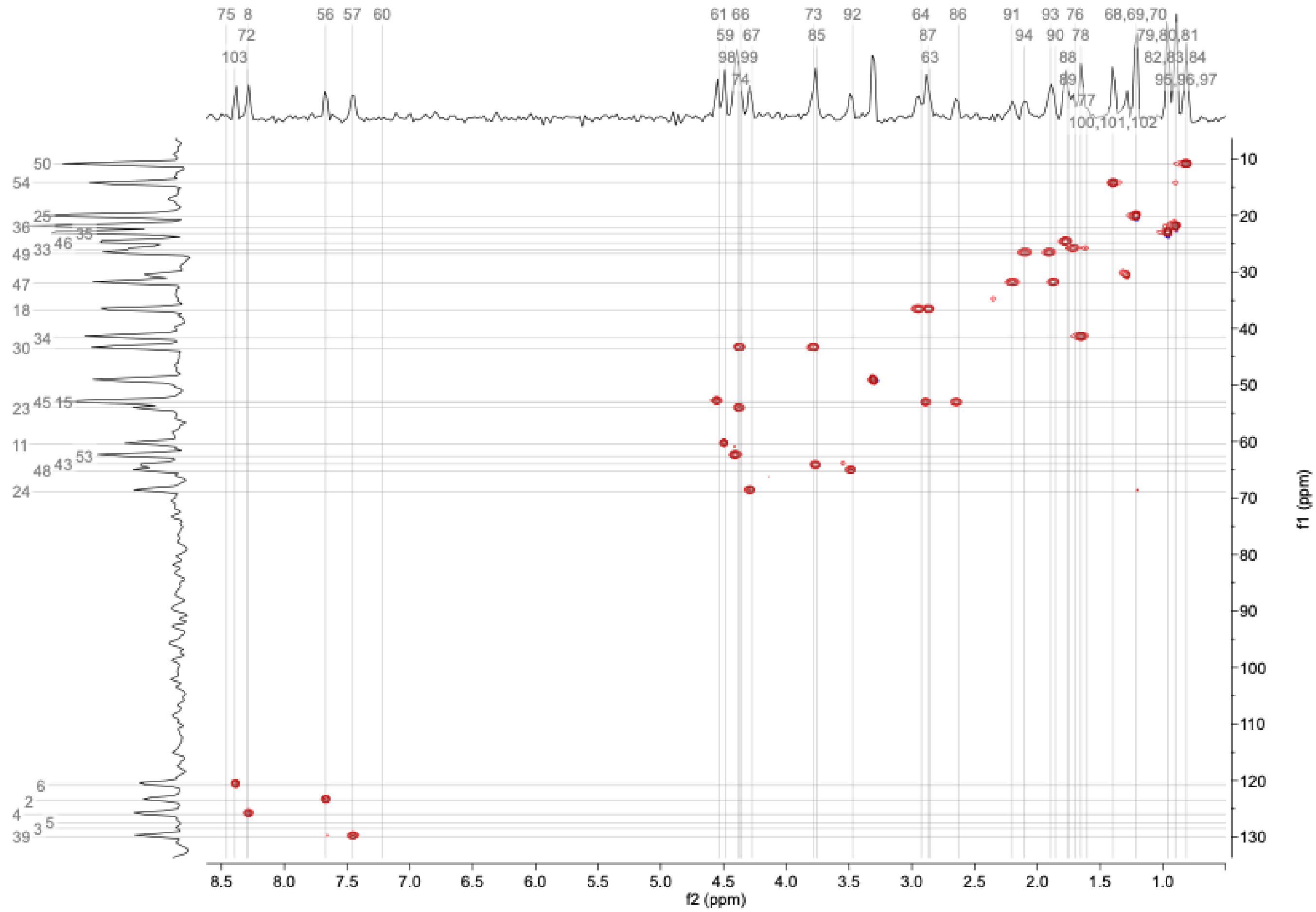

Figure S59. HSQC spectrum of cyclic peptide **CP2** at 300 K in methanol-*d4* at 600 MHz. NS=12, d1 = 1.3 s, SW 10 (F2) x 170 (F1), TD 1k x 512, echo-antiecho.

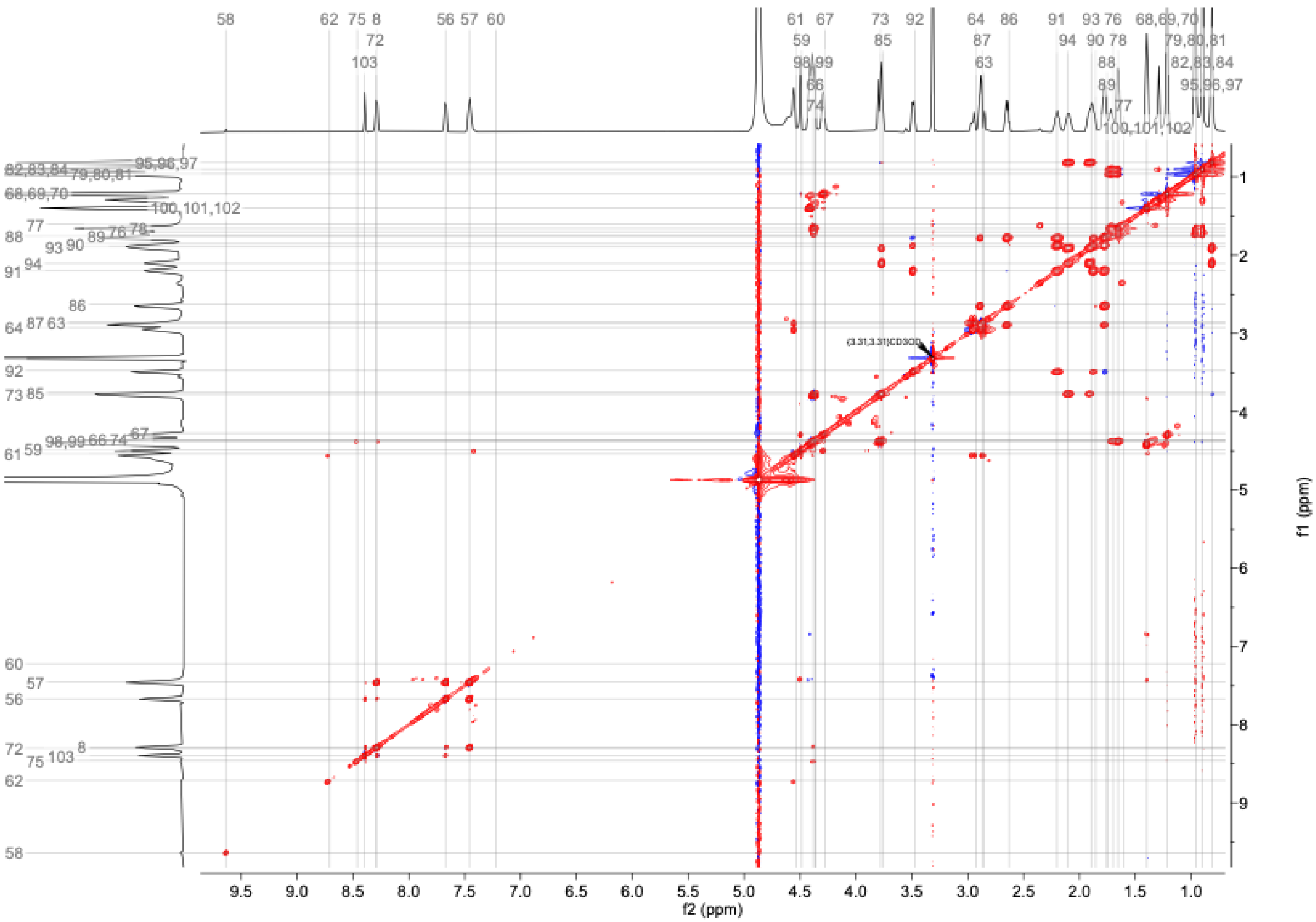

Figure S60. CLIP-COSY spectrum of cyclic peptide **CP2** at 300 K in methanol-*d4* at 600 MHz. NS=8, d1= 1.3s, SW 14 (F2) x 14 (F1), TD 4k x 1k, States-TPPI.

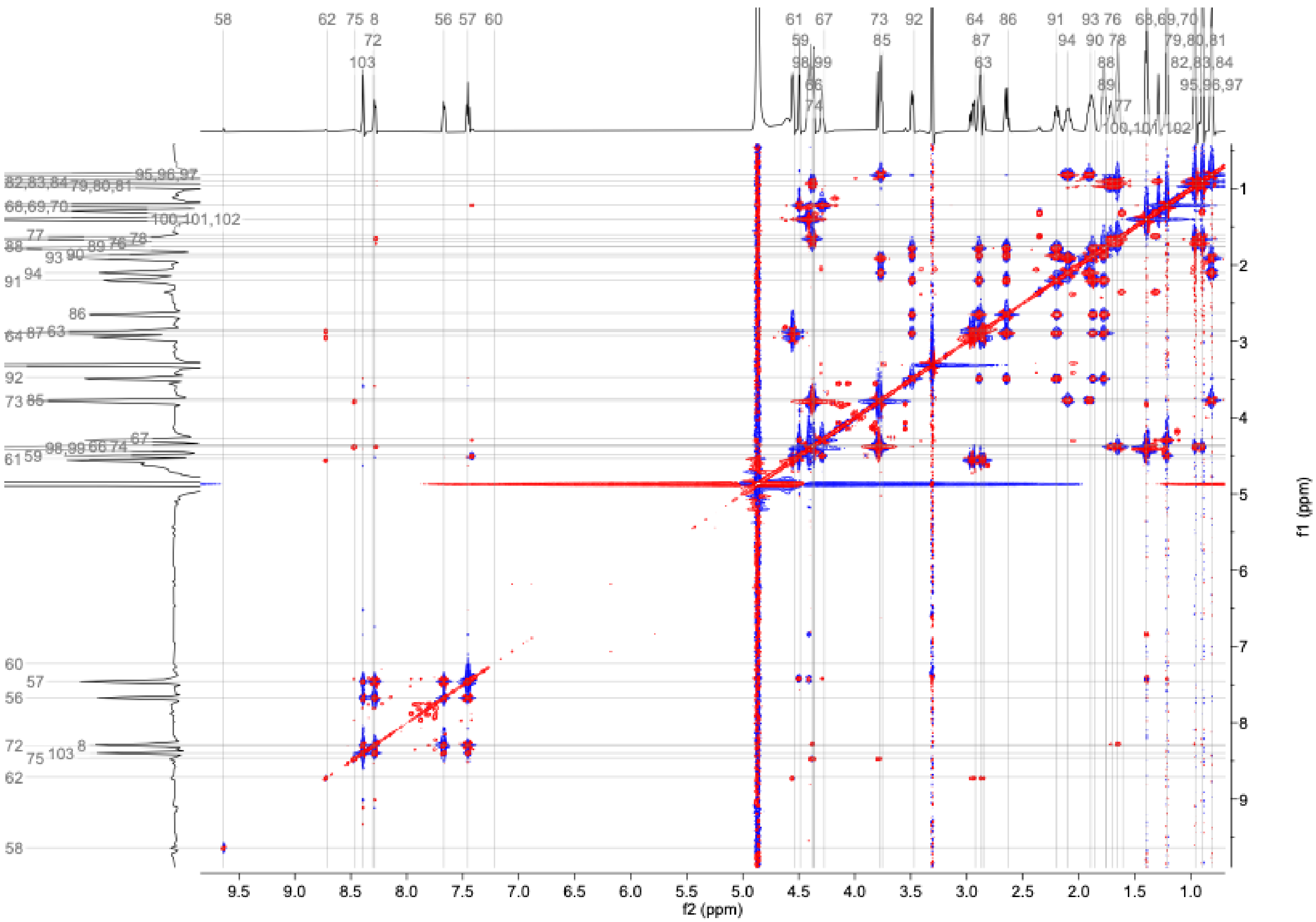

Figure S61. TOCSY spectrum of cyclic peptide **CP2** at 300 K in methanol-*d4* at 600 MHz. NS=8, d1= 1.3s, SW 14 (F2) x 14 (F1), TD 4k x 1k, States-TPPI, 80 ms mixing time.

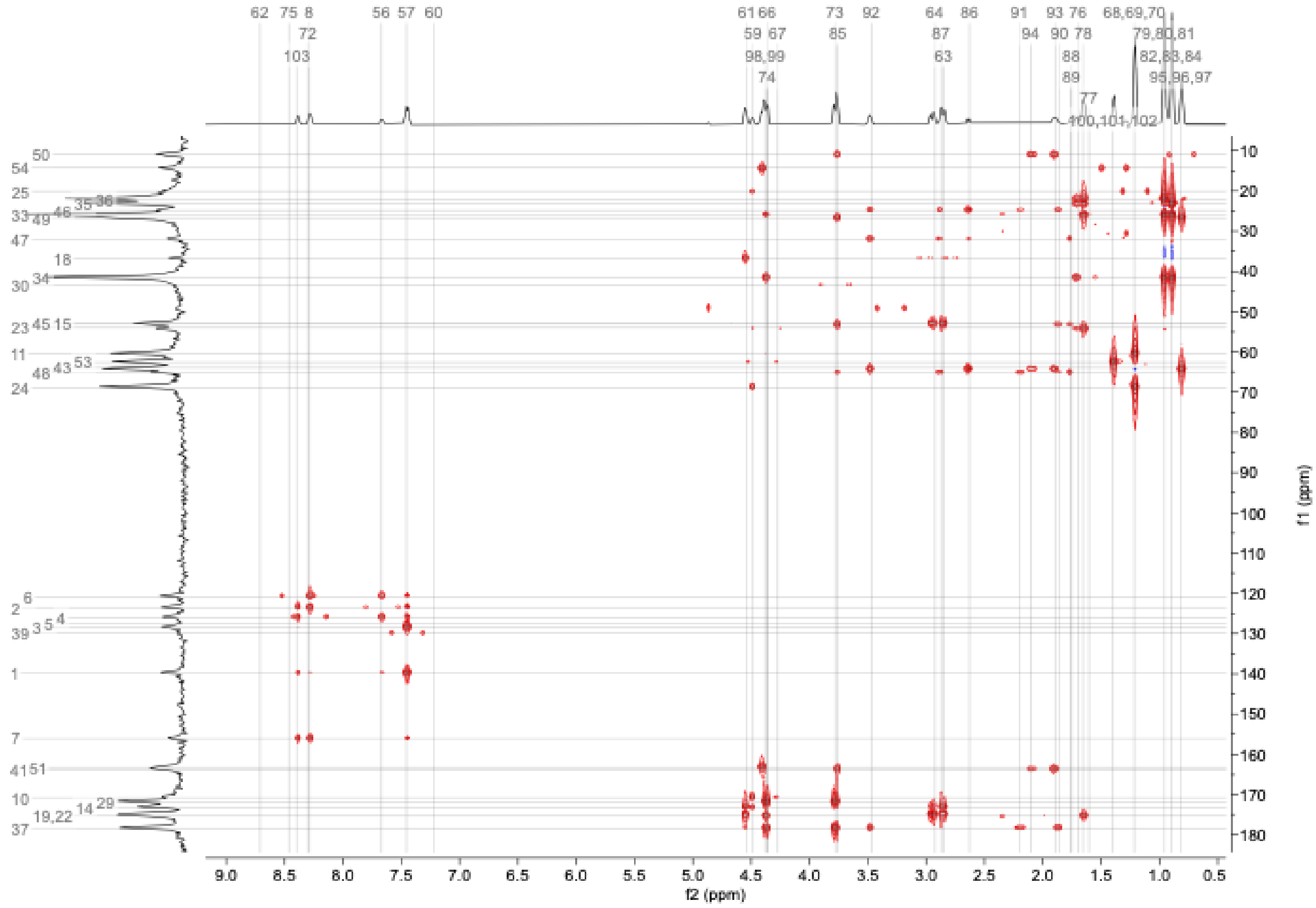

Figure S62. HMBC spectrum of cyclic peptide **CP2** at 300 K in methanol-*d4* at 600 MHz. NS=32, d1= 1.3s, SW 16.6 (F2) x 207 (F1), TD 2k x 1k, delay for long range coupling 70 ms, echo-antiecho.

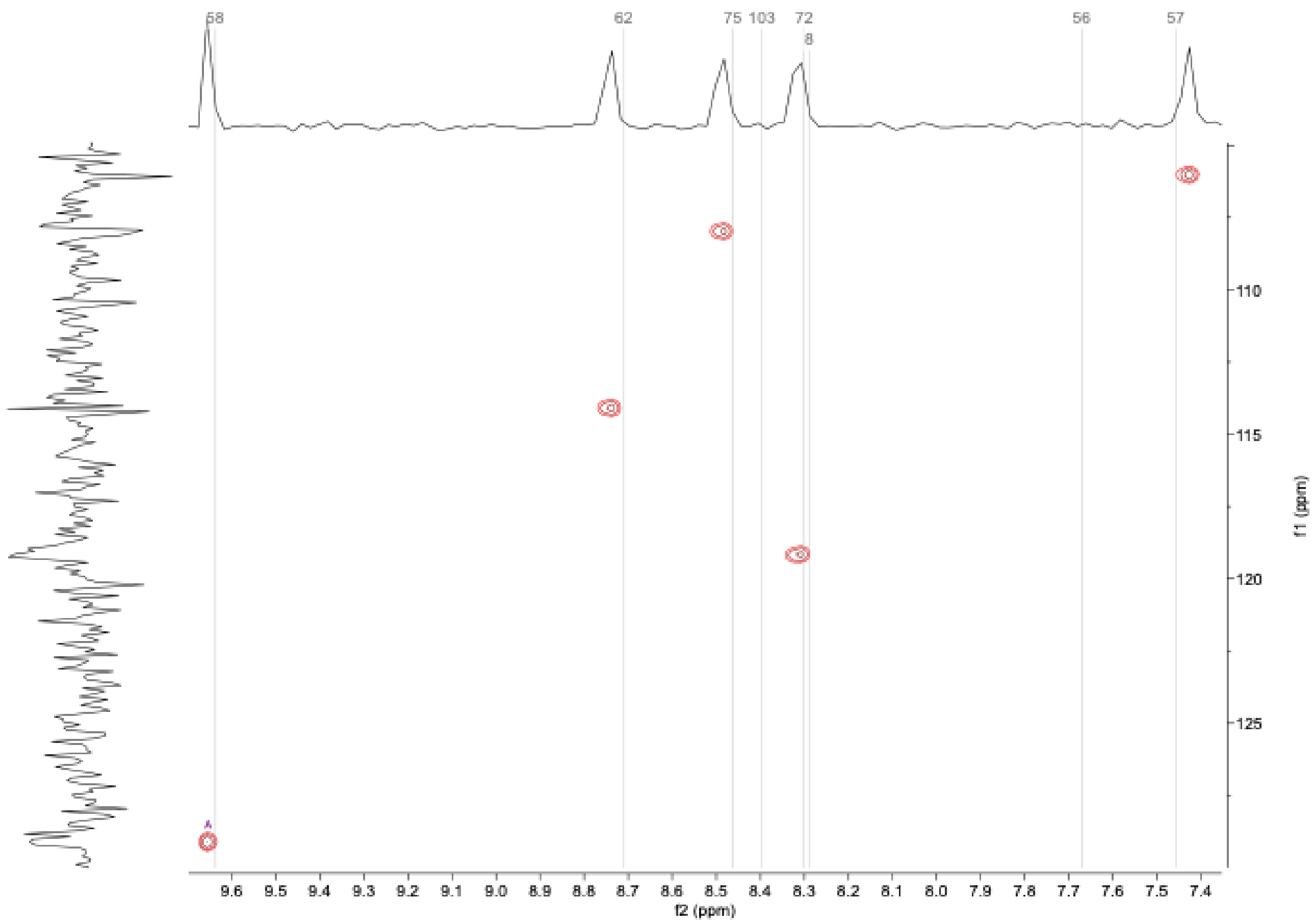

Figure S63. 15N-HSQC spectrum of cyclic peptide **CP2** at 300 K in methanol-*d3* at 600 MHz. NS=48, d1= 1.3s, SW 10 (F2) x 50 (F1), TD 1k x 256, States-TPPI, WaterGATE.

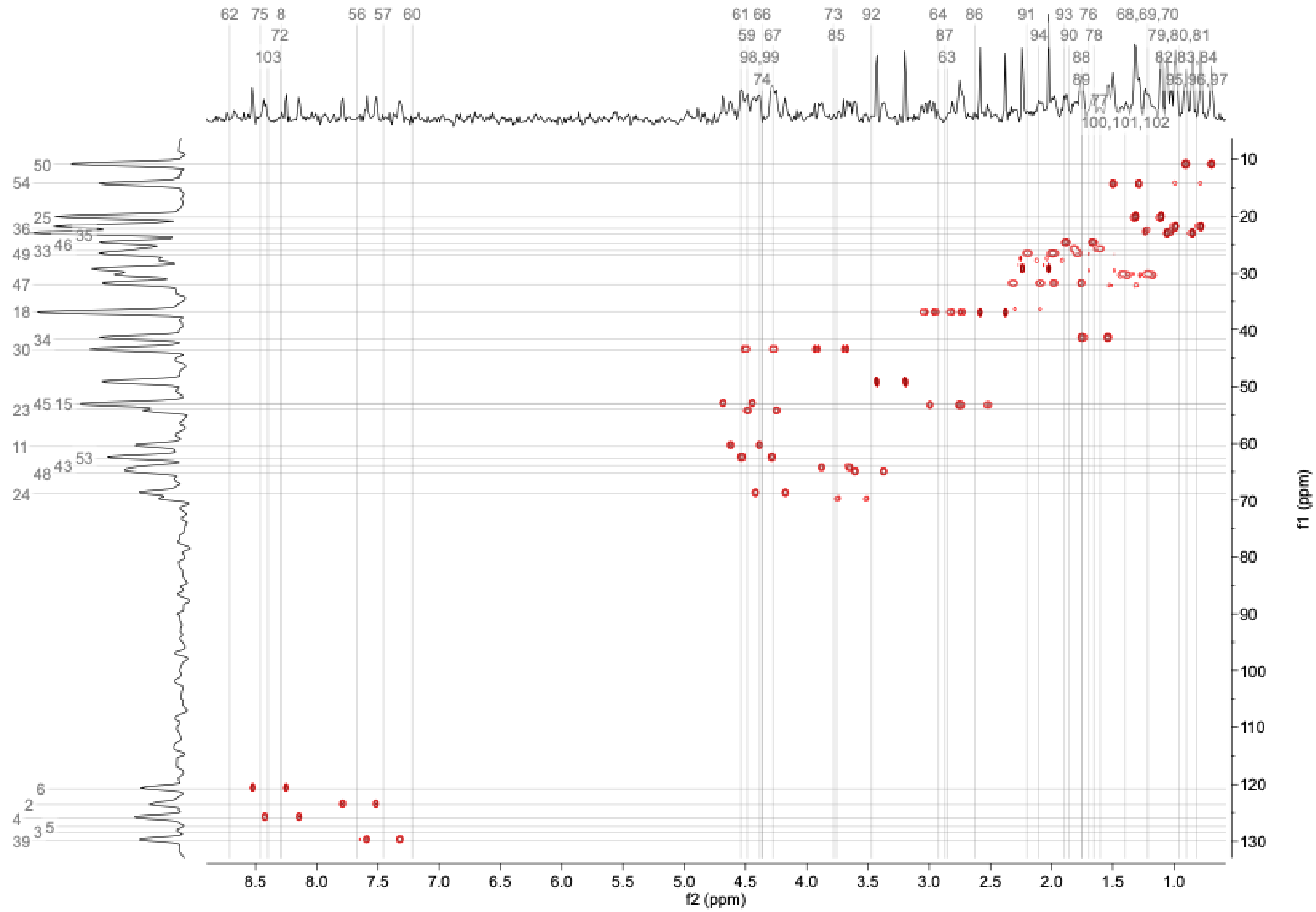

Figure S64. CLIP-HSQC spectrum of cyclic peptide **CP2** at 300 K in methanol-*d3* at 600 MHz. NS=16, d1 = 1.3 s, SW 10 (F2) x 200 (F1), TD 2k x 512, echo-antiecho, coupling in F2.

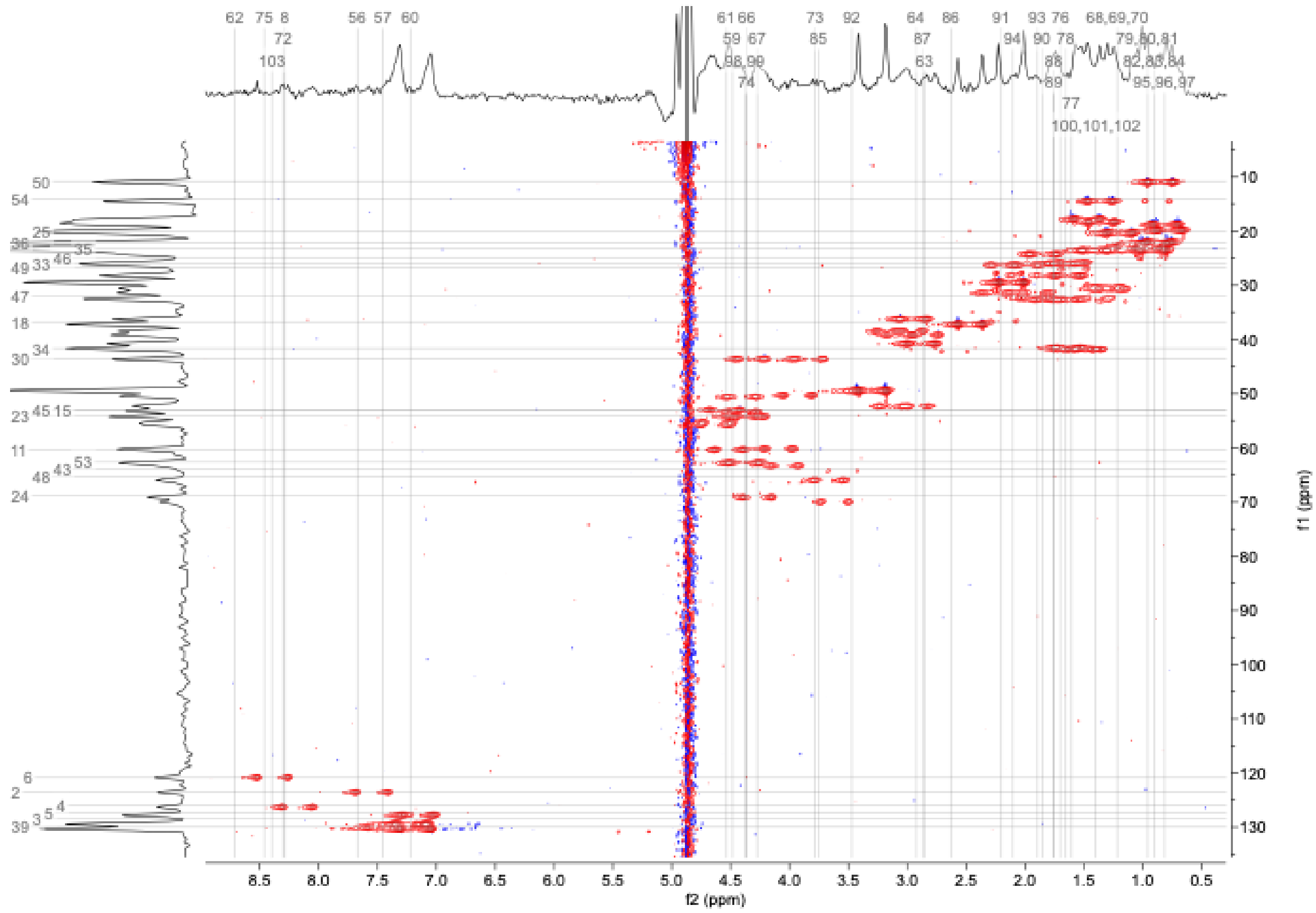

Figure S65. CLIP-HSQC spectrum of cyclic peptide **CP2** at 300 K in methanol-*d3*/AAKLVFF at 600 MHz. NS=16, d1 = 1.3 s, SW 10 (F2) x 200 (F1), TD 2k x 512, echo-antiecho, coupling in F2.

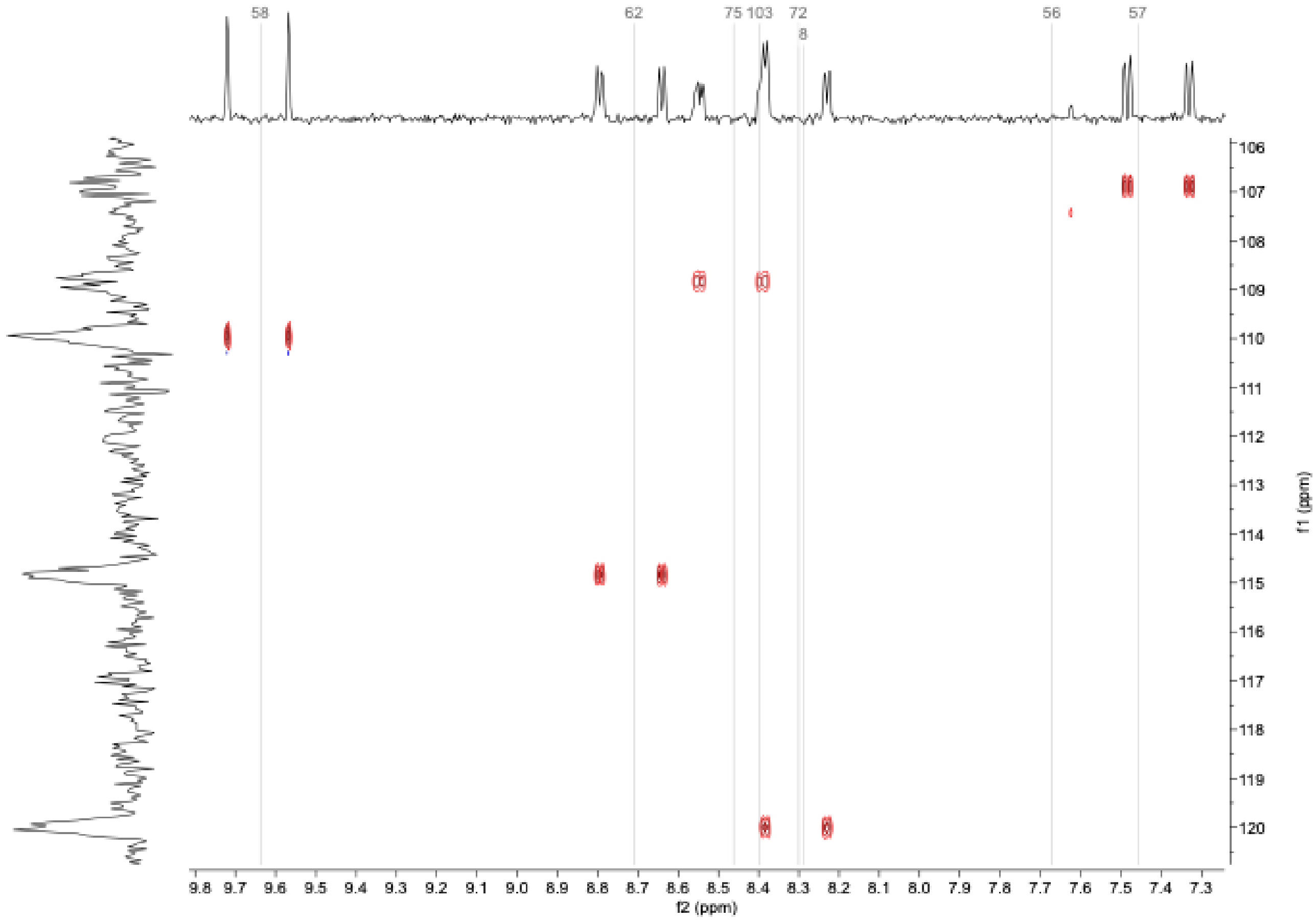

Figure S66. ^{15}N-CLIP-HSQC spectrum of cyclic peptide **CP2** at 300 K in methanol-*d3* at 600 MHz. NS=256, d1 = 1.3 s, SW 3 (F2) x 20 (F1), TD 2k x 128, echo-antiecho, coupling in F2. WaterGATE.

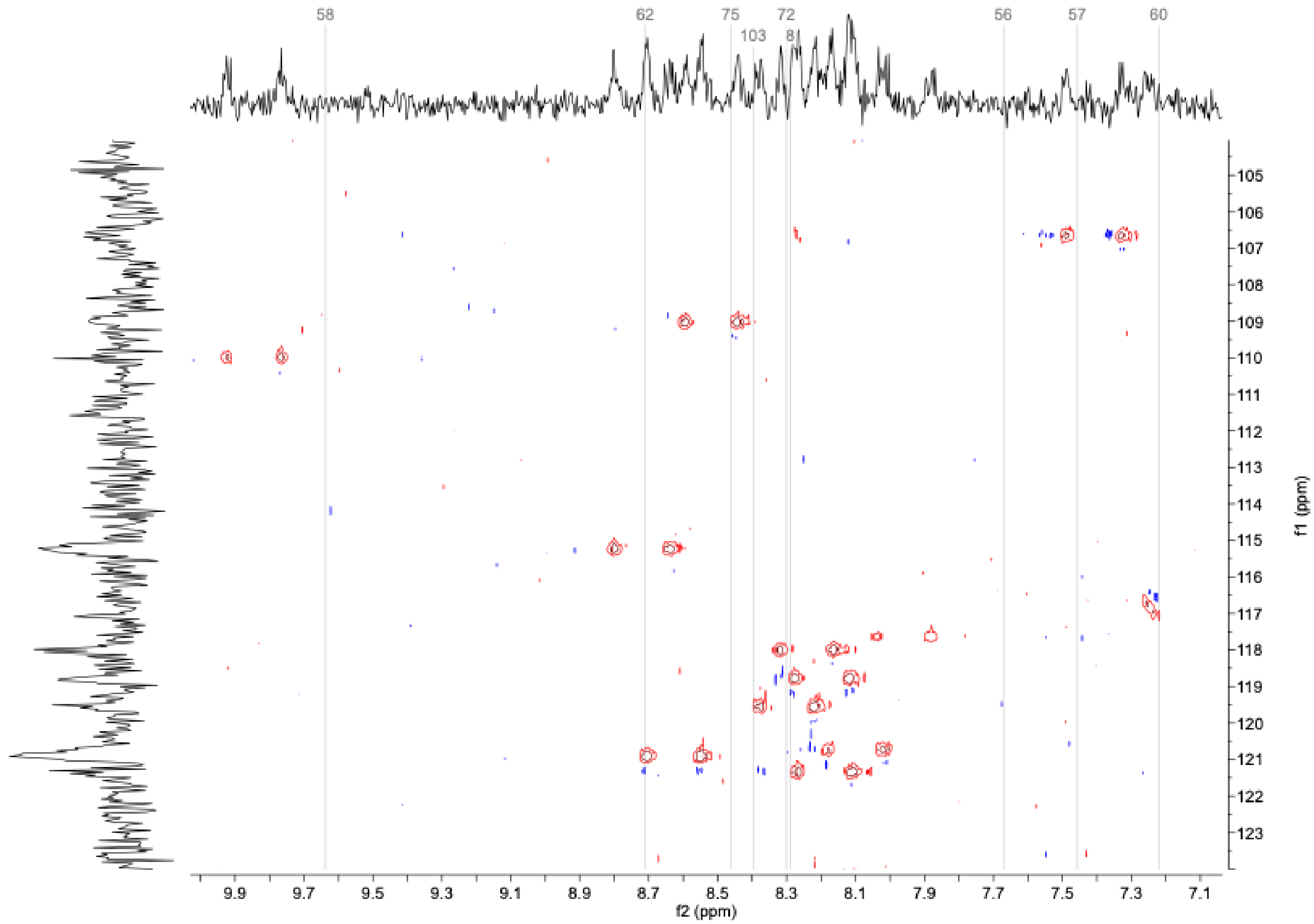

Figure S67. ^{15}N-CLIP-HSQC spectrum of cyclic peptide **CP2** at 300 K in methanol-*d3*/AAKLVFF at 600 MHz. NS=256, d1 = 1.3 s, SW 3 (F2) x 20 (F1), TD 2k x 128, echo-antiecho, coupling in F2. WaterGATE.

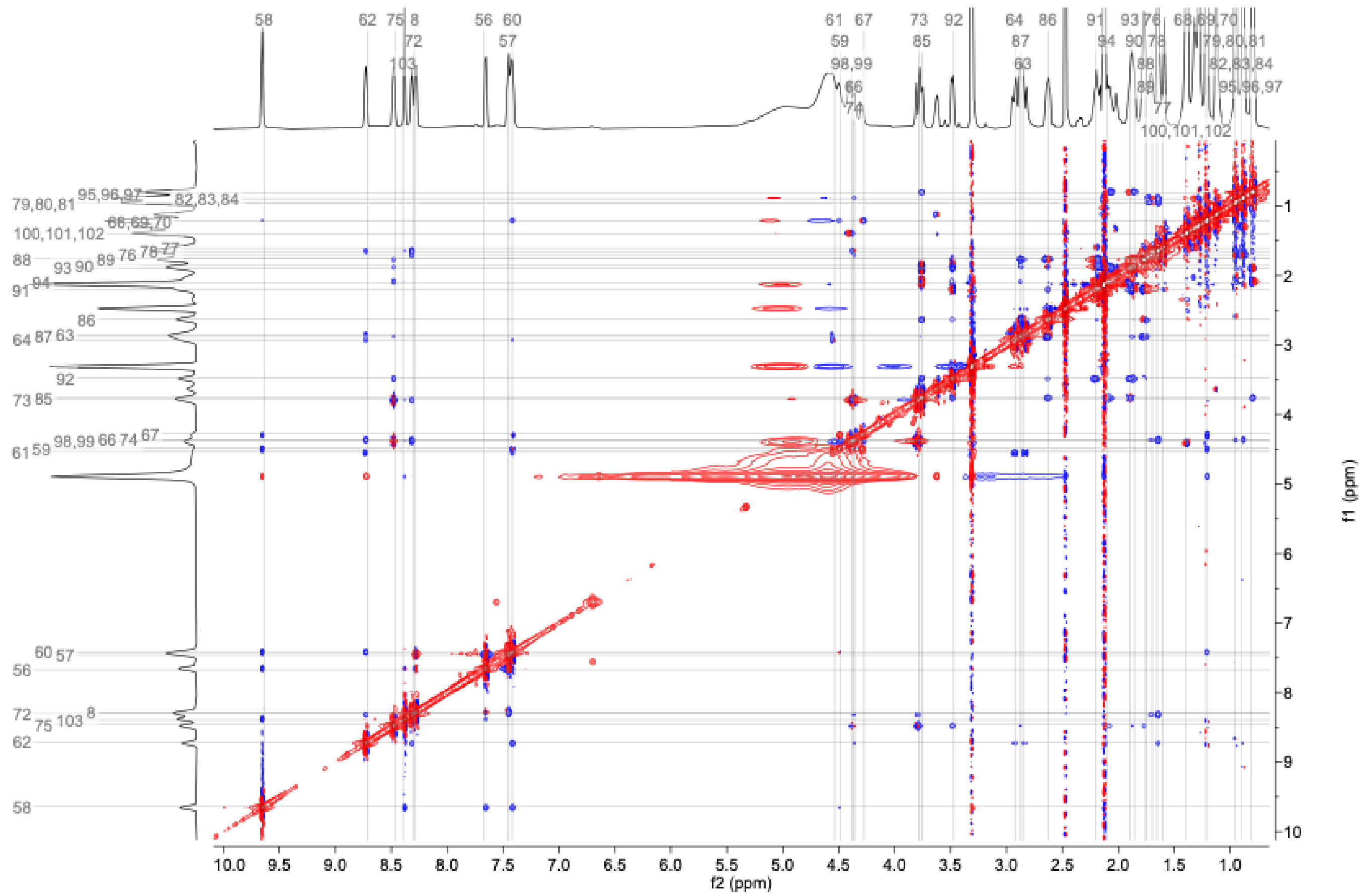

Figure S68. ROESY spectrum of cyclic peptide **CP2** at 300 K in methanol-*d3* at 600 MHz. NS=32, d1 = 1.3 s, SW 13 (F2) x 13 (F1), TD 2k x 512, States-TPPI, mixing time = 80 ms.

SI.16.3 Spectra for CP3

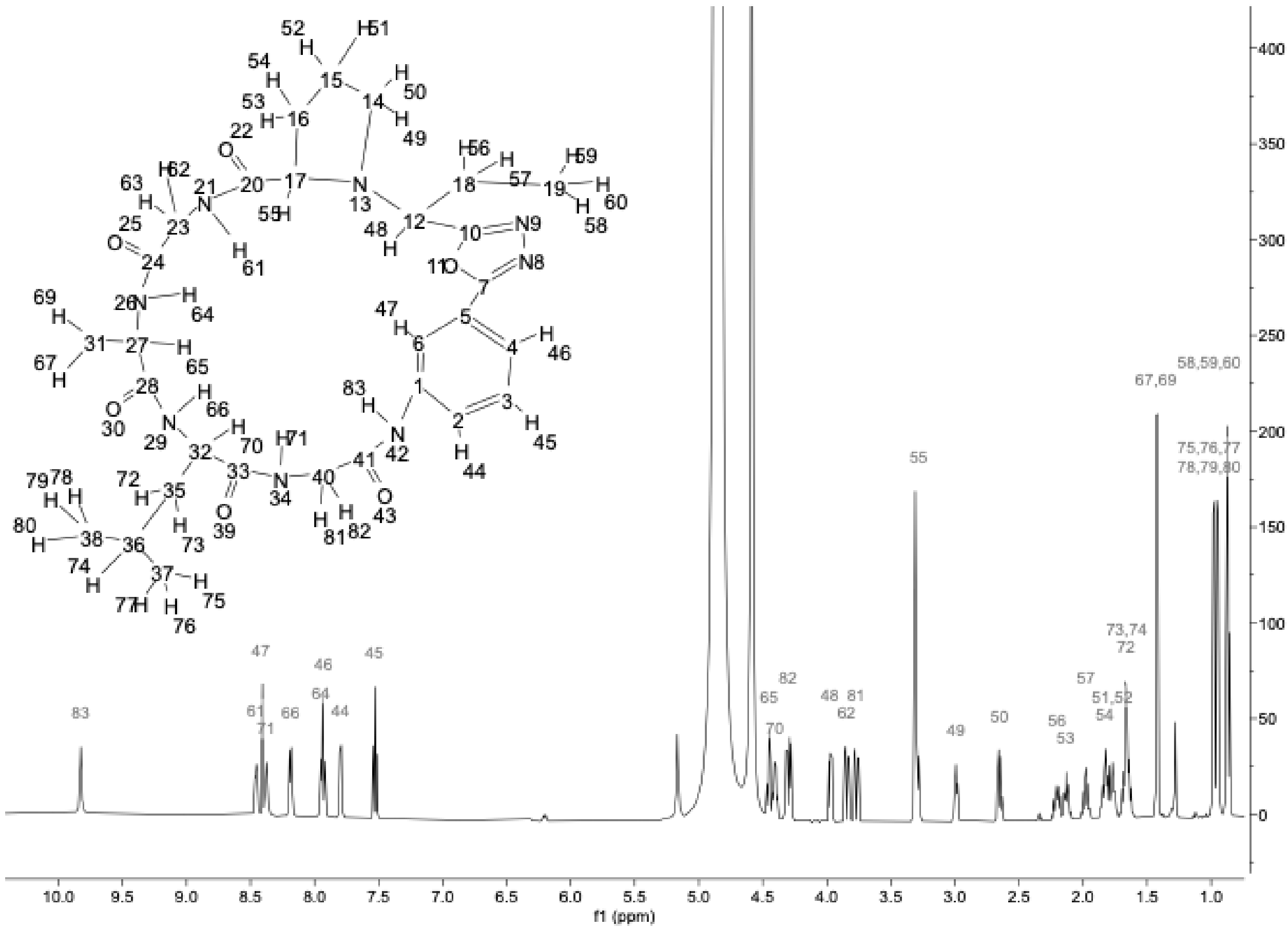

Figure S69. Proton spectrum of cyclic peptide **CP3** at 300 K in methanol-*d3* at 600 MHz. NS =32, d1= 5 s, AQ = 1.6 s, SW = 16.6 ppm, TD = 32k.

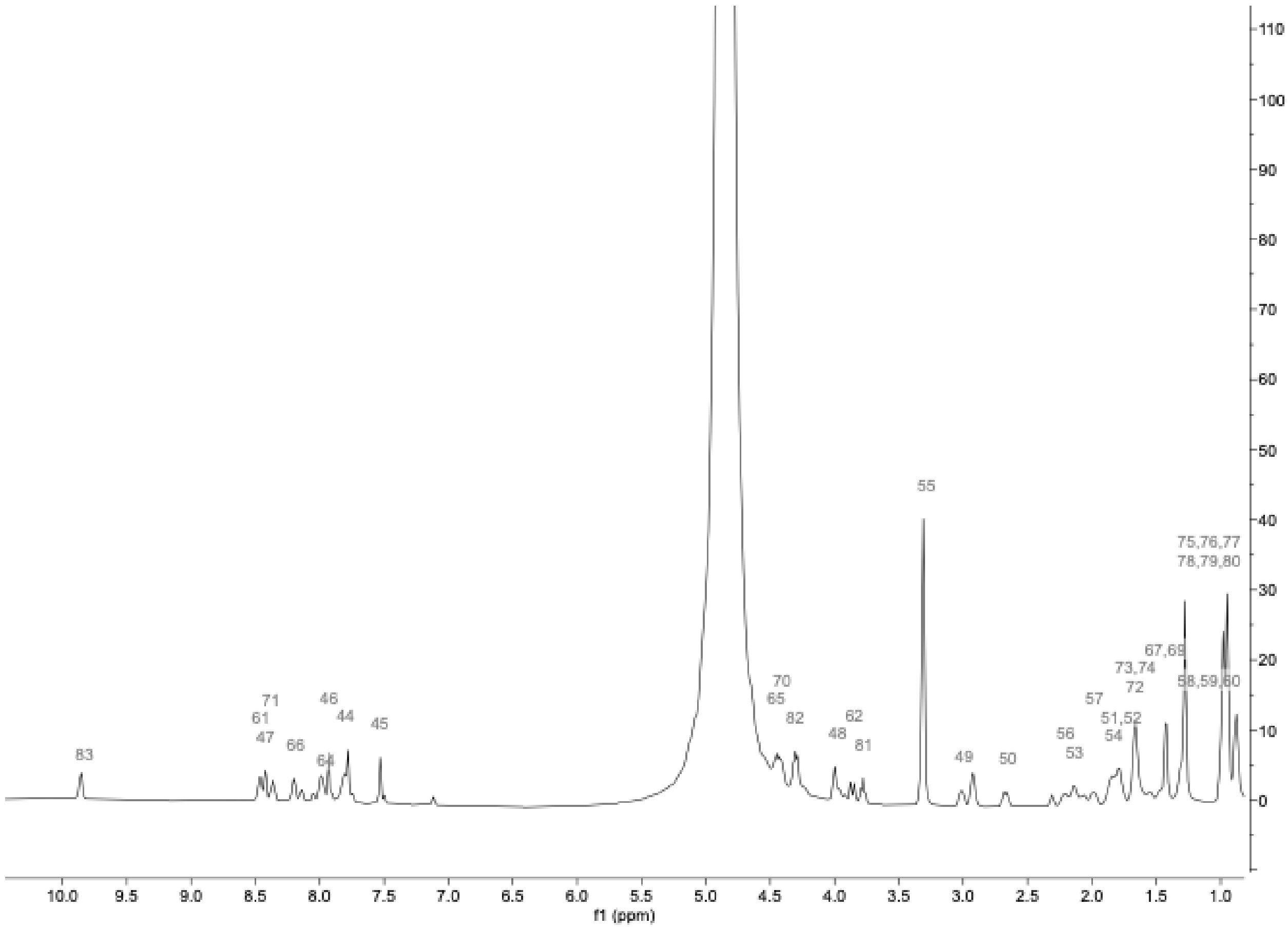

Figure S70. Proton spectrum of cyclic peptide **CP3** at 300 K in anisotropic methanol-*d3*/OPA at 600 MHz. NS =32, d1= 5 s, AQ = 1.6 s, SW = 16.6 ppm, TD = 32k.

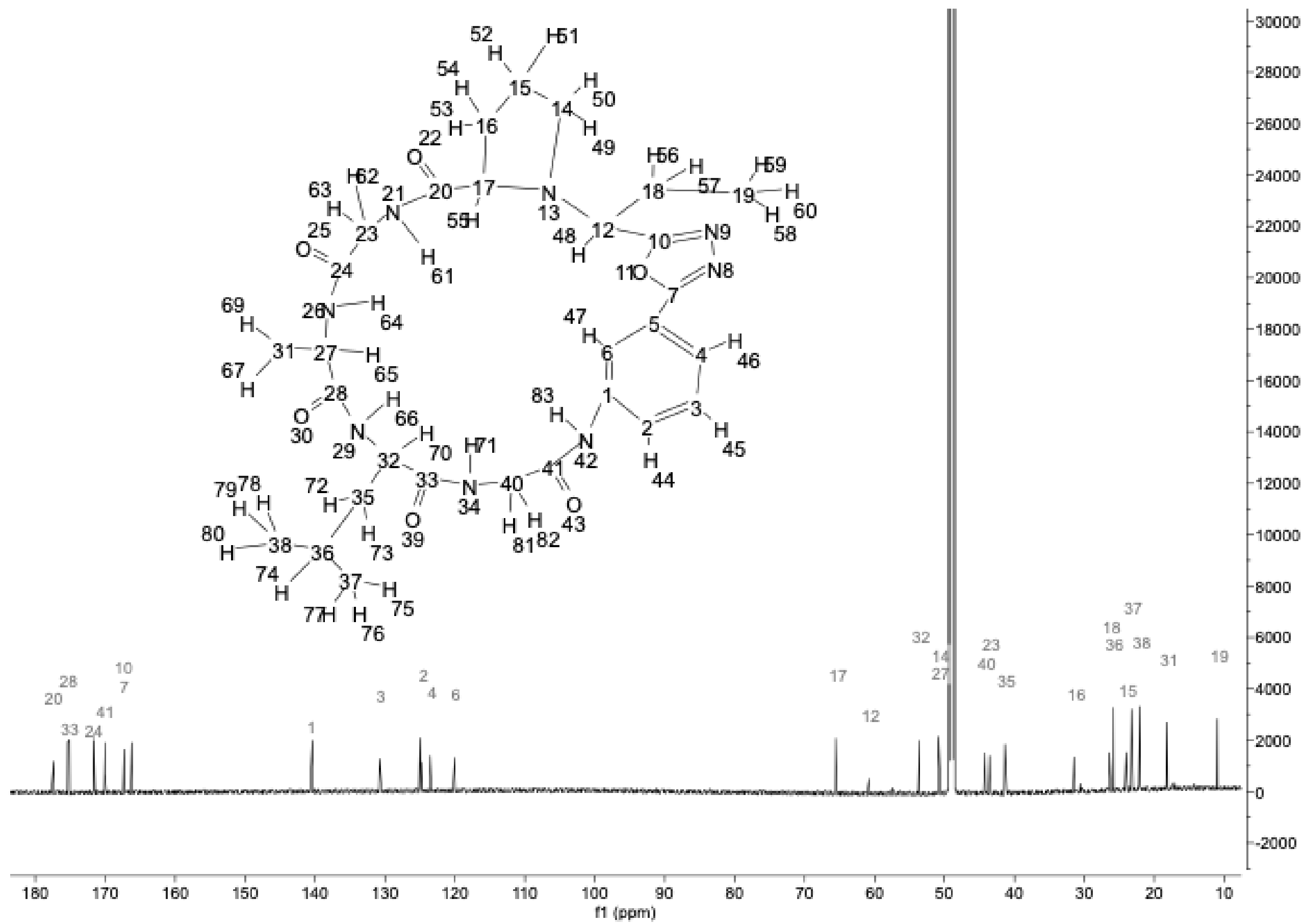

Figure S71. Carbon spectrum of cyclic peptide **CP3** at 300 K in methanol-*d3* at 600 MHz. NS =1024 (TD0 =25), d1= 2.5 s, AQ = 2.0 s, SW = 212.4 ppm, TD = 128k, 5.0 Hz LB.

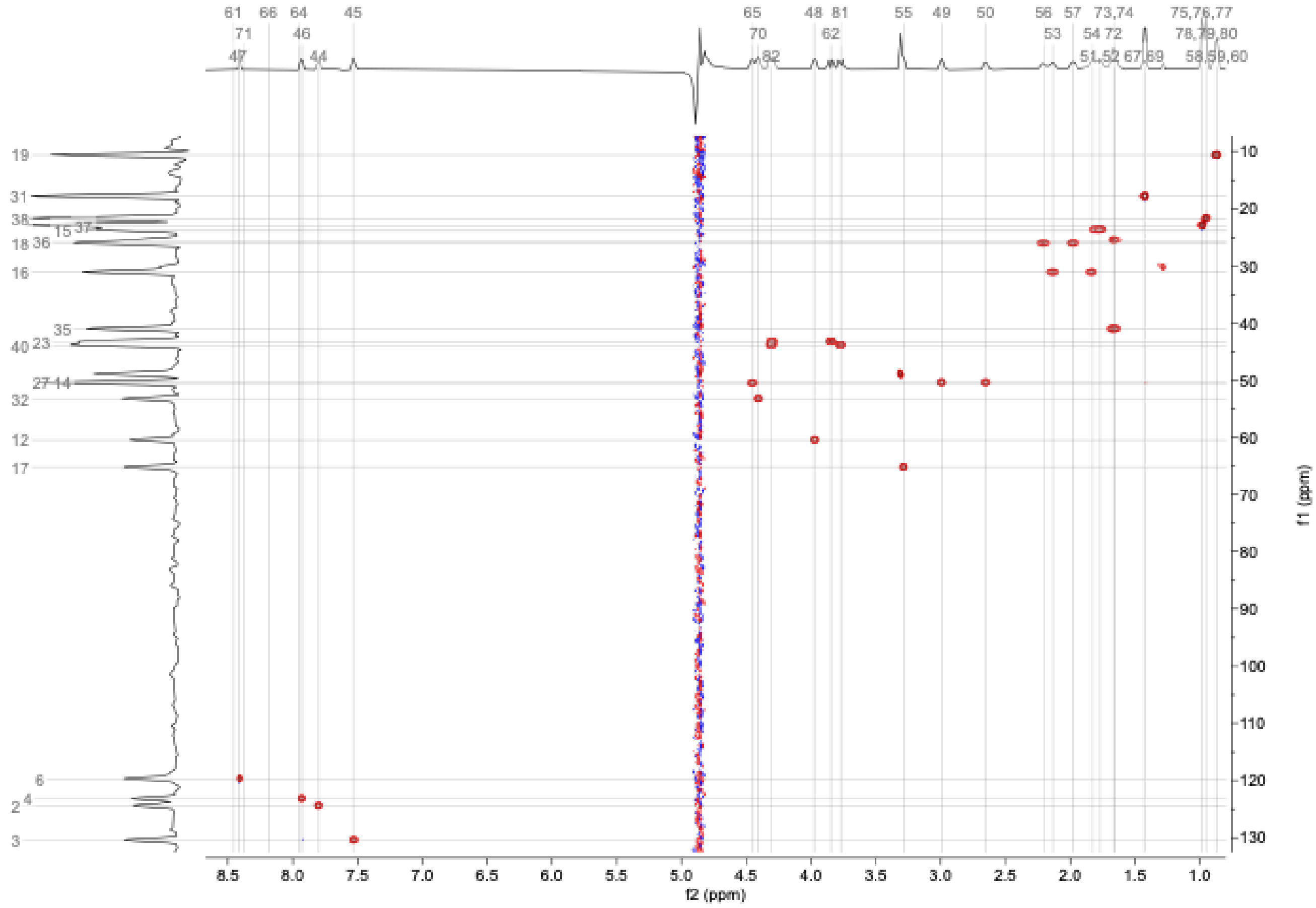

Figure S72. HSQC spectrum of cyclic peptide **CP3** at 300 K in methanol-*d3* at 600 MHz. NS=32, d1 = 1.3 s, SW 12 (F2) x 160 (F1), TD 2k x 512, echo-antiecho.

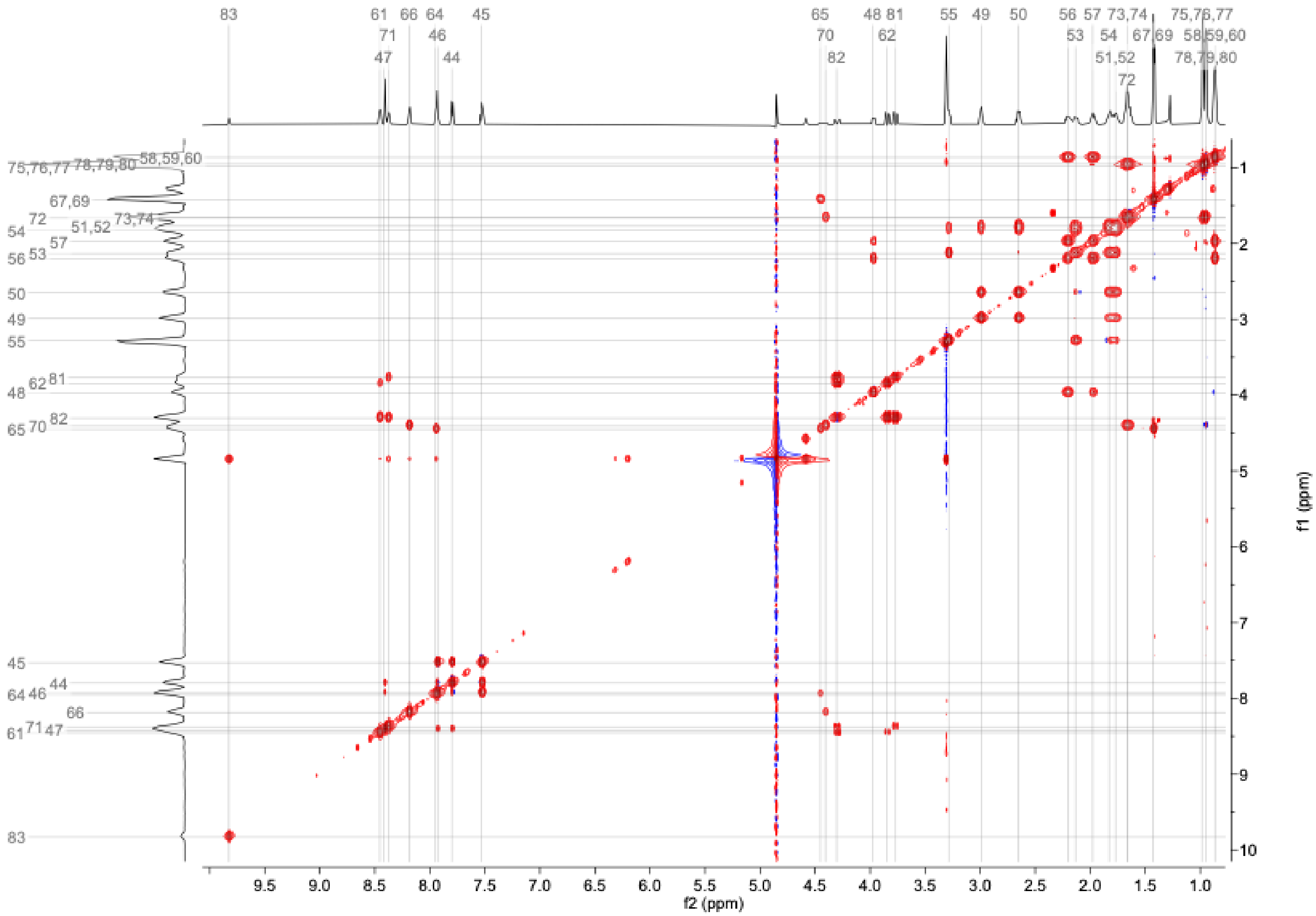

Figure S73. CLIP-COSY spectrum of cyclic peptide **CP3** at 300 K in methanol-*d3* at 600 MHz. NS=16, d1= 1.3s, SW 14 (F2) x 14 (F1), TD 4k x 512, States-TPPI.

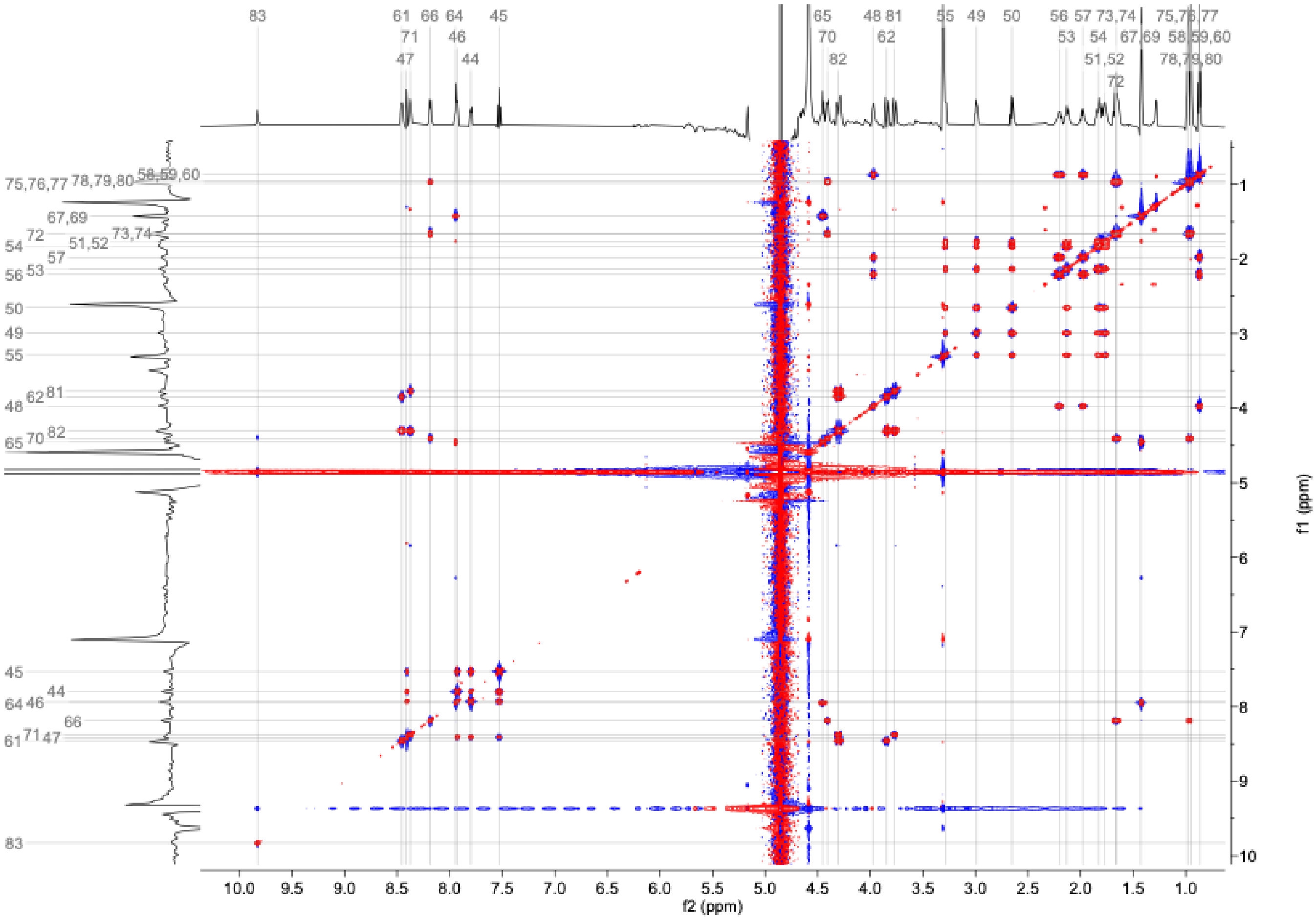

Figure S74. TOCSY spectrum of cyclic peptide **CP3** at 300 K in methanol-*d3* at 600 MHz. NS=16, d1= 1.3s, SW 14 (F2) x 14 (F1), TD 2k x 1k, States-TPPI, 80 ms mixing time.

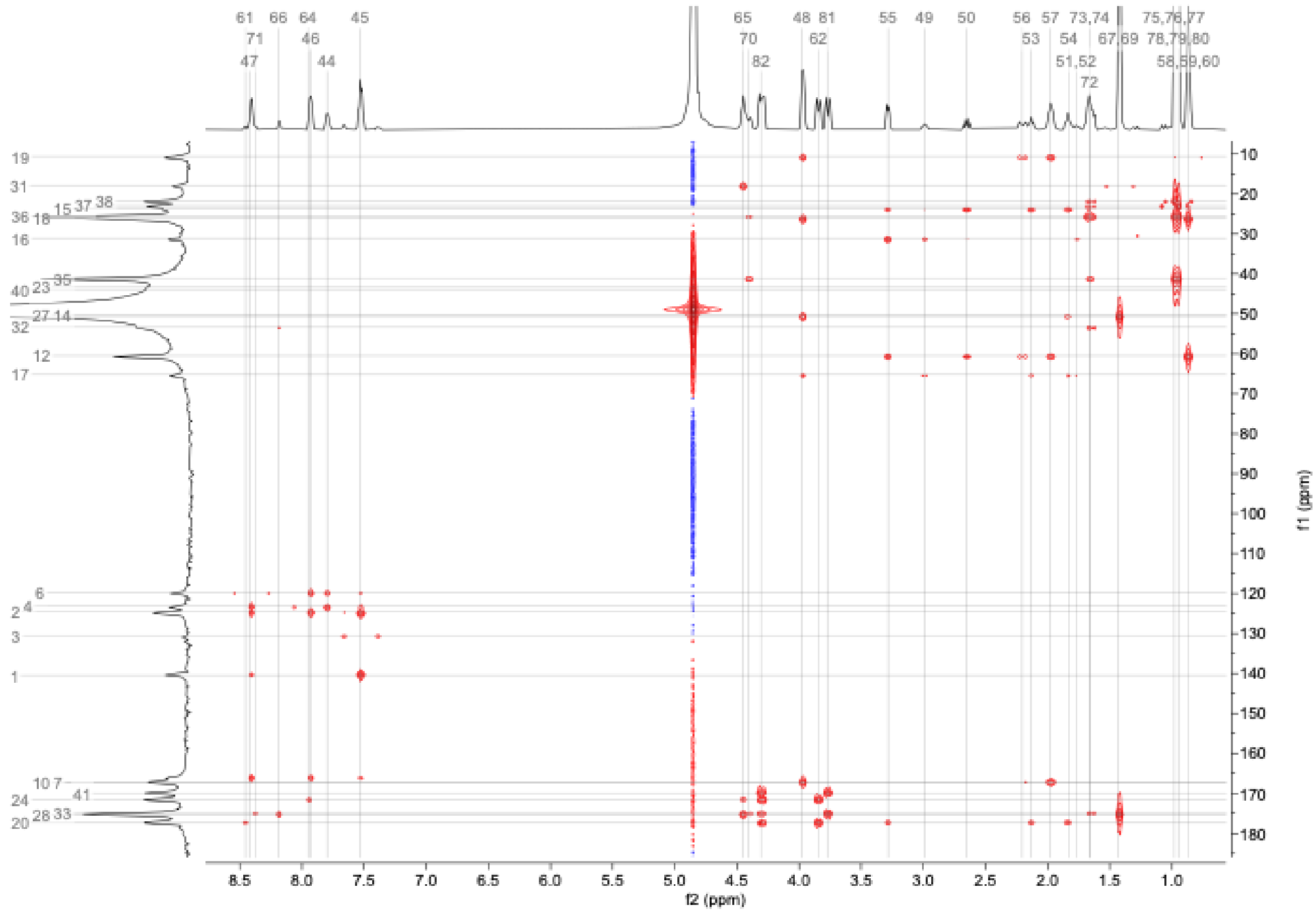

Figure S75. HMBC spectrum of cyclic peptide **CP3** at 300 K in methanol-*d3* at 600 MHz. NS=32, d1= 1.3s, SW 16.6 (F2) x 207 (F1), TD 2k x 1k, delay for long range coupling 70 ms, echo-antiecho.

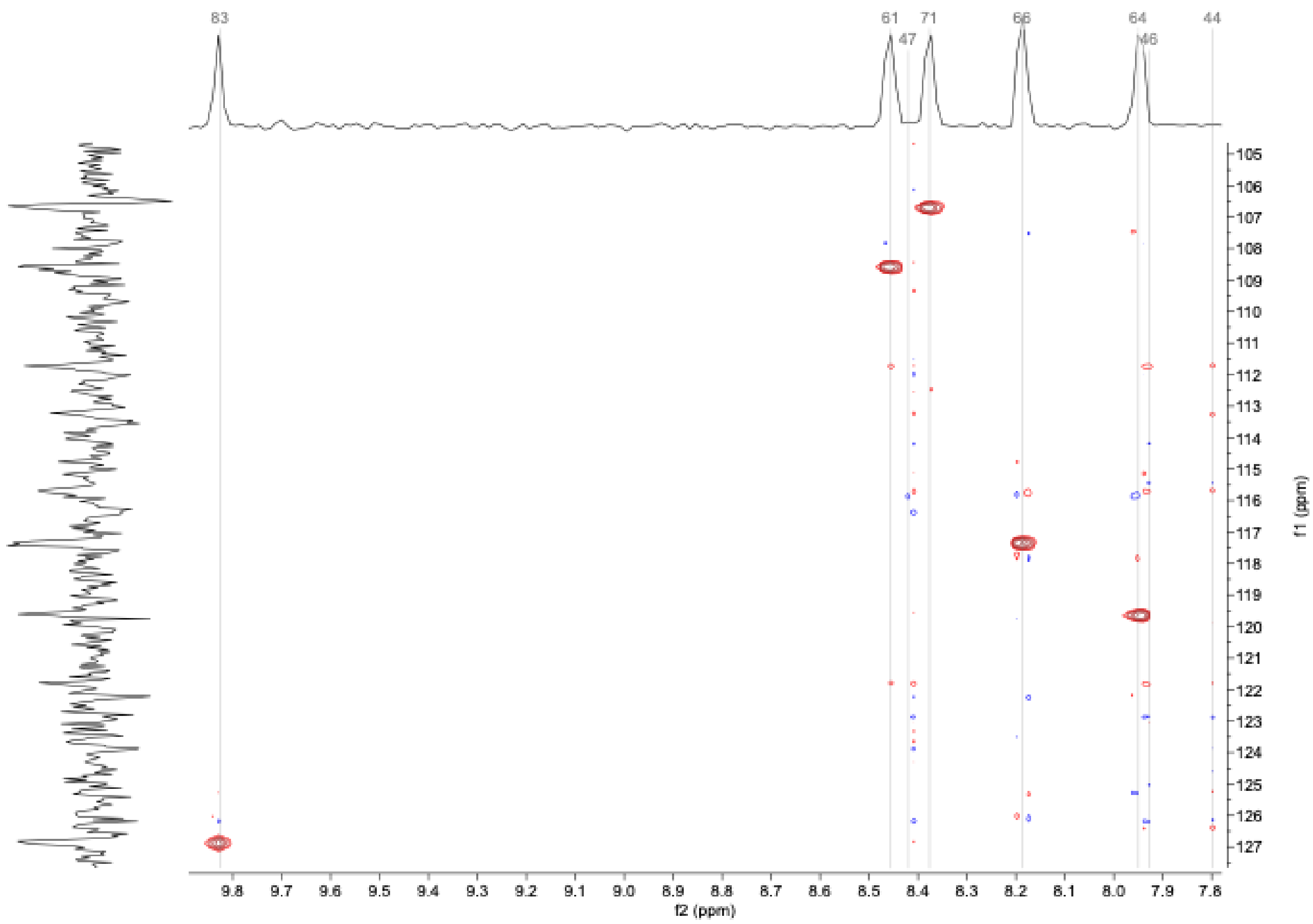

Figure S76. ^{15}N-HSQC spectrum of cyclic peptide **CP3** at 300 K in methanol-*d3* at 600 MHz. NS=32, d1= 1.3s, SW 12 (F2) x 49 (F1), TD 2k x 512, States-TPPI, WaterGATE.

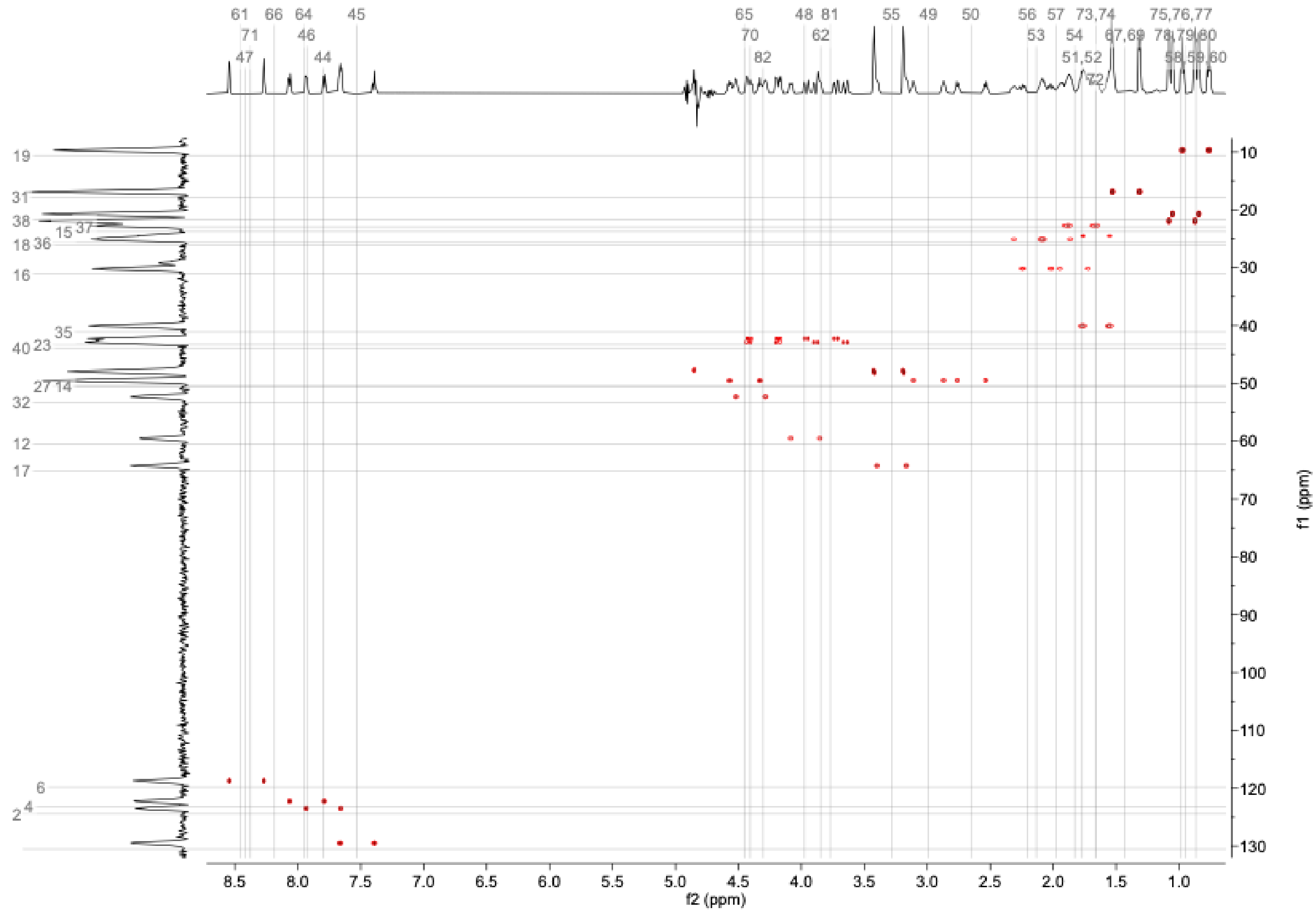

Figure S77. CLIP-HSQC spectrum of cyclic peptide **CP3** at 300 K in methanol-*d3* at 600 MHz. NS=56, d1 = 1.3 s, SW 11 (F2) x 150 (F1), TD 4k x 512, echo-antiecho, coupling in F2.

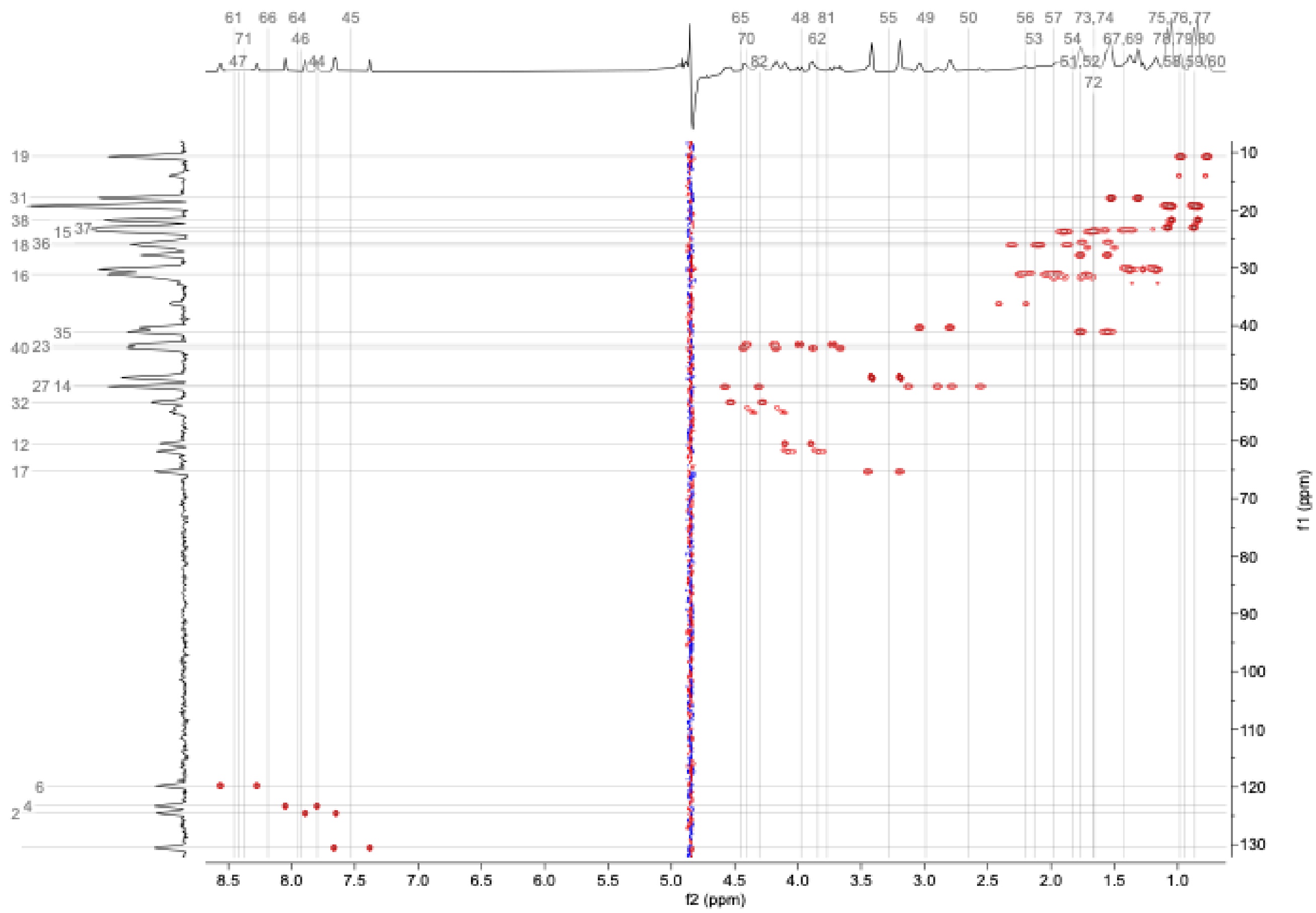

Figure S78. CLIP-HSQC spectrum of cyclic peptide **CP3** at 300 K in methanol-*d3*/OPA at 600 MHz. NS=56, d1 = 1.3 s, SW 11 (F2) x 150 (F1), TD 4k x 512, echo-antiecho, coupling in F2.

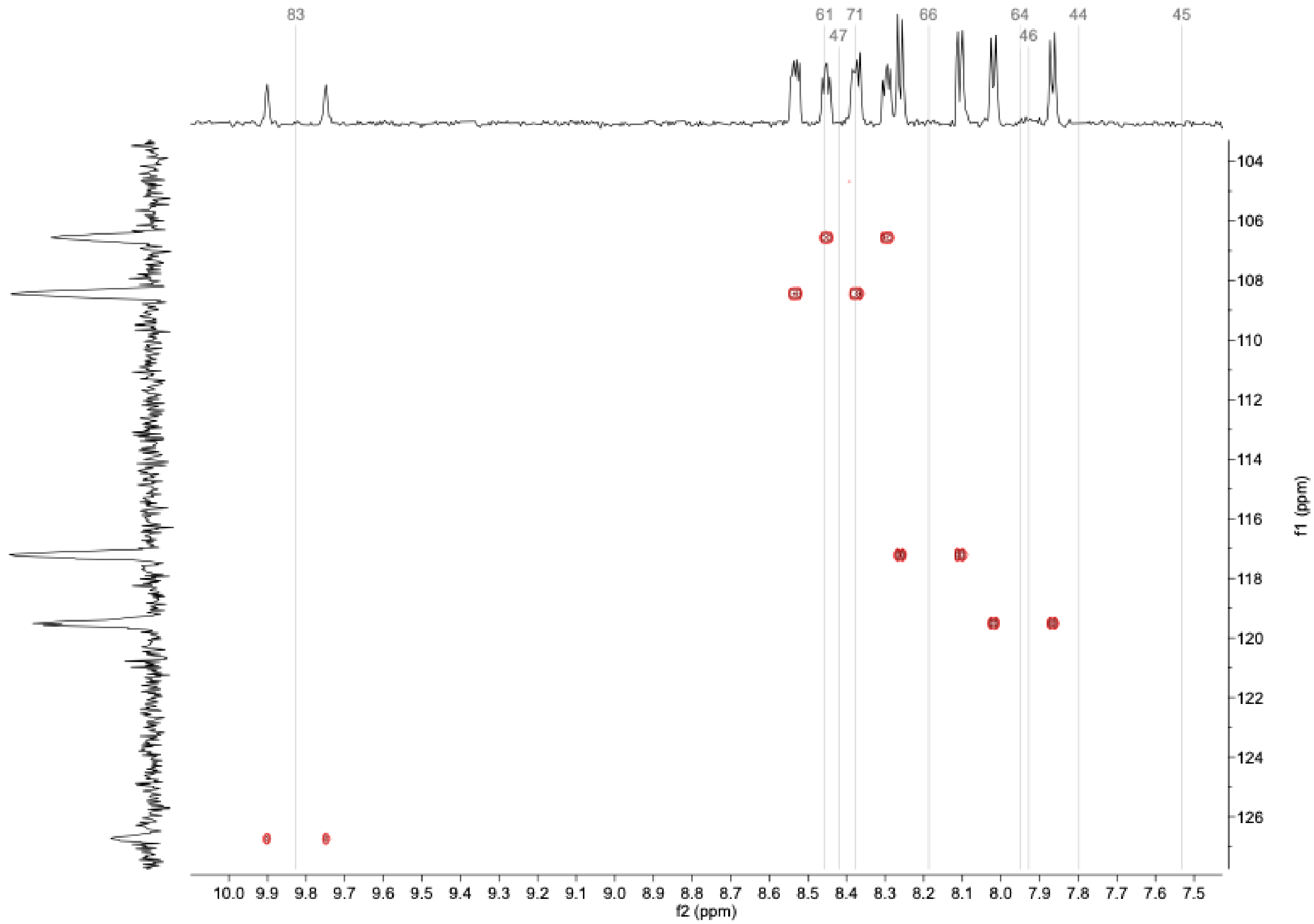

Figure S79. ^{15}N-CLIP-HSQC spectrum of cyclic peptide **CP3** at 300 K in methanol-*d3* at 600 MHz. NS=200, d1 = 1.3 s, SW 4 (F2) x 30 (F1), TD 2k x 256, echo-antiecho, coupling in F2. WaterGATE.

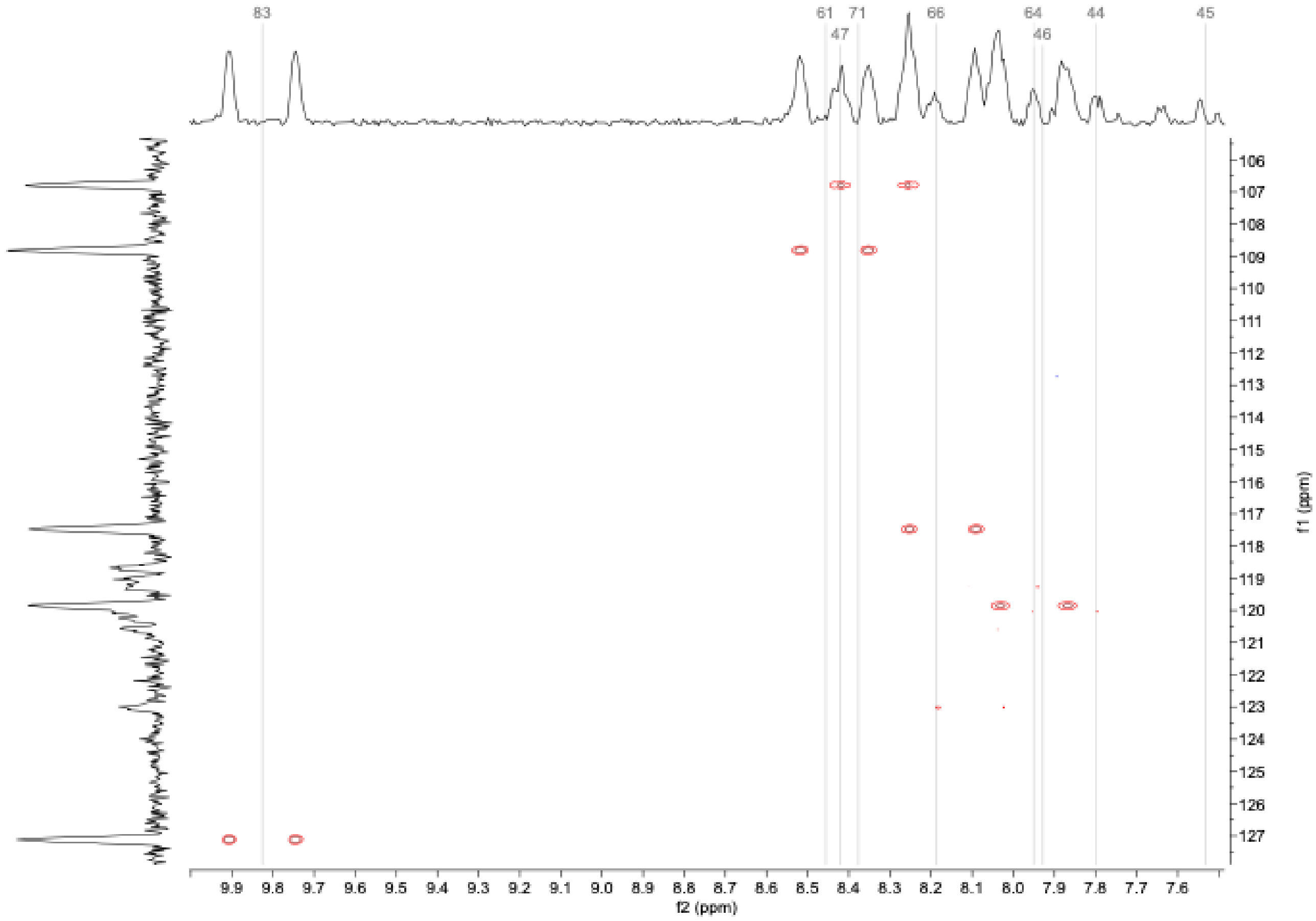

Figure S80. ^{15}N-CLIP-HSQC spectrum of cyclic peptide **CP3** at 300 K in methanol-*d3*/OPA at 600 MHz. NS=340, d1 = 1.3 s, SW 4 (F2) x 30 (F1), TD 2k x 256, echo-antiecho, coupling in F2. WaterGATE.

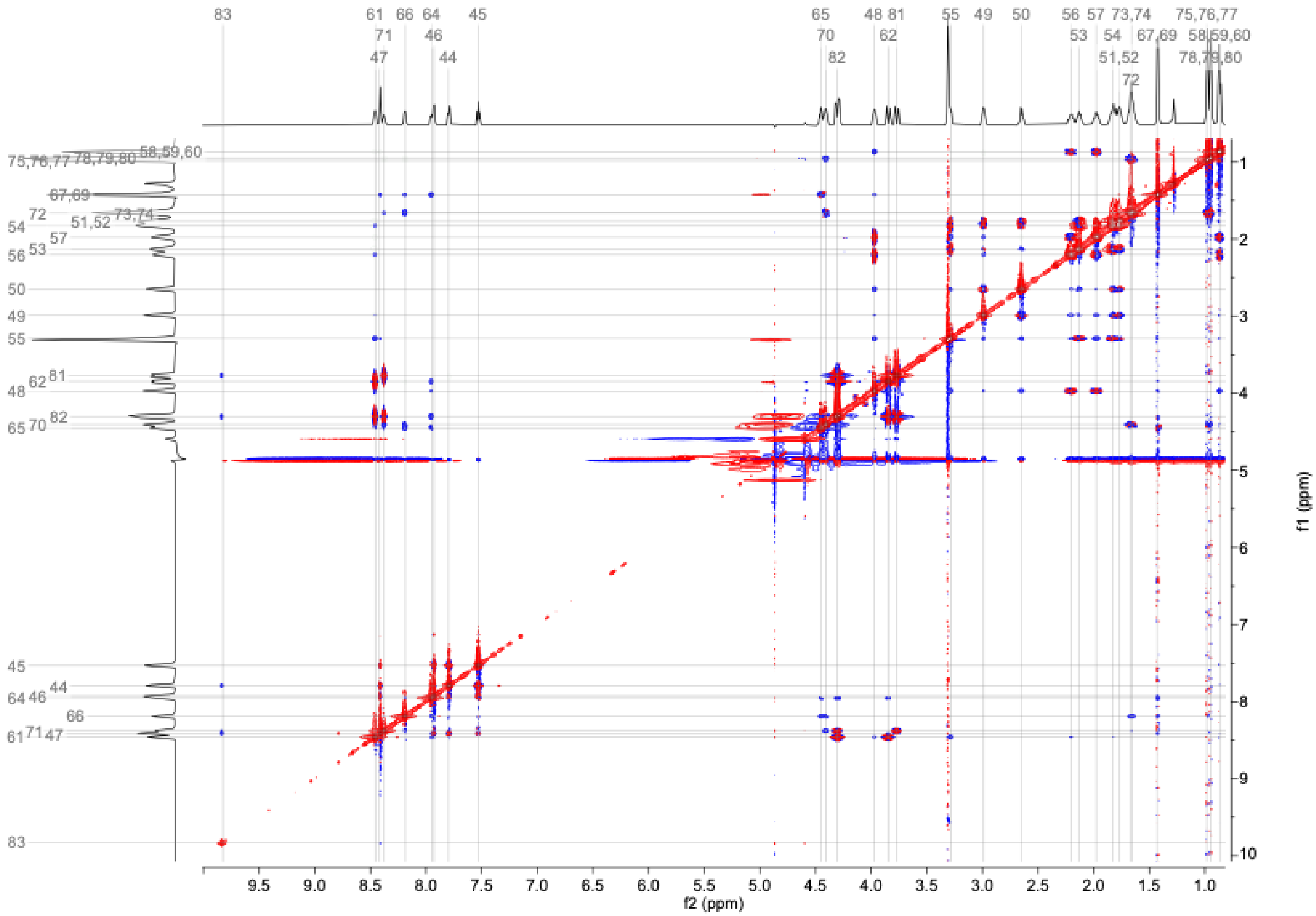

Figure S81. EASY-ROESY spectrum of cyclic peptide **CP3** at 300 K in methanol-*d3* at 600 MHz.. Solvent signal has been removed in post-processing. NS=32, d1 = 1.3 s, SW 11.9 (F2) x 11.9 (F1), TD 4k x 1k, States-TPPI, mixing time = 80 ms.

SI.16.4 Spectra for CP4

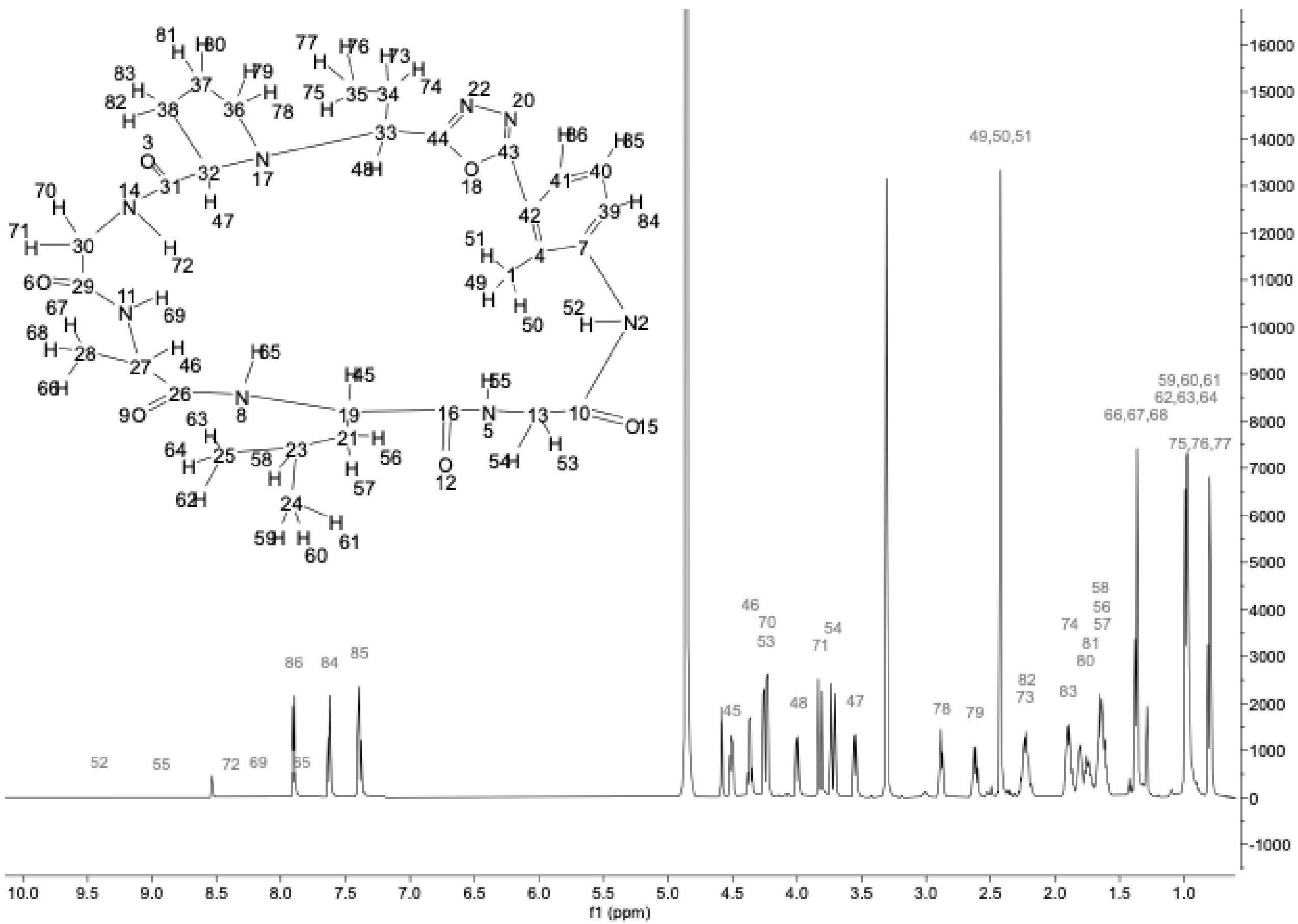

Figure S82. Proton spectrum of cyclic peptide **CP4** at 300 K in methanol-*d4* at 600 MHz. NS =32, d1= 5 s, AQ = 0.4 s, SW = 16.6 ppm, TD = 8k.

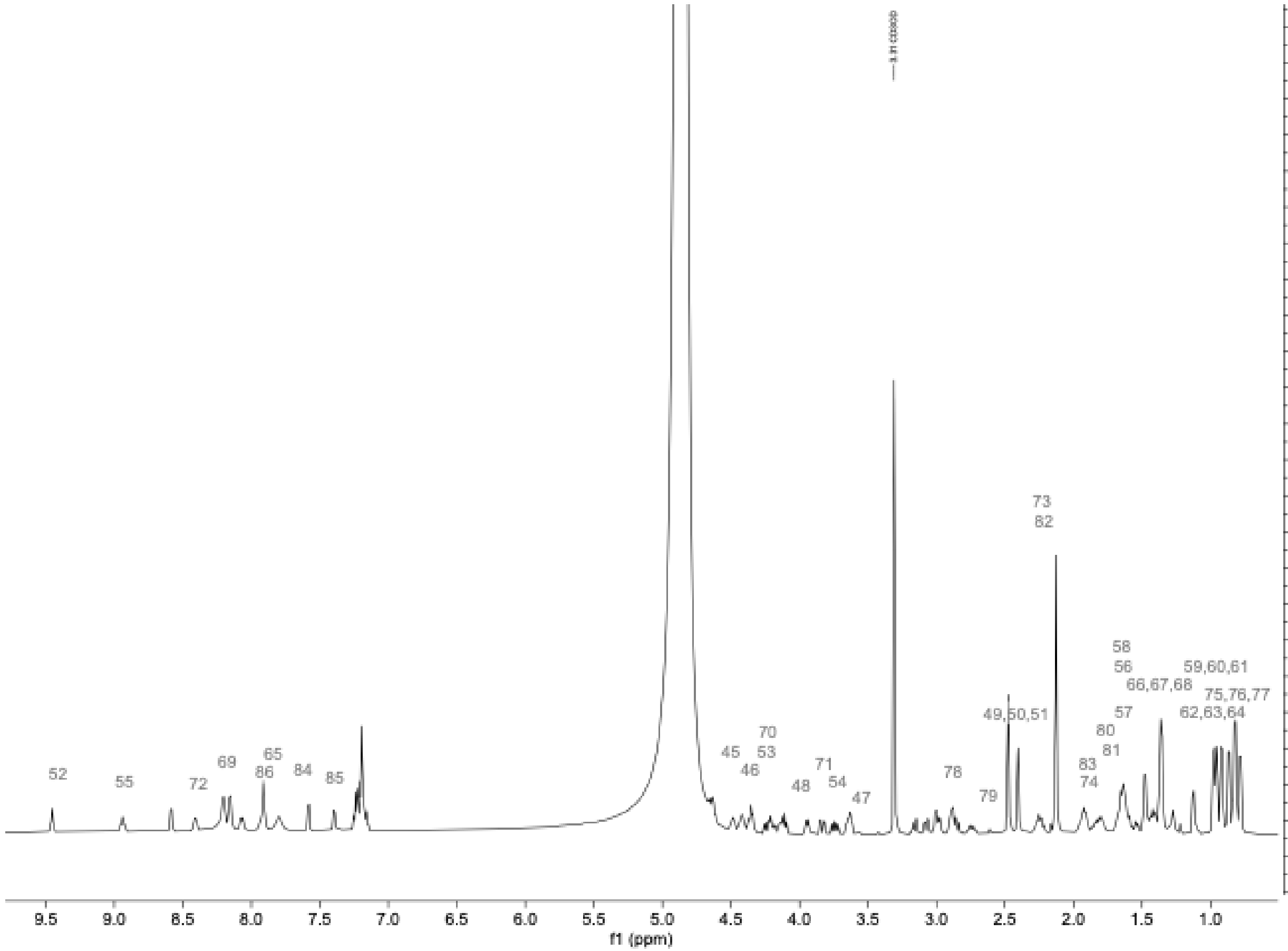

Figure S83. Proton spectrum of cyclic peptide **CP4** at 300 K in anisotropic methanol-*d3*/AAKLVFF at 600 MHz. NS =32, d1= 5 s, AQ = 0.4 s, SW = 16.6 ppm, TD = 8k.

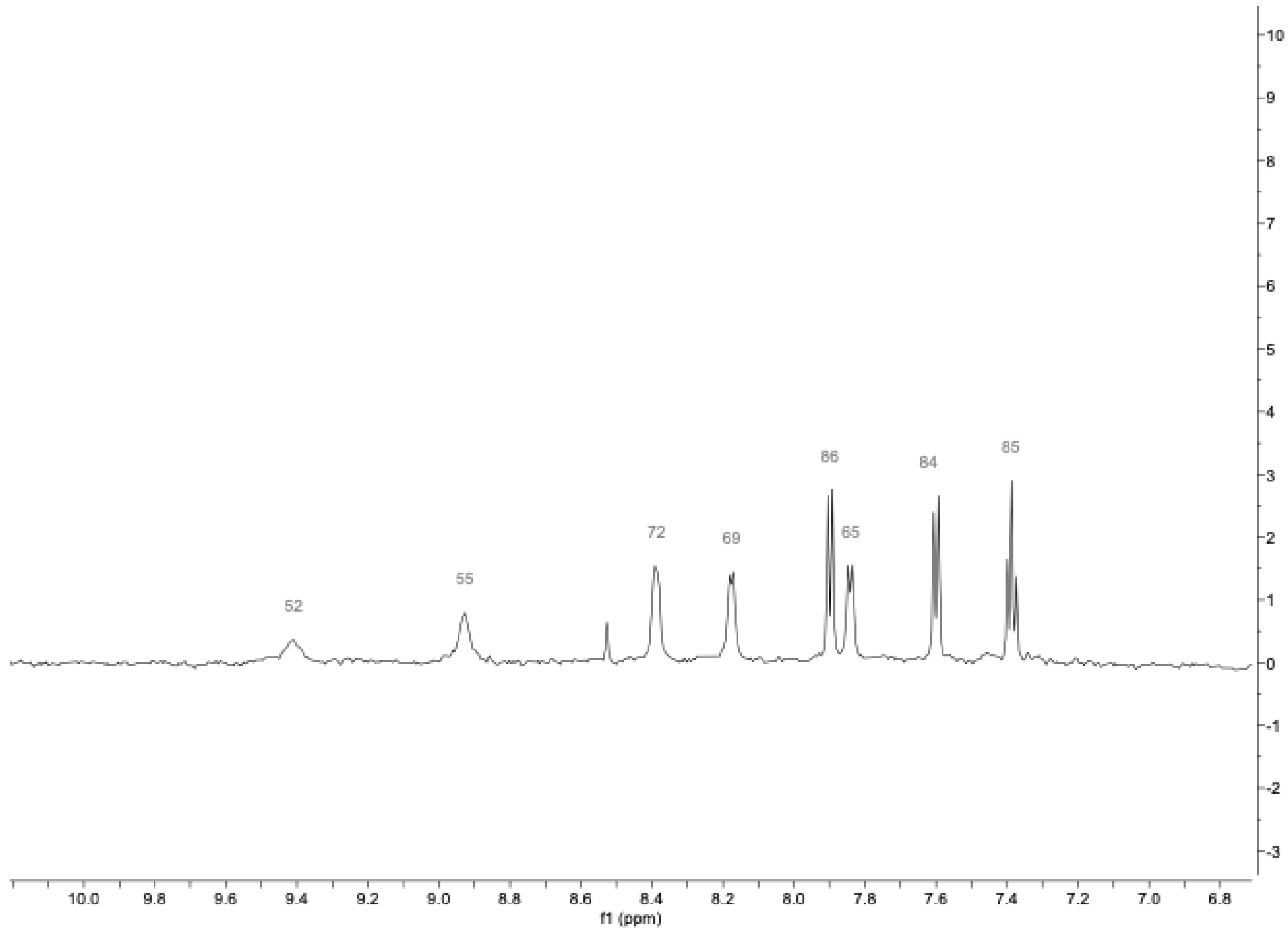

Figure S84. Excerpt of the amide region [7.00 ppm – 10.00 ppm] of the proton spectrum of cyclic peptide **CP4** at 300 K in methanol-*d3* at 600 MHz. NS =32, d1= 5 s, AQ = 0.4 s, SW = 16.6 ppm, TD = 8k.

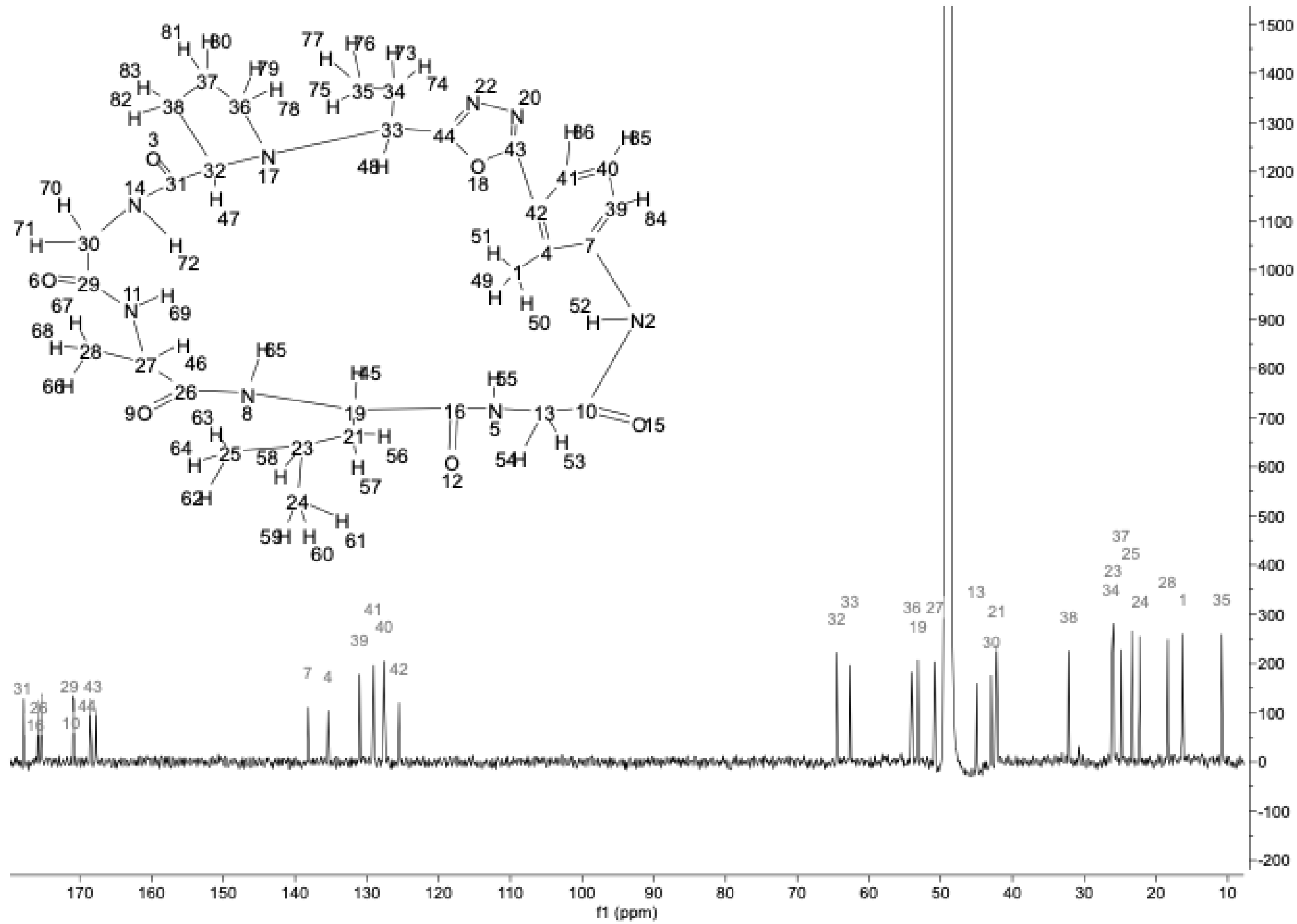

Figure S85. Carbon spectrum of cyclic peptide **CP4** at 300 K in methanol-*d4* at 600 MHz. NS =1024 (TD0 =14), d1= 2.5 s, AQ = 2.1 s, SW = 207 ppm, TD = 128k, 5.0 Hz LB.

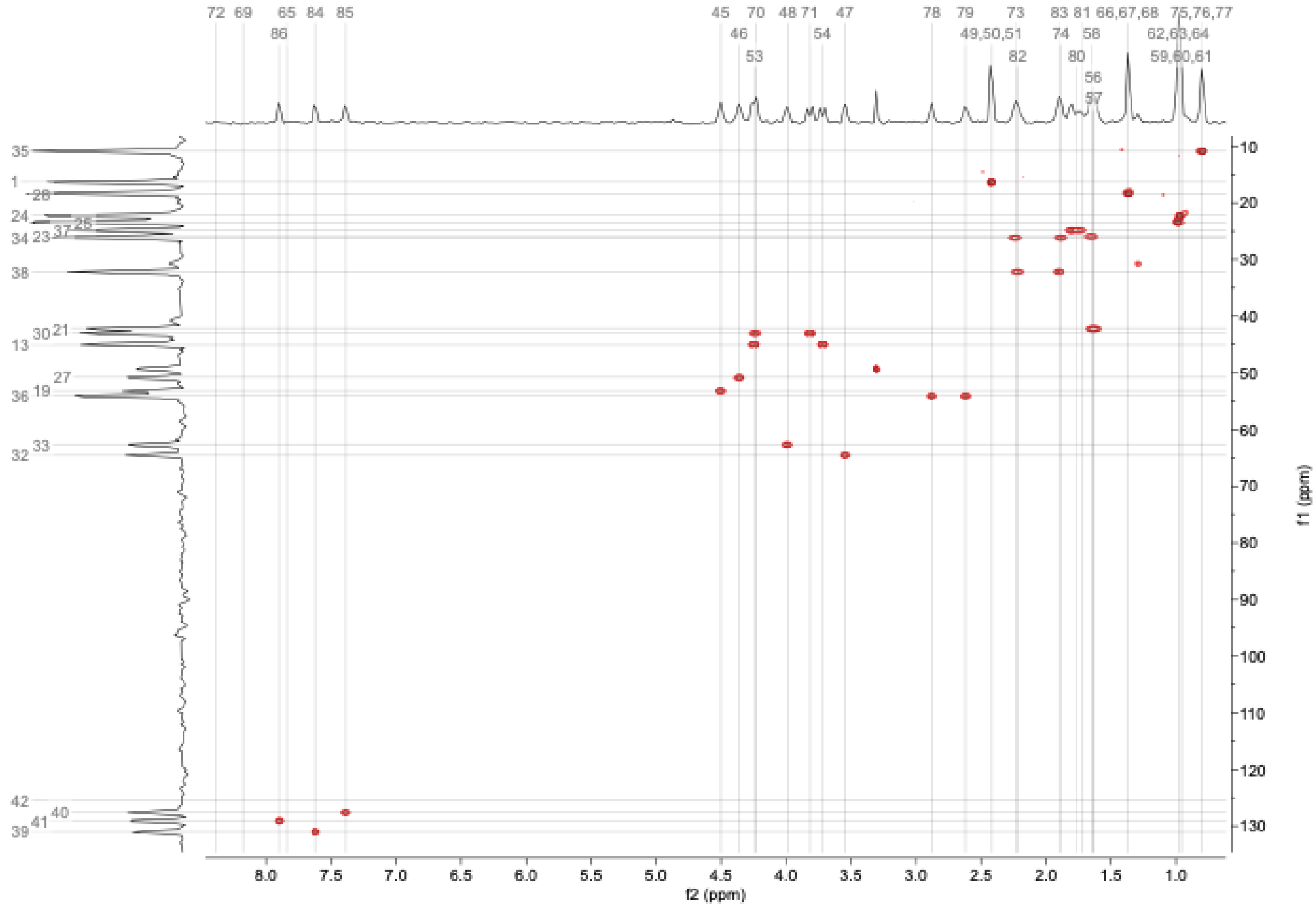

Figure S86. HSQC spectrum of cyclic peptide **CP4** at 300 K in methanol-*d4* at 600 MHz. NS=12, d1 = 1.3 s, SW 10 (F2) x 138 (F1), TD 1k x 512, echo-antiecho.

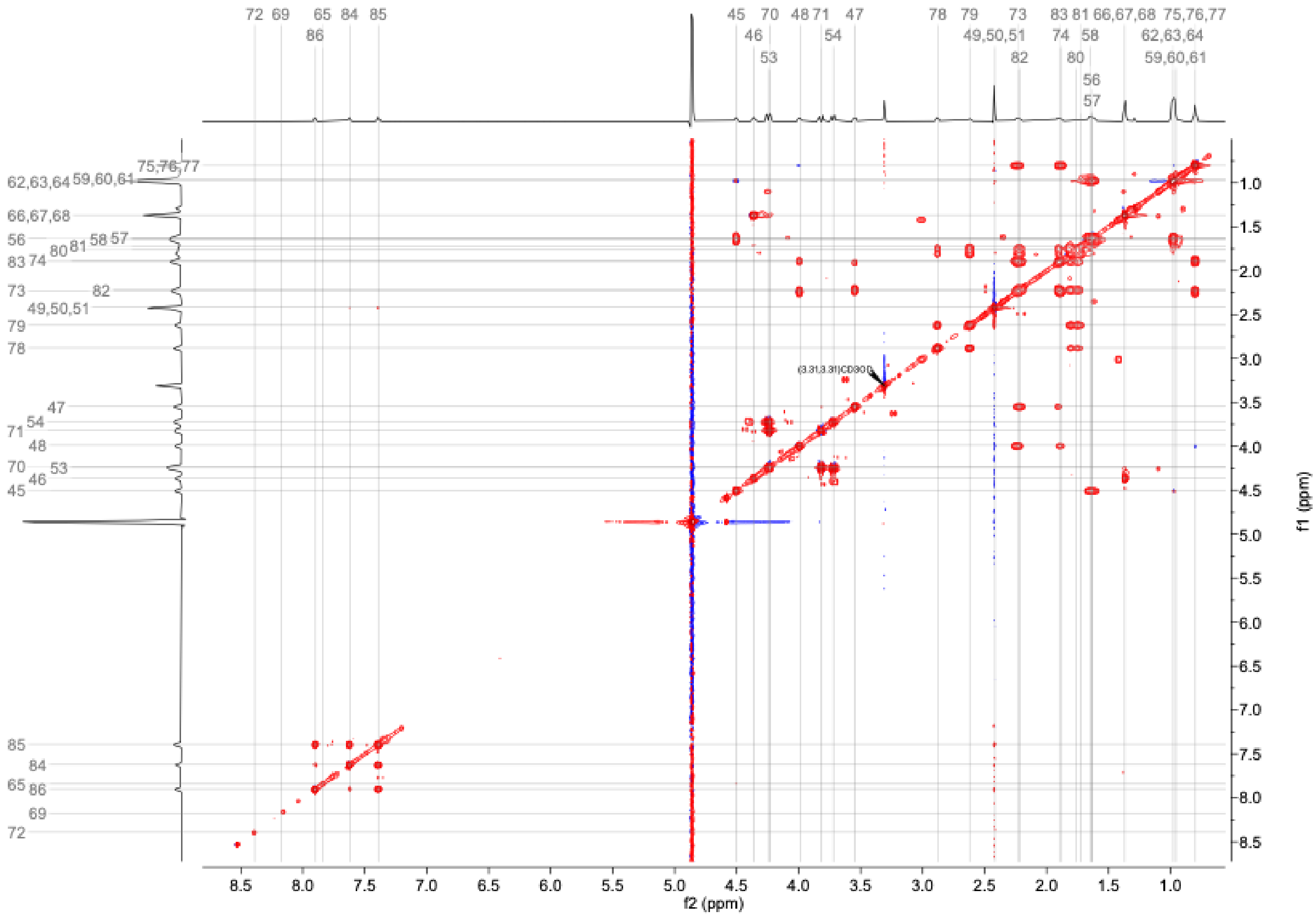

Figure S87. CLIP-COSY spectrum of cyclic peptide 067 at **CP4** at 300 K in methanol-*d4* at 600 MHz. NS=8, d1= 1.3s, SW 16.7 (F2) x 13.9 (F1), TD 4k x 1k, States-TPPI.

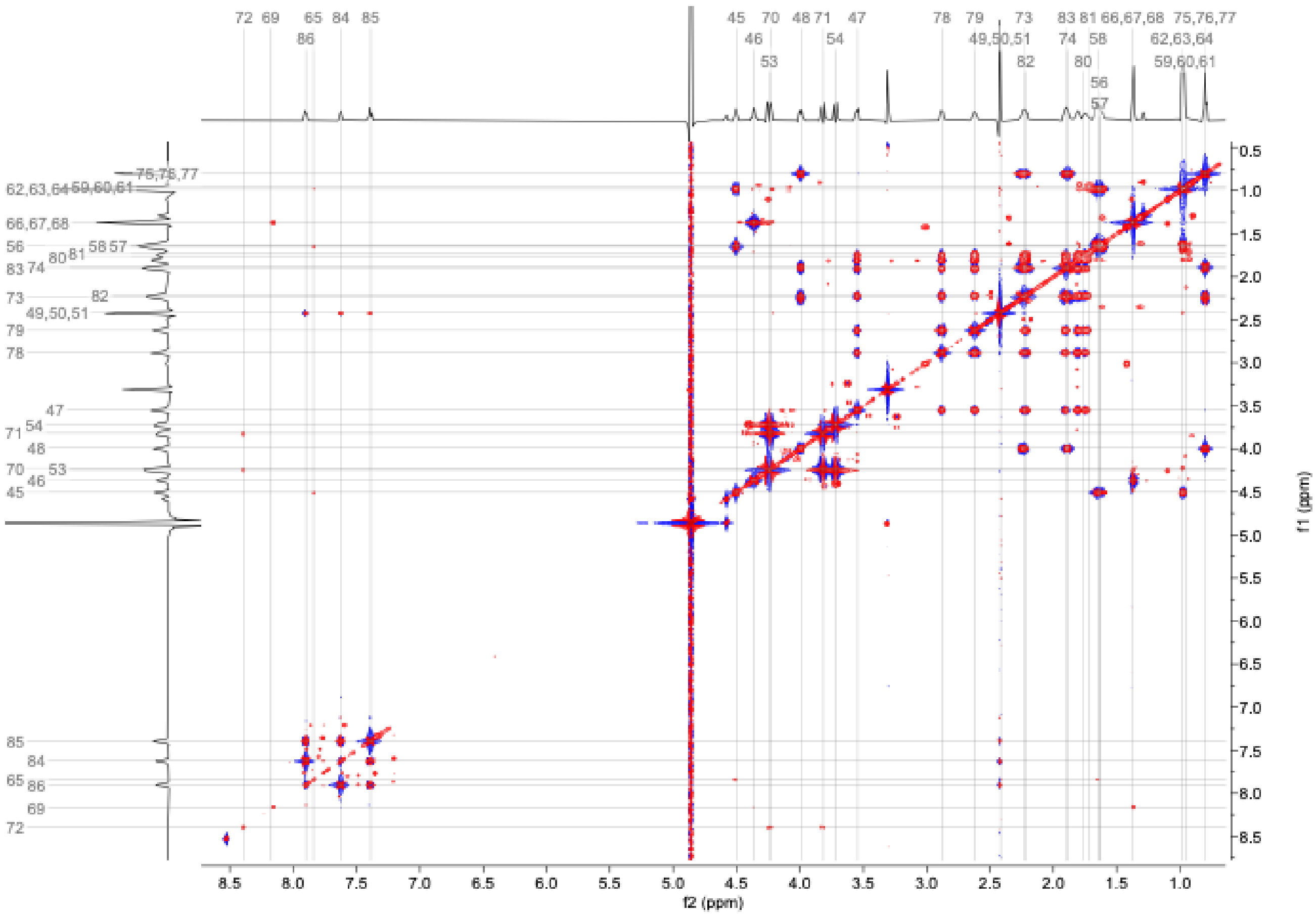

Figure S88. TOCSY spectrum of cyclic peptide **CP4** at 300 K in methanol-*d4* at 600 MHz. NS=8, d1= 1.3s, SW 16.7 (F2) x 13.9 (F1), TD 4k x 1k, States-TPPI, 80 ms mixing time.

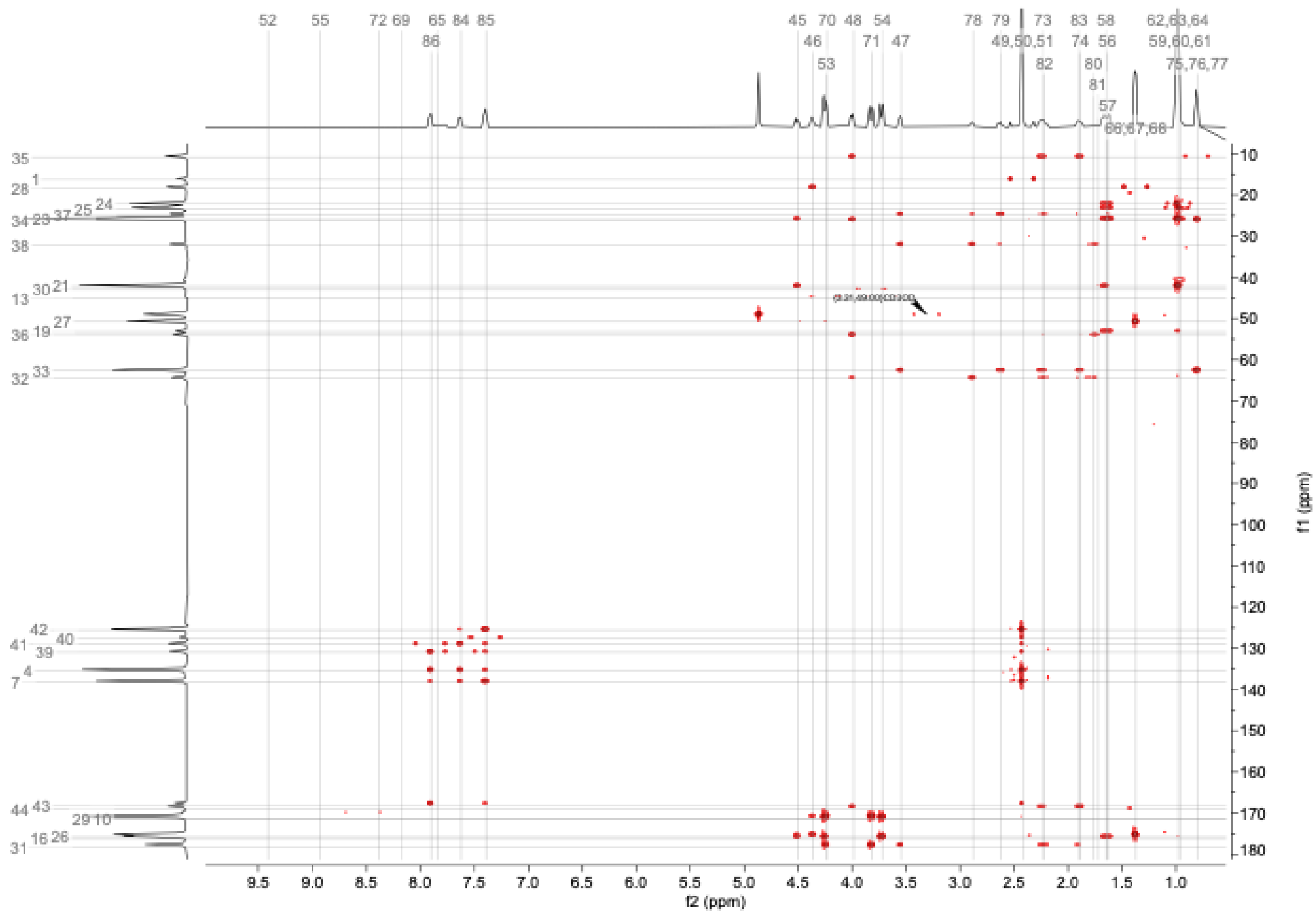

Figure S89. HMBC spectrum of cyclic peptide **CP4** at 300 K in methanol-*d4* at 600 MHz. NS=32, d1= 1.3s, SW 16.6 (F2) x 207 (F1), TD 4k x 1k, delay for long range coupling 70 ms, echo-antiecho.

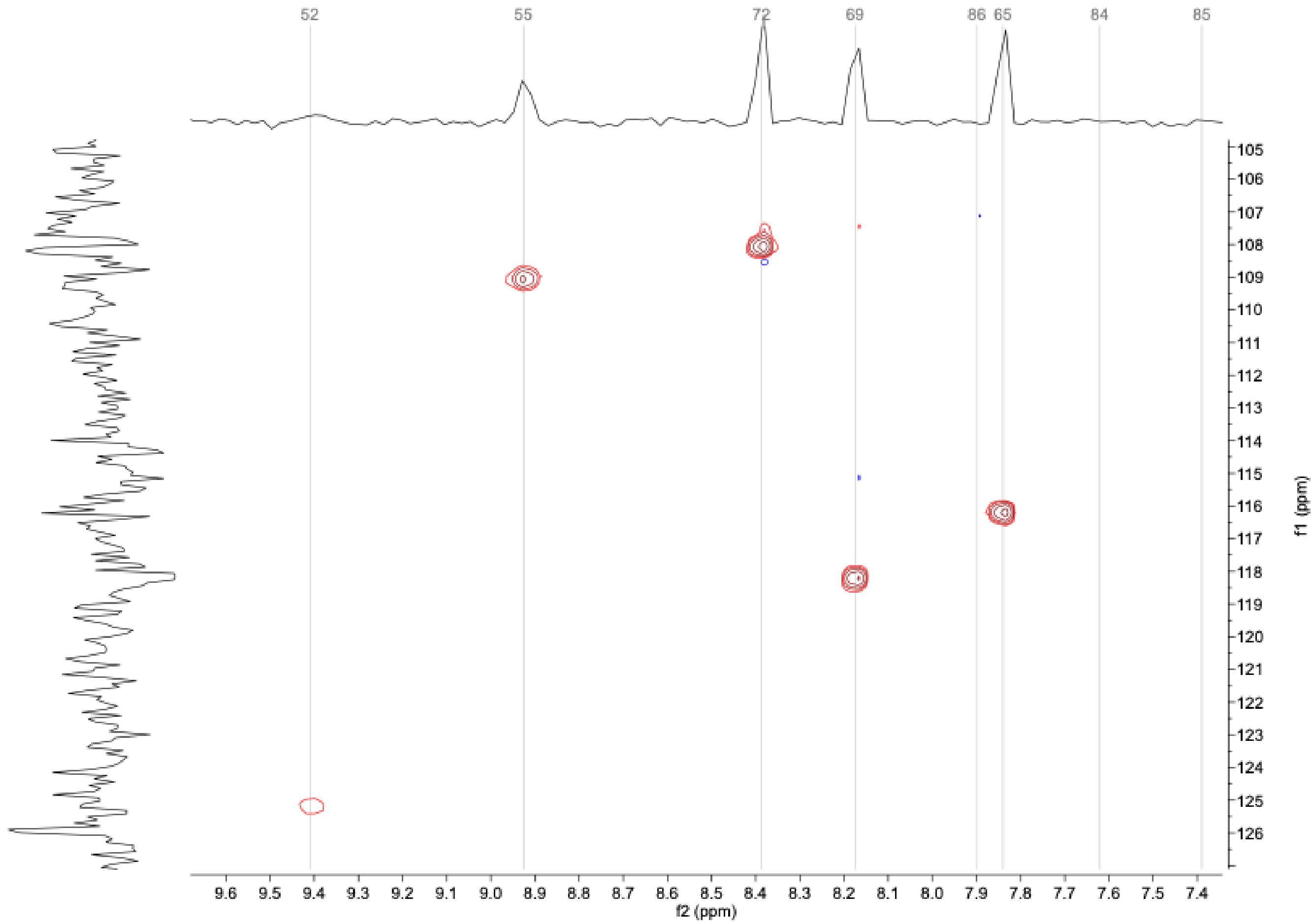

Figure S90. ^{15}N-HSQC spectrum of cyclic peptide **CP4** at 300 K in methanol-*d3* at 600 MHz. NS=48, d1= 1.3s, SW 10 (F2) x 49.5 (F1), TD 1k x 256, States-TPPI, WaterGATE.

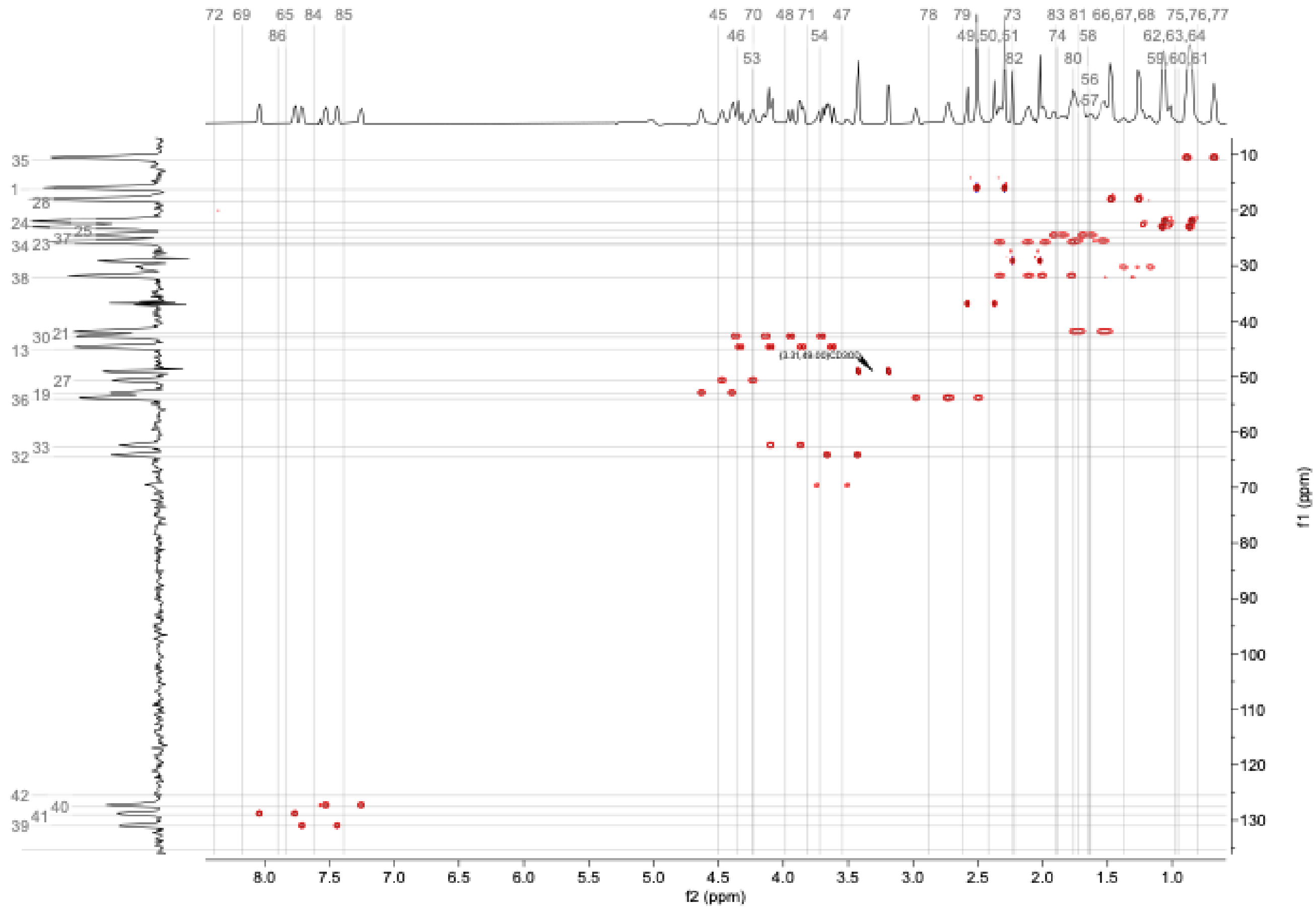

Figure S91. CLIP-HSQC spectrum of cyclic peptide **CP4** at 300 K in methanol-*d3* at 600 MHz. NS=24, d1 = 1.3 s, SW 10 (F2) x 150 (F1), TD 4k x 512, echo-antiecho, coupling in F2.

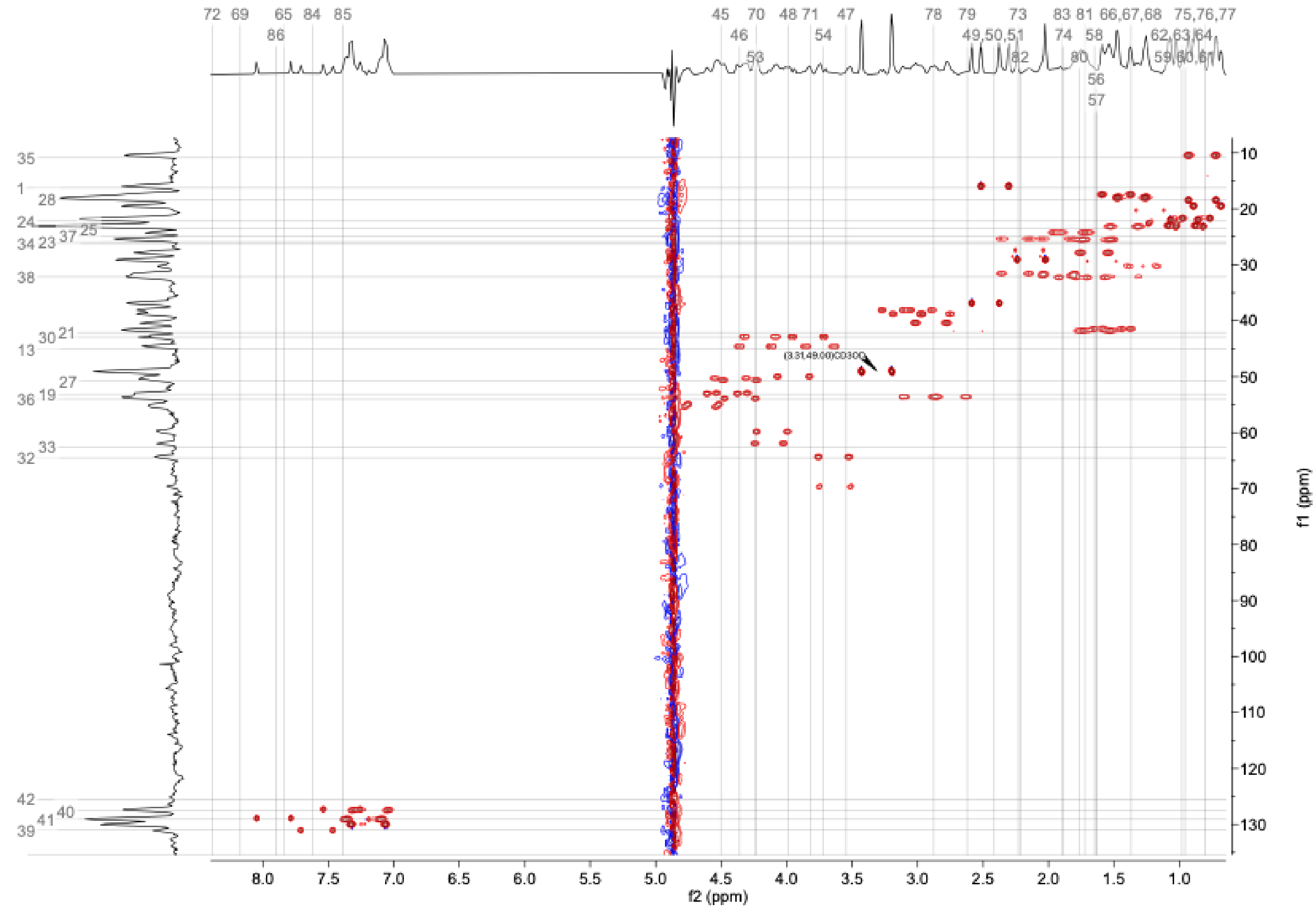

Figure S92. CLIP-HSQC spectrum of cyclic peptide **CP4** at 300 K in methanol-*d3*/AAKLVFF at 600 MHz. NS=32, d1 = 1.3 s, SW 10 (F2) x 150 (F1), TD 4k x 512, echo-antiecho, coupling in F2.

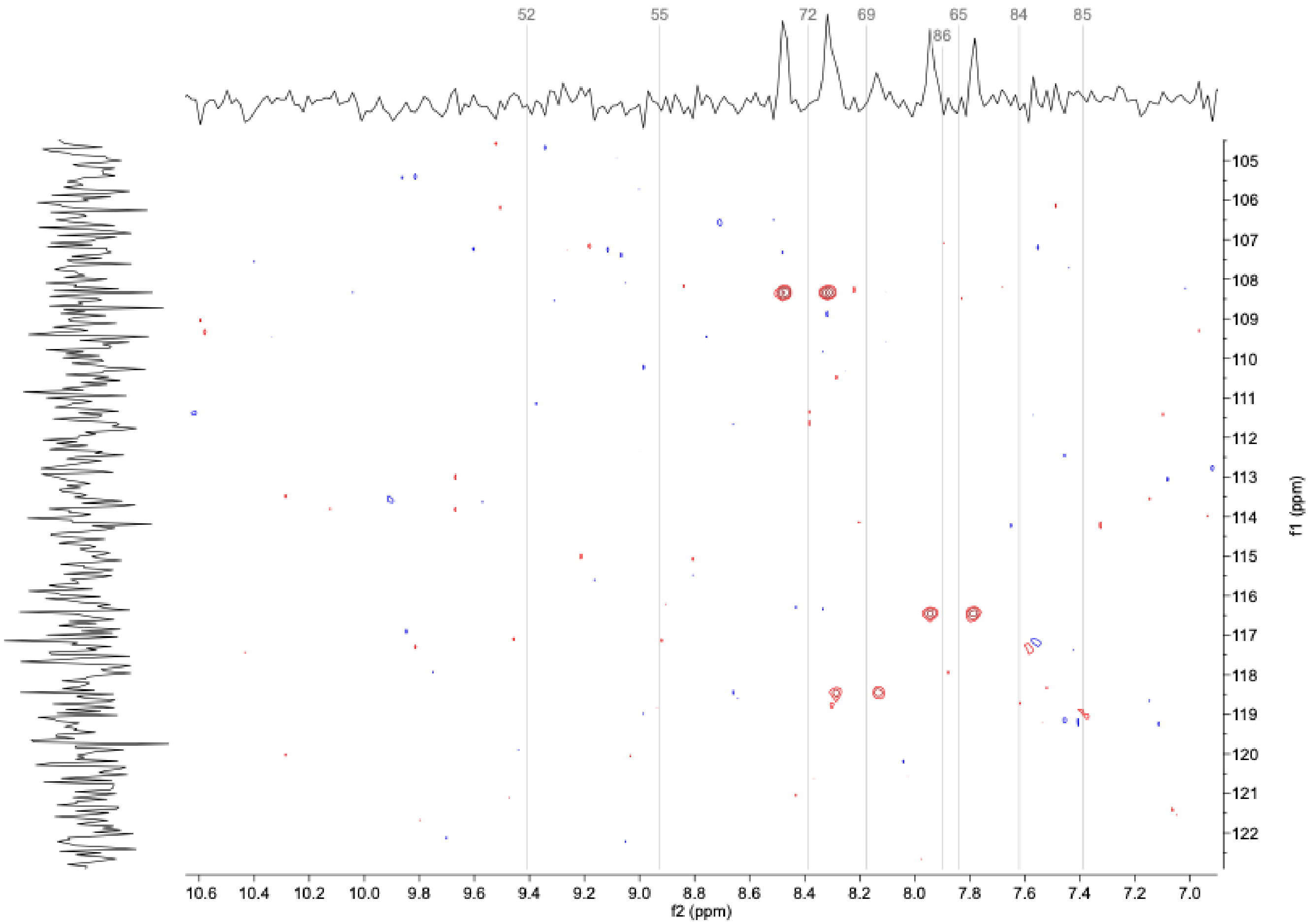

Figure S93. ^{15}N-CLIP-HSQC spectrum of cyclic peptide **CP4** at 300 K in methanol-*d3* at 600 MHz. NS=320, d1 = 1.3 s, SW 3 (F2) x 20 (F1), TD 2k x 128, echo-antiecho, coupling in F2. WaterGATE.

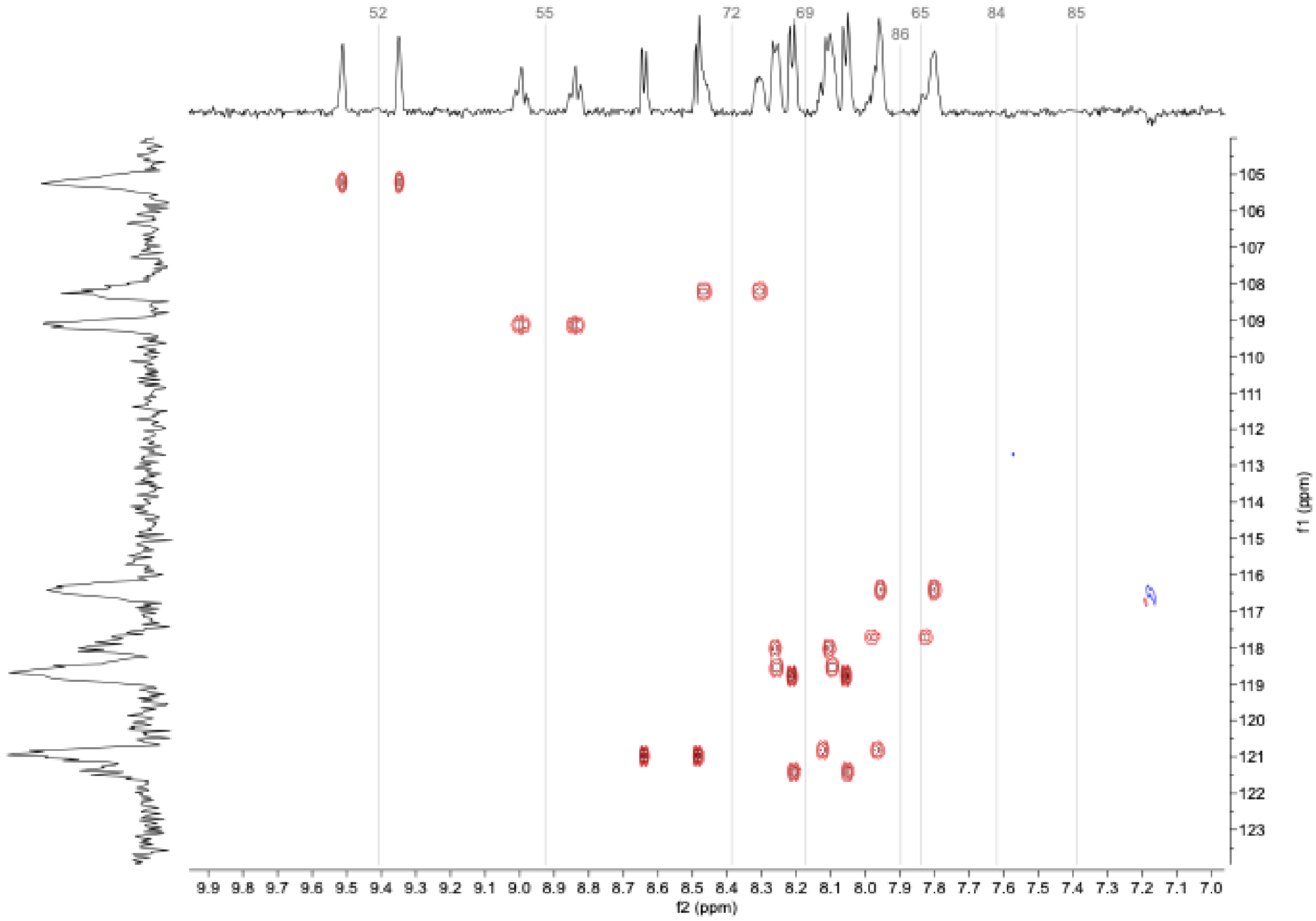

Figure S94. ^{15}N-CLIP-HSQC spectrum of cyclic peptide **CP4** at 300 K in methanol-*d3*/AAKLVFF at 600 MHz. NS=312, d1 = 1.3 s, SW 3 (F2) x 20 (F1), TD 2k x 128, echo-antiecho, coupling in F2. WaterGATE.

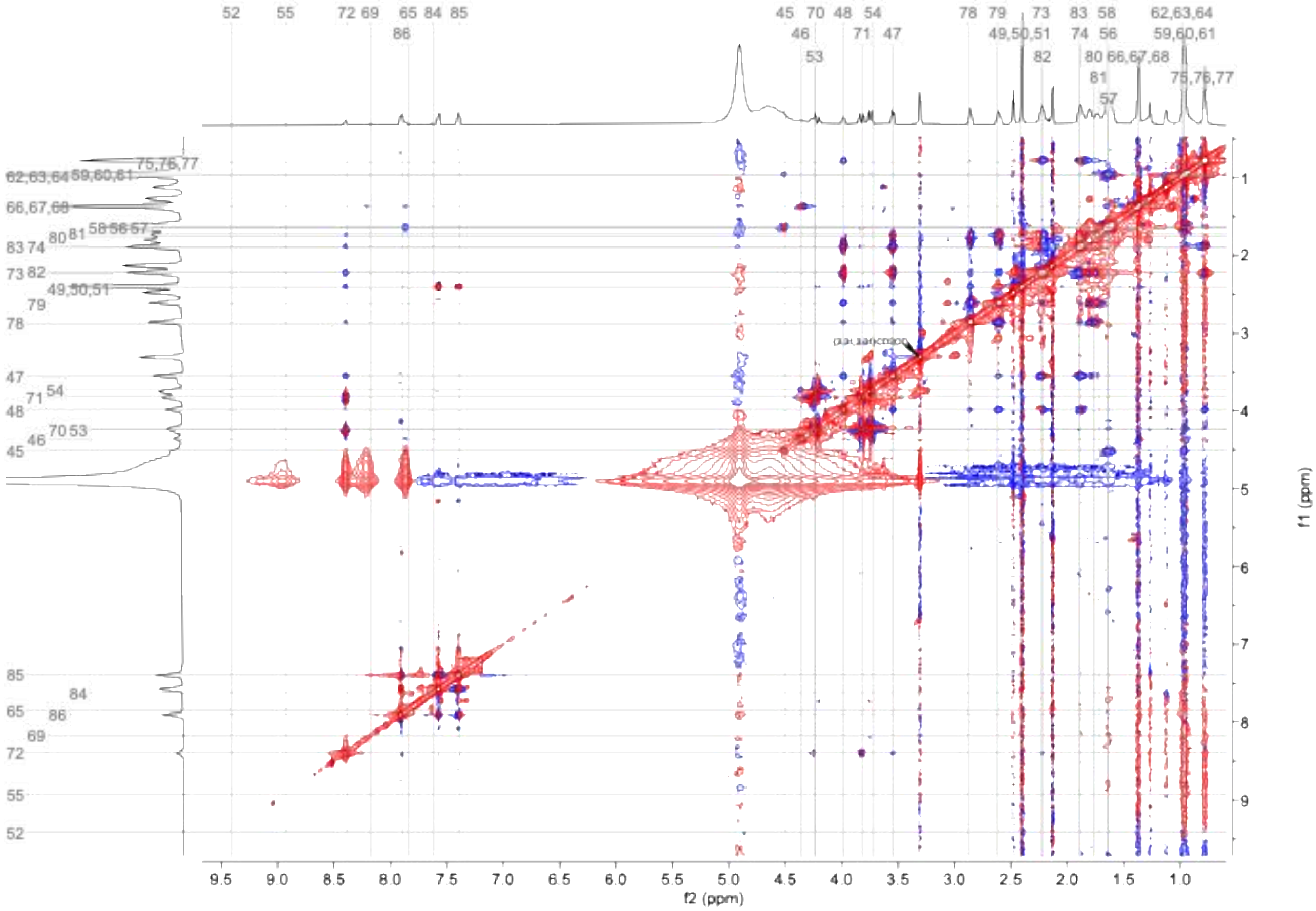

Figure S95. ROESY spectrum of cyclic peptide **CP4** at 300 K in methanol-*d3* at 600 MHz. NS=8, d1 = 1.3 s, SW 10 (F2) x 10 (F1), TD 2k x 512, States-TPPI, mixing time = 80 ms.

SI.16.5 Spectra for CP5

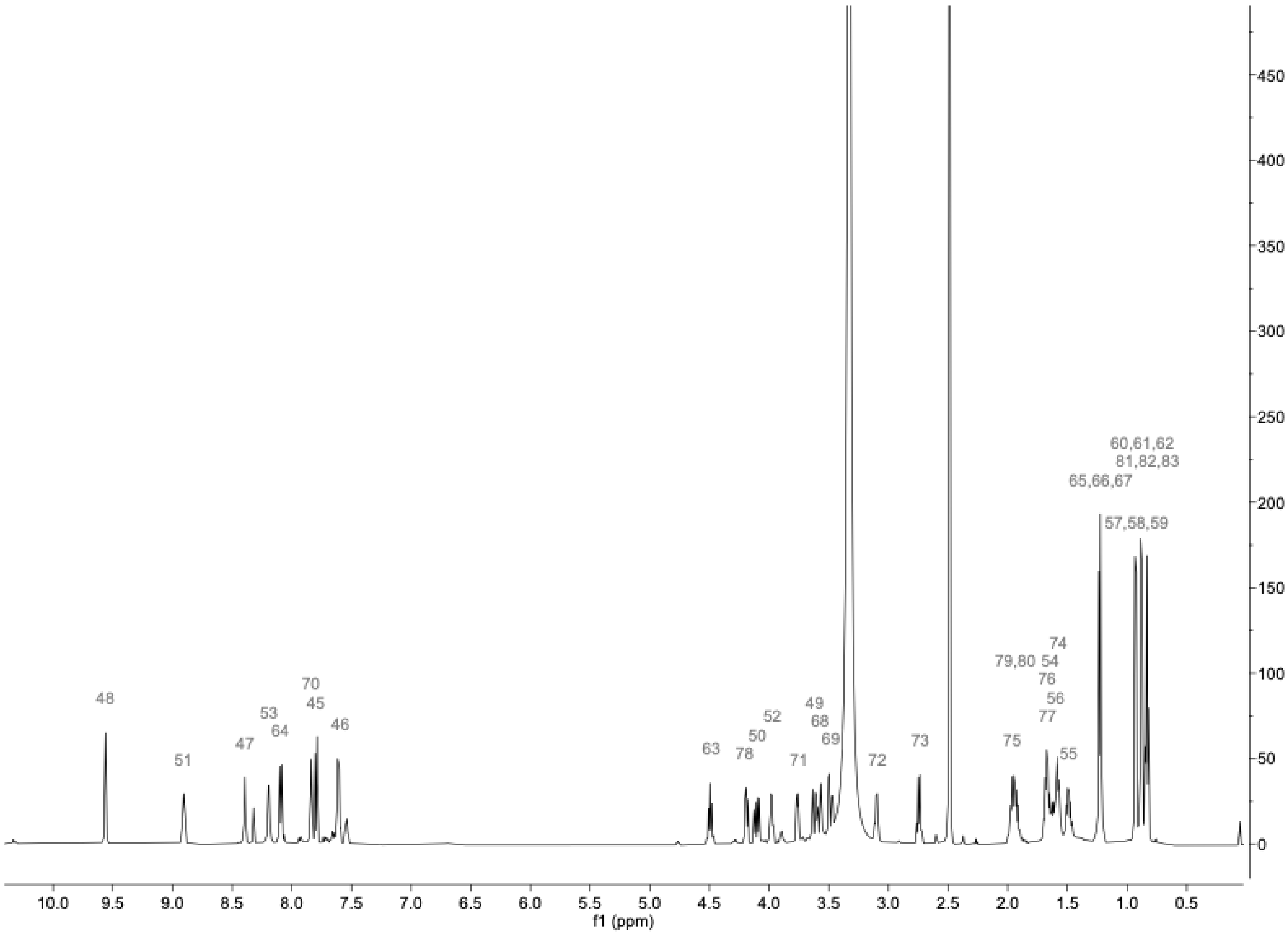

Figure S96. Proton spectrum of cyclic peptide **CP5** at 300 K in DMSO-*d6* at 600 MHz. NS =32, d1= 5 s, AQ = 0.4s, SW = 16.6 ppm, TD = 8k.

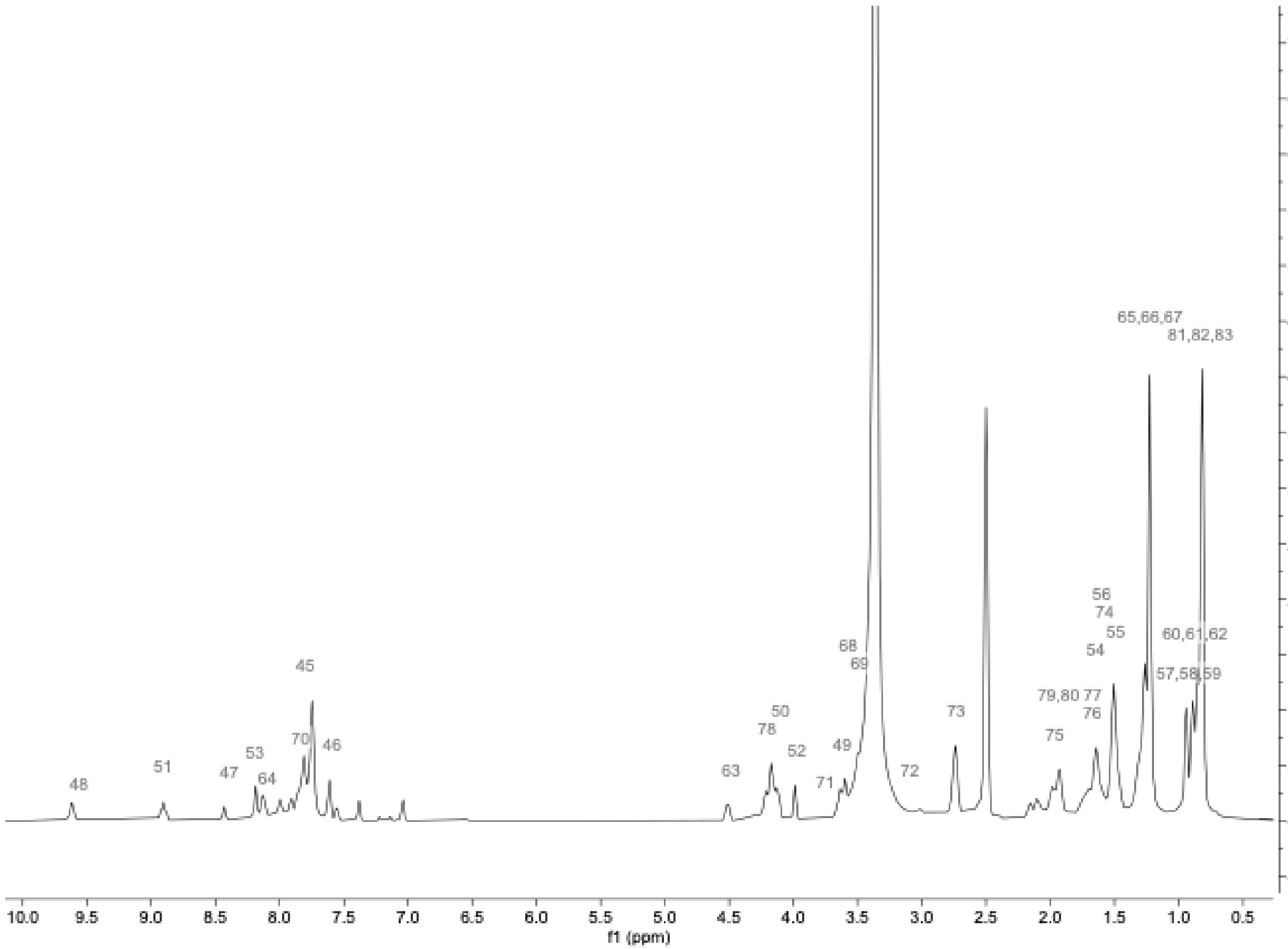

Figure S97. Proton spectrum of cyclic peptide **CP5** at 300 K in anisotropic DMSO-*d6*/OPA at 600 MHz. NS =32, d1= 5 s, AQ = 0.4s, SW = 16.6 ppm, TD = 8k.

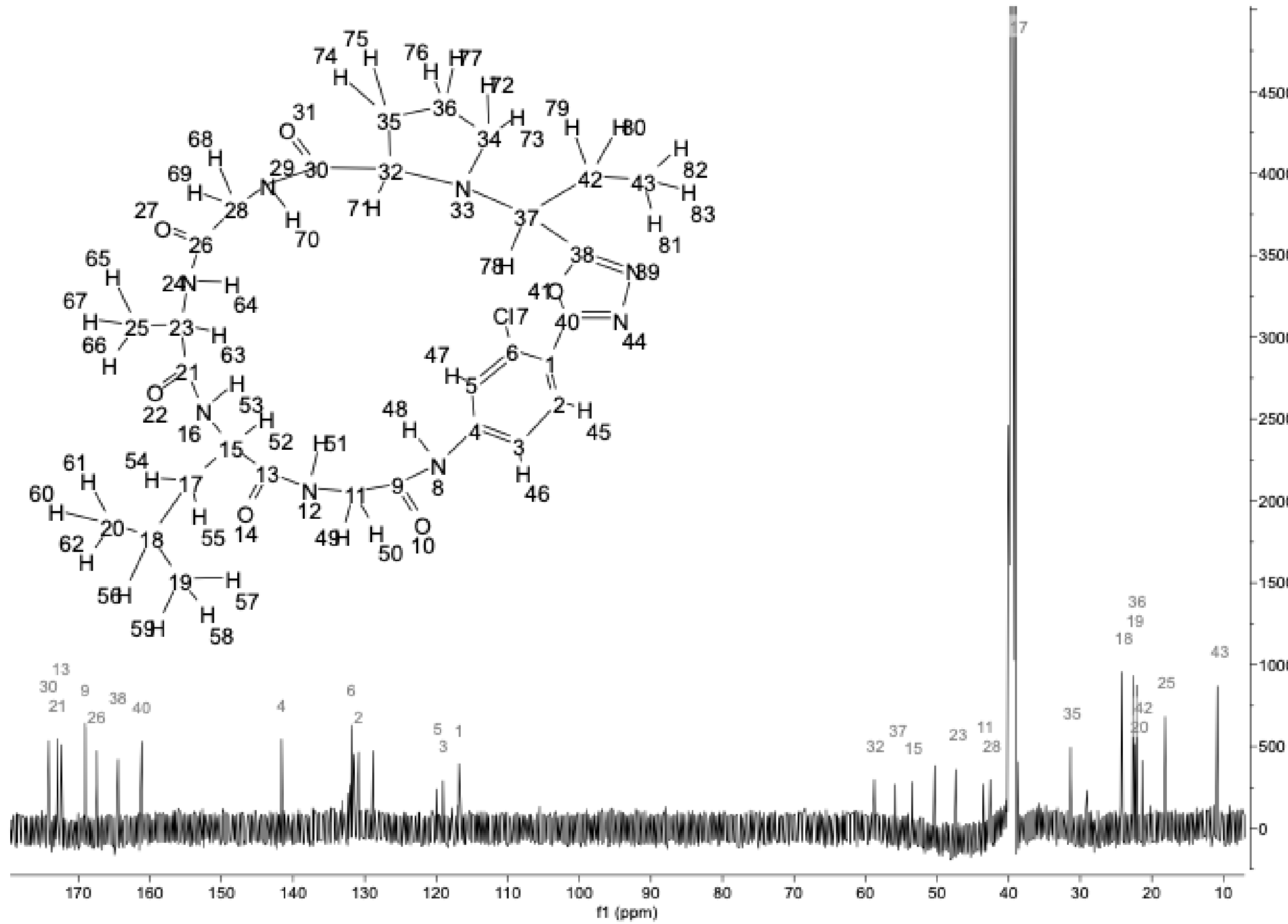

Figure S98. Carbon spectrum of cyclic peptide **CP5** at 300 K in DMSO-*d6* at 600 MHz. NS =1024 (TD0 =30), d1= 2.5 s, AQ = 2.1 s, SW = 207 ppm, TD = 128k, 5.0 Hz LB.

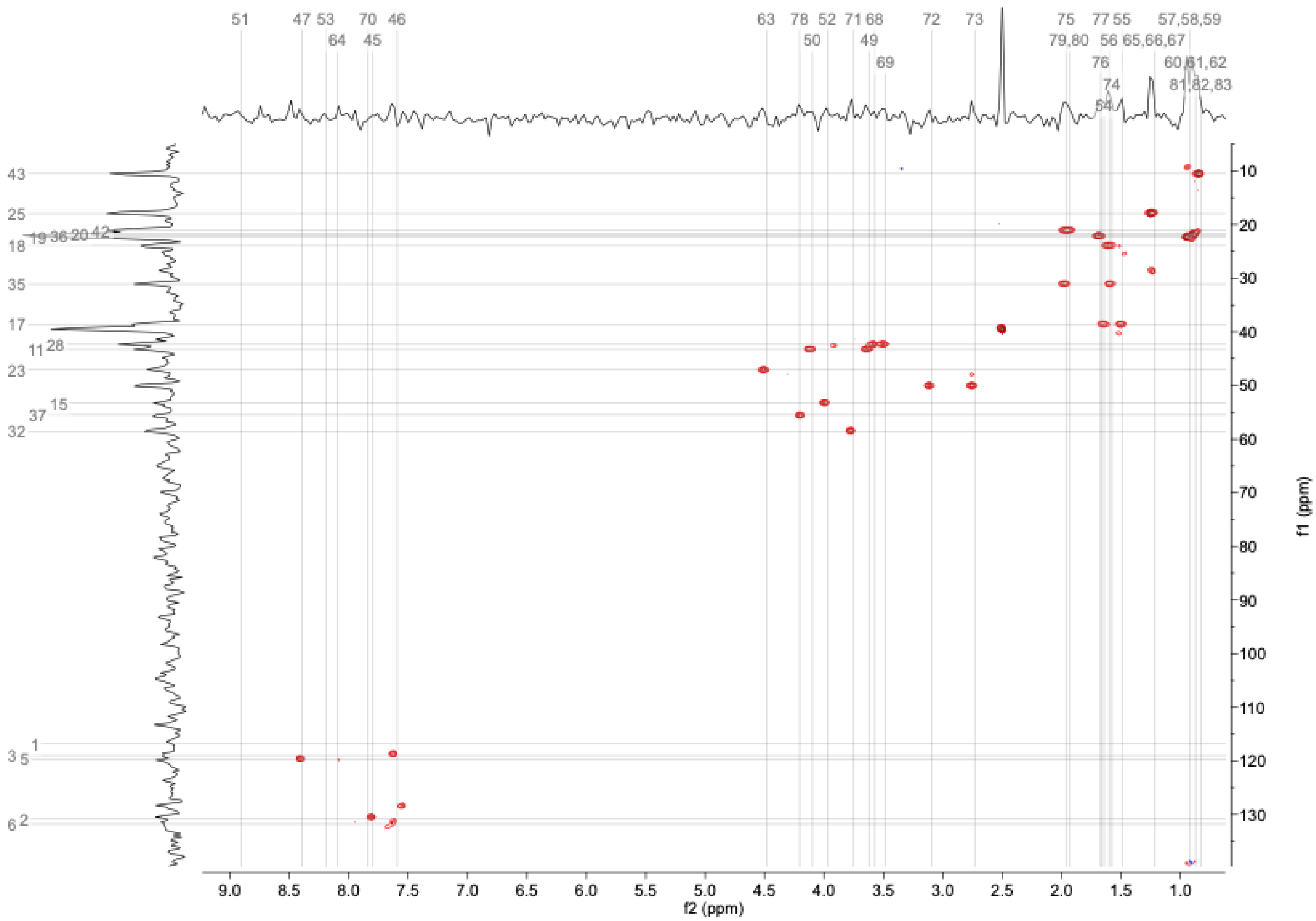

Figure S99. HSQC spectrum of cyclic peptide **CP5** at 300 K in DMSO-*d6* at 600 MHz. NS=12, d1 = 1.3 s, SW 12 (F2) x 160 (F1), TD 1k x 512, echo-antiecho.

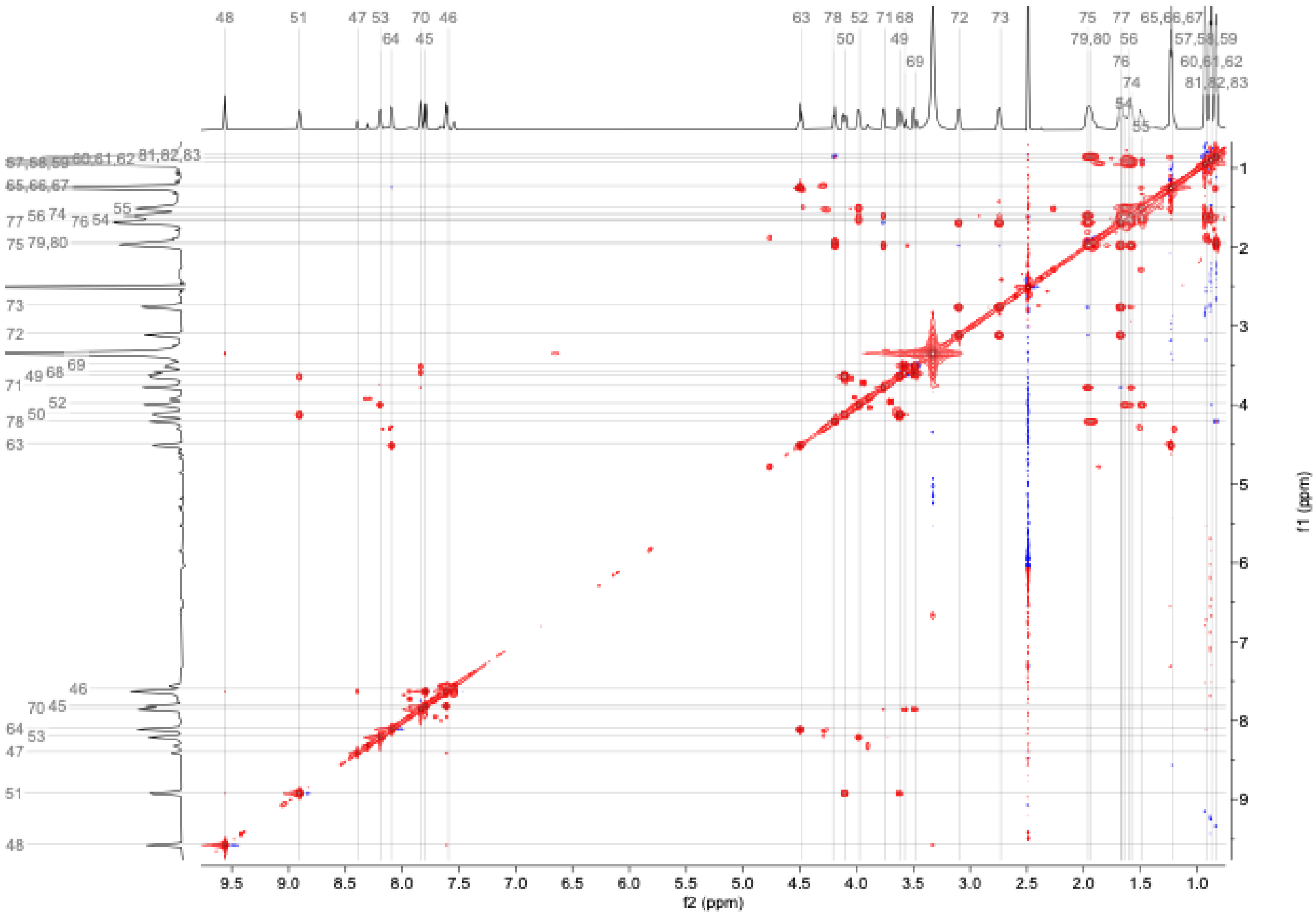

Figure S100. CLIP-COSY spectrum of cyclic peptide **CP5** at 300 K in DMSO-*d6* at 600 MHz. NS=8, d1= 1.3s, SW 14 (F2) x 14 (F1), TD 4k x 1k, States-TPPI.

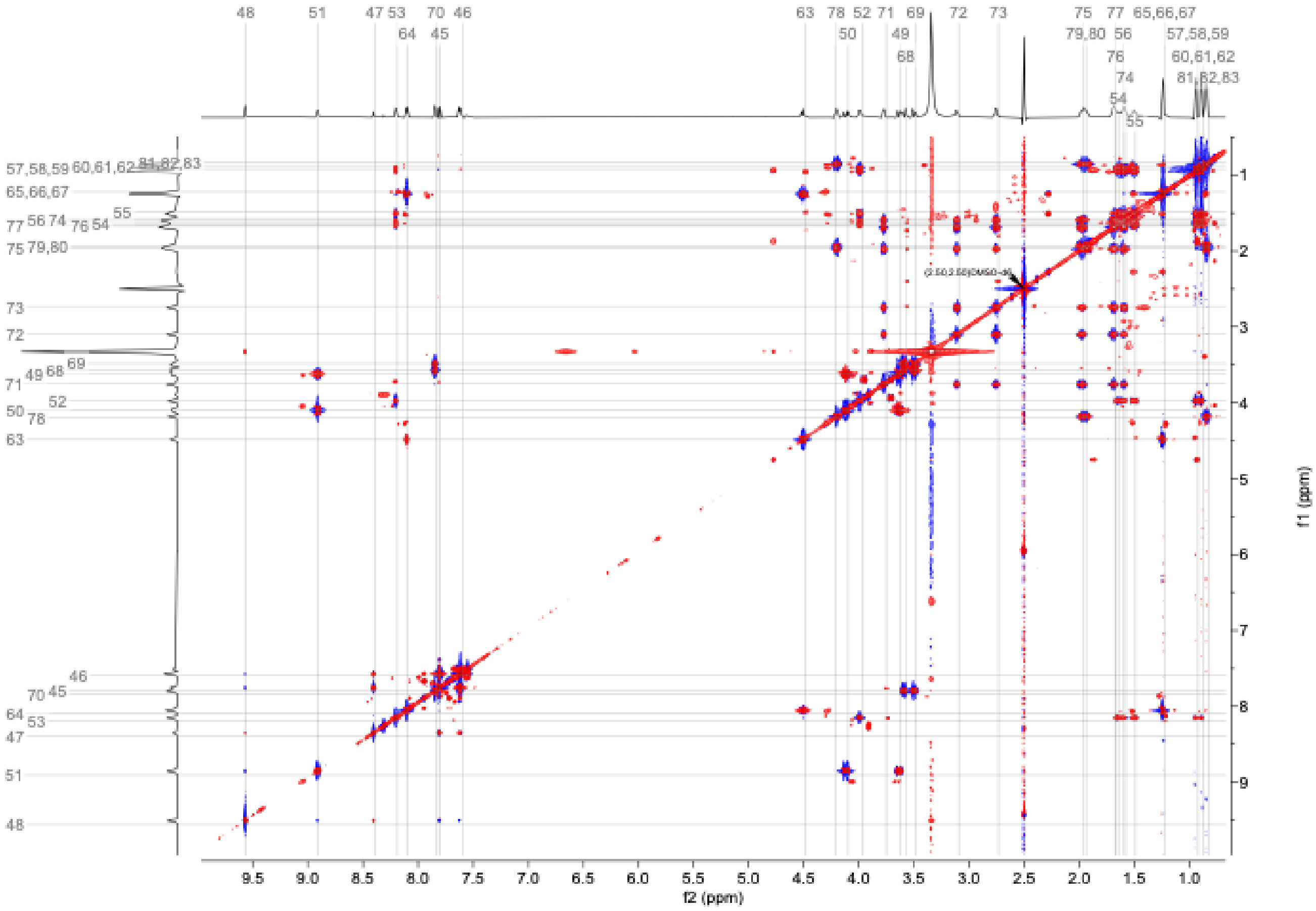

Figure S101. TOCSY spectrum of cyclic peptide **CP5** at 300 K in DMSO-*d6* at 600 MHz. NS=8, d1= 1.3s, SW 14 (F2) x 14 (F1), TD 4k x 1k, States-TPPI, 80 ms mixing time.

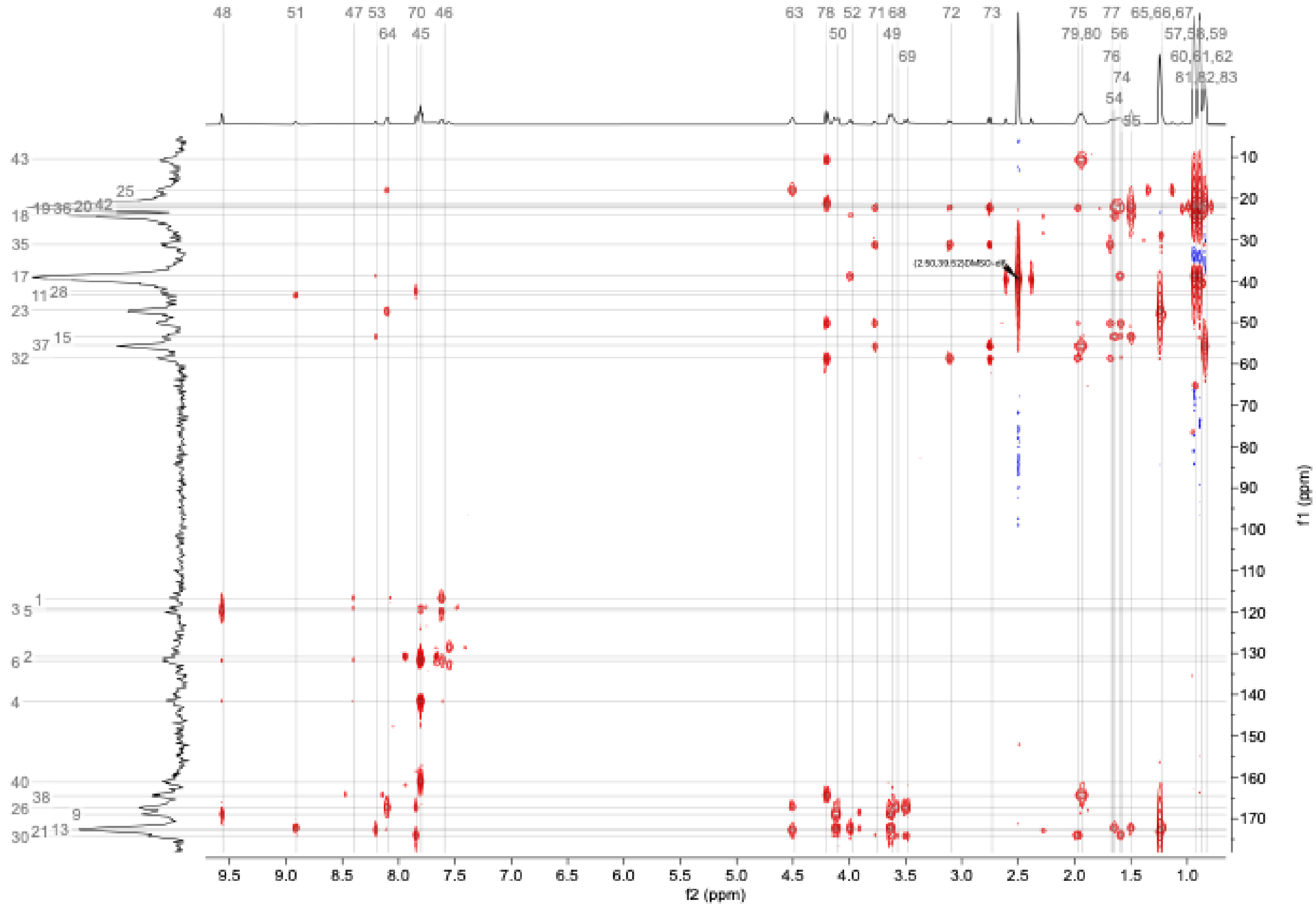

Figure S102. HMBC spectrum of cyclic peptide **CP5** at 300 K in DMSO-*d6* at 600 MHz. NS=32, d1= 1.3s, SW 16.6 (F2) x 207 (F1), TD 2k x 1k, delay for long range coupling 70 ms, echo-antiecho.

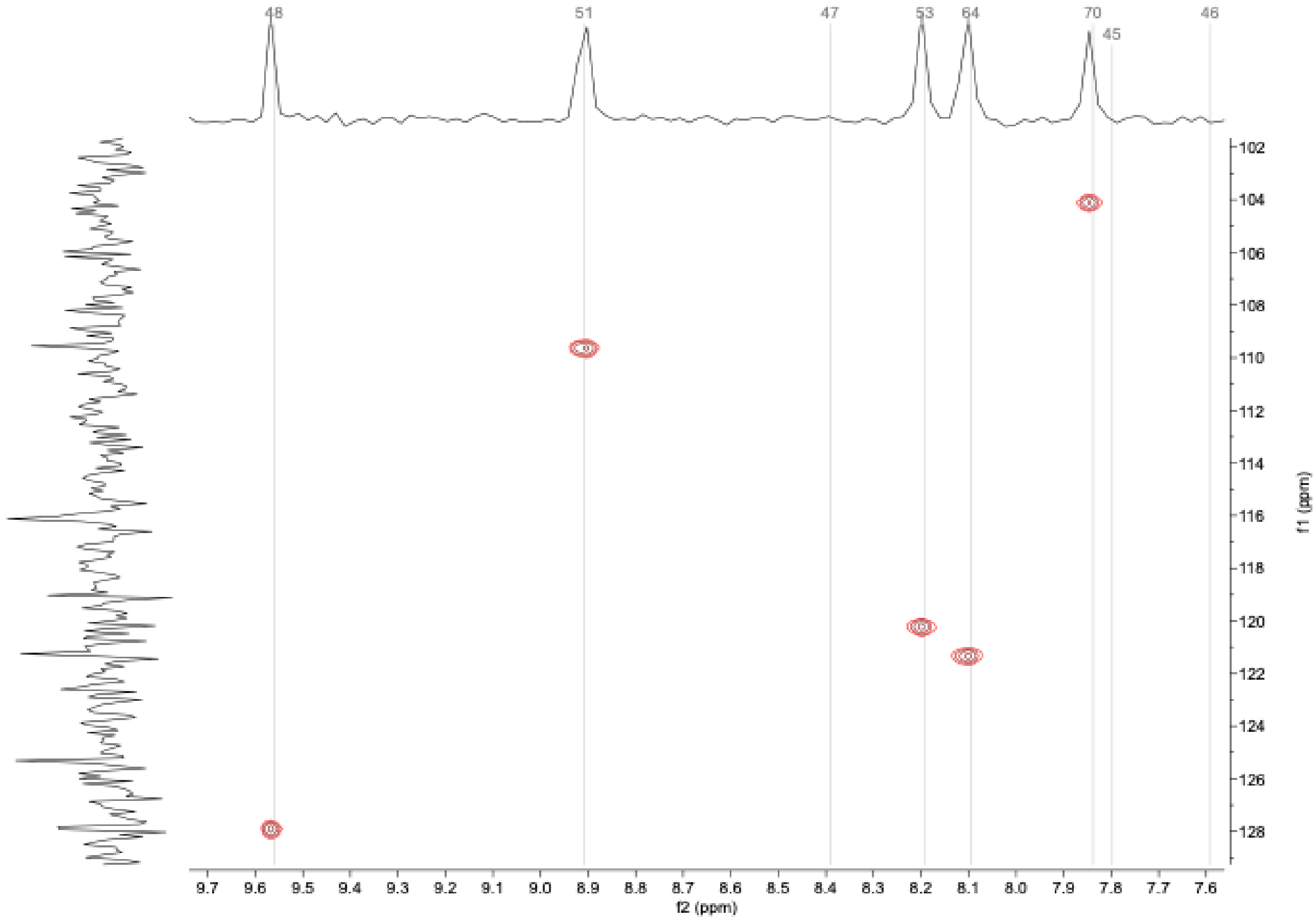

Figure S103. ^{15}N-HSQC spectrum of cyclic peptide **CP5** at 300 K in DMSO-*d6* at 600 MHz. NS=48, d1= 1.3s, SW 10 (F2) x 49.5 (F1), TD 1k x 256, States-TPPI, WaterGATE.

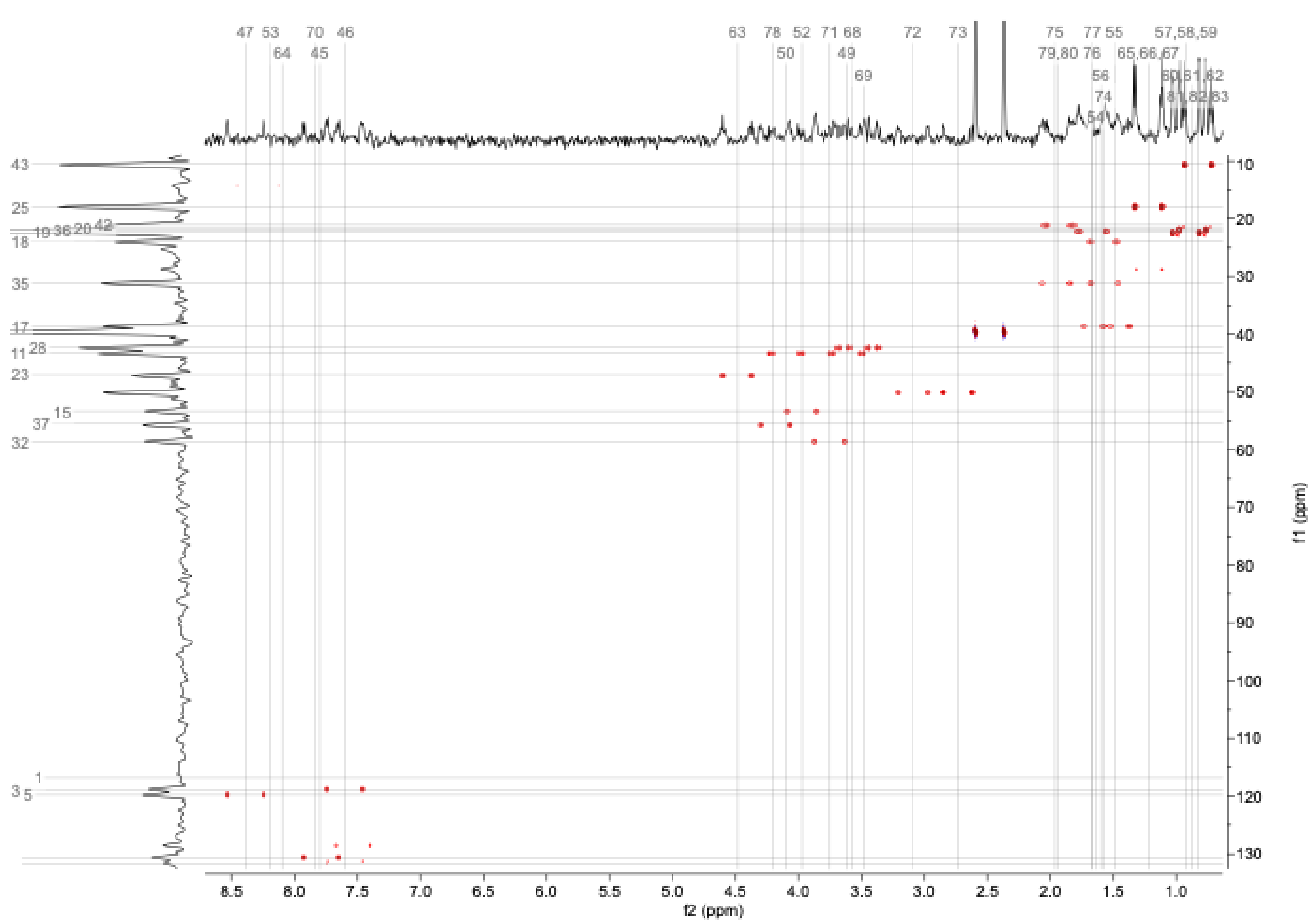

Figure S104. CLIP-HSQC spectrum of cyclic peptide **CP5** at 300 K in DMSO-*d6* at 600 MHz. NS=24, d1 = 1.3 s, SW 10 (F2) x 150 (F1), TD 4k x 512, echo-antiecho, coupling in F2.

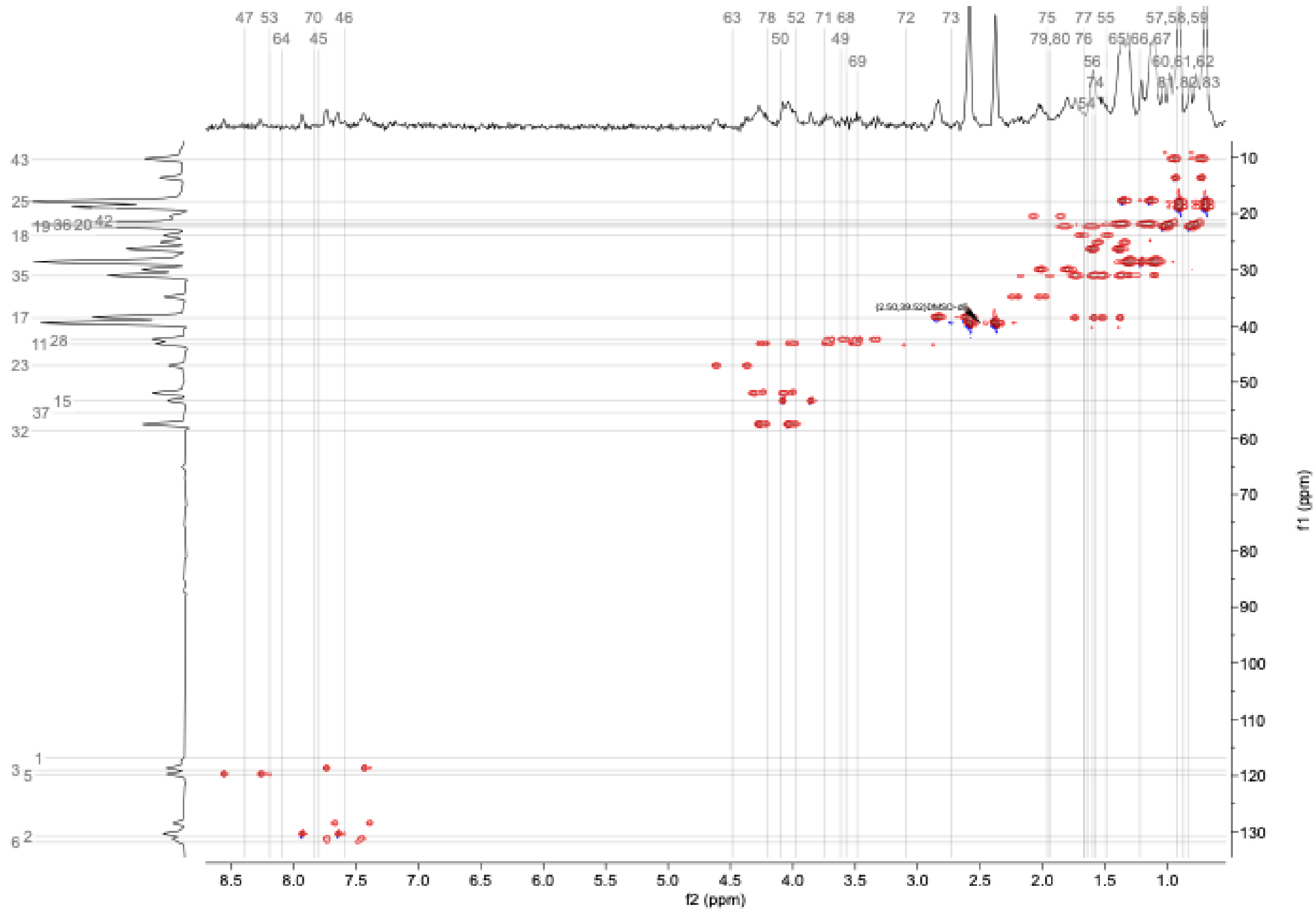

Figure S105. CLIP-HSQC spectrum of cyclic peptide **CP5** at 300 K in DMSO-*d6*/OPA at 600 MHz. NS=56, d1 = 1.3 s, SW 10 (F2) x 150 (F1), TD 4k x 512, echo-antiecho, coupling in F2.

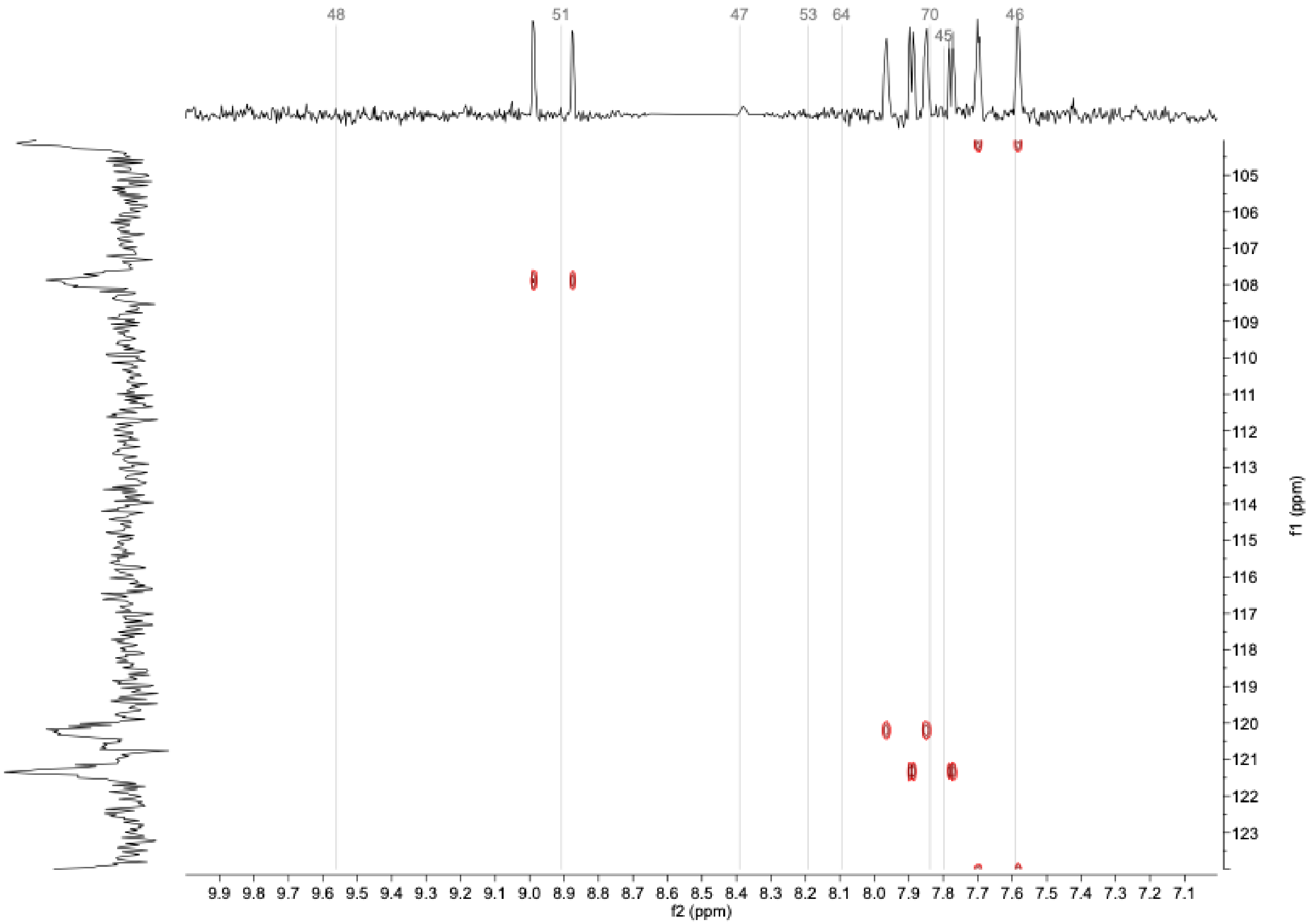

Figure S106. ^{15}N-CLIP-HSQC spectrum of cyclic peptide **CP5** at 300 K in DMSO-*d6* at 600 MHz. NS=240, d1 = 1.3 s, SW 4 (F2) x 35 (F1), TD 2k x 128, echo-antiecho, coupling in F2. WaterGATE.

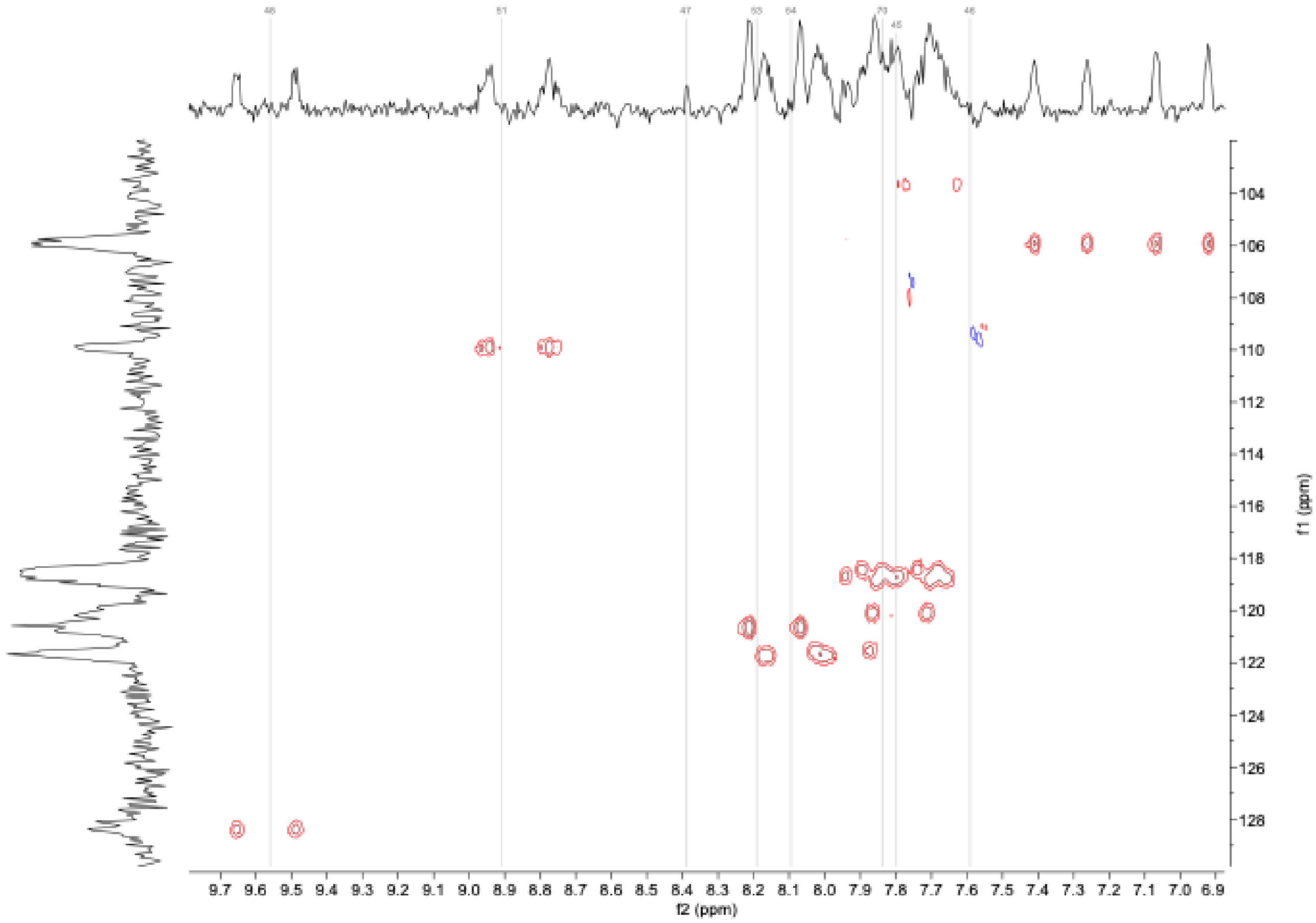

Figure S107. ^{15}N-CLIP-HSQC spectrum of cyclic peptide **CP5** at 300 K in DMSO-*d6*/OPA at 600 MHz. NS=320, d1 = 1.3 s, SW 4 (F2) x 35 (F1), TD 2k x 128, echo-antiecho, coupling in F2. WaterGATE.

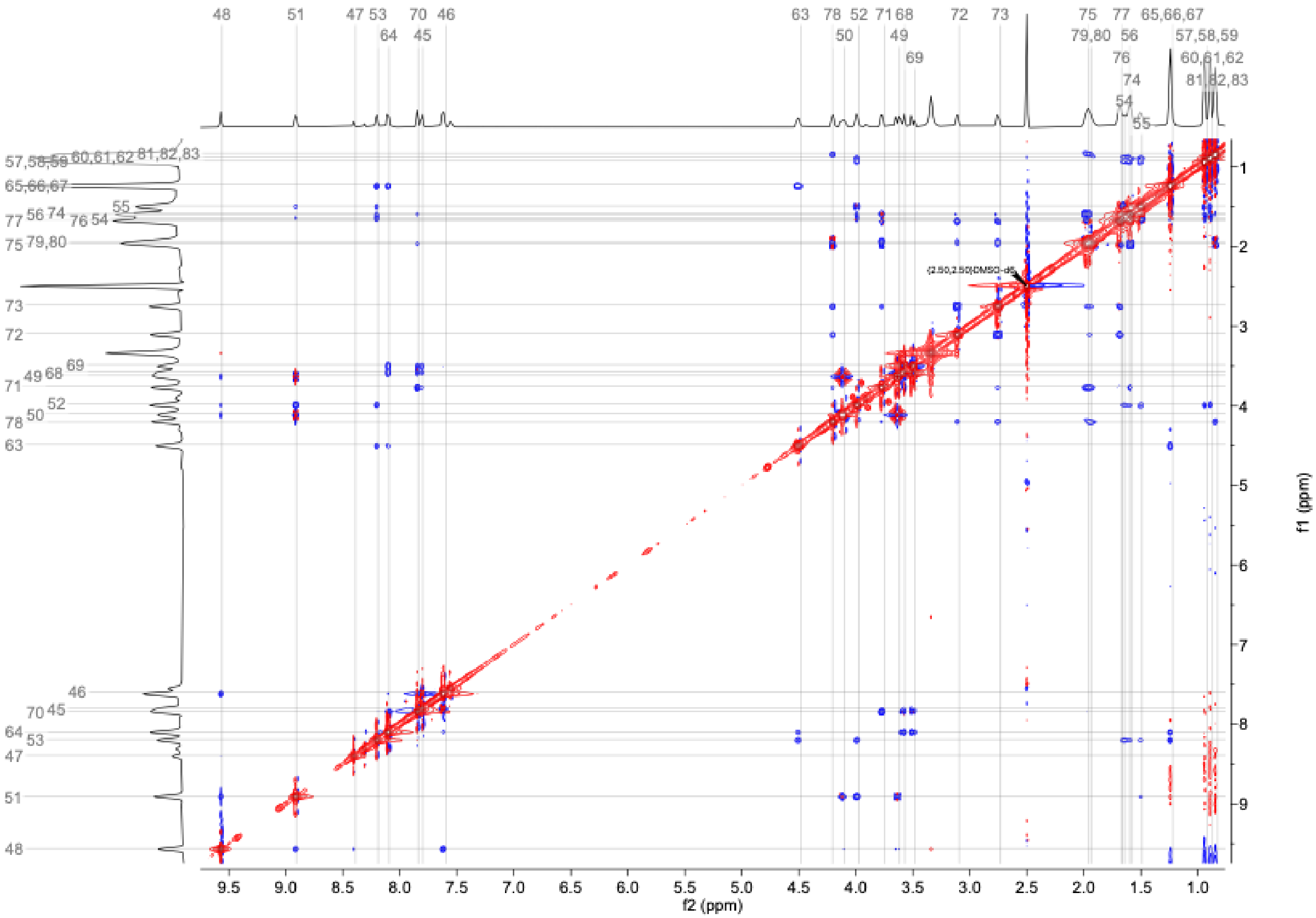

Figure S108. ROESY spectrum of cyclic peptide **CP5** at 300 K in DMSO-*d6* at 600 MHz. NS=24, d1 = 1.3 s, SW 10 (F2) x 10 (F1), TD 2k x 512, States-TPPI, mixing time = 80 ms.

SI.16.6 Spectra for CP6

Unfortunately, even after long acquisition time for a ^{13}C spectra (NS=1024 (TD0=30)), SW 212 ppm, AQ 2.0 s, d1 = 2.5 s, TD = 128 k) no clear spectra could be obtained for this cyclic peptide. Therefore, the numbered structure has been added to the HSQC spectrum. The EASY-ROESY spectrum contains peaks, looking like EXSY peaks, however, because they can also be observed in the CLIP-COSY and TOCSY spectrum, we suspect it to be an artifact and does not reflect chemical exchange.

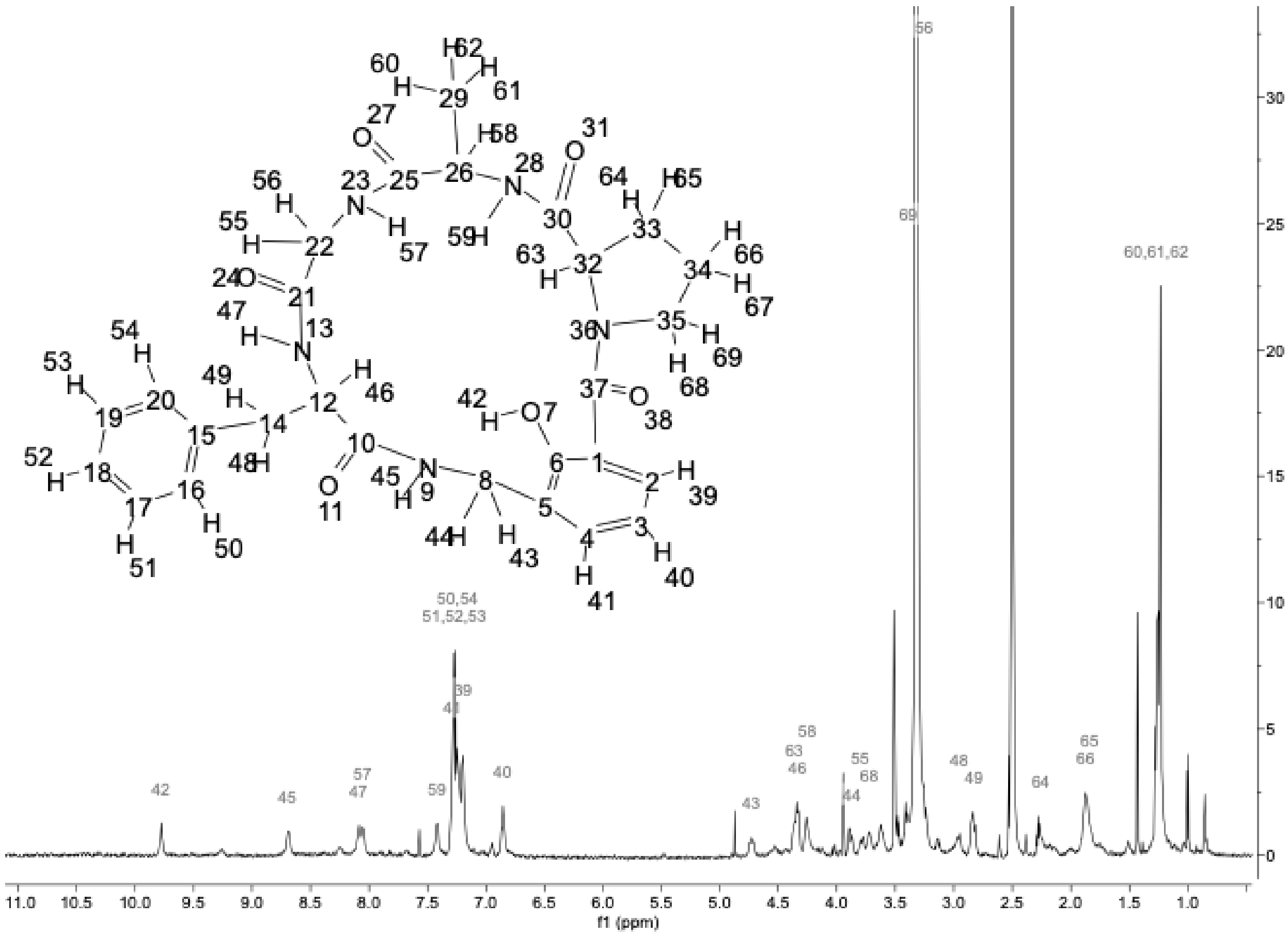

Figure S109. Proton spectrum of cyclic peptide **CP6** at 300 K in DMSO-*d6* at 600 MHz. NS =8, d1= 5 s, AQ = 1.6 s, SW = 16.6 ppm, TD = 32k.

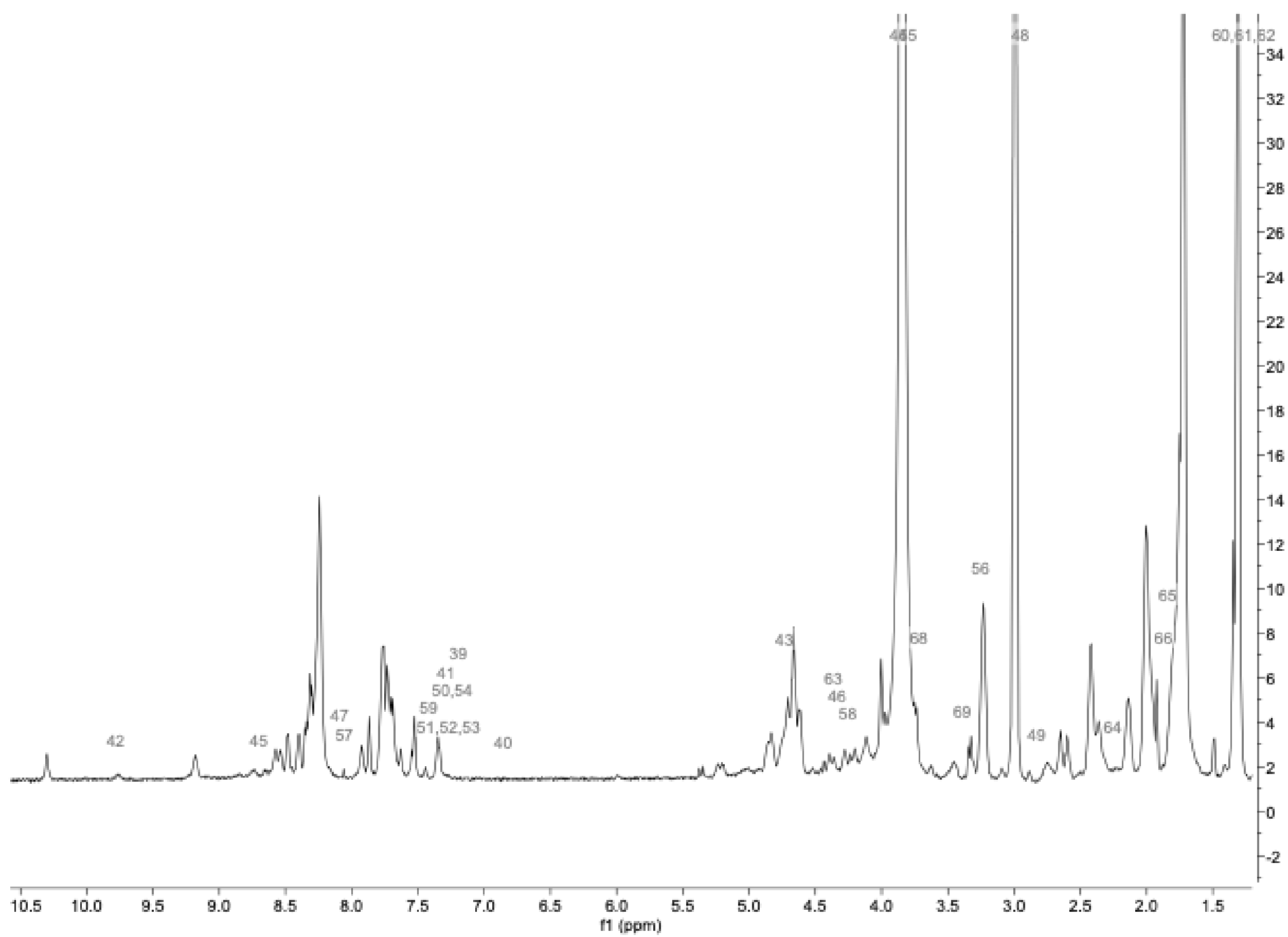

Figure S110. Proton spectrum of cyclic peptide **CP6** at 300 K in anisotropic DMSO-*d6*/OPA at 600 MHz. NS =8, d1= 5 s, AQ = 1.6 s, SW = 16.6 ppm, TD = 32k.

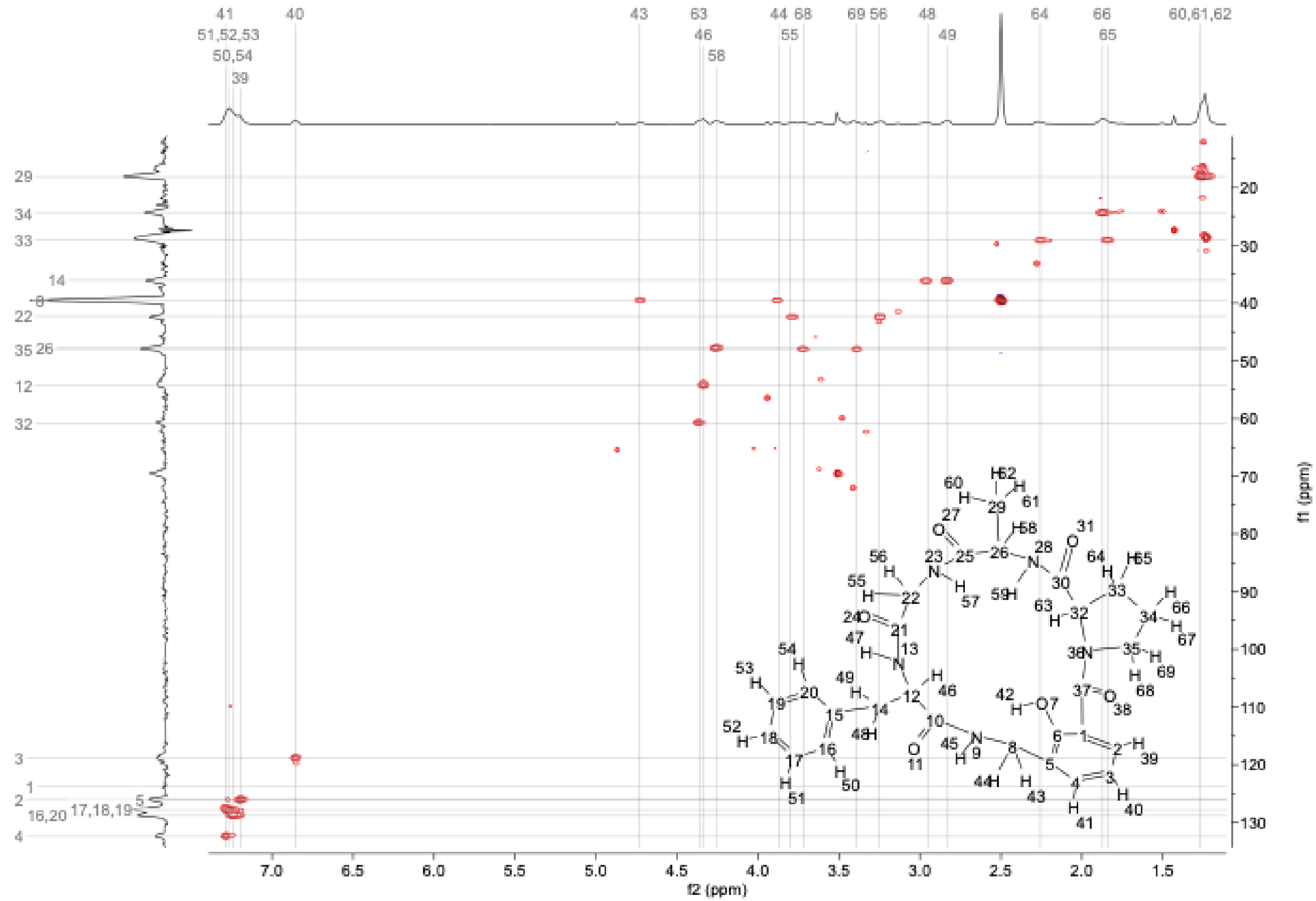

Figure S111. HSQC spectrum of cyclic peptide **CP6** at 300 K in DMSO-*d6* at 600 MHz. NS=32, d1 = 1.3 s, SW 14 (F2) x 160 (F1), TD 2k x 512, echo-antiecho.

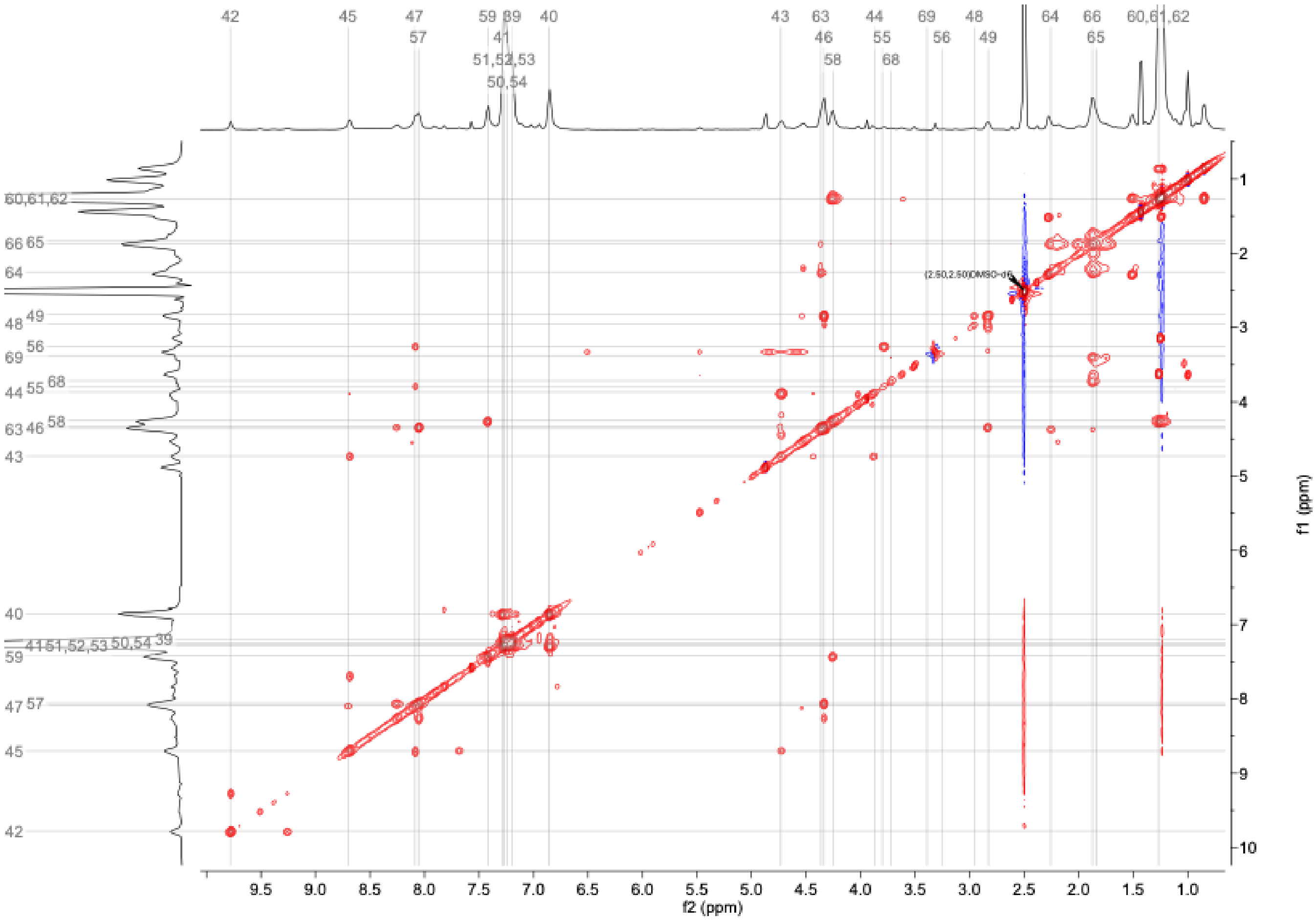

Figure S112. CLIP-COSY spectrum of cyclic peptide **CP6** at 300 K in DMSO-*d6* at 600 MHz. NS=16, d1= 1.3s, SW 16 (F2) x 16 (F1), TD 2k x 512, States-TPPI.

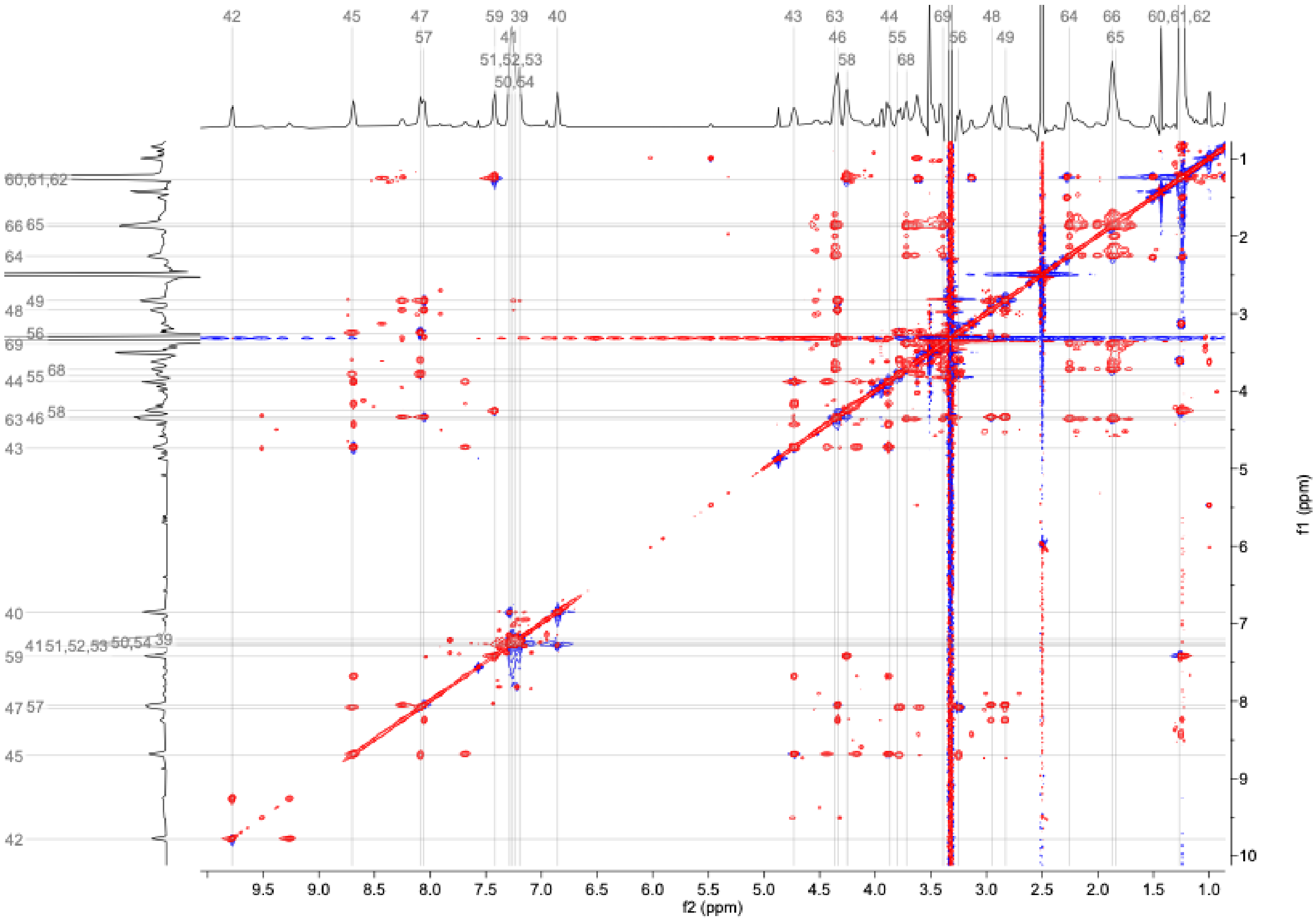

Figure S113. TOCSY spectrum of cyclic peptide **CP6** at 300 K in DMSO-*d6* at 600 MHz. NS=16, d1= 1.3s, SW 14 (F2) x 14 (F1), TD 2k x 1k, States-TPPI, 80 ms mixing time.

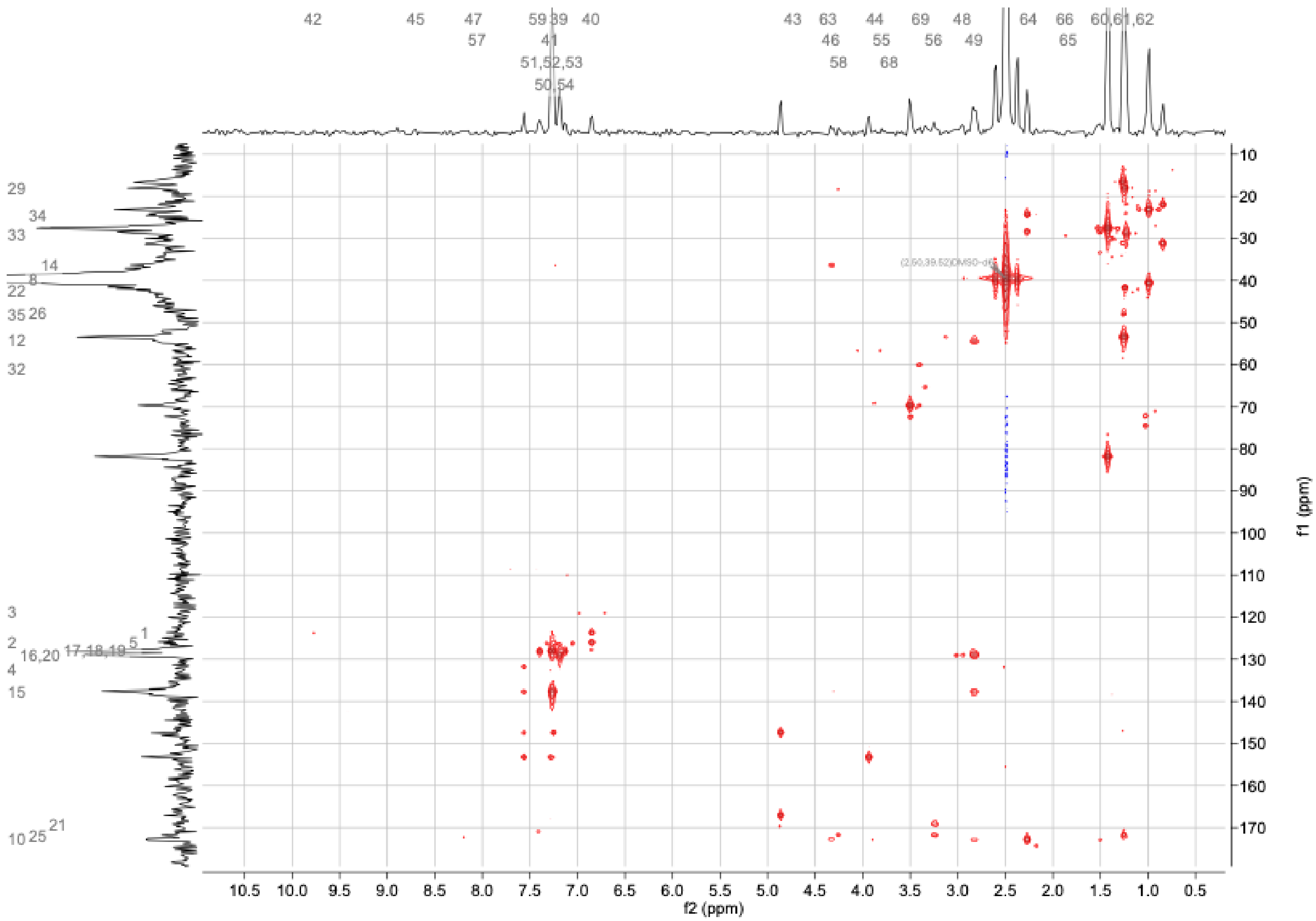

Figure S114. HMBC spectrum of cyclic peptide **CP6** at 300 K in DMSO-*d6* at 600 MHz. NS=32, d1= 1.3s, SW 16.6 (F2) x 207 (F1), TD 2k x 1k, delay for long range coupling 70 ms, echo-antiecho.

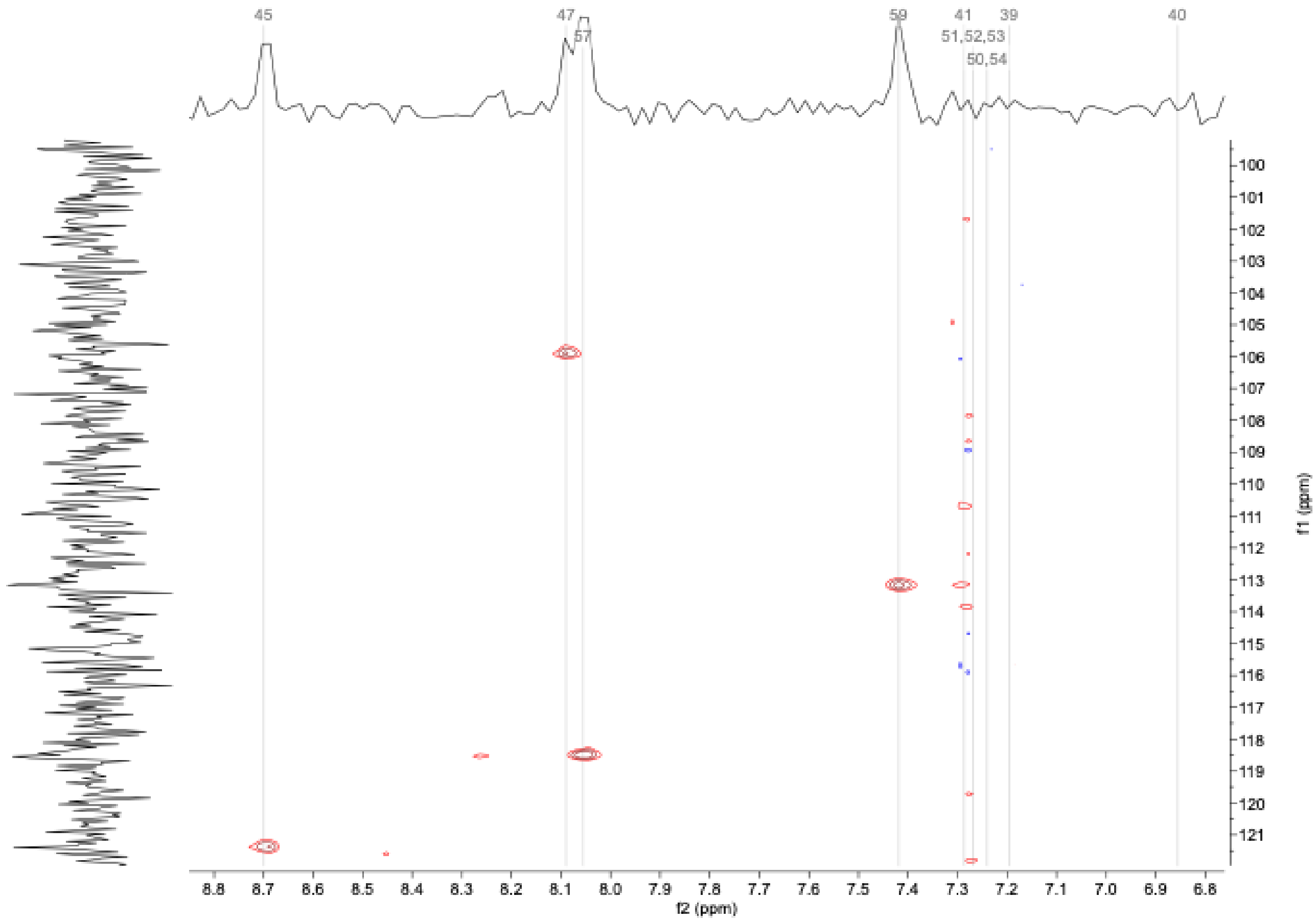

Figure S115. ^{15}N-HSQC spectrum of cyclic peptide **CP6** at 300 K in DMSO-*d6* at 600 MHz. NS=32, d1= 1.3s, SW 16 (F2) x 49.5 (F1), TD 2k x 512, States-TPPI, WaterGATE.

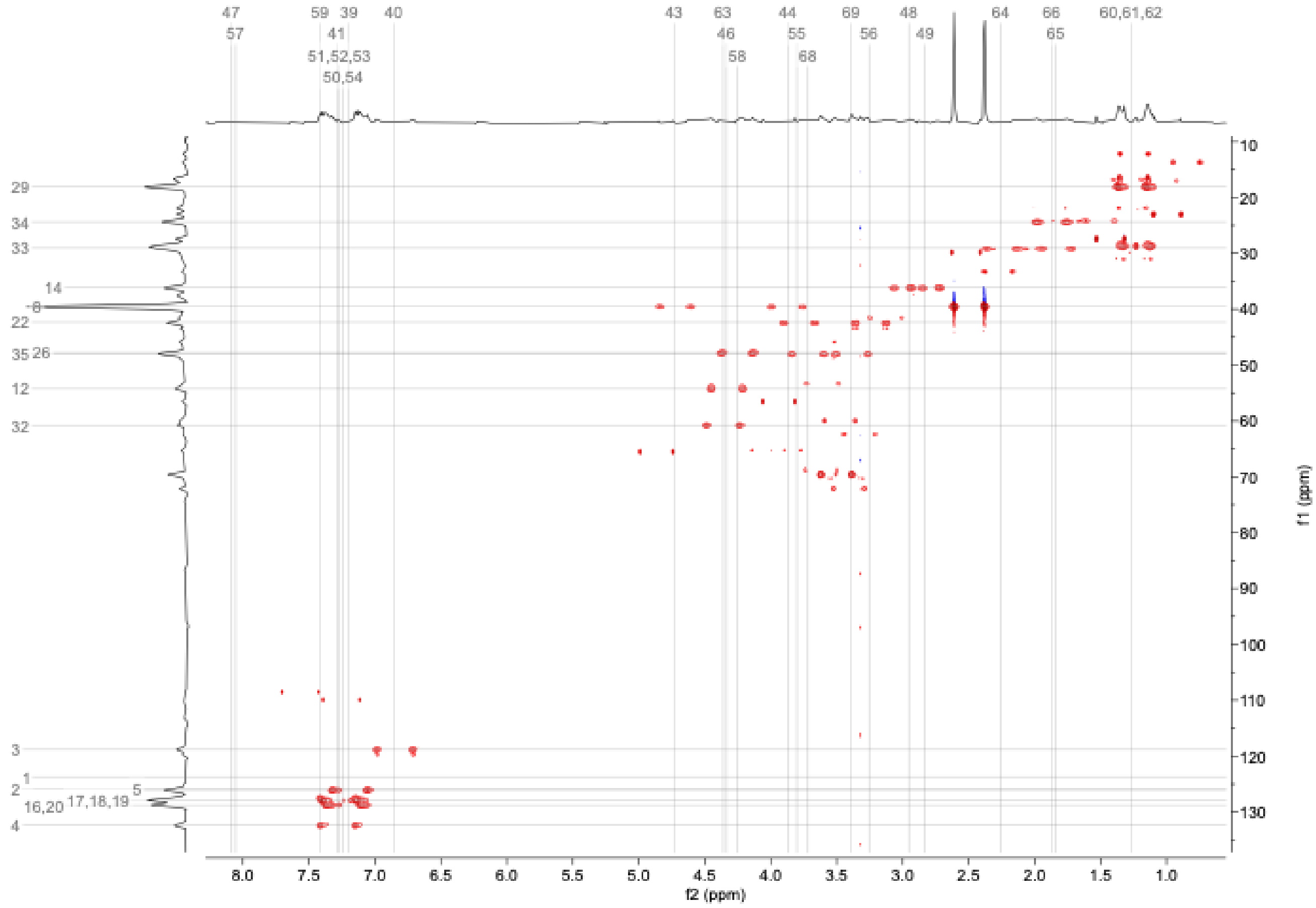

Figure S116. CLIP-HSQC spectrum of cyclic peptide **CP6** at 300 K in DMSO-*d6* at 600 MHz. NS=48, d1 = 1.3 s, SW 11 (F2) x 150 (F1), TD 4k x 512, echo-antiecho, coupling in F2.

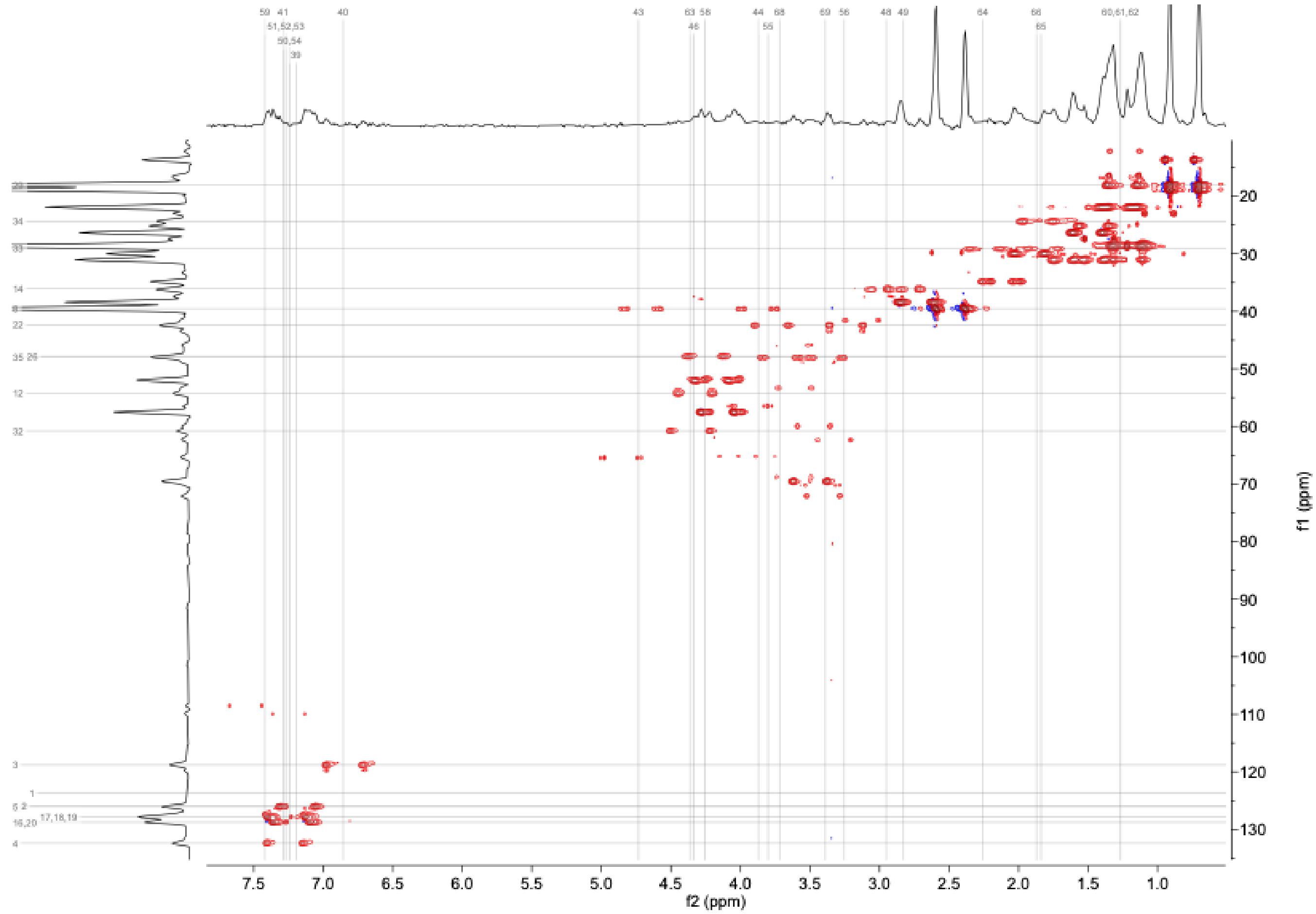

Figure S117. CLIP-HSQC spectrum of cyclic peptide **CP6** at 300 K in DMSO-*d6*/OPA at 600 MHz. NS=88, d1 = 1.3 s, SW 11 (F2) x 150 (F1), TD 4k x 512, echo-antiecho, coupling in F2.

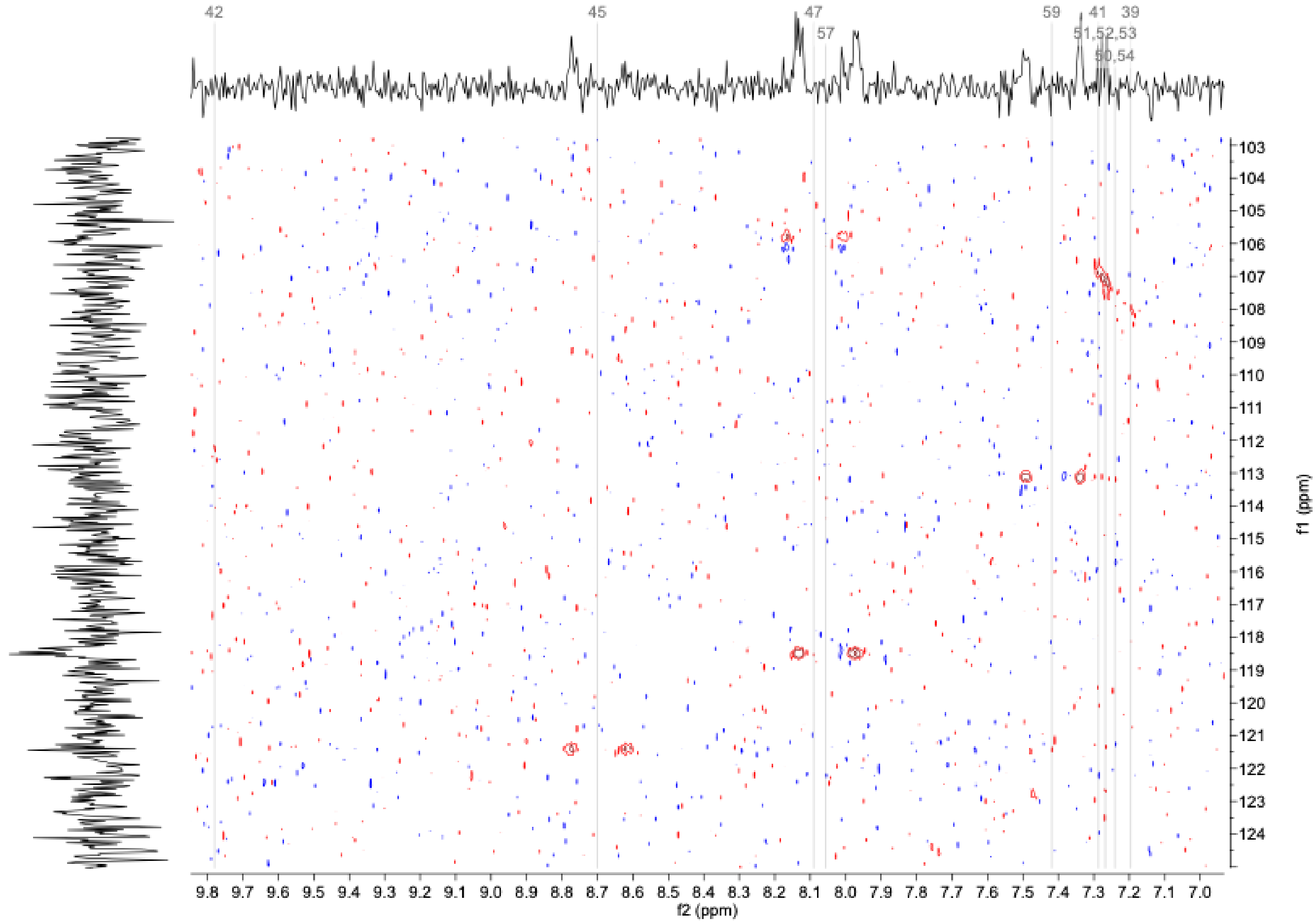

Figure S118. ^{15}N-CLIP-HSQC spectrum of cyclic peptide **CP6** at 300 K in DMSO-*d6* at 600 MHz. NS=138, d1 = 1.3 s, SW 4 (F2) x 30 (F1), TD 2k x 256, echo-antiecho, coupling in F2. WaterGATE.

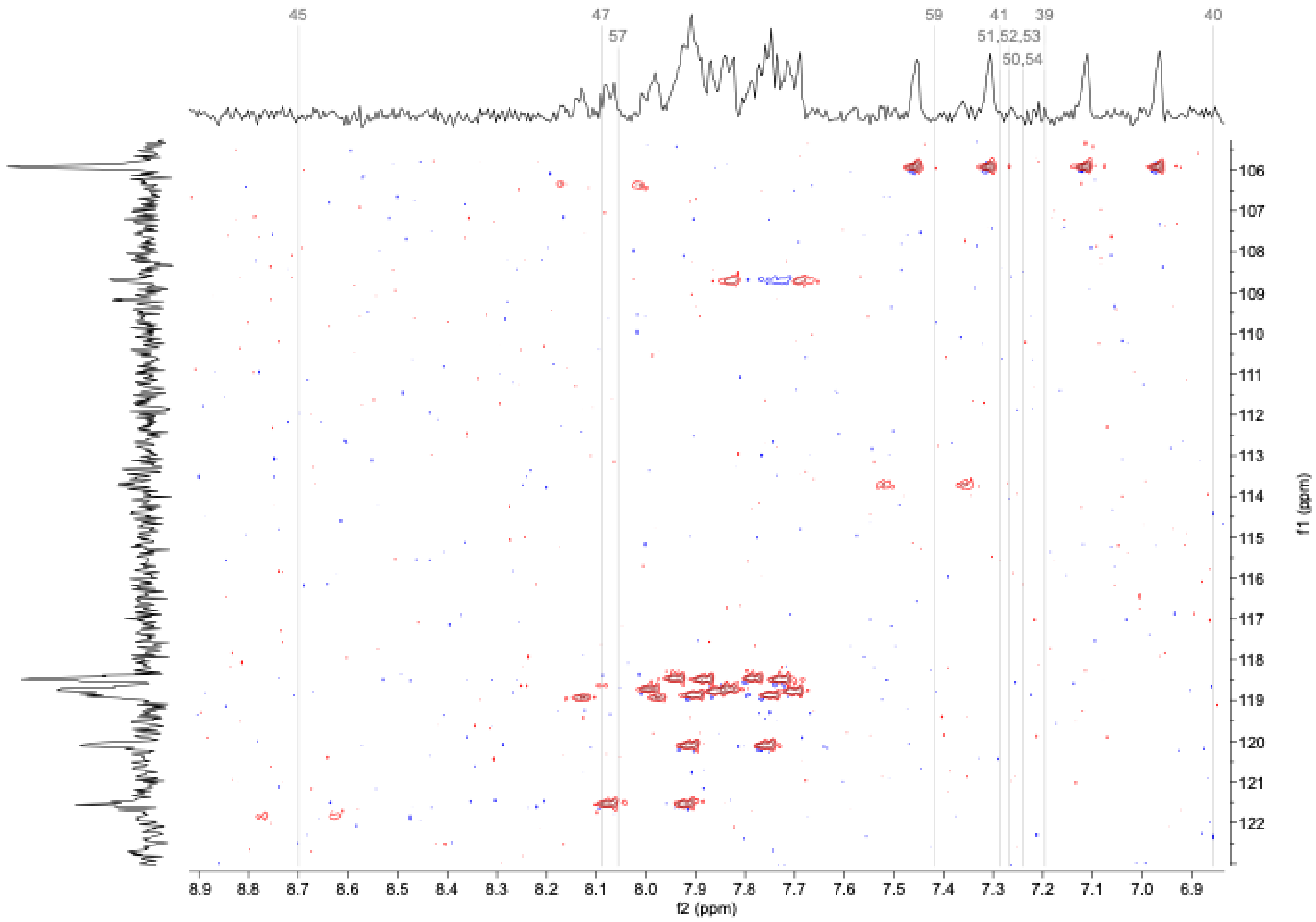

Figure S119. ^{15}N-CLIP-HSQC spectrum of cyclic peptide **CP6** at 300 K in DMSO-*d6*/OPA at 600 MHz. NS=188, d1 = 1.3 s, SW 4 (F2) x 30 (F1), TD 2k x 256, echo-antiecho, coupling in F2. WaterGATE.

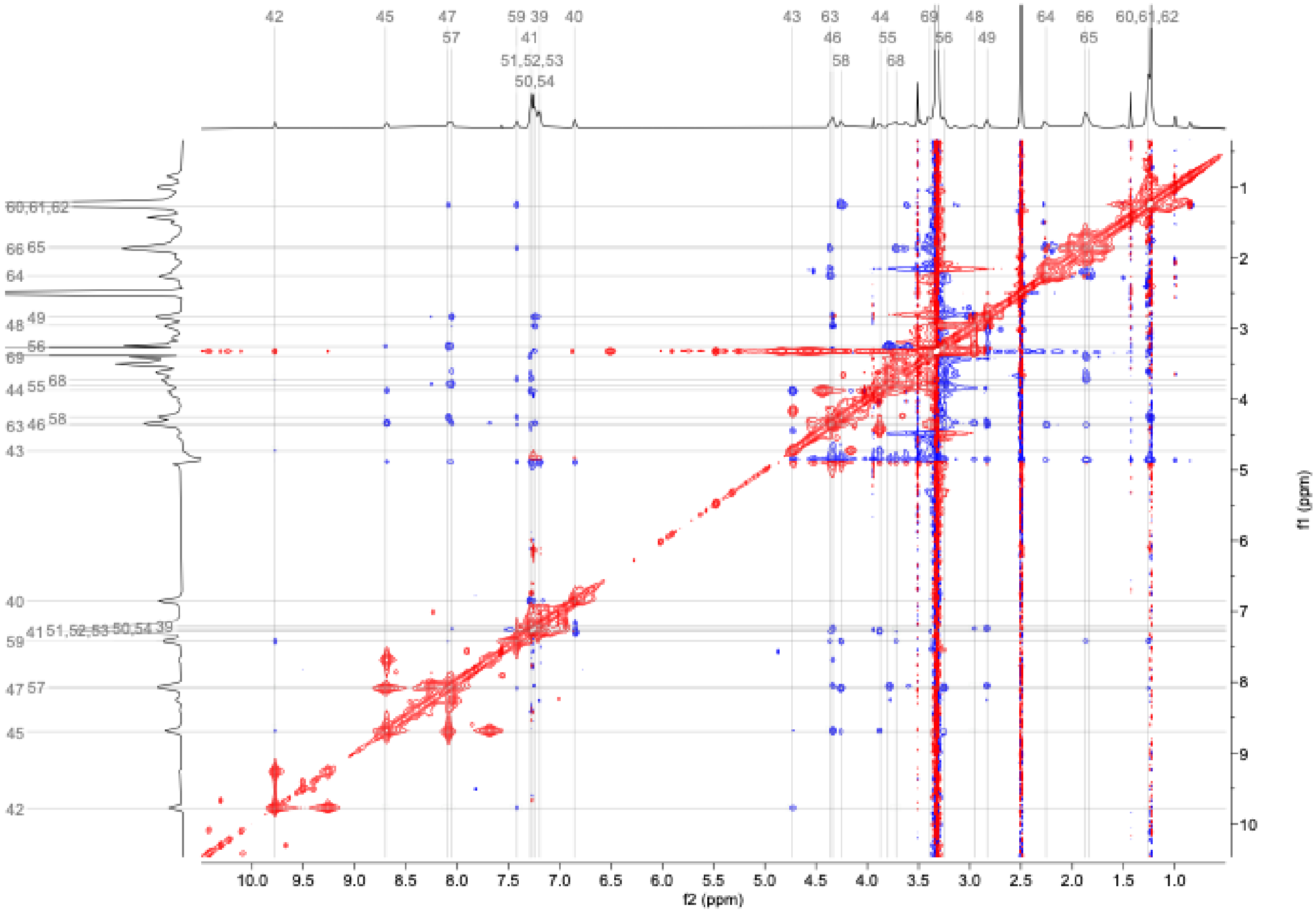

Figure S120. EASY-ROESY spectrum of cyclic peptide **CP6** at 300 K in DMSO-*d6* at 600 MHz. NS=24, d1 = 1.3 s, SW 11.9 (F2) x 11.9 (F1), TD 4k x 512, States-TPPI, mixing time = 80 ms.

SI.16.7 Spectra for CP7

For better visibility, the assignments for the minor are not indicated in the spectra below, only the assignments for the major conformer are shown.

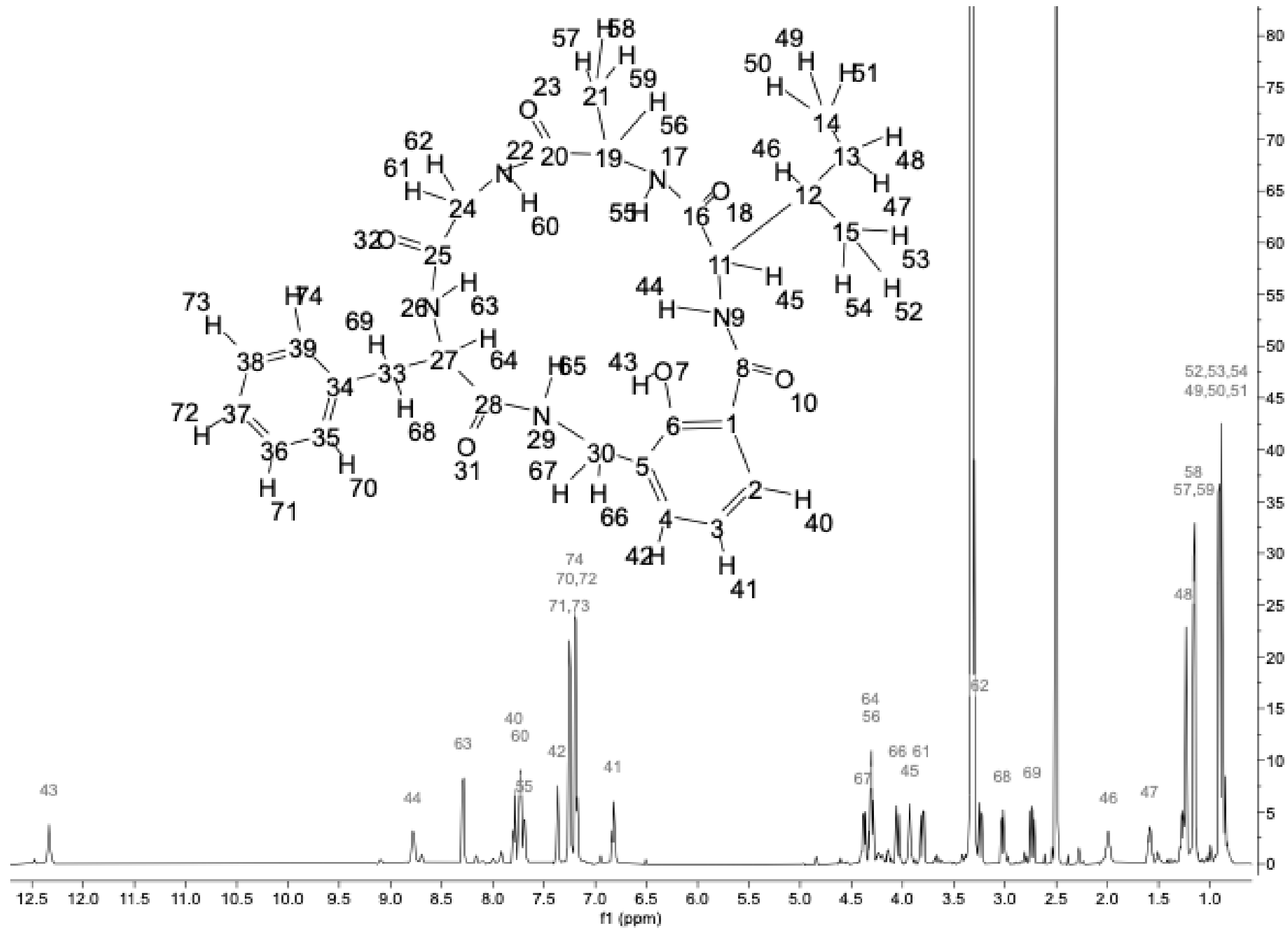

Figure S121. Proton spectrum of cyclic peptide **CP7** at 300 K in DMSO-*d6* at 600 MHz. NS =8, d1= 5 s, AQ = 1.6 s, SW = 16.6 ppm, TD = 32k.

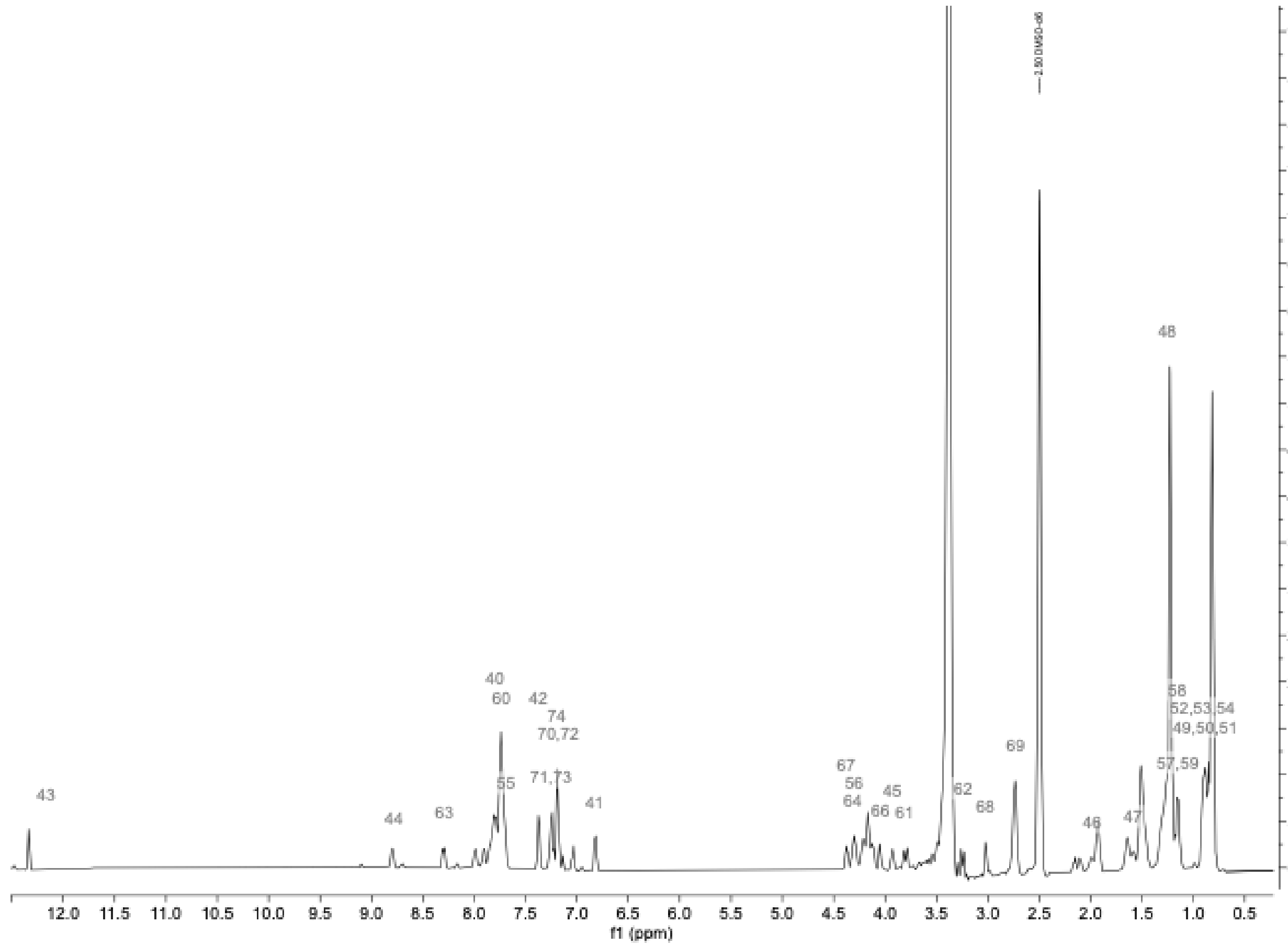

Figure S122. Proton spectrum of cyclic peptide **CP7** at 300 K in anisotropic DMSO-*d6*/OPA at 600 MHz. NS =8, d1= 5 s, AQ = 1.6 s, SW = 16.6 ppm, TD = 32k.

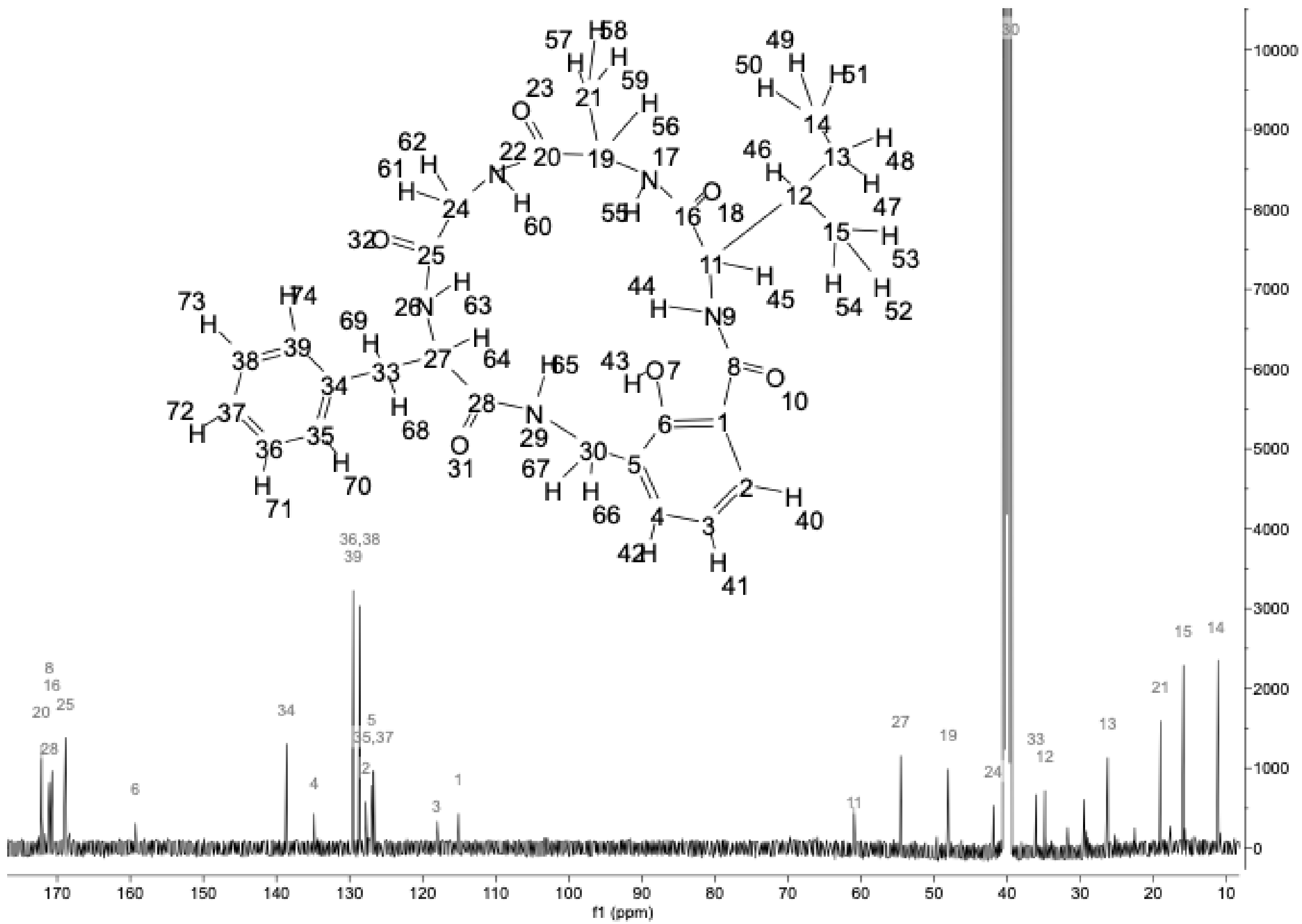

Figure S123. Carbon spectrum of cyclic peptide **CP7** at 300 K in DMSO-*d6* at 600 MHz. NS =1024 (TD0 =27), d1= 2.5 s, AQ = 2.0 s, SW = 212.4 ppm, TD = 128k, 5.0 Hz LB.

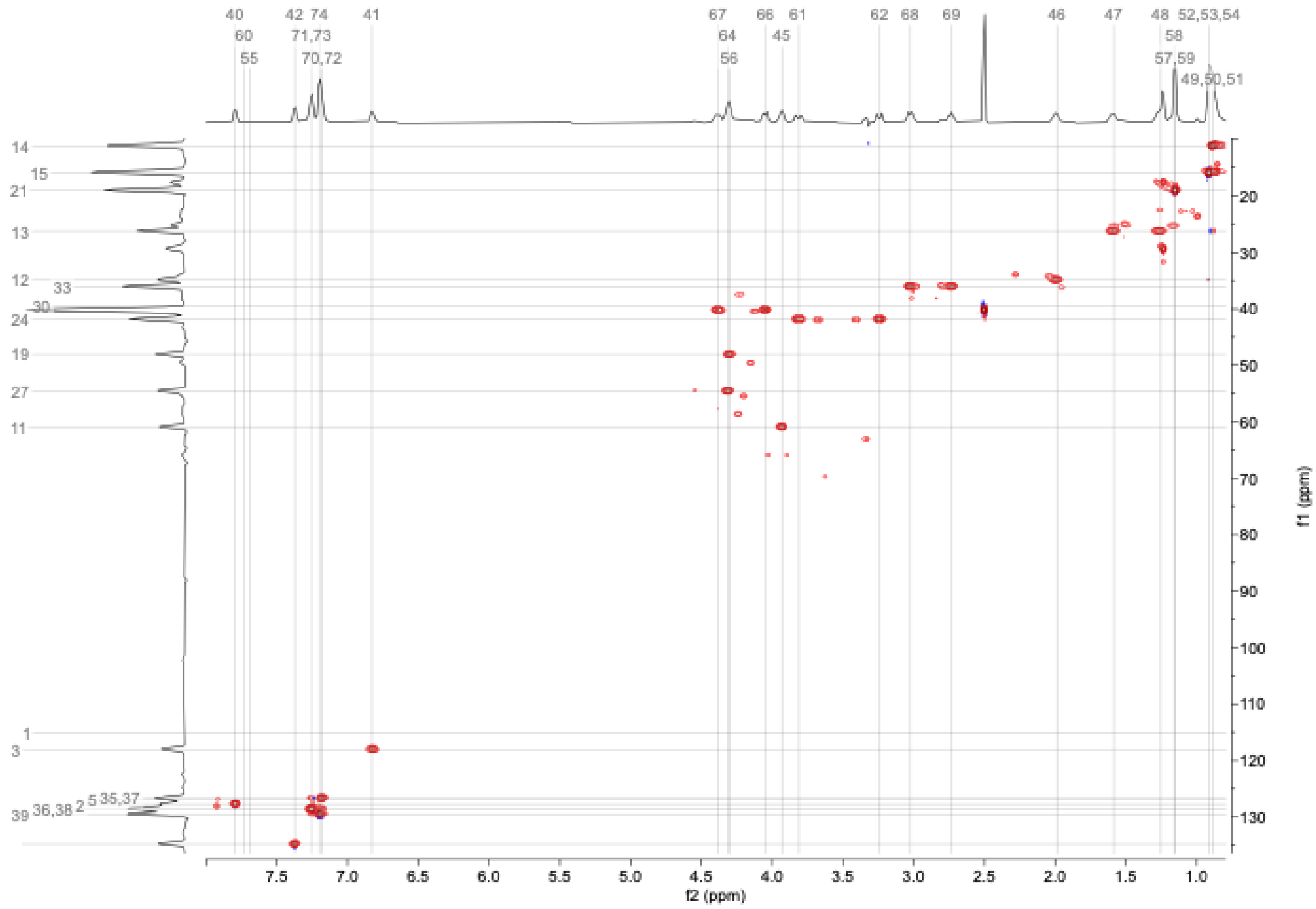

Figure S124. HSQC spectrum of cyclic peptide **CP7** at 300 K in DMSO-*d6* at 600 MHz. NS=32, d1 = 1.3 s, SW 12 (F2) x 160 (F1), TD 2k x 512, echo-antiecho.

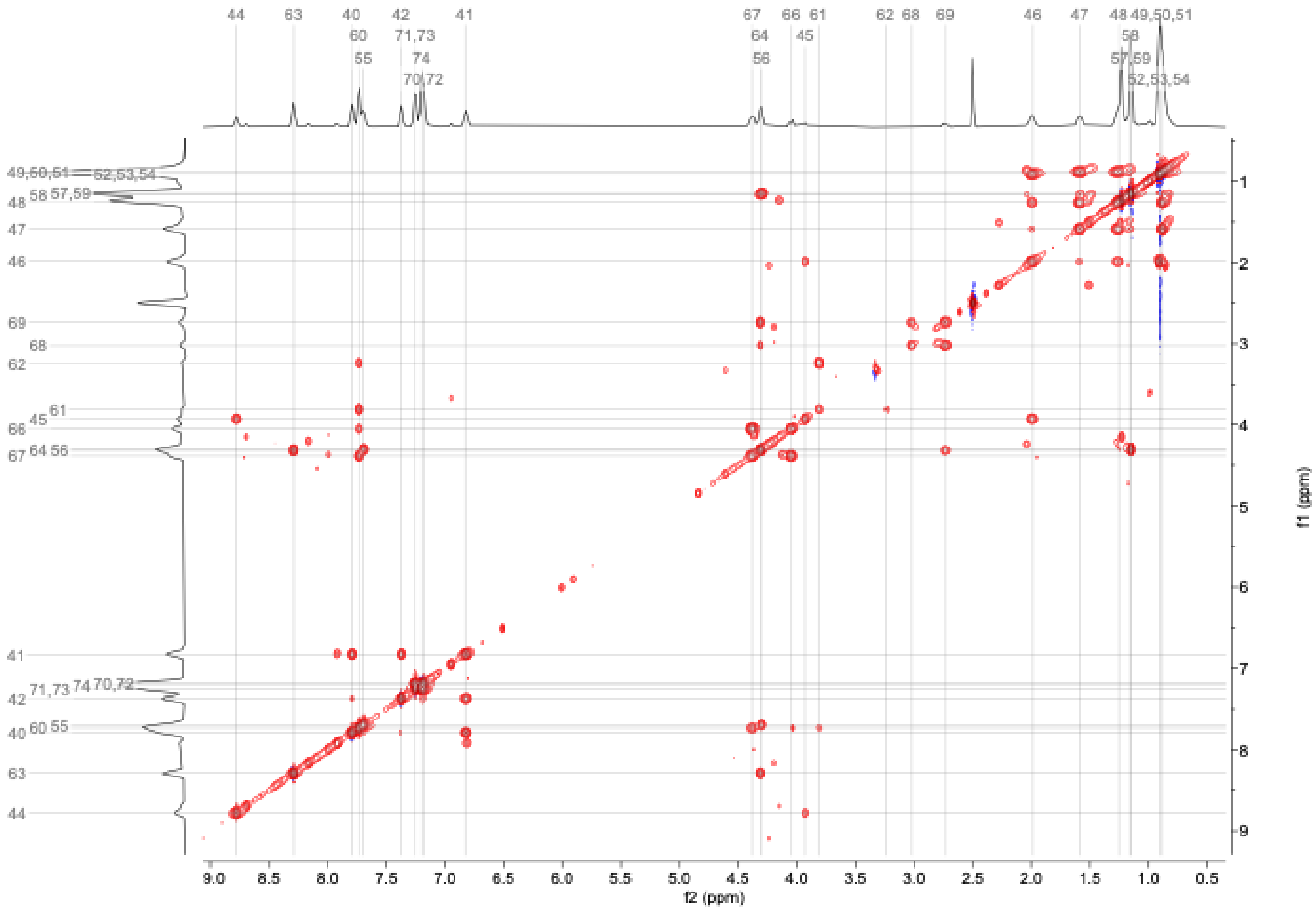

Figure S125. CLIP-COSY spectrum of cyclic peptide **CP7** at 300 K in DMSO-*d6* at 600 MHz. NS=16, d1= 1.3s, SW 16 (F2) x 16 (F1), TD 2k x 512, States-TPPI.

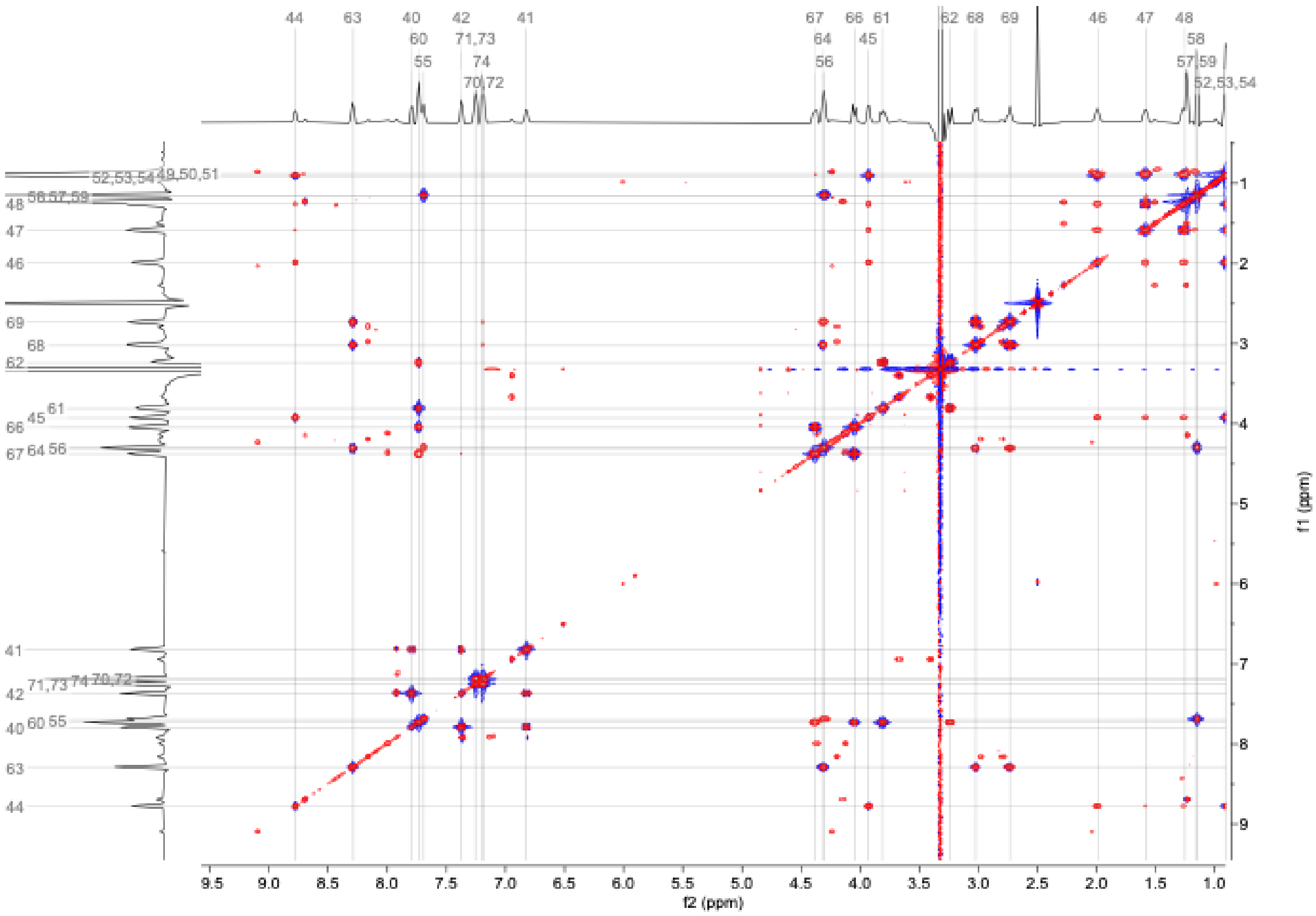

Figure S126. TOCSY spectrum of cyclic peptide **CP7** at 300 K in DMSO-*d6* at 600 MHz. NS=16, d1= 1.3s, SW 14 (F2) x 14 (F1), TD 2k x 1k, States-TPPI, 80 ms mixing time.

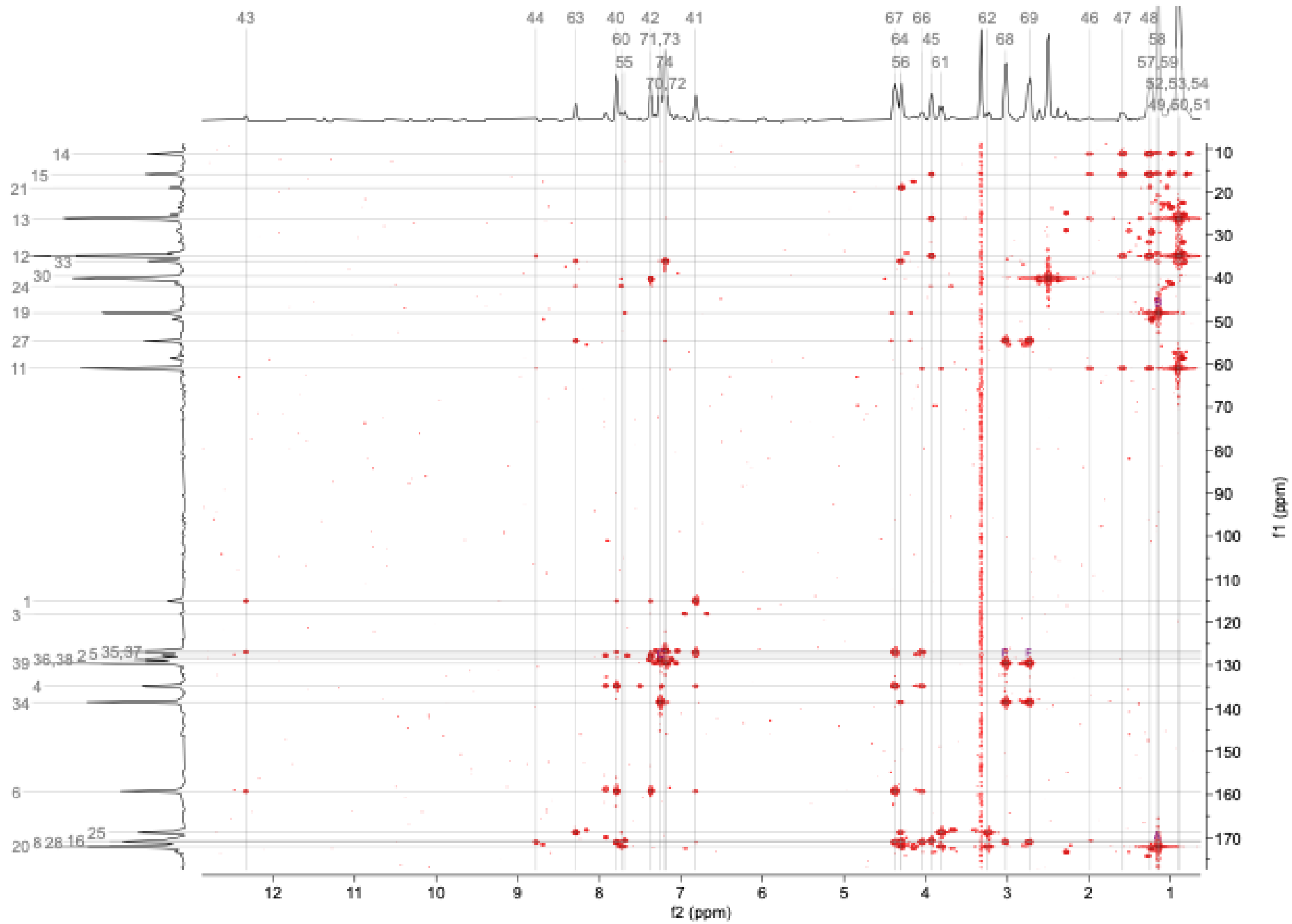

Figure S127. HMBC spectrum of cyclic peptide **CP7** at 300 K in DMSO-*d6* at 600 MHz. NS=32, d1= 1.3s, SW 16.6 (F2) x 207 (F1), TD 2k x 1k, delay for long range coupling 70 ms, echo-antiecho.

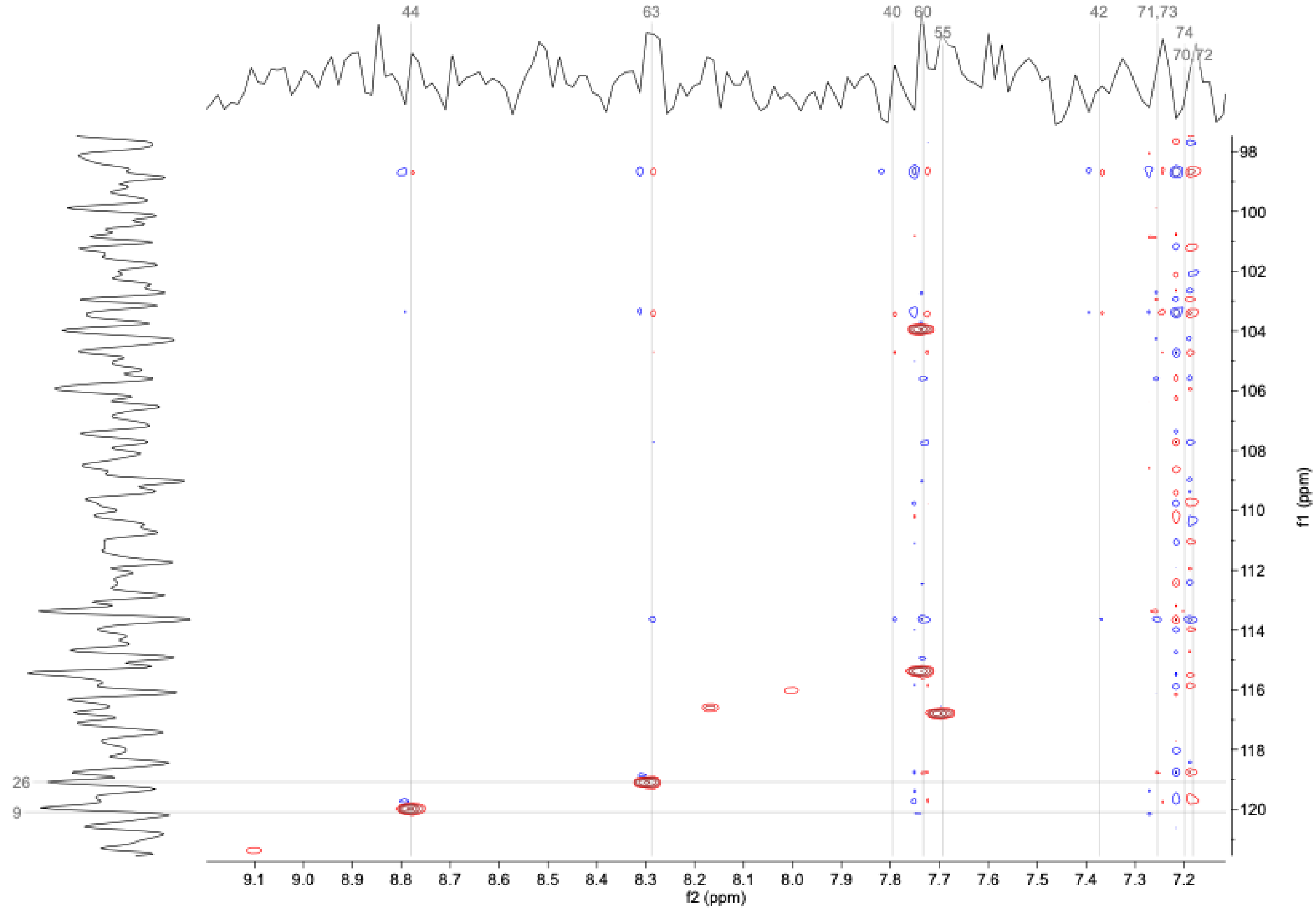

Figure S128. ^{15}N-HSQC spectrum of cyclic peptide **CP7** at 300 K in DMSO-*d6* at 600 MHz. NS=32, d1= 1.3s, SW 14 (F2) x 49.5 (F1), TD 2k x 512, States-TPPI, WaterGATE.

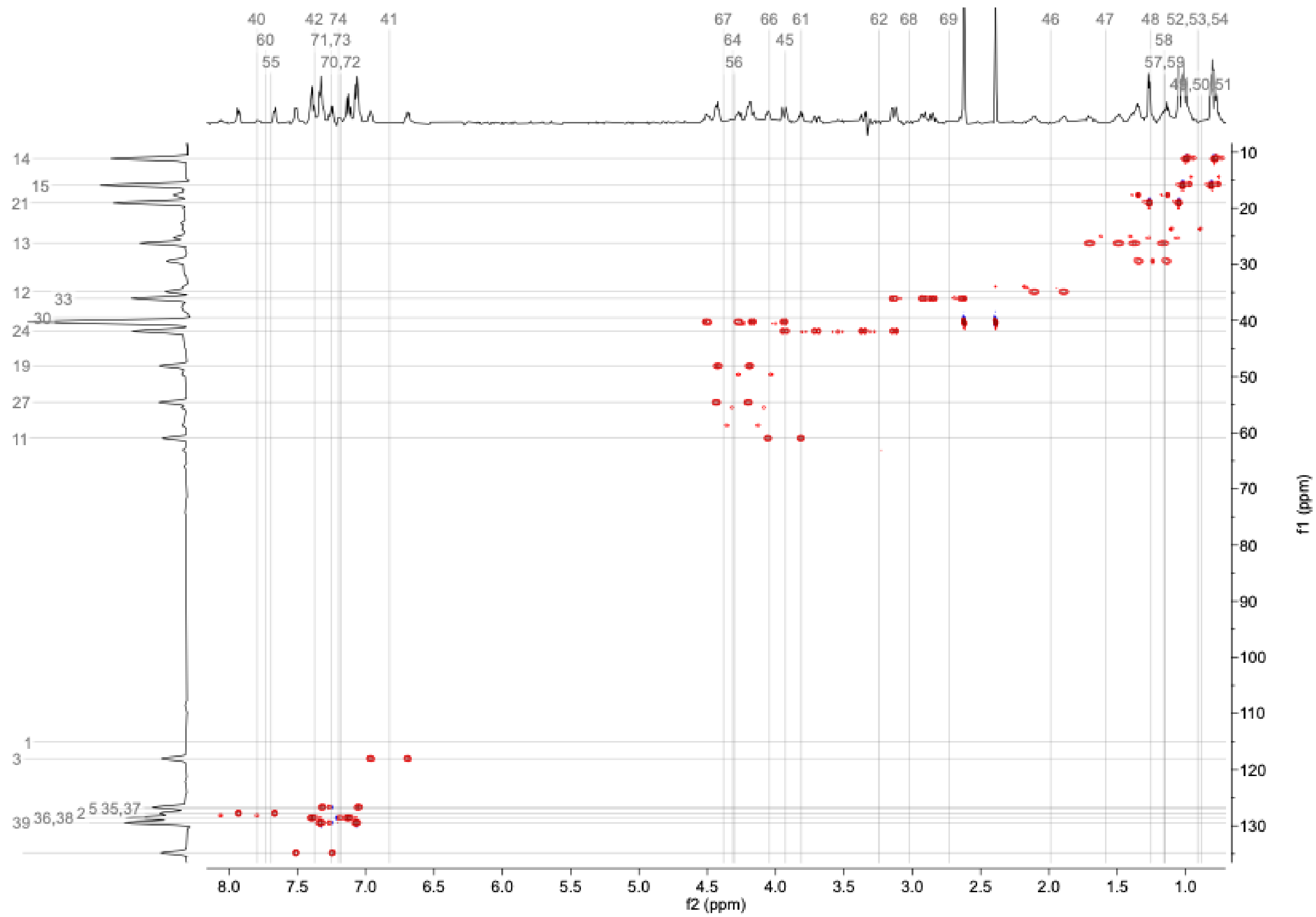

Figure S129. CLIP-HSQC spectrum of cyclic peptide **CP7** at 300 K in DMSO-*d6* at 600 MHz. NS=56, d1 = 1.3 s, SW 11 (F2) x 150 (F1), TD 4k x 512, echo-antiecho, coupling in F2.

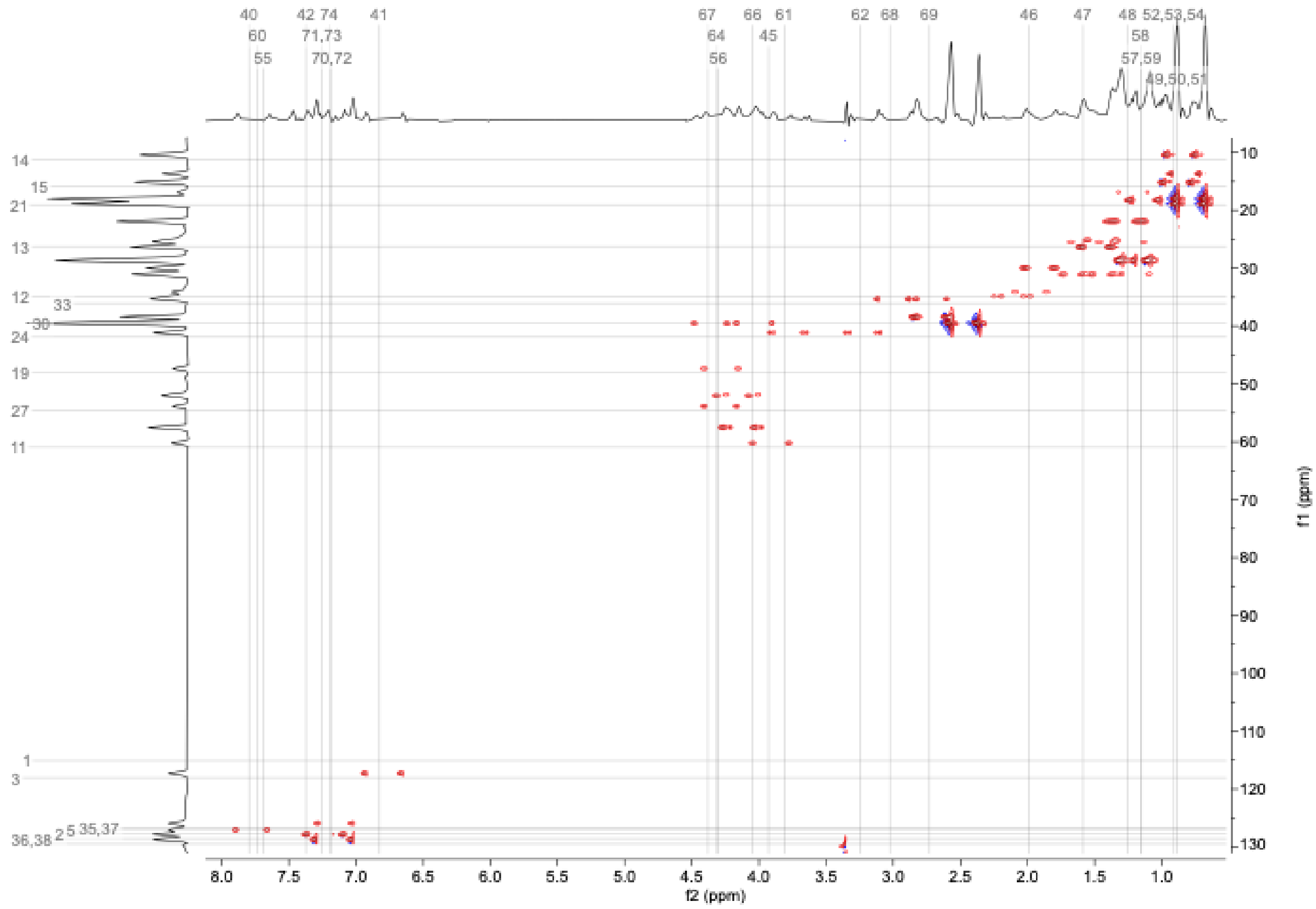

Figure S130. CLIP-HSQC spectrum of cyclic peptide **CP7** at 300 K in DMSO-*d6*/OPA at 600 MHz. NS=96, d1 = 1.3 s, SW 11 (F2) x 130 (F1), TD 4k x 512, echo-antiecho, coupling in F2.

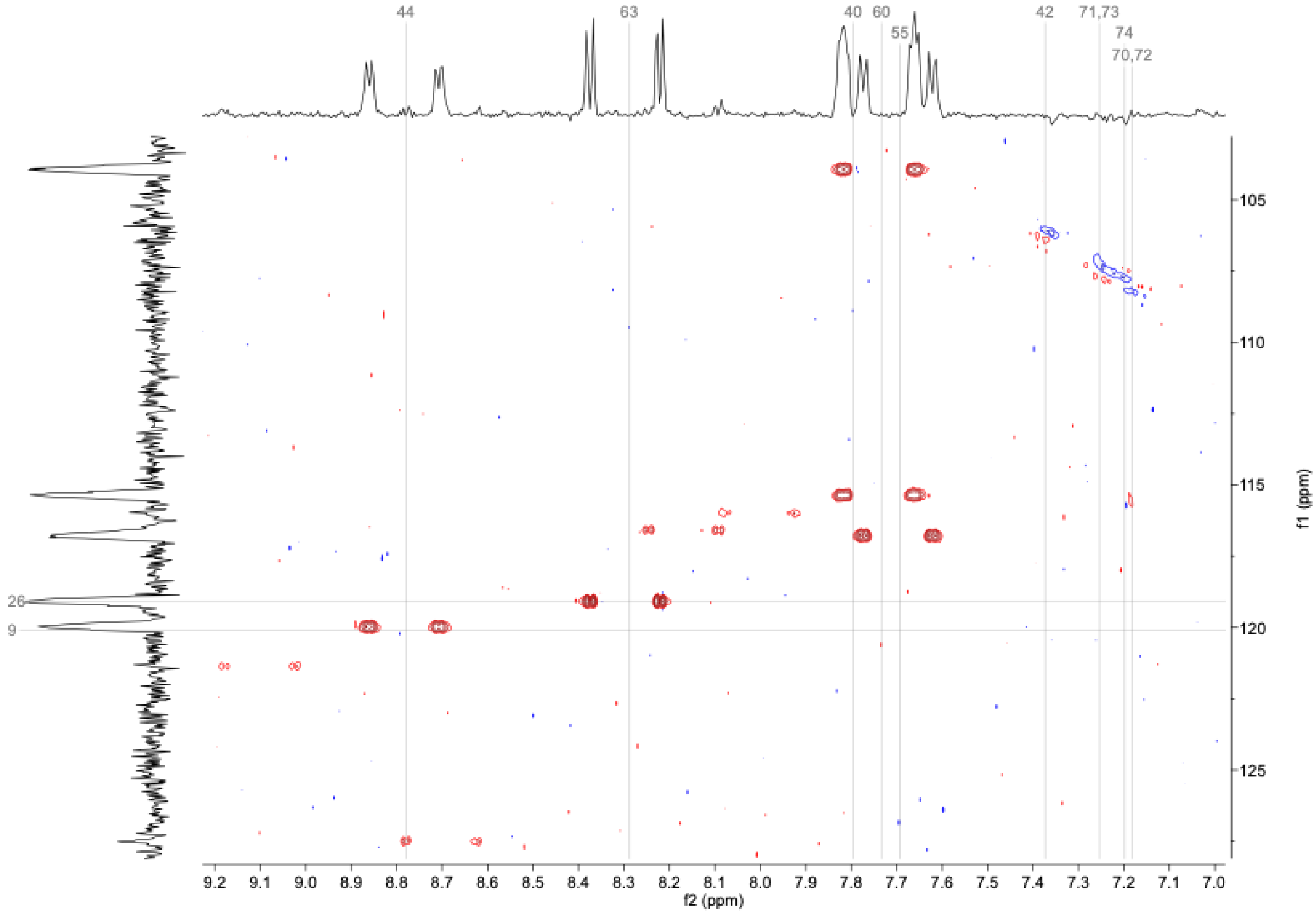

Figure S131. ^{15}N-CLIP-HSQC spectrum of cyclic peptide **CP7** at 300 K in DMSO-*d6* at 600 MHz. NS=146, d1 = 1.3 s, SW 4 (F2) x 30 (F1), TD 2k x 256, echo-antiecho, coupling in F2. WaterGATE.

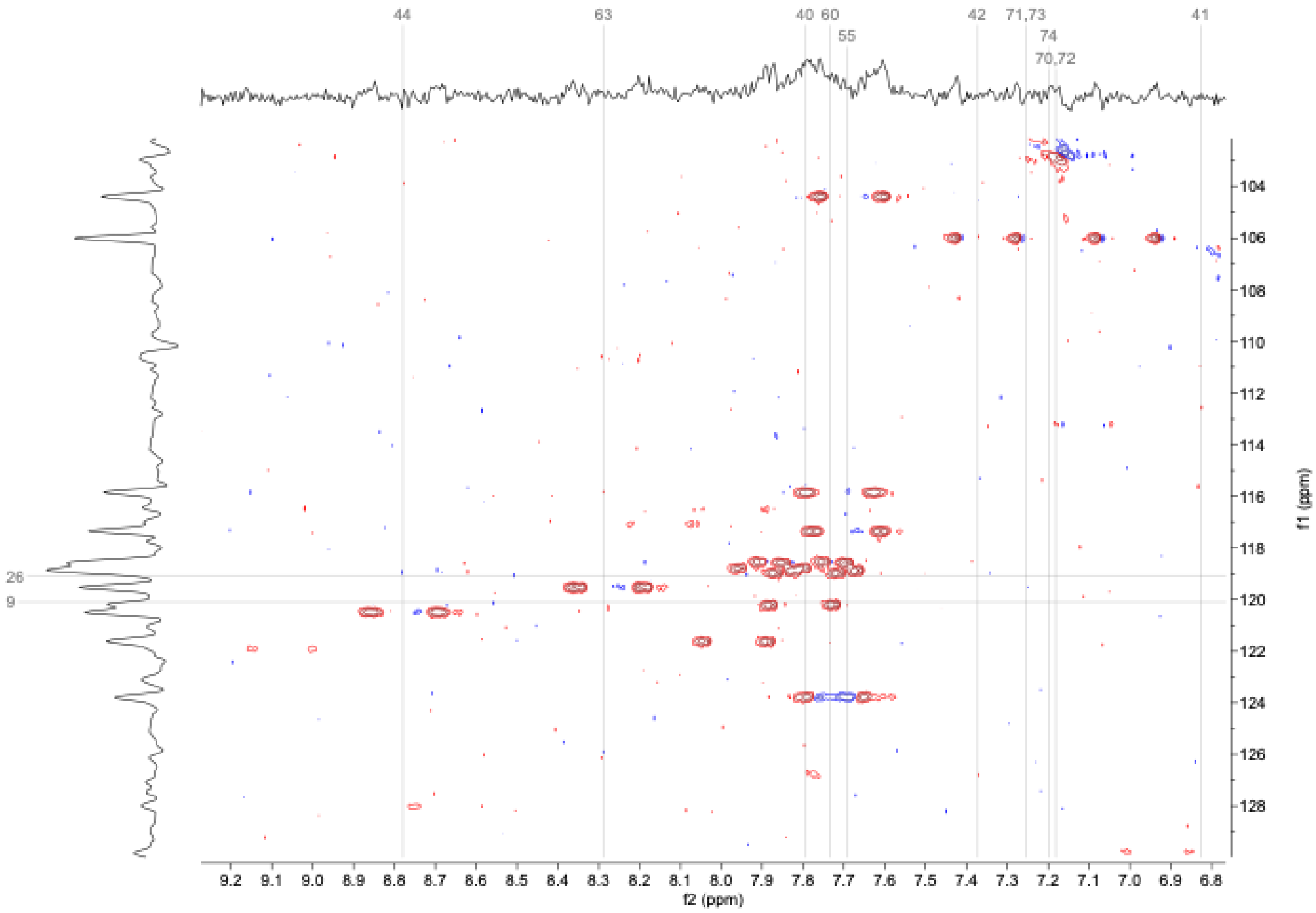

Figure S132. ^{15}N-CLIP-HSQC spectrum of cyclic peptide **CP7** at 300 K in DMSO-*d6*/OPA at 600 MHz. NS=160, d1 = 1.3 s, SW 4 (F2) x 30 (F1), TD 2k x 256, echo-antiecho, coupling in F2. WaterGATE.

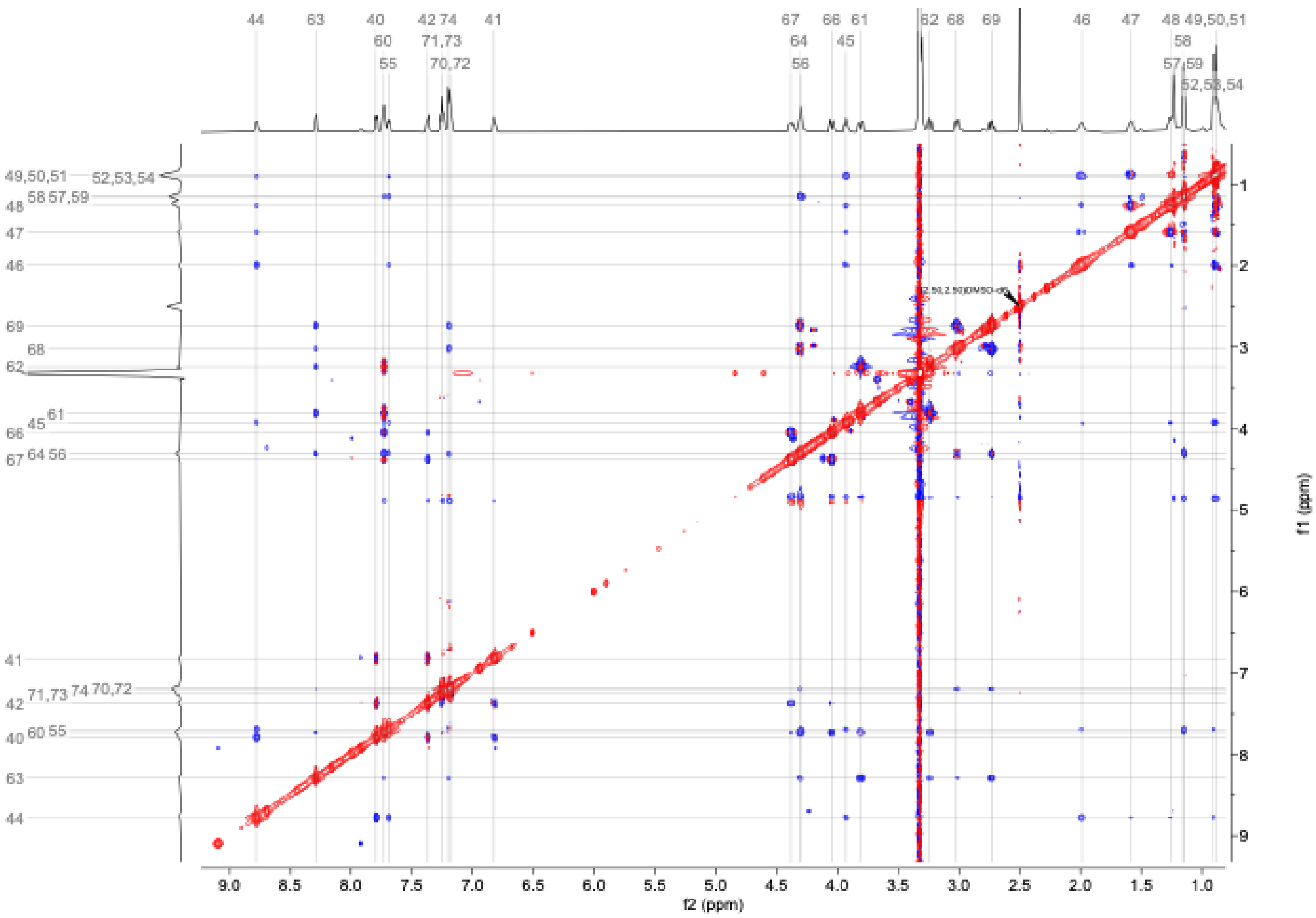

Figure S133. EASY-ROESY spectrum of cyclic peptide **CP7** at 300 K in DMSO-*d6* at 600 MHz. NS=24, d1 = 1.3 s, SW 11.9 (F2) x 11.9 (F1), TD 4k x 512, States-TPPI, mixing time = 80 ms.

SI.16.8 Spectra for CP8

The EASY-ROESY spectrum contains peaks, looking like EXSY peaks, however, because they can also be observed in the CLIP-COSY and TOCSY spectrum, we suspect it to be an artifact and does not reflect chemical exchange.

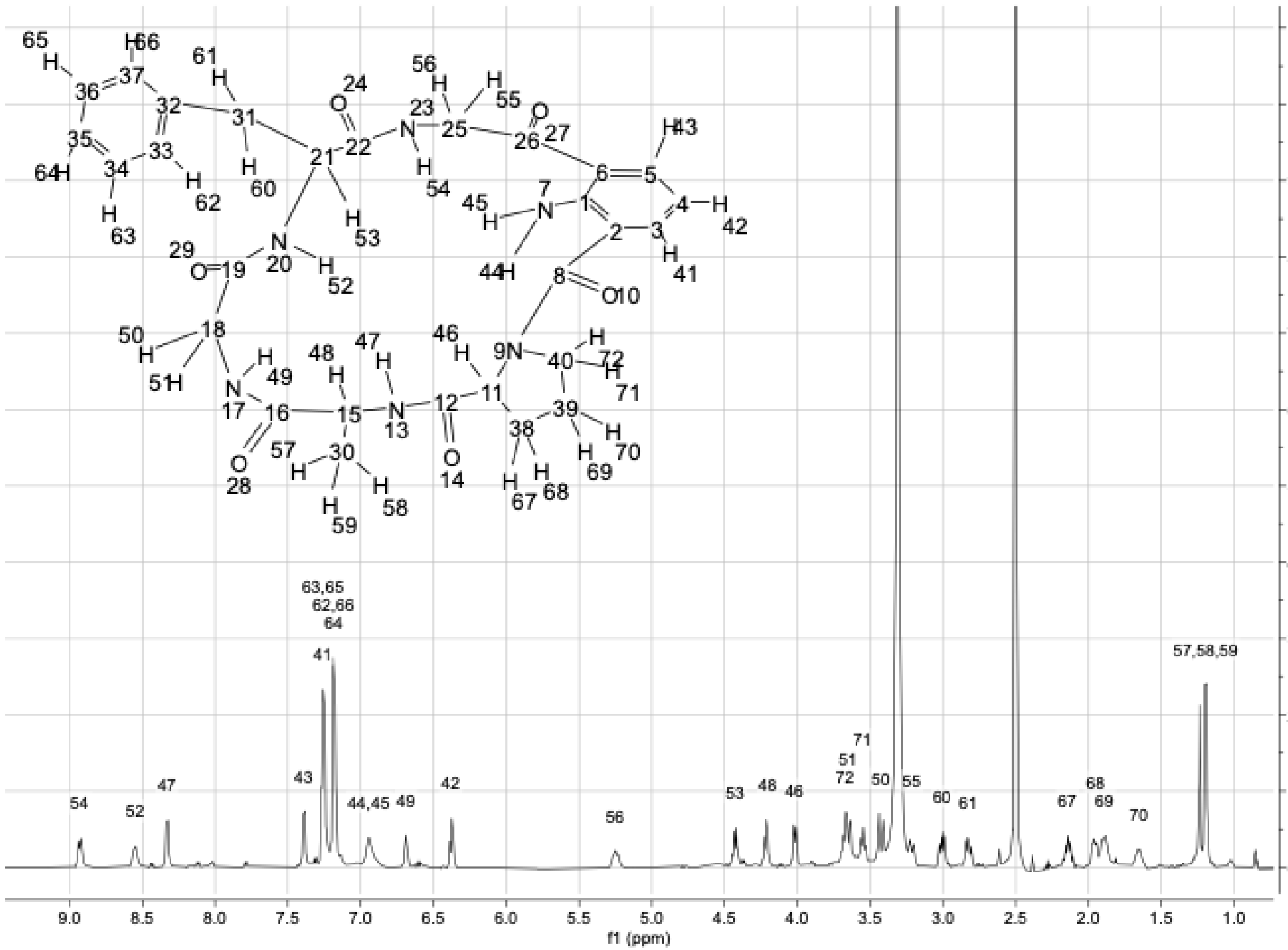

Figure S134. Proton spectrum of cyclic peptide **CP8** at 300 K in DMSO-*d6* at 600 MHz. NS =8, d1= 5 s, AQ = 1.6 s, SW = 16.6 ppm, TD = 32k.

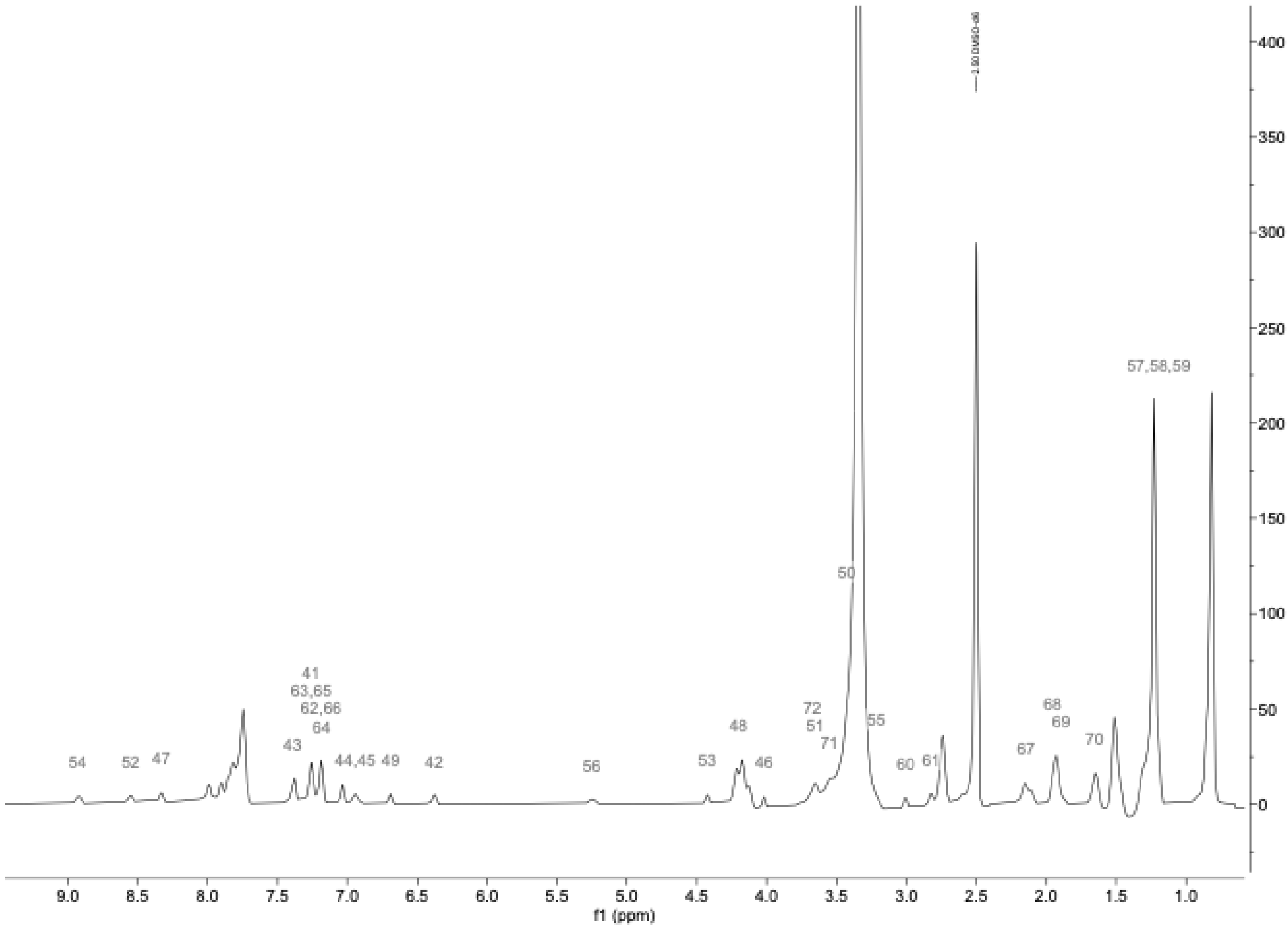

Figure S135. Proton spectrum of cyclic peptide **CP8** at 300 K in anisotropic DMSO-*d6*/OPA at 600 MHz. NS =8, d1= 5 s, AQ = 1.6 s, SW = 16.6 ppm, TD = 32k.

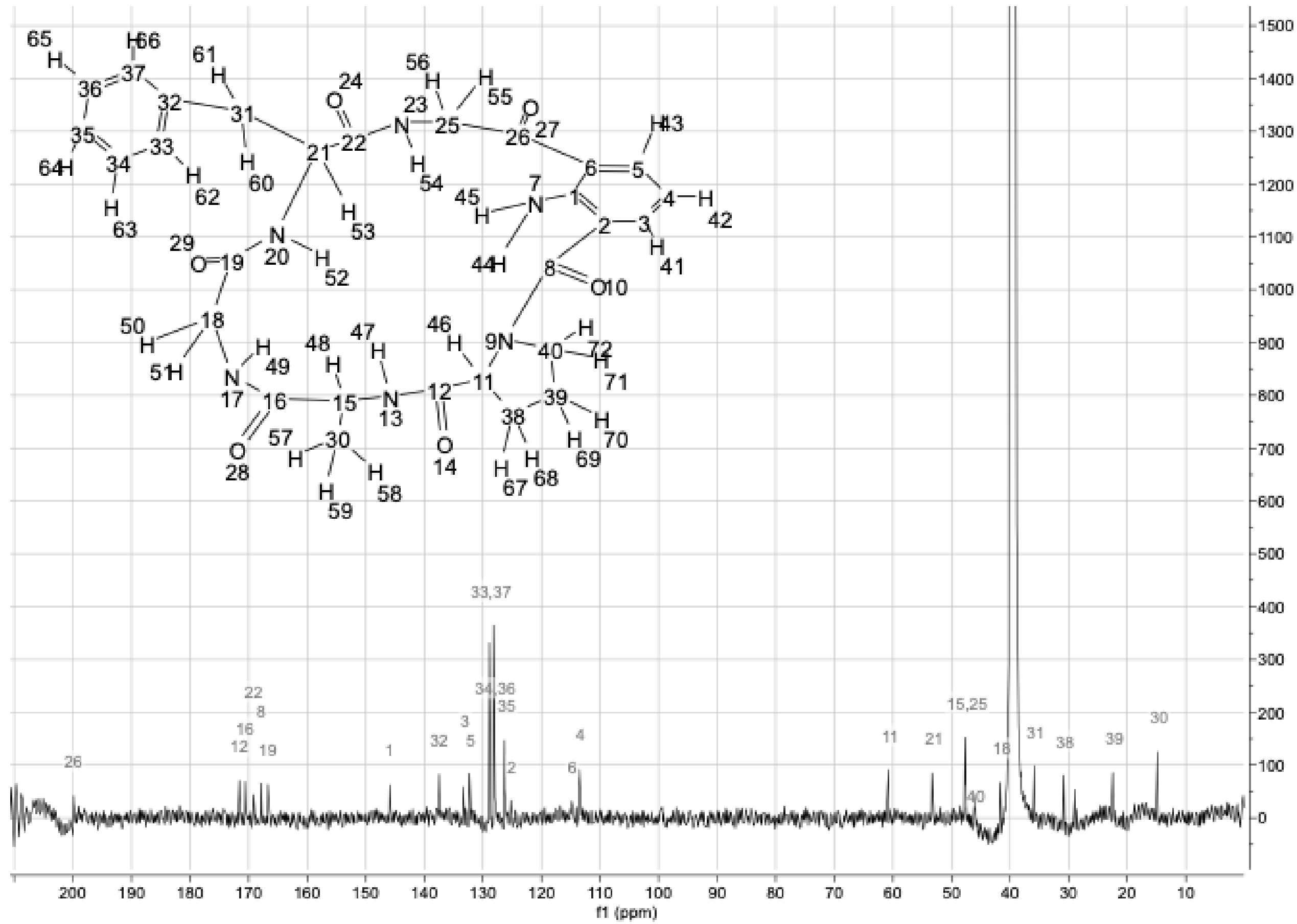

Figure S136. Carbon spectrum of cyclic peptide **CP8** at 300 K in DMSO-*d6* at 600 MHz. NS =1024 (TD0 =26), d1= 2.5 s, AQ = 2.0 s, SW = 212.4 ppm, TD = 128k, 5.0 Hz LB.

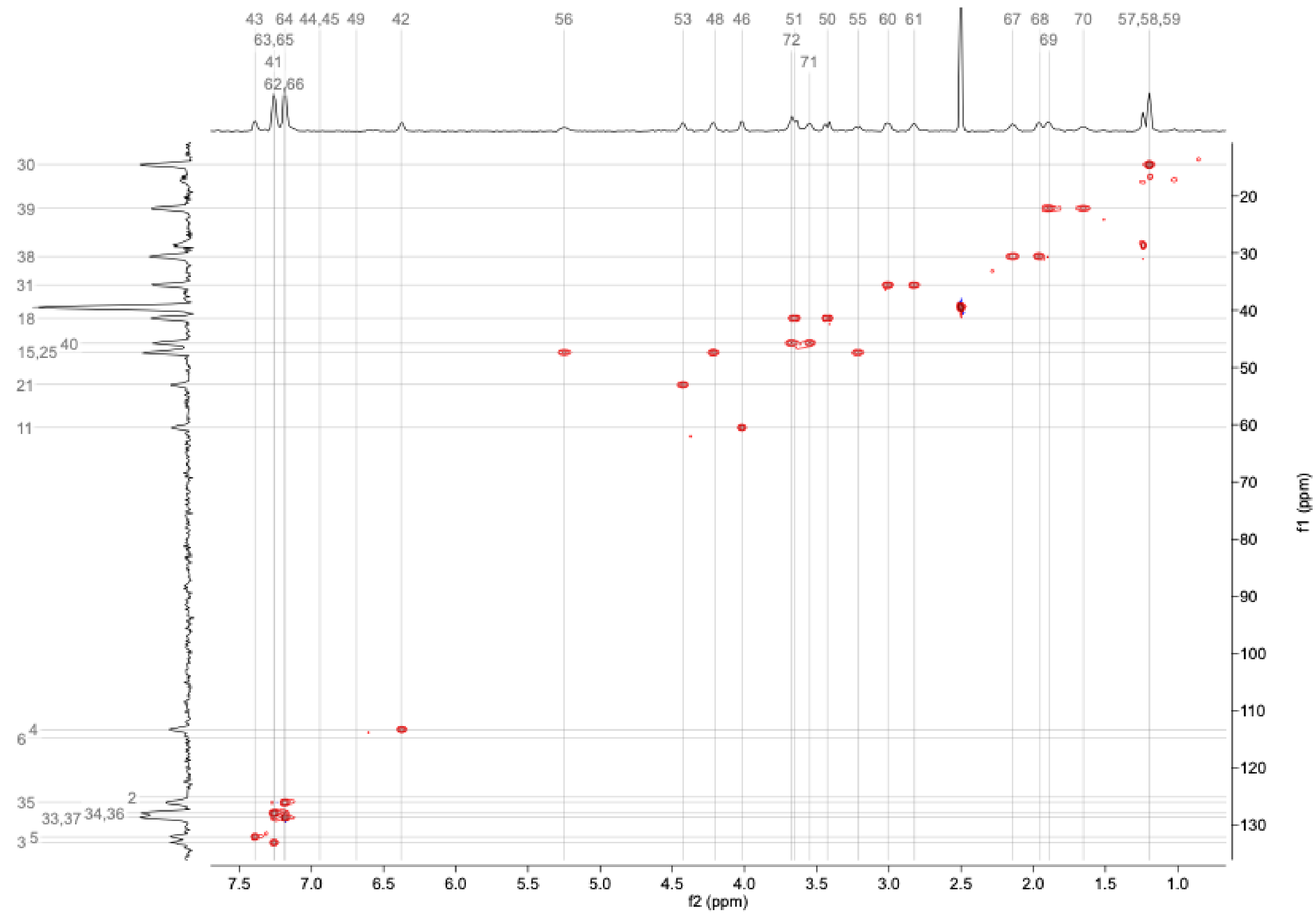

Figure S137. HSQC spectrum of cyclic peptide **CP8** at 300 K in DMSO-*d6* at 600 MHz. NS=32, d1 = 1.3 s, SW 12 (F2) x 160 (F1), TD 2k x 512, echo-antiecho.

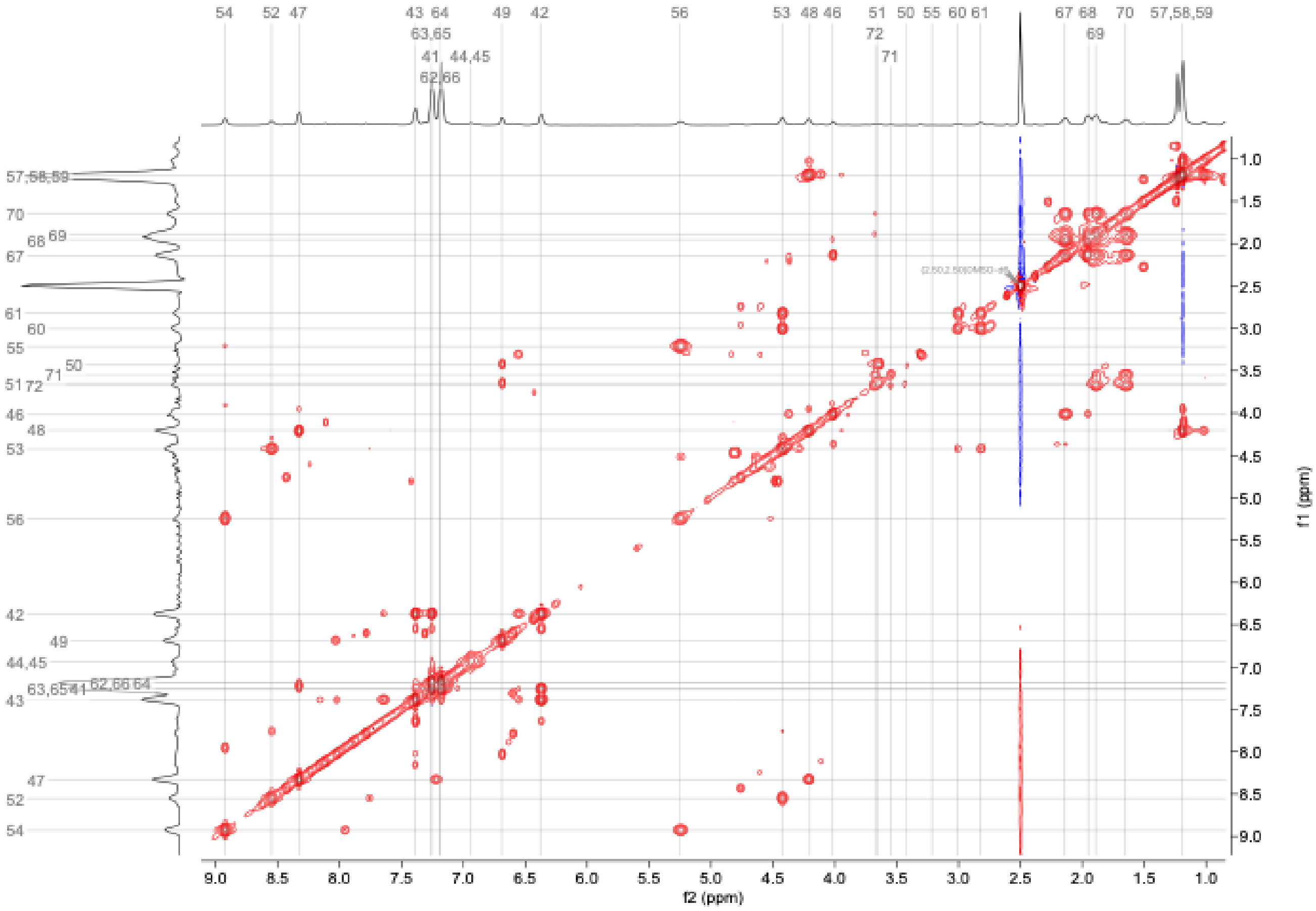

Figure S138. CLIP-COSY spectrum of cyclic peptide **CP8** at 300 K in DMSO-*d6* at 600 MHz. NS=16, d1= 1.3s, SW 16 (F2) x 16 (F1), TD 2k x 512, States-TPPI.

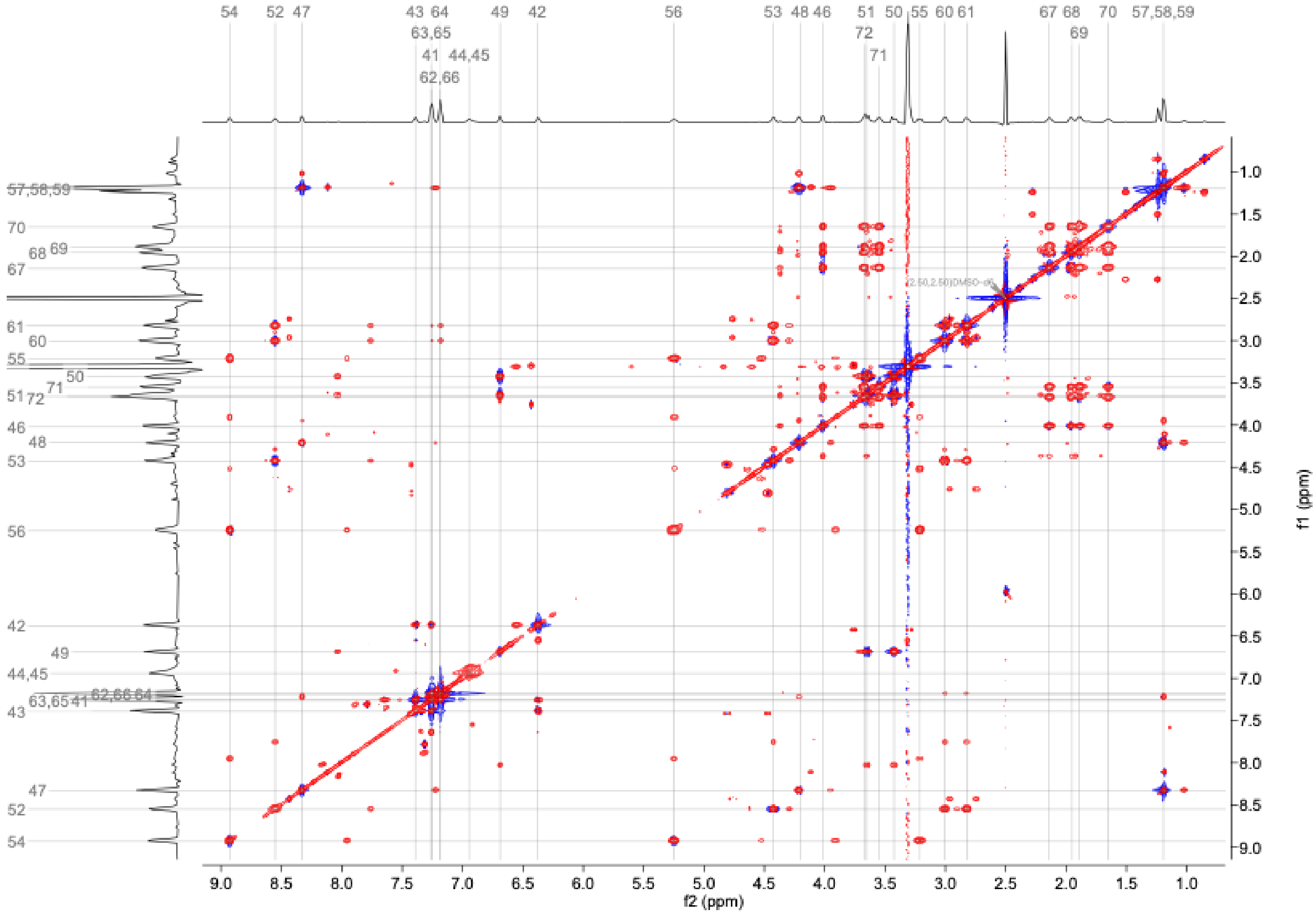

Figure S139. TOCSY spectrum of cyclic peptide **CP8** at 300 K in DMSO-*d6* at 600 MHz. NS=16, d1= 1.3s, SW 14 (F2) x 14 (F1), TD 2k x 1k, States-TPPI, 80 ms mixing time.

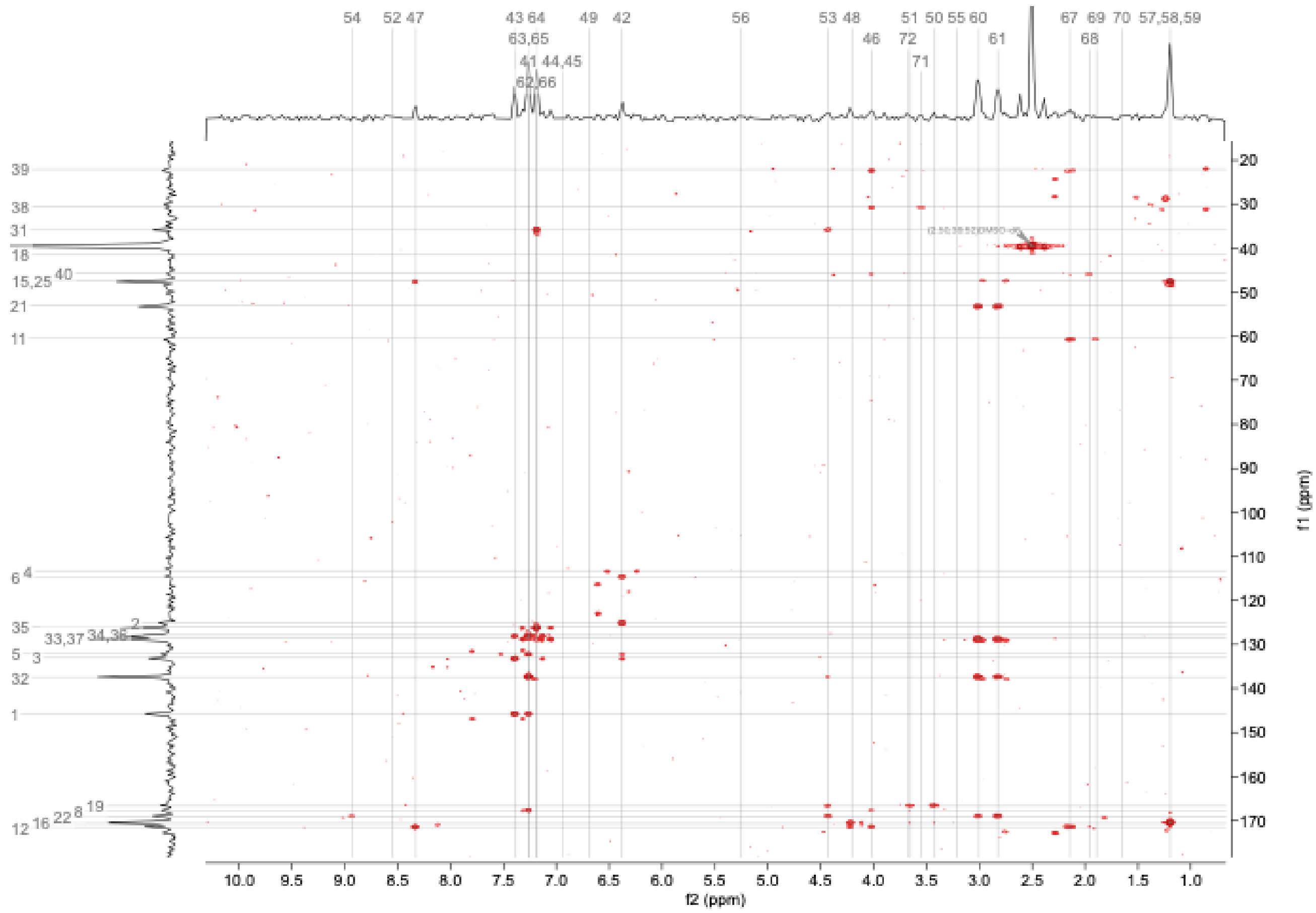

Figure S140. HMBC spectrum of cyclic peptide **CP8** at 300 K in DMSO-*d6* at 600 MHz. NS=32, d1= 1.3s, SW 16.6 (F2) x 207 (F1), TD 2k x 1k, delay for long range coupling 70 ms, echo-antiecho.

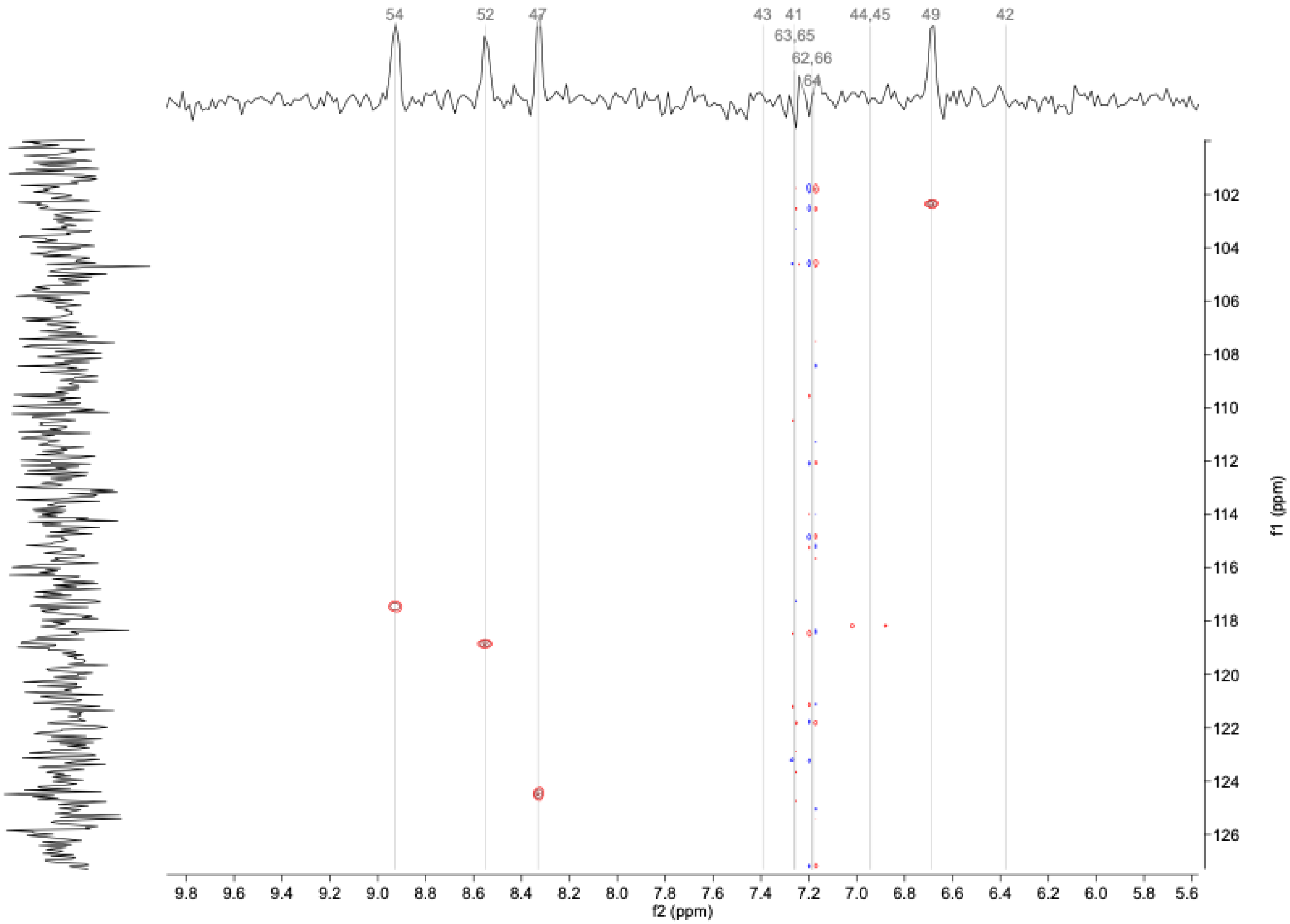

Figure S141. ^{15}N-HSQC spectrum of cyclic peptide **CP8** at 300 K in DMSO-*d6* at 600 MHz. NS=32, d1= 1.3s, SW 14 (F2) x 49.5 (F1), TD 2k x 512, States-TPPI, WaterGATE.

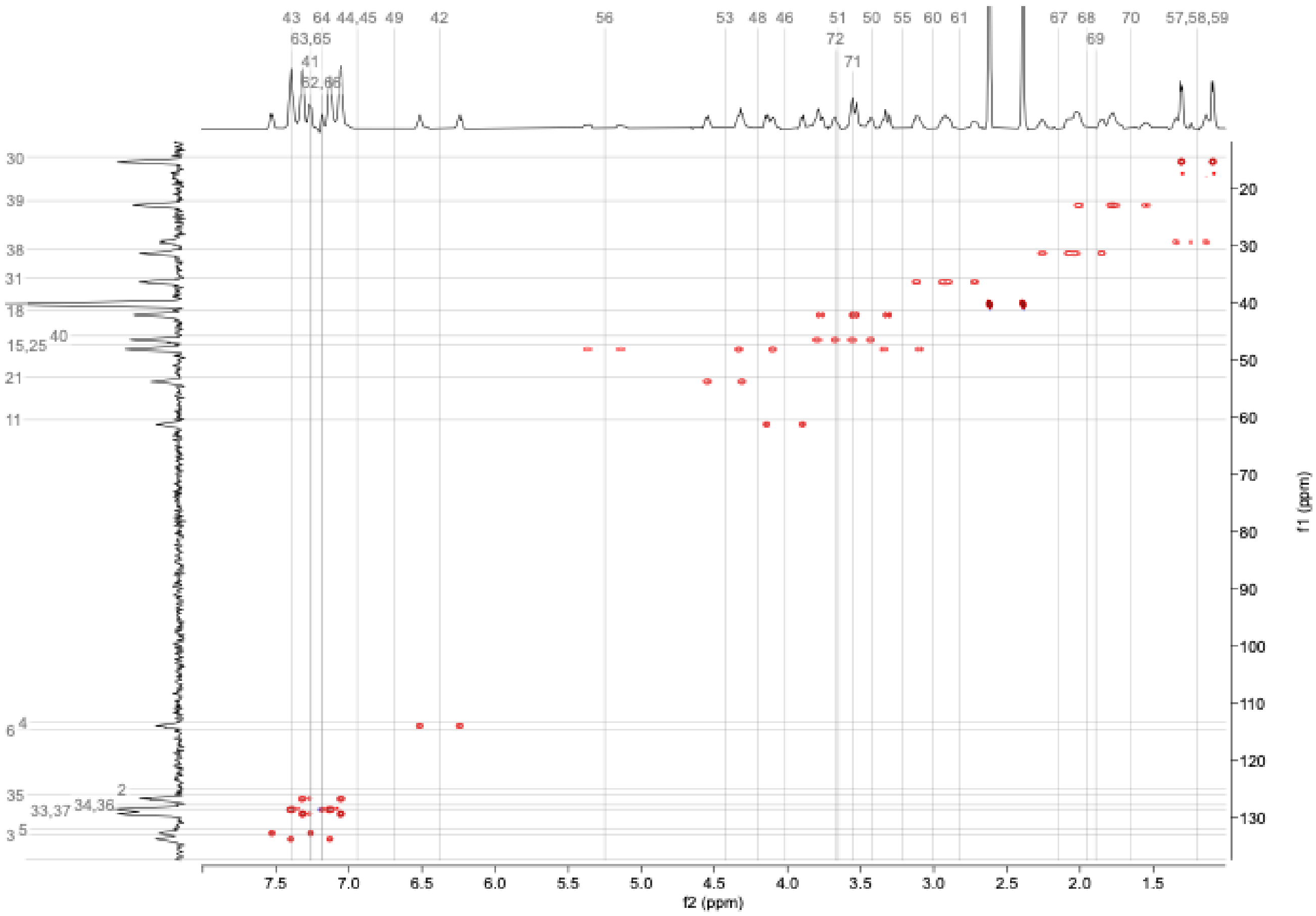

Figure S142. CLIP-HSQC spectrum of cyclic peptide **CP8** at 300 K in DMSO-*d6* at 600 MHz. NS=56, d1 = 1.3 s, SW 11 (F2) x 150 (F1), TD 4k x 512, echo-antiecho, coupling in F2.

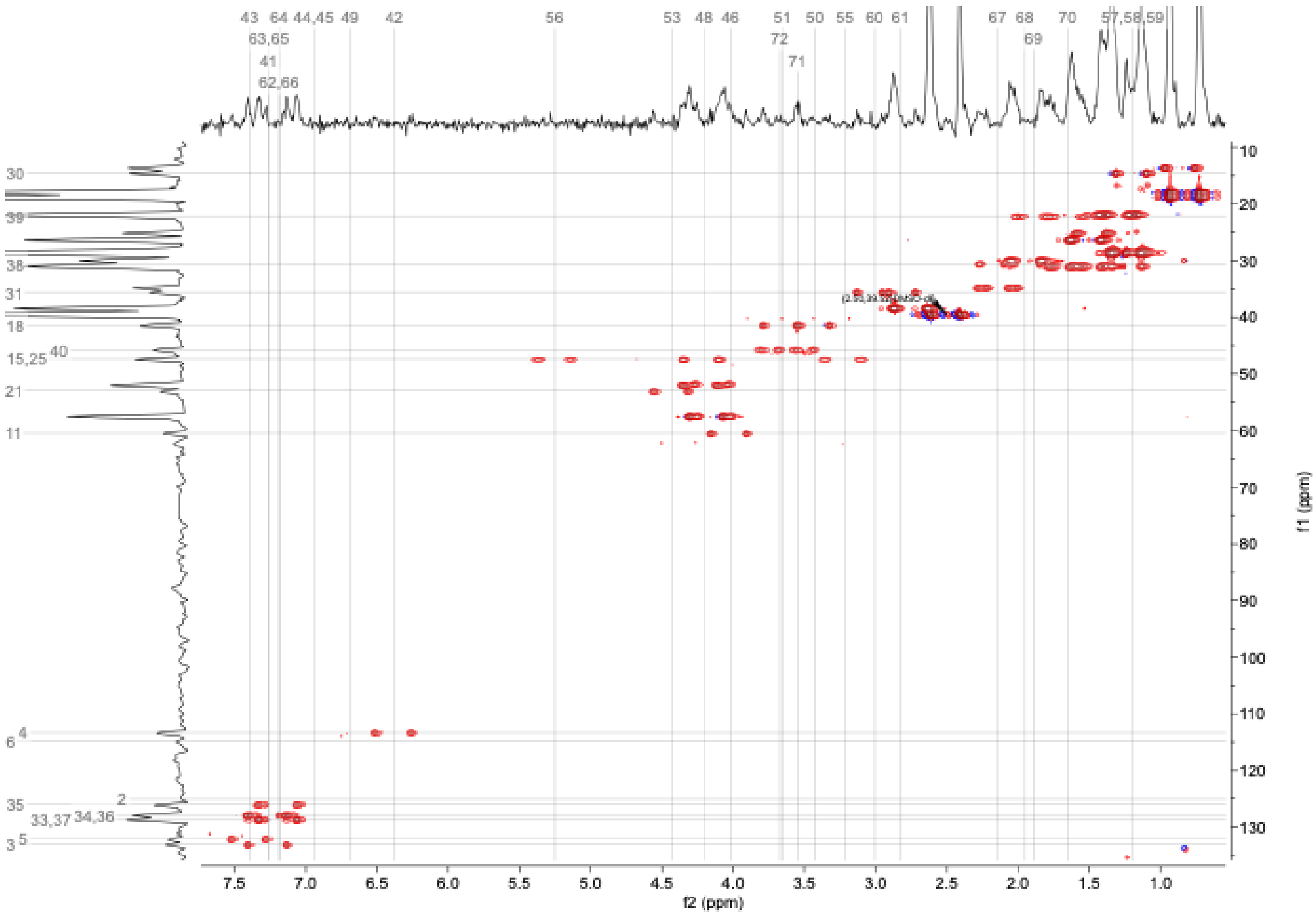

Figure S143. CLIP-HSQC spectrum of cyclic peptide **CP8** at 300 K in DMSO-*d6*/OPA at 600 MHz. NS=64, d1 = 1.3 s, SW 10 (F2) x 150 (F1), TD 4k x 512, echo-antiecho, coupling in F2.

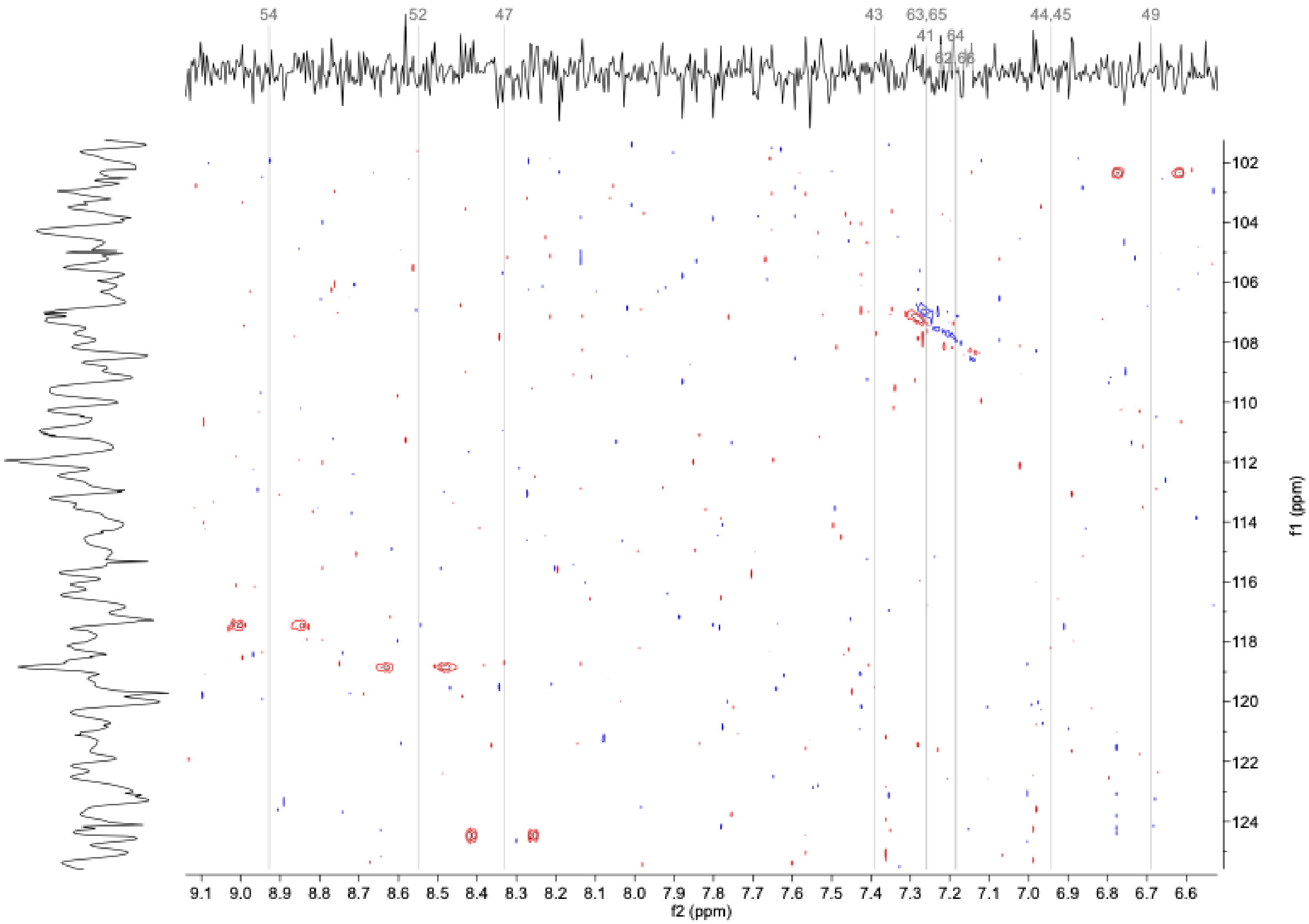

Figure S144. ^{15}N-CLIP-HSQC spectrum of cyclic peptide **CP8** at 300 K in DMSO-*d6* at 600 MHz. NS=160, d1 = 1.3 s, SW 4 (F2) x 30 (F1), TD 2k x 256, echo-antiecho, coupling in F2. WaterGATE.

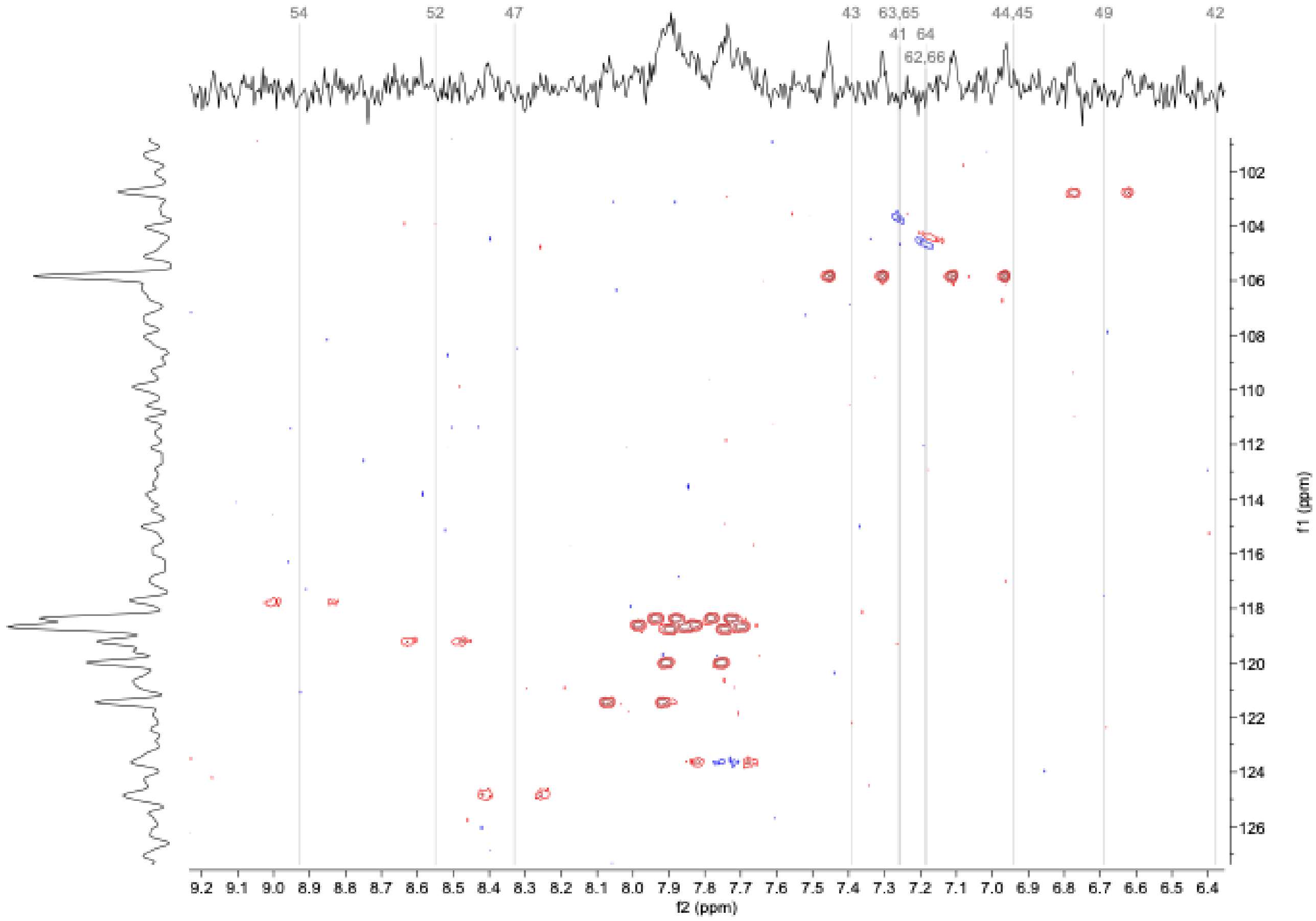

Figure S145. ^{15}N-CLIP-HSQC spectrum of cyclic peptide **CP8** at 300 K in DMSO-*d6*/OPA at 600 MHz. NS=160, d1 = 1.3 s, SW 4 (F2) x 30 (F1), TD 2k x 256, echo-antiecho, coupling in F2. WaterGATE.

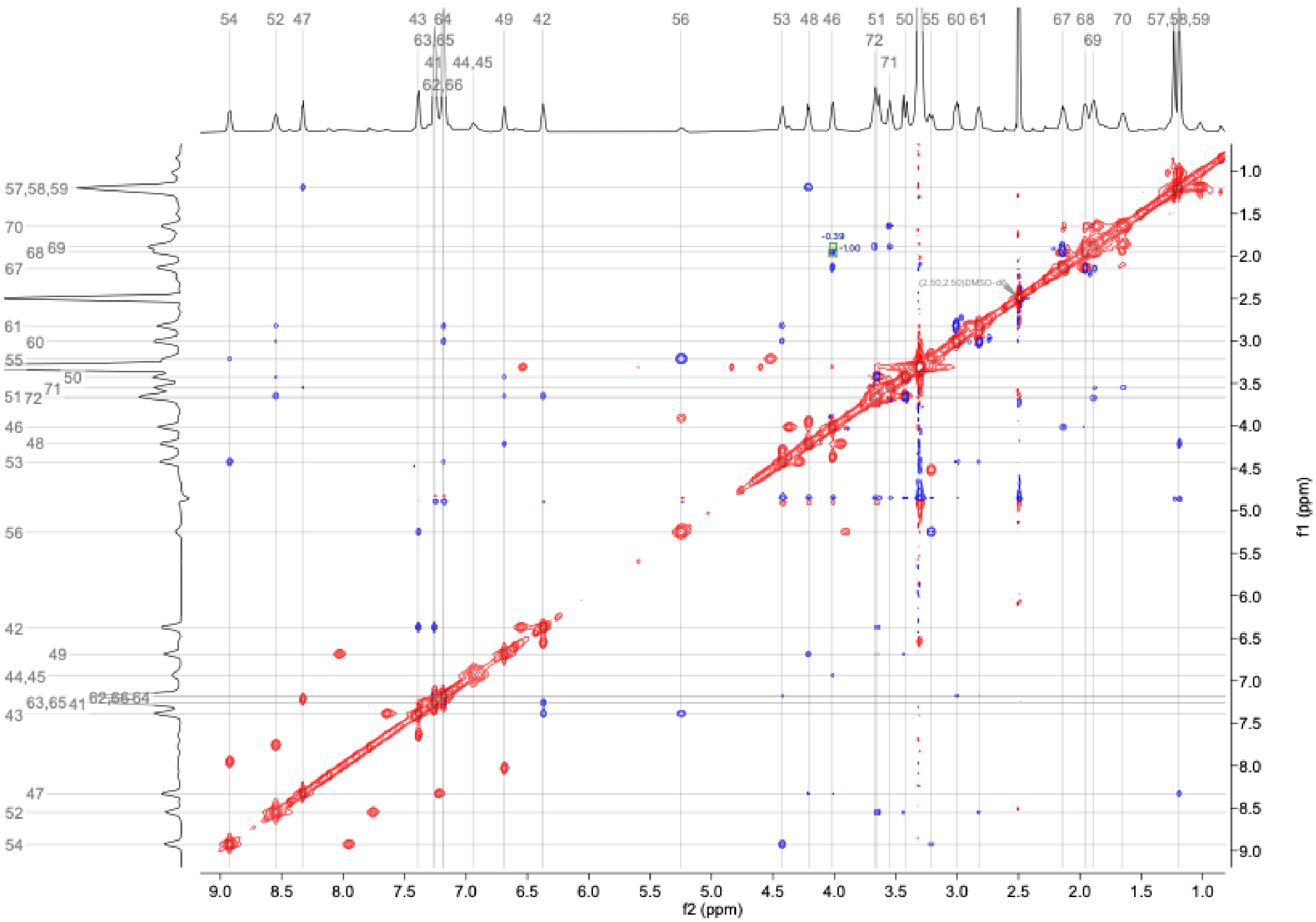

Figure S146. EASY-ROESY spectrum of cyclic peptide **CP8** at 300 K in DMSO-*d6* at 600 MHz. S=24, d1 = 1.3 s, SW 11.9 (F2) x 11.9 (F1), TD 4k x 512, States-TPPI, mixing time = 80 ms.

SI.17 Visualization of principal components of shared PCA

In the following the idealized effects of the principal components on the peptide backbones are shown for **Figure 4 (CP1–CP5)** and **Figure S25** (**CP6–CP8**). For that a reference starting conformer has been modified by the PC1 and PC2 vector separately each by +5 and –5 .

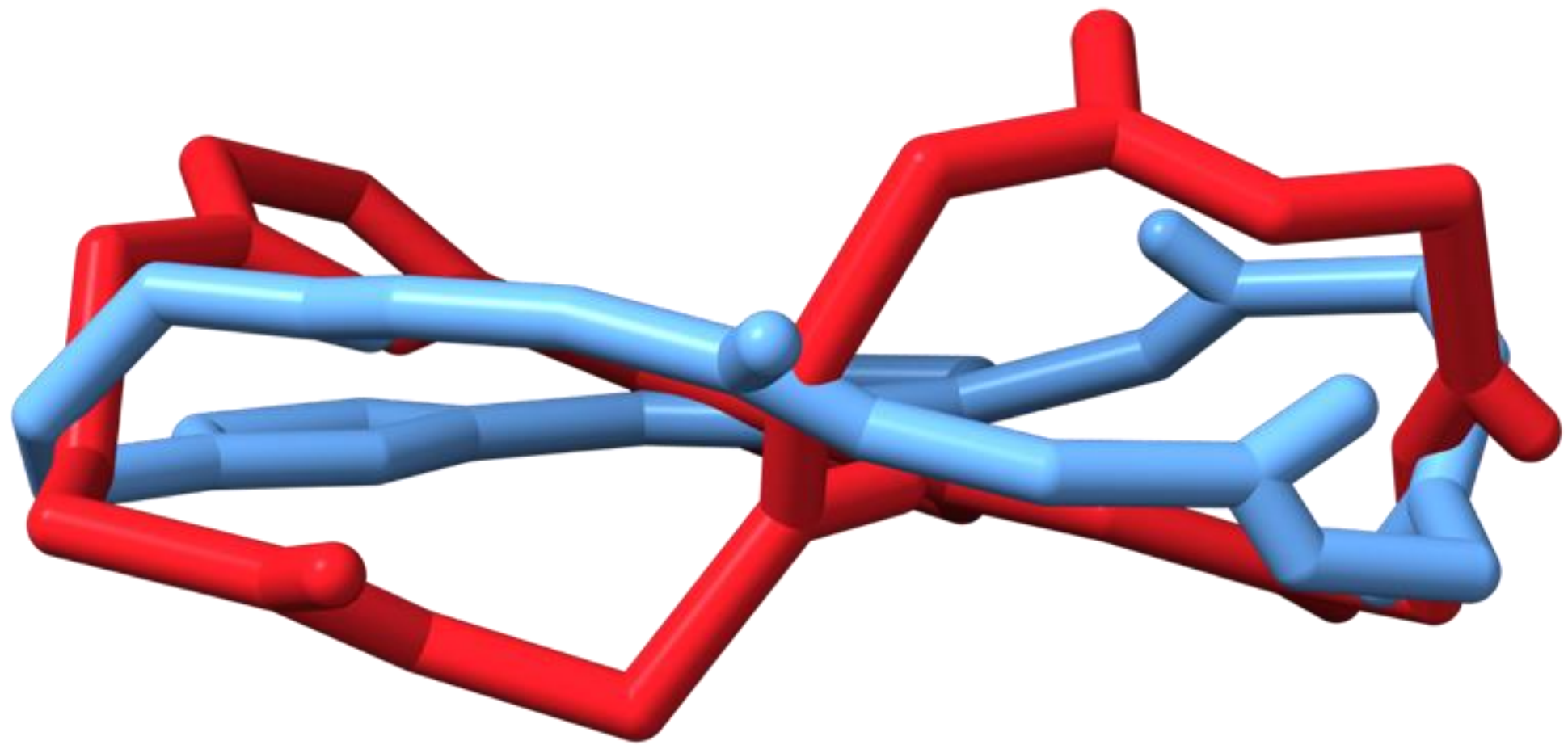

Figure S147. Conformational change of backbone for PC1 of **Figure 4** for **CP1**-**CP5**. Red indicates +5 PC1 and blue -5 PC1.

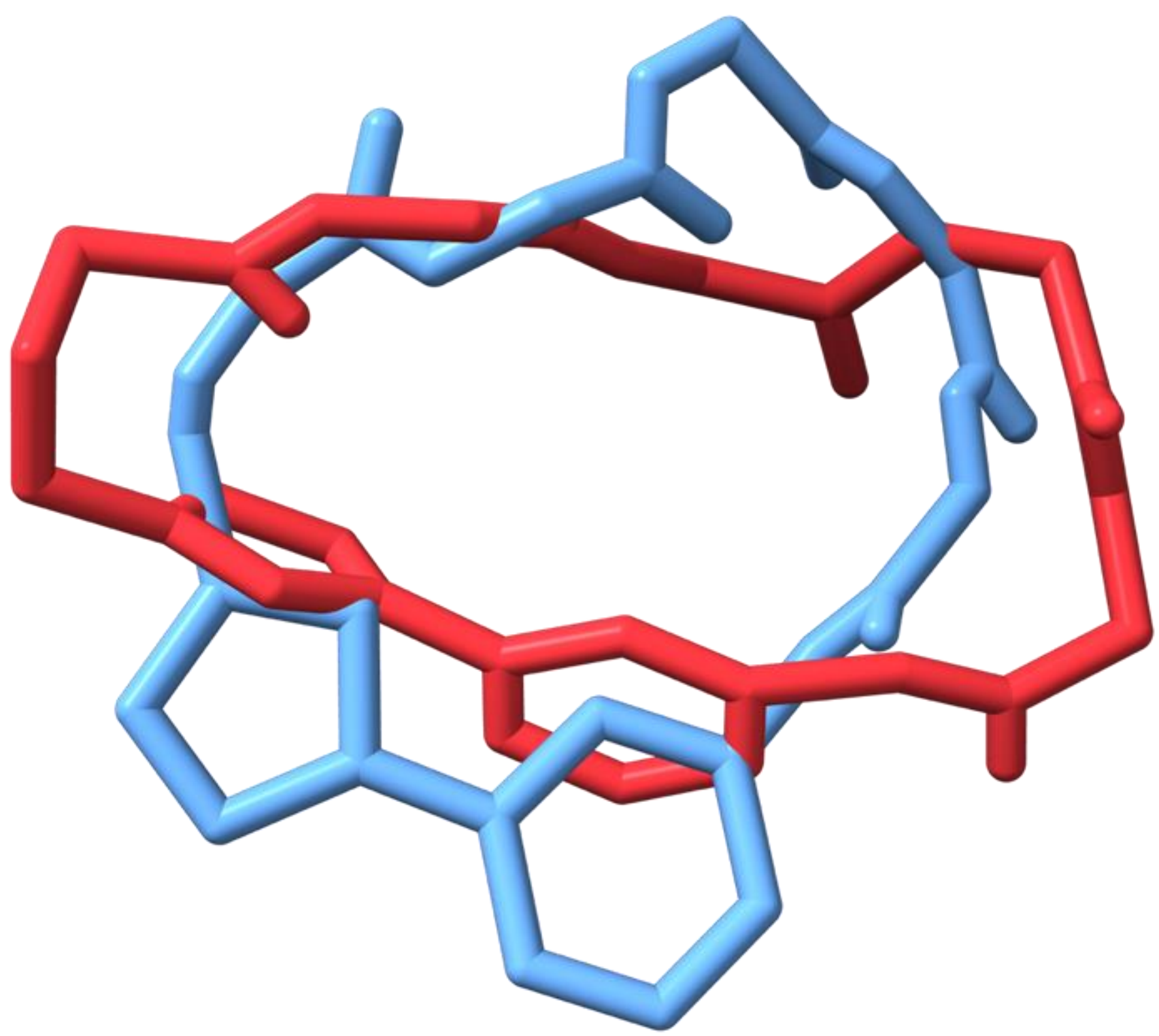

Figure S148. Conformational change of backbone for PC2 of **Figure 4** for **CP1**-**CP5**. Red indicates +5 PC2 and blue -5 PC2.

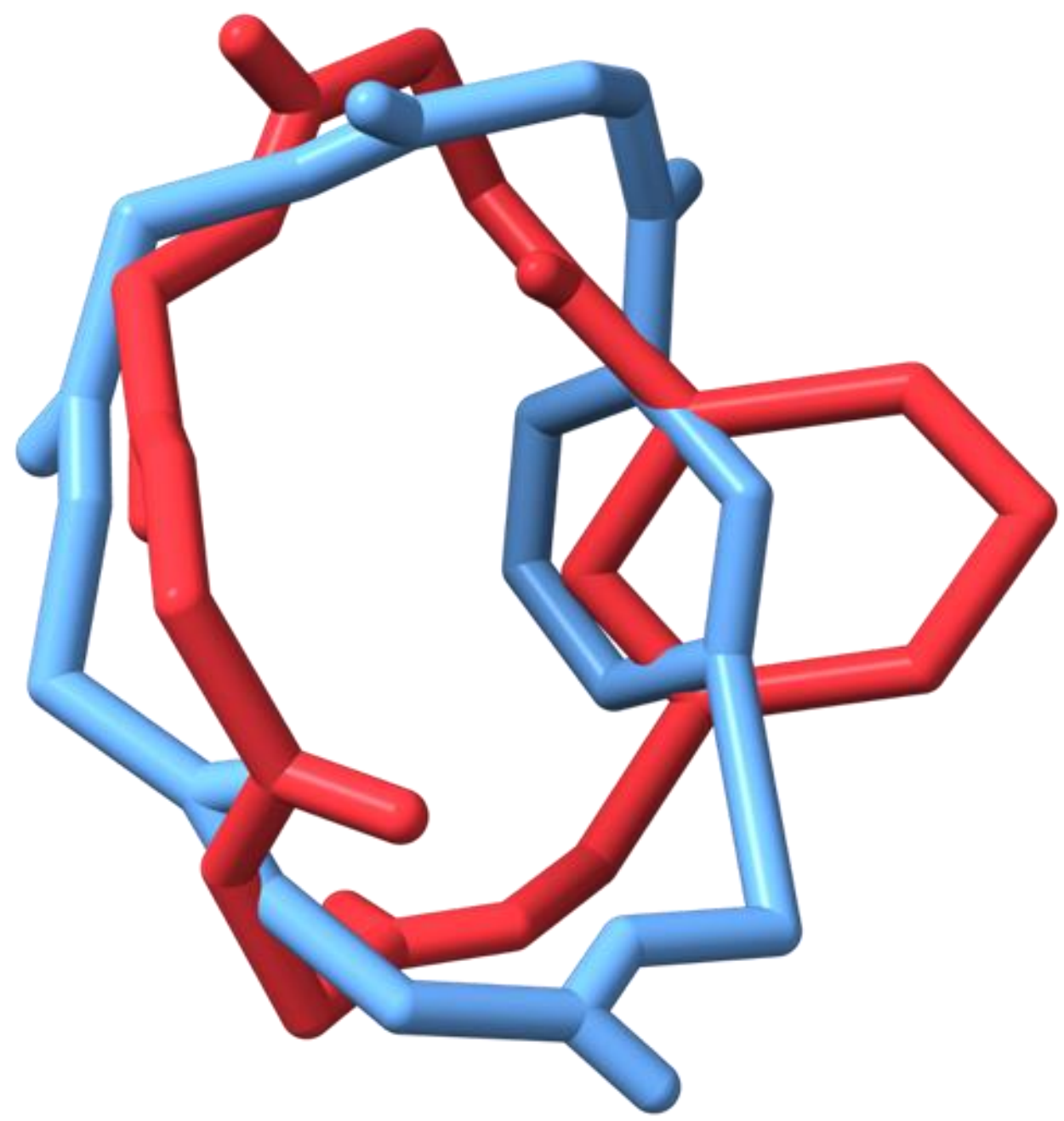

Figure S149. Conformational change of backbone for PC1 of **Figure S25** for **CP6**-**CP8**. Red indicates +5 PC1 and blue -5 PC1.

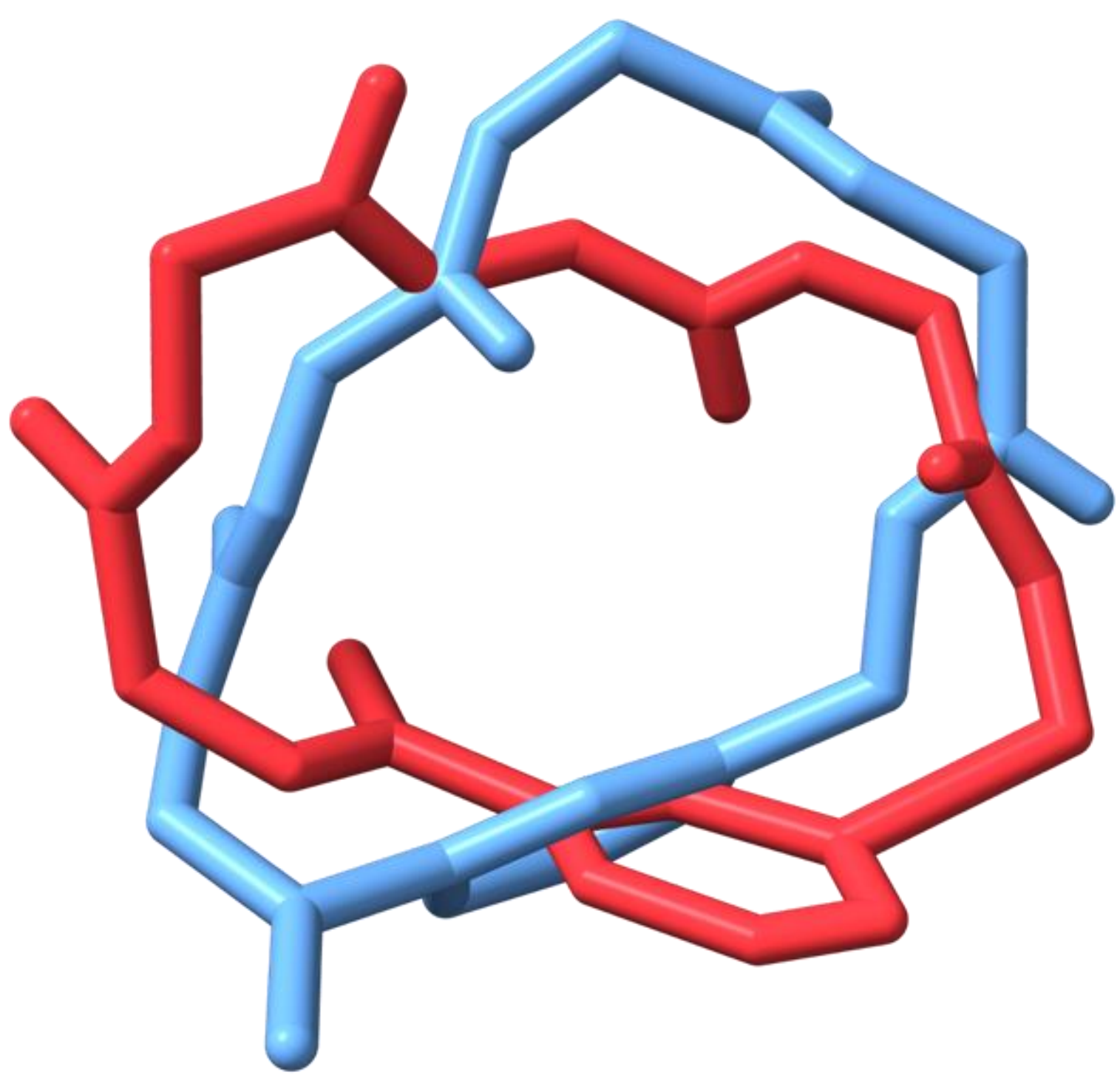

Figure S150. Conformational change of backbone for PC2 of **Figure S25** for **CP6**-**CP8**. Red indicates +5 PC2 and blue -5 PC2.

SI.18 Additional Literature for the SI

(1) Diamandas, M.; Heller, N. W.; Yudin, A. K. Nitrilium Ion Trapping as a Strategy to Access Structurally Diverse Hetero-biaryl-Containing Peptide Macrocycles. *Chem. Sci.* **2023**, *14* (35), 9482–9487. https://doi.org/10.1039/D3SC03058J.

(2) Diamandas, M.; Ou, Y. (Daniel); Huang, B. Z.; Yudin, A. K. Pseudo-Ring Methodology to Control the Conformations of Macrocyclic Peptides. *Chem. – Eur. J.* **2025**, *31* (24), e202500581. https://doi.org/10.1002/chem.202500581.
(3) Ämmälahti, E.; Bardet, M.; Molko, D.; Cadet, J. Evaluation of Distances from ROESY Experiments with the Intensity-Ratio Method. *J. Magn. Reson. A* **1996**, *122* (2), 230–232. https://doi.org/10.1006/jmra.1996.0199.
(4) Thiele, C. M.; Petzold, K.; Schleucher, J. EASY ROESY: Reliable Cross-Peak Integration in Adiabatic Symmetrized ROESY. *Chem. – Eur. J.* **2009**, *15* (3), 585–588. https://doi.org/10.1002/chem.200802027.
(5) Lei, X.; Qiu, F.; Sun, H.; Bai, L.; Wang, W.-X.; Xiang, W.; Xiao, H. A Self-Assembled Oligopeptide as a Versatile NMR Alignment Medium for the Measurement of Residual Dipolar Couplings in Methanol. *Angew. Chem. Int. Ed.* **2017**, *129* (42), 13037–13041. https://doi.org/10.1002/ange.201705123.
(6) Qin, S.-Y.; Jiang, Y.; Sun, H.; Liu, H.; Zhang, A.-Q.; Lei, X. Measurement of Residual Dipolar Couplings of Organic Molecules in Multiple Solvent Systems Using a Liquid-Crystalline-Based Medium. *Angew. Chem. Int. Ed.* **2020**, *59* (39), 17097–17103. https://doi.org/10.1002/anie.202007243.
(7) Schrödinger, LLC. Schrödinger Release 2023-1, 2023. https://www.schrodinger.com.
(8) Grimme, S. Exploration of Chemical Compound, Conformer, and Reaction Space with Meta-Dynamics Simulations Based on Tight-Binding Quantum Chemical Calculations. *J. Chem. Theory Comput.* **2019**, *15* (5), 2847–2862. https://doi.org/10.1021/ACS.JCTC.9B00143.
(9) Pracht, P.; Grimme, S.; Bannwarth, C.; Bohle, F.; Ehlert, S.; Feldmann, G.; Gorges, J.; Müller, M.; Neudecker, T.; Plett, C.; Spicher, S.; Steinbach, P.; Wesołowski, P. A.; Zeller, F. CREST—A Program for the Exploration of Low-Energy Molecular Chemical Space. *J. Chem. Phys.* **2024**, *160* (11), 114110. https://doi.org/10.1063/5.0197592.
(10) Bannwarth, C.; Caldeweyher, E.; Ehlert, S.; Hansen, A.; Pracht, P.; Seibert, J.; Spicher, S.; Grimme, S. Extended Tight-Binding Quantum Chemistry Methods. *Wiley Interdiscip. Rev. Comput. Mol. Sci.* **2021**, *11* (2), e1493. https://doi.org/10.1002/WCMS.1493.
(11) Bannwarth, C.; Ehlert, S.; Grimme, S. GFN2-xTB - An Accurate and Broadly Parametrized Self-Consistent Tight-Binding Quantum Chemical Method with Multipole Electrostatics and Density-Dependent Dispersion Contributions. *J. Chem. Theory Comput.* **2019**, *15* (3), 1652–1671. https://doi.org/10.1021/acs.jctc.8b01176.
(12) Ehlert, S.; Stahn, M.; Spicher, S.; Grimme, S. Robust and Efficient Implicit Solvation Model for Fast Semiempirical Methods. *J. Chem. Theory Comput.* **2021**, *17* (7), 4250–4261. https://doi.org/10.1021/acs.jctc.1c00471.
(13) Grimme, S.; Bohle, F.; Hansen, A.; Pracht, P.; Spicher, S.; Stahn, M. Efficient Quantum Chemical Calculation of Structure Ensembles and Free Energies for Nonrigid Molecules. *J. Phys. Chem. A* **2021**, *125* (19), 4039–4054. https://doi.org/10.1021/ACS.JPCA.1C00971.
(14) Neese, F. Software Update: The ORCA Program System—Version 6.0. *WIREs Comput. Mol. Sci.* **2025**, *15* (2), e70019. https://doi.org/10.1002/wcms.70019.
(15) Neese, F.; Wennmohs, F.; Becker, U.; Riplinger, C. The ORCA Quantum Chemistry Program Package. *J. Chem. Phys.* **2020**, *152* (22), 224108. https://doi.org/10.1063/5.0004608.
(16) Neese, F. Software Update: The ORCA Program System—Version 5.0. *Wiley Interdiscip. Rev. Comput. Mol. Sci.* **2022**, *12* (5), e1606. https://doi.org/10.1002/WCMS.1606.
(17) Neese, F. The ORCA Program System. *Wiley Interdiscip. Rev. Comput. Mol. Sci.* **2012**, *2* (1), 73–78. https://doi.org/10.1002/WCMS.81.
(18) Stephens, P. J.; Devlin, F. J.; Chabalowski, C. F.; Frisch, M. J. Ab Initio Calculation of Vibrational Absorption and Circular Dichroism Spectra Using Density Functional Force Fields. *J. Phys. Chem.* **1994**, *98*, 11623–11627. https://doi.org/10.1021/j100096a001.
(19) Weigend, F.; Ahlrichs, R. Balanced Basis Sets of Split Valence, Triple Zeta Valence and Quadruple Zeta Valence Quality for H to Rn: Design and Assessment of Accuracy. *Phys. Chem. Chem. Phys.* **2005**, *7* (18), 3297–3305. https://doi.org/10.1039/B508541A.
(20) Caldeweyher, E.; Mewes, J. M.; Ehlert, S.; Grimme, S. Extension and Evaluation of the D4 London-Dispersion Model for Periodic Systems. *Phys. Chem. Chem. Phys.* **2020**, *22* (16), 8499–8512. https://doi.org/10.1039/D0CP00502A.
(21) Marenich, A. V.; Cramer, C. J.; Truhlar, D. G. Universal Solvation Model Based on Solute Electron Density and on a Continuum Model of the Solvent Defined by the Bulk Dielectric Constant and Atomic Surface Tensions. *J. Phys. Chem. B* **2009**, *113* (18), 6378–6396. https://doi.org/10.1021/JP810292N.
(22) Zhao, Y.; Truhlar, D. G. Design of Density Functionals That Are Broadly Accurate for Thermochemistry, Thermochemical Kinetics, and Nonbonded Interactions. *J. Phys. Chem. A* **2005**, *109*, 5656–5667. https://doi.org/10.1021/jp050536c.
(23) Bursch, M.; Neugebauer, H.; Ehlert, S.; Grimme, S. Dispersion Corrected r2SCAN Based Global Hybrid Functionals: r2SCANh, r2SCAN0, and r2SCAN50. *J. Chem. Phys.* **2022**, *156* (13), 134105. https://doi.org/10.1063/5.0086040.
(24) Jensen, F. Segmented Contracted Basis Sets Optimized for Nuclear Magnetic Shielding. *J. Chem. Theory Comput.* **2015**, *11* (1), 132–138. https://doi.org/10.1021/ct5009526.
(25) Dobson, J. F. Alternative Expressions for the Fermi Hole Curvature. *J. Chem. Phys.* **1993**, *98*, 8870–8872. https://doi.org/10.1063/1.464444.
(26) Feyereisen, M.; Fitzgerald, G.; Komornicki, A. Use of Approximate Integrals in Ab Initio Theory. An Application in MP2 Energy Calculations. *Chem. Phys. Lett.* **1993**, *208* (5), 359–363. https://doi.org/10.1016/0009-2614(93)87156-W.

(27) Barone, V.; Cossi, M. Quantum Calculation of Molecular Energies and Energy Gradients in Solution by a Conductor Solvent Model. *J. Phys. Chem. A* **1998**, *102* (11), 1995–2001. https://doi.org/10.1021/jp9716997.
(28) Kozuch, S.; Martin, J. M. L. DSD-PBEP86: In Search of the Best Double-Hybrid DFT with Spin-Component Scaled MP2 and Dispersion Corrections. *Phys. Chem. Chem. Phys.* **2011**, *13* (45), 20104–20107. https://doi.org/10.1039/C1CP22592H.
(29) Goerigk, L.; Hansen, A.; Bauer, C.; Ehrlich, S.; Najibi, A.; Grimme, S. A Look at the Density Functional Theory Zoo with the Advanced GMTKN55 Database for General Main Group Thermochemistry, Kinetics and Noncovalent Interactions. *Phys. Chem. Chem. Phys.* **2017**, *19* (48), 32184–32215. https://doi.org/10.1039/C7CP04913G.
(30) Ketzel, A. F.; Hu, Y.; Li, X.-L.; Li, J.; Lei, X.; Sun, H. Heterophyllin B: Combining Isotropic and Anisotropic NMR for the Conformational Analysis of a Natural Occurring Cyclic Peptide. *Magn. Reson. Chem.* **2025**, *63* (5–6), 417–423. https://doi.org/10.1002/mrc.5523.
(31) Cohen, R. D.; Wood, J. S.; Lam, Y.-H.; Buevich, A. V.; Sherer, E. C.; Reibarkh, M.; Williamson, R. T.; Martin, G. E. DELTA50: A Highly Accurate Database of Experimental 1H and 13C NMR Chemical Shifts Applied to DFT Benchmarking. *Molecules* **2023**, *28* (6), 2449. https://doi.org/10.3390/molecules28062449.
(32) Fulmer, G. R.; Miller, A. J. M.; Sherden, N. H.; Gottlieb, H. E.; Nudelman, A.; Stoltz, B. M.; Bercaw, J. E.; Goldberg, K. I. NMR Chemical Shifts of Trace Impurities: Common Laboratory Solvents, Organics, and Gases in Deuterated Solvents Relevant to the Organometallic Chemist. *Organometallics* **2010**, *29* (9), 2176–2179. https://doi.org/10.1021/OM100106E.
(33) Navarro-Vázquez, A. *Stereofitter* version 1.17; Mestrelab Research: Santiago de Compostela, Spain, **2024**. https://mestrelab.com/main-product/stereo-fitter (accessed 2025-07-18).
(34) Pardi, A.; Billeter, M.; Wüthrich, K. Calibration of the Angular Dependence of the Amide Proton-Cα Proton Coupling Constants, 3*J*HNα, in a Globular Protein. *J. Mol. Biol.* **1984**, *180* (3), 741–751. https://doi.org/10.1016/0022-2836(84)90035-4.
(35) Ásgeirsson, V.; Birgisson, B. O.; Bjornsson, R.; Becker, U.; Neese, F.; Riplinger, C.; Jónsson, H. Nudged Elastic Band Method for Molecular Reactions Using Energy-Weighted Springs Combined with Eigenvector Following. *J. Chem. Theory Comput.* **2021**, *17* (8), 4929–4945. https://doi.org/10.1021/acs.jctc.1c00462.
(36) Lindahl, E.; Hess, B.; van der Spoel, D. GROMACS 3.0: A Package for Molecular Simulation and Trajectory Analysis. *Mol. Model. Annu.* **2001**, *7* (8), 306–317. https://doi.org/10.1007/s008940100045.
(37) Hess, B.; Kutzner, C.; van der Spoel, D.; Lindahl, E. GROMACS 4: Algorithms for Highly Efficient, Load-Balanced, and Scalable Molecular Simulation. *J. Chem. Theory Comput.* **2008**, *4* (3), 435–447. https://doi.org/10.1021/ct700301q.
(38) Pronk, S.; Páll, S.; Schulz, R.; Larsson, P.; Bjelkmar, P.; Apostolov, R.; Shirts, M. R.; Smith, J. C.; Kasson, P. M.; van der Spoel, D.; Hess, B.; Lindahl, E. GROMACS 4.5: A High-Throughput and Highly Parallel Open Source Molecular Simulation Toolkit. *Bioinforma. Oxf. Engl.* **2013**, *29* (7), 845–854. https://doi.org/10.1093/bioinformatics/btt055.
(39) Berendsen, H. J. C.; van der Spoel, D.; van Drunen, R. GROMACS: A Message-Passing Parallel Molecular Dynamics Implementation. *Comput. Phys. Commun.* **1995**, *91* (1), 43–56. https://doi.org/10.1016/0010-4655(95)00042-E.
(40) Van Der Spoel, D.; Lindahl, E.; Hess, B.; Groenhof, G.; Mark, A. E.; Berendsen, H. J. C. GROMACS: Fast, Flexible, and Free. *J. Comput. Chem.* **2005**, *26* (16), 1701–1718. https://doi.org/10.1002/jcc.20291.
(41) Abraham, M. J.; Murtola, T.; Schulz, R.; Páll, S.; Smith, J. C.; Hess, B.; Lindahl, E. GROMACS: High Performance Molecular Simulations through Multi-Level Parallelism from Laptops to Supercomputers. *SoftwareX* **2015**, *1–2*, 19–25. https://doi.org/10.1016/j.softx.2015.06.001.
(42) Páll, S.; Abraham, M. J.; Kutzner, C.; Hess, B.; Lindahl, E. Tackling Exascale Software Challenges in Molecular Dynamics Simulations with GROMACS. In *Solving Software Challenges for Exascale*; Markidis, S., Laure, E., Eds.; Lecture Notes in Computer Science, Vol. 8759; Springer: Cham, **2015**; pp 3–27. https://doi.org/10.1007/978-3-319-15976-8_1.
(43) Wang, J.; Wolf, R. M.; Caldwell, J. W.; Kollman, P. A.; Case, D. A. Development and Testing of a General Amber Force Field. *J. Comput. Chem.* **2004**, *25* (9), 1157–1174. https://doi.org/10.1002/jcc.20035.
(44) Frisch, M. J.; Trucks, G. W.; Schlegel, H. B.; Scuseria, G. E.; Robb, M. A.; Cheeseman, J. R.; Scalmani, G.; Barone, V.; Petersson, G. A.; Nakatsuji, H.; Li, X.; Caricato, M.; Marenich, A. V.; Bloino, J.; Janesko, B. G.; Gomperts, R.; Mennucci, B.; Hratchian, H. P.; Ortiz, J. V.; Izmaylov, A. F.; Sonnenberg, J. L.; Williams-Young, D.; Ding, F.; Lipparini, F.; Egidi, F.; Goings, J.; Peng, B.; Petrone, A.; Henderson, T.; Ranasinghe, D.; Zakrzewski, V. G.; Gao, J.; Rega, N.; Zheng, G.; Liang, W.; Hada, M.; Ehara, M.; Toyota, K.; Fukuda, R.; Hasegawa, J.; Ishida, M.; Nakajima, T.; Honda, Y.; Kitao, O.; Nakai, H.; Vreven, T.; Throssell, K.; Montgomery, J. A., Jr.; Peralta, J. E.; Ogliaro, F.; Bearpark, M. J.; Heyd, J. J.; Brothers, E. N.; Kudin, K. N.; Staroverov, V. N.; Keith, T. A.; Kobayashi, R.; Normand, J.; Raghavachari, K.; Rendell, A. P.; Burant, J. C.; Iyengar, S. S.; Tomasi, J.; Cossi, M.; Millam, J. M.; Klene, M.; Adamo, C.; Cammi, R.; Ochterski, J. W.; Martin, R. L.; Morokuma, K.; Farkas, O.; Foresman, J. B.; Fox, D. J. Gaussian 16 Revision C.01, 2016.
(45) Wang, J.; Wolf, R. M.; Caldwell, J. W.; Kollman, P. A.; Case, D. A. Development and Testing of a General Amber Force Field. *J. Comput. Chem.* **2004**, *25* (9), 1157–1174. https://doi.org/10.1002/jcc.20035.
(46) Wang, J.; Wang, W.; Kollman, P. A.; Case, D. A. Automatic Atom Type and Bond Type Perception in Molecular Mechanical Calculations. *J. Mol. Graph. Model.* **2006**, *25* (2), 247–260. https://doi.org/10.1016/j.jmgm.2005.12.005.
(47) Tribello, G. A.; Bonomi, M.; Branduardi, D.; Camilloni, C.; Bussi, G. PLUMED 2: New Feathers for an Old Bird. *Comput. Phys. Commun.* **2014**, *185* (2), 604–613. https://doi.org/10.1016/j.cpc.2013.09.018.

(48) Bonomi, M.; Branduardi, D.; Bussi, G.; Camilloni, C.; Provasi, D.; Raiteri, P.; Donadio, D.; Marinelli, F.; Pietrucci, F.; Broglia, R. A.; Parrinello, M. PLUMED: A Portable Plugin for Free-Energy Calculations with Molecular Dynamics. *Comput. Phys. Commun.* **2009**, *180* (10), 1961–1972. https://doi.org/10.1016/j.cpc.2009.05.011.

(49) Bonomi, M.; Bussi, G.; Camilloni, C.; Tribello, G. A.; Banáš, P.; Barducci, A.; Bernetti, M.; Bolhuis, P. G.; Bottaro, S.; Branduardi, D.; Capelli, R.; Carloni, P.; Ceriotti, M.; Cesari, A.; Chen, H.; Chen, W.; Colizzi, F.; De, S.; De La Pierre, M.; Donadio, D.; Drobot, V.; Ensing, B.; Ferguson, A. L.; Filizola, M.; Fraser, J. S.; Fu, H.; Gasparotto, P.; Gervasio, F. L.; Giberti, F.; Gil-Ley, A.; Giorgino, T.; Heller, G. T.; Hocky, G. M.; Iannuzzi, M.; Invernizzi, M.; Jelfs, K. E.; Jussupow, A.; Kirilin, E.; Laio, A.; Limongelli, V.; Lindorff-Larsen, K.; Löhr, T.; Marinelli, F.; Martin-Samos, L.; Masetti, M.; Meyer, R.; Michaelides, A.; Molteni, C.; Morishita, T.; Nava, M.; Paissoni, C.; Papaleo, E.; Parrinello, M.; Pfaendtner, J.; Piaggi, P.; Piccini, G.; Pietropaolo, A.; Pietrucci, F.; Pipolo, S.; Provasi, D.; Quigley, D.; Raiteri, P.; Raniolo, S.; Rydzewski, J.; Salvalaglio, M.; Sosso, G. C.; Spiwok, V.; Šponer, J.; Swenson, D. W. H.; Tiwary, P.; Valsson, O.; Vendruscolo, M.; Voth, G. A.; White, A.; The PLUMED consortium. Promoting Transparency and Reproducibility in Enhanced Molecular Simulations. *Nat. Methods* **2019**, *16* (8), 670–673. https://doi.org/10.1038/s41592-019-0506-8.
